# Unraveling local tissue changes within severely injured skeletal muscles in response to MSC-based intervention using MALDI Imaging mass spectrometry

**DOI:** 10.1038/s41598-018-30990-w

**Published:** 2018-08-23

**Authors:** Oliver Klein, Kristin Strohschein, Grit Nebrich, Michael Fuchs, Herbert Thiele, Patrick Giavalisco, Georg N. Duda, Tobias Winkler, Jan Hendrik Kobarg, Dennis Trede, Sven Geissler

**Affiliations:** 1 Berlin-Brandenburg Center for Regenerative Therapies, Charité – Universitätsmedizin Berlin, Augustenburger Platz 1, 13353 Berlin, Germany; 2 Julius Wolff Institute & Center for Musculoskeletal Surgery, Charité – Universitätsmedizin Berlin, Augustenburger Platz 1, 13353 Berlin, Germany; 3 Experimental Systems Biology Max-Planck-Institute of Molecular Plant Physiology, Am Mühlenberg, 14476 Golm, Germany; 4 Fraunhofer - Inst. Medical Image Computing MEVIS, Maria-Goeppert-Straße 3, 23562 Lübeck, Germany; 5 SCiLS, Zweigniederlassung Bremen der Bruker Daltonik, Fahrenheitstr. 1, 28359 Bremen, Germany

## Abstract

Pre-clinical and clinical studies are now beginning to demonstrate the high potential of cell therapies in enhancing muscle regeneration. We previously demonstrated functional benefit after the transplantation of autologous bone marrow mesenchymal stromal cells (MSC-TX) into a severe muscle crush trauma model. Despite our increasing understanding of the molecular and cellular mechanisms underlying MSC’s regenerative function, little is known about the local molecular alterations and their spatial distribution within the tissue after MSC-TX. Here, we used MALDI imaging mass spectrometry (MALDI-IMS) in combination with multivariate statistical strategies to uncover previously unknown peptide alterations within severely injured skeletal muscles. Our analysis revealed that very early molecular alterations in response to MSC-TX occur largely in the region adjacent to the trauma and only to a small extent in the actual trauma region. Using “bottom up” mass spectrometry, we subsequently identified the proteins corresponding to the differentially expressed peptide intensity distributions in the specific muscle regions and used immunohistochemistry to validate our results. These findings extend our current understanding about the early molecular processes of muscle healing and highlights the critical role of trauma adjacent tissue during the early therapeutic response upon treatment with MSC.

## Introduction

Skeletal muscles have a significant regenerative potential. However, these endogenous processes are often insufficient to recover from severe injuries, leading to fatty degeneration and scar formation, which compromise muscle function and its structural integrity^1^. Severe skeletal muscle injuries are frequently encountered in orthopedics and traumatology^2^.

Novel therapeutic strategies that aim to enhance skeletal muscle regeneration involve the local delivery of biologics such as growth factors and cells^3^. Mesenchymal stromal cells (MSCs) are promising cell source for such applications due to their immunomodulatory, paracrine, and differentiation potential^4^. Their regenerative capability has already been validated in several animal models of muscular dystrophy, myocardial infarction, and skeletal muscle trauma. We previously reported a clinically relevant muscle injury model that consists of multiple severe crush trauma to the soleus muscles of Sprague Dawley rats. In this model, the untreated controls consistently exhibit a permanent loss in muscle function of approximately 50% compared to the uninjured contralateral tissue^5^. However, the injection of MSCs immediately after the trauma significantly improves the functional healing outcome in as early as 28 days^6,7^. Using placental-derived MSCs, we recently obtained similar promising results in a human phase I/II clinical study (phase I/II)^8^.

Although evidently successful, the mechanisms underlying the regenerative function of MSC are still unclear. Transplanted MSCs are mainly active within the early phase of muscle repair^9^. They seem to promote muscle regeneration not because of their differentiation into muscle cells or fusion with existing myofibers, but apparently via paracrine effects^5^. Successful healing is accomplished by the spatiotemporal interplay between various cell types and biological processes, which is mirrored by large changes in the protein repertoire of injured muscle^10^. Thus, the characterization of the muscle proteome after injury and how it is altered after cell therapy could elucidate mechanisms by which MScs induce muscle regeneration and may also enable the discovery of new therapeutic targets for directed interventions. This is challenging because such an endeavor requires the spatial assessment of local molecular alterations within the injured muscle.

Commonly used techniques to investigate tissue proteomes include liquid based proteomic approaches, e. g. 2D gel electrophoresis or liquid chromatography (LC) based mass spectrometry^11,12^. However, these techniques do not enable a direct correlation between differentially expressed protein profiles and the tissue histology. Since, previous investigations have suggested that the regenerative changes are spatially restricted and dependent on pathophysiological surroundings, liquid based approaches might be suboptimal to gain insights into MSC’s mode of action^13^. Matrix-assisted laser desorption/ionization imaging mass spectrometry (MALDI-IMS) enables the spatially resolved tissue assessment via specific molecular signatures (e.g. proteins, peptides, lipids, and molecules of cell metabolites) and allows their correlation with alterations in the tissue histology^14–18^. Recently, we showed that MALDI-IMS enables the discrimination and classification of pathophysiological muscle regions through the direct (*in-situ*) analysis of tissue sections^13^. Thereby, the primary traumatized muscle region can be distinguished from trauma adjacent tissue via the lower intensity distribution of the muscle proteins carbonic anhydrase III (Ca3) and skeletal muscle alpha actin (Acts).

In the current study, we used the MALDI-IMS workflow to determine spatial peptide alterations in response to MSC-TX. We use m/z values from Acts (as described earlier^13^) to discriminate between the primary traumatized (tm) and trauma adjacent (tam) muscle areas, allowing us to assign peptide distributions to these specific pathophysiological regions. Next, we used multivariate classification methods, such as segmentation mapping, principle component analysis (PCA) and probabilistic latent semantic analysis (PLSA), to identify differential peptide distributions between the tm and tam areas of injured skeletal muscles from the MSC-TX and control groups. This analysis revealed that MSC-TX predominantly affects the tam region, whereas its influence on the tm regions is minimal. Subsequently, we use LC mass spectrometry and immunohistochemistry to identify the proteins corresponding to uncover peptide intensity distributions and to validate our findings in the specific muscle regions.

## Results

### MALDI IMS

We analyzed muscle specimens at day seven after injury to investigate early effects of MSC-TX. Mass spectra of MSC-TX and control muscles were extracted and statistical data analysis was performed by SCiLS Lab software. In total, we extracted 534 aligned m/z values in the typical mass range for tryptic peptides between m/z 800 to 3.500 (Supplementary Table 1). Average spectra of tam and tm regions of MSC-TX and control muscle tissues are shown exemplarily in Supplementary Fig. 1.

### Determination of pathophysiological regions

**Figure 1:**
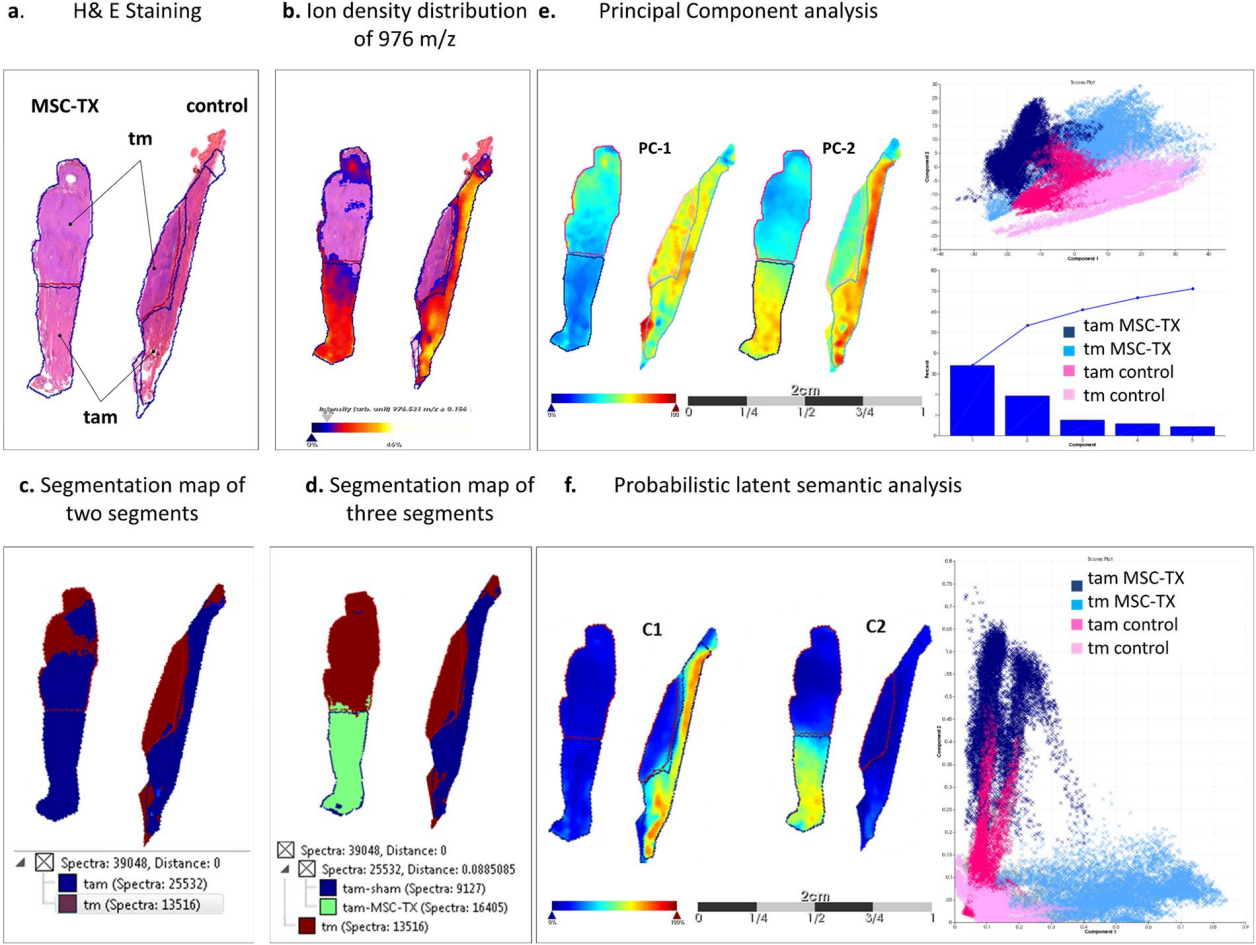
Discrimination pathophysiological region within injured skeletal muscles of MSC-TX and control group. **(a)** H&E staining of traumatized soleus muscles from the MSC-TX (left) and control group (right). Primary trauma region are indicated as tm and the trauma adjacent area as tam. (**b)** Discrimination of tam and tm region by alpha skeletal muscle actin (Acts). The m/z value 976 shows significantly lower intensities (p < 0.05) in the tm regions compared to the tam regions of both group. **(c)** Spatial segmentation analysis with two segments results in a clear discrimination of tam tissue area (blue region) from the tm (red). **(d)**. A third segment (green region) could be determined exclusively in the tam region of MSC-TX muscle tissue. **(e)** Intensity distribution of the first principal component PC1 for the considered tissue sections, which clearly discriminates MSC-TX muscles from corresponding regions of the control. Principal component PC2 clearly discriminates between tm and tam region in both groups. **(f)**. Probabilistic latent semantic analysis discriminates tam region of MSC-TX from the corresponding region of the control muscles. First component C1 shows a higher intensity in tam region of control muscles. In contrast, the second component C2 shows an increased intensity exclusive in the tam region of MSC-TX muscles.

According to our previous study^13^, we initially determined the pathophysiological regions, trauma adjacent (tam) and direct trauma (tm), within the muscles from both treatment groups by using the m/z values from 976 Da and 1198. We noted that the intensity distributions of m/z value 976 Da and 1198, significantly vary between the tam and tm region in muscles from the MSC-TX and control group (Table 1, Fig. 1b; AUC <0.35; p < 0.001; m/z correlation value > 0.65).The unsupervised data analysis of the peptide signatures by the segmentation mapping approach also results in two discriminative segments according to the m/z values in the soleus muscle of control and MSC-TX group (tam, blue, and tm region red, Fig. 1c).In total, determined regions includes 9864 (tam-MSC-TX), 9236 (tam-w/o MSC-TX), 10041 (tm-MSC-TX) and 6809 (tm-w/o MSC-TX) spectra.

**Table 1:** Discrimination between primary trauma and trauma adjacent region in muscle tissue with and w/o MSC-TX by m/z values of ACTS.

IMS m/z observed	IMS m/z Mr (exp)	LC-MS/MS Mr (exp)	Delta [Da]	Delta [ppm]	ROC [AUC] tm/tam	p-value
976.44	975.43	975.43	0.0037	3.79	0.086	<0.001
1198.63	1197.62	1197.07	0.0653	54.52	0.153	<0.001

### Determination of differences within the pathophysiological regions between MSC-TX and control muscle tissue by multivariate statistical data analysis strategies

The determination of differences between the tam or the tm region of MSC-TX treated and corresponding control muscles requires an unbiased analysis of spatial changes. The hierarchical expansion of the segmentation map, calculated by the bisecting k-means clustering method, results in a third segment exclusively describing the tam region of MSC-TX treated muscles (green, Fig. 1d). All biological replicates are shown in Supplementary Fig. 2.

Subsequently, we performed a Principal Component Analysis (PCA) to determine the largest difference in both pathophysiological regions between MSC-TX and control group (Fig. 1e) The principal component 1 (PC-1) mainly captured the differences within the tam region between MSC-TX treated and control muscles (PC-1 33.9%). PC-2 clearly discriminated tm and tam of the MSC-TX group from the corresponding regions of control muscles (PC-2 19.27%). All biological replicates are shown in Supplementary Fig. 3. Additionally, we performed a probabilistic latent semantic analysis (PLSA), which allowed the direct interpretation of score images and loadings. The spectra from the tam region of control muscles were characterized by high values for component 1, whereas the component 2 shows high values in the tam region of the MSC-TX muscles. Component 1 and 2 show low values in the tm region of both control and MSC-TX group (Fig. 1f). All biological replicates are shown in Supplementary Fig. 4. In summary, applying different unsupervised multivariate classification methods, we could discriminate between tam and tm region of the muscles and more importantly identified distinct differences in the tam region between the MSC-TX treated and control muscles. Consequently, even the individual comparison between the corresponding pathological regions of both treatment groups revealed that both approaches only very weakly discriminates between tm-CTRL vs tm-MSC-TX region, while they very well separate the tam-CTRL from the tam-MSC-TX area (Supplementary Fig. 5).

### Determination of characteristic m/z values of MSC-TX muscle tissue

**Figure 2:**
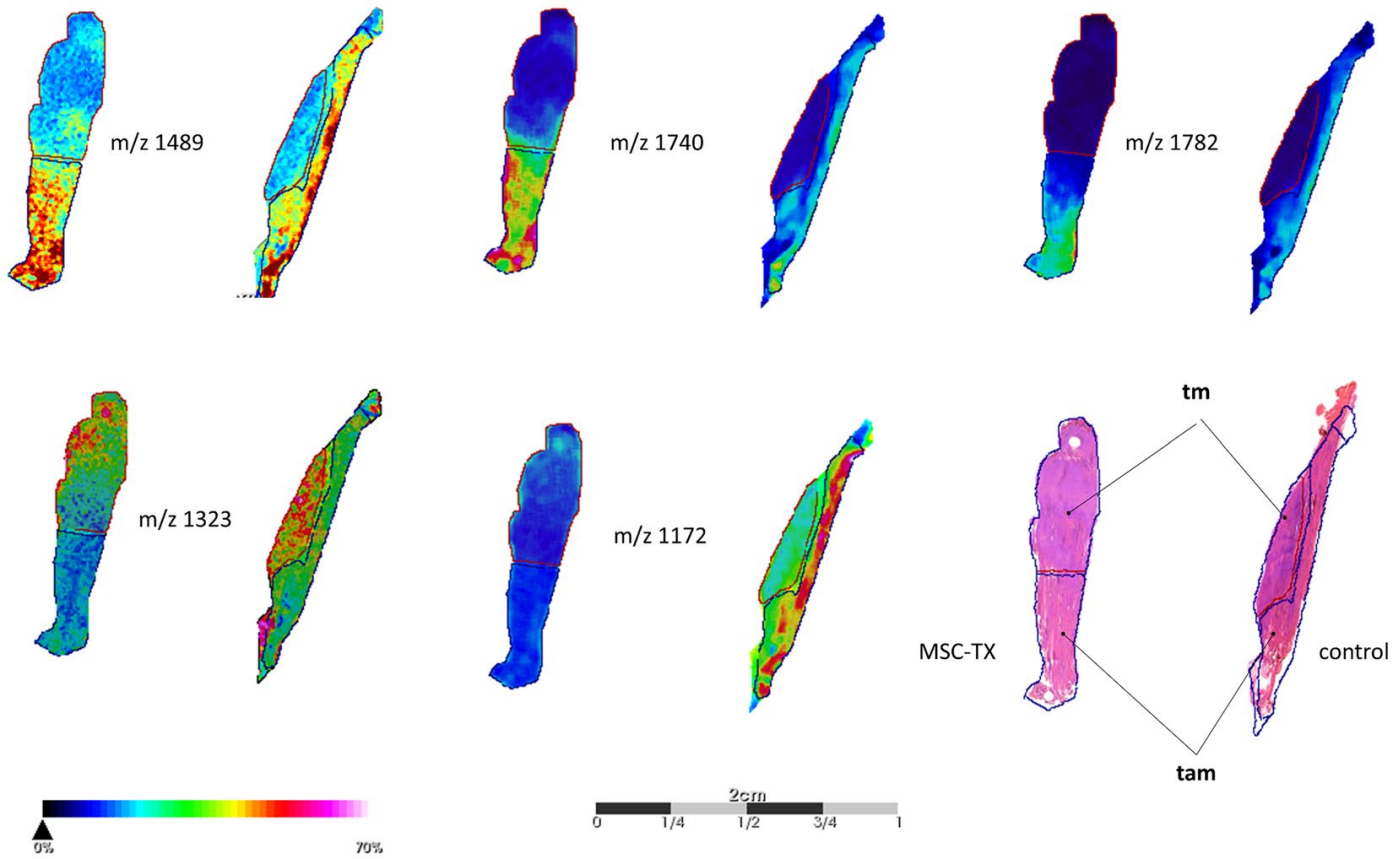
Spatial distribution of characteristic m/z values for the traumatized and trauma adjacent regions of MSC-TX and control muscle tissue. The m/z-values 1489, 1740 and 1782 are significant increased (p < 0.05) in tam region of MSC-TX treated muscles in comparison to the control samples. In contrast, intensity distribution of m/z-values 1323 is significantly increased (p < 0.05) in tm-region and m/z-values 1172 is increased in the tam-region of control muscles.

To determine characteristic m/z values between the unsupervised determined regions of both groups, we performed direct (region-specific) comparison of all 534 aligned m/z values using ROC analysis and hypothesis tests (Supplementary Table 1). A visualization of selected m/z-values correlating to a specific pathophysiological region is shown in Fig. 2. In total, we found 45 m/z-values that discriminate the tm region of MSC-TX treated muscles from the corresponding region of the control muscles (AUC <0.35 or >0.65; p < 0.01; peak intensity ratio >1.2 and <0.8, Supplementary Table 2). For instance, we found a decreased spatial ion distribution with m/z value 1323 Da in the tm region of MSC-TX group compared to the corresponding region of the control group. We identified 96 m/z values which distinguish the tam region of MSC-TX treated muscles from the corresponding region of control muscles (AUC <0.35 or >0.65; p < 0.01; peak intensity ratio >1.2 and <0.8; Supplementary Table 3). In particular, the spatial ion distributions of the peptide-values 1489 Da, 1740Da and 1782 Da are significantly higher in the tam region of MSC-TX group compared to the control group. Conversely, we identified a higher intensity of m/z value 1172 Da in the tam region of control muscles compared MSC-TX treated muscles. Since the number of spectra in the tm region apparently differs between both groups (MSC-TX: 10041 and control: 6809) and thereby compromise the discrimination potential of the method, we performed ROC analysis also with 6000 randomly selected spectra from the tm region of each group. The obtained results are nearly identical to the data obtained above and provide evidence for the robustness of the approach (Supplementary Table 5).

### Identification and validation of proteins affected by MSC-TX

To identify the proteins corresponding to the detected discriminative m/z values, adjacent tissue sections were analyzed with a “bottom up” LC-MS/MS approach (Supplementary Table 4). This analysis assigned 64 out of 141 m/z values (AUC <0.35 or >0.65; p < 0.01; peak intensity ratio >1.2 and <0.8) from the MALDI IMS experiment to 52 proteins (Supplementary Tables 2, 3). More than one identified m/z value of Col6a1 (Collagen alpha-1(VI) chain), Myl3 (myosin light chain 3), Hspa8 (Heat shock cognate 71 kDa) (hsp8) Tnc (tenascin-C), and Flnc (filamin C) was assigned to the observed m/z values from the MALDI-IMS experiment and assumed as correctly identified according to the IMS guidelines (Table 2)^19^.

**Table 2:** MALDI imaging derived identified proteins which different intensity distribution between muscle with and w/o MSC-TX.

IMS m/z observed	IMS m/z Mr (exp)	Ratio [intensity] tam MSC-TX/control	Ratio [intensity] tm MSC-TX/control	ROC(AUC) tam MSC-TX /control	ROC(AUC) tm MSC-TX /control	LC-MS/MS Mr (exp)	Delta [Da]	Delta [ppm]	Score	Sequence	Gen name	Description
1323.71	1322.70	0.63	0.70	0.178	0.252	1.322.70	0.003	2.2	40	DRLLPPTQNNR	Col6a	Collagen VI alpha 1
1701.98	1700.97	0.54	0.59	0.192	0.216	1.700.86	0.112	65.6	93	VAVVQYSGQGQQQPGR		
979.58	978.57	0.81	0.45	0.361	0.172	978.49	0.079	80.5	33	LMQVEFGR		
1328.64	1327.63	0.54	0.67	0.333	0.232	1.327.71	0.074	55.4	34	SGDDVRGPSVVLK		
1199.70	1198.69	1.74	2.3	0.773	0.857	1.198.67	0.026	22.0	47	DAGTIAGLNVLR	Hspa8	Heat shockcognate 71 kDa
1487.82	1486.81	2.4	1.28	0.815	0.672	1.486.69	0.116	77.8	63	TTPSYVAFTDTER		
1172.57	1171.57	0.44	0.73	0.097	0.251	1.171.64	0.077	65.4	35	GGPLSGPYRLR	Ca3	Carbonic anhydrase 3
1390.75	1389.74	0.51	0.79	0.111	0.310	1.389.72	0.023	16.7	40	SMLRGGPLSGPYR		
1396.76	1395.76	1.56	0.819	0.708	0.335	1.395.75	0.011	7.5	73	ALGQNPTQAEVLR	Myl3	Myosin light chain 3
1782.99	1781.98	1.37	1.1	0.688	0.538	1.781.94	0.040	22.6	101	AAPAPAAAPAAAPEPERPK		
937.56	936.55	2.0	1.42	0.712	0.595	936.50	0.048	51.4	42	LGSFGSITR	Flnc	Filamin C
1489.90	1488.89	1.56	1.28	0.728	0.620	1.488.77	0.116	78.2	31	GVAGVPAEFSIWTR		
1668.81	1667.80	4.14	1.44	0.977	0.696	1.667.81	0.013	60.4	32	YAPISGGDHAEIDVPK	Tnc	Tenascin-C
1.740.95	1.739.94	2.0	1.61	0.852	0.738	1.739.83	0.105	60.4	32	EGDPATINAATEIDAPR		

The m/z values of Col6a1 showed a decrease in the density distribution in the tm as well as tam region of MSC-TX compared to the control muscle. Ion intensity of m/z values corresponding to Ca3, are significantly higher (AUC <0.35; p < 0.01; peak intensity ratio >1.2 and <0.8) in tam region of control compared to the tam region of MSC-TX muscle tissue, but only minor differences between the tm region of both groups. Furthermore, we found that ion intensity of m/z values of Hspa8 significantly increased in the tam as well as in tm region of MSC-TX treated muscles compared to the control (AUC <0.35; p < 0.01; peak intensity ratio >1.2 and <0.8). M/z values of TNC, FlnC and Myl3 were significantly higher in tam region of MSC-TX in comparison to tam-control muscle tissue.

**Figure 3:**
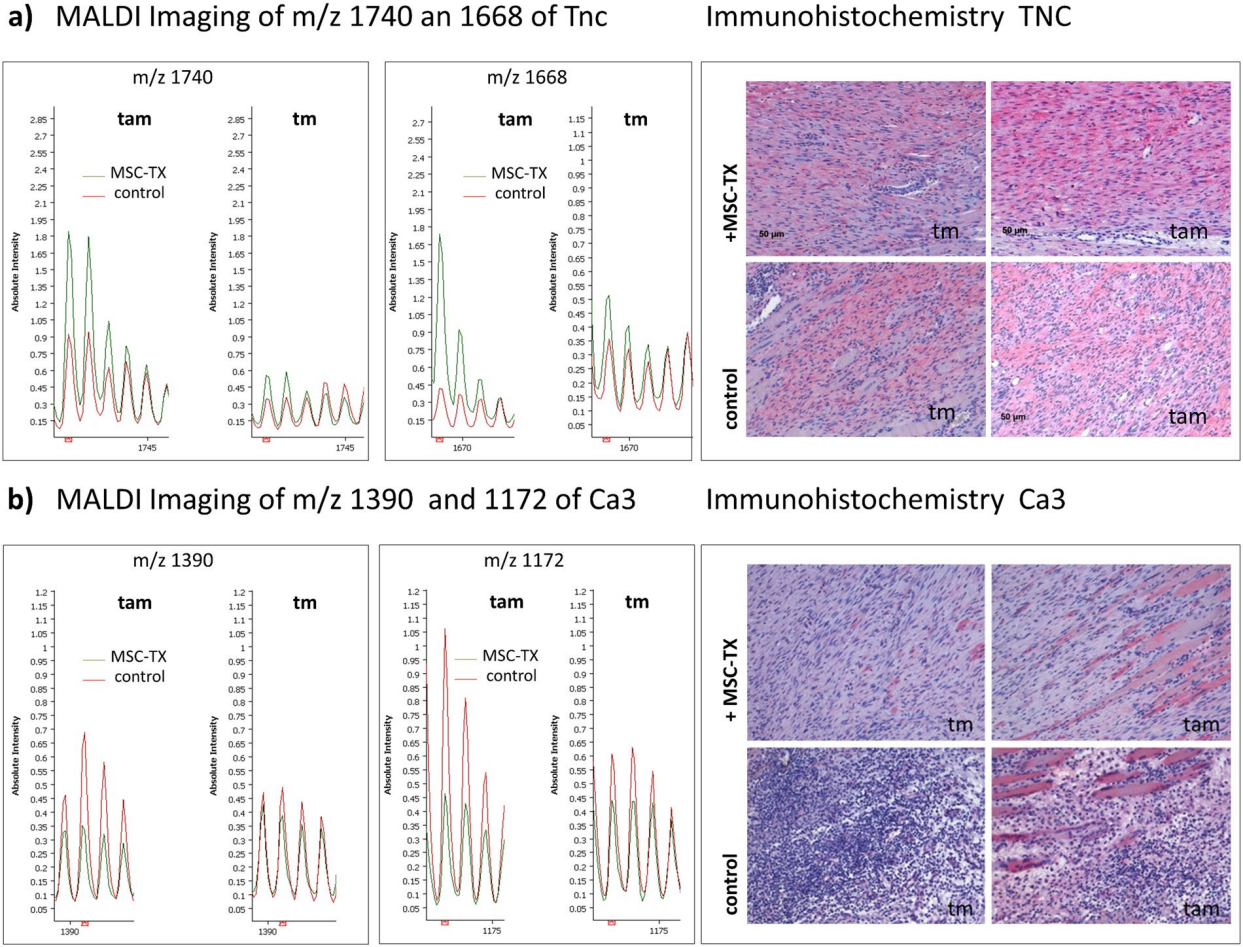
Immunohistochemical validation of tenascin-C and carbonic anhydrase 3. Shown are MALDI IMS spectra displaying the identified average m/z species of **(a)** tenascin-C (TnC) and **(b)** carbonic anhydrase 3 (Ca3) within the tam and tm region of MSC-TX and control muscles, respectively. Immunohistochemical staining revealed a stronger expression of TnC (above right subplot) in tam region of MSC-TX muscle, whereas the expression of Ca3 is elevated in the tam region of control muscles.

The assignments based on LC-MS/MS-identified peptide lists have to be verified by immunohistological staining (IHC) of the respective tissue^20^. Exemplarily, tenascin-C and carbonic anhydrase III were validated by immunohistochemistry. We observed a clear positive staining of tenacin-C in the tam region of the MSC-TX group compared to the same region of the control group. Similarly, IHC staining against carbonic anhydrase III revealed a positive staining in the tam region of control muscles compared to the corresponding region of MSC-TX treated muscles (Fig. 3). These results confirm the observations based on IMS experiments, which determine increased intensity distributions of peptide values from carbonic anhydrase III in the tam region of control muscle tissue and from tenacin-C in the tam region of MSC-TX muscle tissue (Fig. 3). As an example, the ion distributions of m/z values 1740Da, 1168 Da (Tnc) and m/z values 1390 Da, 1172 Da (Ca3) for all biological replicates are shown in Supplementary Fig. 6.

## Discussion

An ever-increasing number of reports suggest that the transplantation of MSCs from different sources improves the regeneration in a wide range of disease related to muscle tissue, including muscular dystrophy, cardiomyopathy, and severely injured skeletal muscles^7,21–23^. In a clinically relevant injury model, we recently showed that MSC-TX improves the functional healing outcome (28 and 56 days after injury) by remodeling scar tissue and promoting the formation of new myofibers^5^. However, the underlying mechanism by which MSCs promote muscle repair remains largely unknown. Recent studies provide evidence that MSC administration improves muscle regeneration, not because of differentiation into muscle fibers, but apparently due to their paracrine and immunomodulatory effects mediated by the secretion of various cytokines and other growth factors^24,25^. Using doxorubicin-induced cardiomyopathy mice model, Zhang *et al*. provided elegant insight into the paracrine effects by which MSCs improve the structural and functional healing outcome, and highlighted the critical role of Rap1-mediated NF-κB signaling and the secretion of MIF and GDF-15 in this process^26,27^. It is important to note that the regenerative properties of MSCs are very highly dependent on their microenvironment^25,28^. For example, Ren *et al*. showed that MSCs have no constitutive immunomodulatory function, but become potently immunosuppressive in conjunction with a pro-inflammatory (injury) environment^29^. It has also been shown that the paracrine effects of MSCs depend on cell-cell interactions and especially structural characteristics of their surrounding matrix^30^. Apart from an incomplete understanding of the precise mechanism by which MSCs promote tissue restoration, there is a paucity of data about early molecular changes within the injured muscle in response to the MSC-based intervention. Since previous studies provide the evidence that MSCs survive at most only the first seven days after their injection into the muscle, their direct regenerative effect seems to be limited to the initial phase of the healing process^9^. Surprisingly, we found no (immune) histological difference between treated and untreated muscles seven days after injury in an earlier investigation^5^. This may be in part due to the limitation of these standard techniques to describe very early structural and molecular alterations within the muscle.

MADLI IMS analysis allowed us to determine *in situ* the spatial intensity distribution of peptide and proteins signatures in skeletal muscle tissue. This methodology provides several advantages in contrast to other supervised imaging methods like IHC or *in situ* hybridization, such as the high specificity of MS detection, picayune sample preparation, wide range of analytes and multiplex capability. Thus, IMS facilitates the acquisition of molecular signatures while preserving their location in the tissue. As disadvantage is that, the technique only allows the determination of abundant proteins and peptides due of the absence of a peptide separation procedure. However, thousands of spectra were acquire in an automatic manner and evaluated by unsupervised multivariate statistical methods. This enables the determination of novel region-specific alterations in response to MSC-TX, which have so far remained hidden from conventional histological methods.

In this work, we analyzed six different muscle tissue sections with the objective of determining local peptide signatures (sub-regions) within the pathophysiological areas, which differ between the muscles of MSC-TX treated and untreated control animals. Since the traditional H&E staining only approximately distinguishes these regions, we used previously published m/z values of alpha skeletal muscle actin to determine primary trauma (tm) and trauma adjacent (tam) regions in injured skeletal muscle tissue^13^. The discrimination of these pathophysiological regions was ensured by an unbiased segmentation approach based on bisecting k-means clustering. This approach results in two clusters, distinguishing this region similar to the Acts m/z values.

The analysis of data sets obtained by IMS experiments represents a challenge when different treatments, replicates and development phases are to be compared and molecular features to be extracted. Segmentation mapping, PCA and PLSA were used in this study as multivariate statistical models for the analysis of a diverse set of MALDI IMS data. The algorithms are designed to categorize the data set according to mass spectral characteristics. The classification of pathophysiological regions by characteristic peptide makers is based on the identification of common signal patterns represented in particular areas (tissues) of the sample sections. The k-means clustering is thought to be well suited to get a first impression about the diversity within the sample set. This information can be used to further apply the PLSA algorithm, for which a prior knowledge about the diversity of the sample material is recommended. The results obtained by PLSA are more specific in displaying molecular features of the tissues than those observed by segmentation mapping based on k-means clustering method.

Consistently, grouping spectra according to spectral similarities (segmentation approach), determination of the largest variance compound (PCA) and calculation of spatial tissue components (PLSA), suggest that MSC-TX predominantly leads to molecular alteration in the tam region of skeletal muscle. The fact that distinct evaluation strategies using different algorithms reveal similar results underscores the robustness of the observation.

These results show that the MALDI-IMS technique in combination with unsupervised data evaluation strategies allows a promising unbiased classification of different regions in injured skeletal muscle. It enables the determination of molecular changes after MSC-TX, which cannot be observed by conventional supervised techniques such as IHC.

Following this evaluation, differential m/z values were identified between pathophysiological regions (tam and tm) of control and MSC-TX groups. A large number of isobaric ions^31^ and the presence of so-called chimera spectra^32^ adversely affected the identification of m/z values by MS/MS on tissues. Therefore, we used a corresponding “bottom-up” LC-MS/MS analysis, which enabled the identification of the obtained MALDI-IMS m/z value^33^. The intensity distribution of these m/z values changes depends on pathophysiological environments of MSC-TX in comparison to the control muscle tissue. Since ion suppression, efficient extraction and sample complexity negatively influence the ionization of peptides, the combination of MALDI-IMS and LC−MS/MS requires more than one peptide to correctly identify the corresponding proteins^34^.

In particular, Ca3, Myl3, TnC, Flnc, Col6a and Hspa8 were reliably identified and indicate the differences in the tam and tm regions between MSC-TX and control muscle tissues. Other differentially expressed m/z species (Supplementary Tables 1–3) remain unidentified so far or were identified with less than two peptides and require further investigation.

As mentioned above, although the benefits of MSC-TX to form and function of the injured muscles are acknowledged, little is still known about mechanistic effects of MSC-TX on the pathophysiological environments in injured skeletal muscle. The identification of differentially regulated proteins allow new insights into the molecular alterations in the muscle tissue in response to MSC-TX. For example, the high intensity distribution of TnC and low intensity distribution of Ca3 m/z species in trauma adjacent regions of injured skeletal muscle after MSC-TX was previously unknown, and could be verified by immunohistological staining. Tenascin-C is present during the wound healing, morphogenesis, and as a component of the stem cell niche^35,36^. It is also expressed after mechanical stress in actively remodeling musculoskeletal tissue and protects naturally occurring MSC from FasL-induced apoptosis^37,38^. Furthermore, TnC is described as an autonomously secreted factor, which supports fetal muscle stem cells regenerative capacity^39^. Thus, results of the present study suggest, that the process of remodeling and morphogenesis is more advanced in the MSC-TX treated muscles in comparison to the control muscles.

This suggestion is in line with a decrease in the intensity distribution of Ca3 in the tam region of MSC-TX muscle tissue. Ca3 is a muscle-specific, cytosolic protein of slow twitch oxidative fibers (type I muscle fibers) and catalyzes the reversible hydration of carbon dioxide and thus is involved in buffer systems and ion fluxes in cells^40^. The native soleus has predominantly slow muscle fiber muscle phenotype muscle, and our previous investigations suggest that successful regeneration is depending on the fast restoration original muscle phenotype^3,5^.

Additionally, we identified Collagen VI alpha 1 (Col6a), Heat shock cognate 71 kDa (Hspa8), and Filamin C (FlnC) as potential biological factors involved in therapeutic muscle response. Col6a shows a decrease in intensity in the tm region of the MSC-TX in comparison to the control injured skeletal muscle. This protein is an extracellular matrix protein and regulates satellite cell self-renewal and muscle regeneration. A lack of Col6a1 results in a reduced self-renewal capability after injury^41^. Peptide intensity distribution of Hspa8 were higher in the primary trauma and the trauma adjacent region of the injured skeletal muscle treated with MSC-TX. The expression of heat shock protein members highly depends on the cell microenvironment and these proteins are involved in the survival, prevention of apoptosis and the defense against extracellular induced stresses of different (mesenchymal) cell types^28^. The corresponding peptide values of FlnC were higher in the tam region of MSC-TX treated muscles and this protein interacts with actin and various proteins in the sarcoglycan complex. It is involved in actin polymerization, mechanoprotection, signal transduction and cellular migration, as well as the regulation of the contractile apparatus^42–45^. Furthermore, filamin C participates in the signal transduction of cellular migration of the myogenic progenitor cell population, which is mandatory for muscle development^46^. Myl3 is an alkali slow Myosin light chain isoform (Mlc1sb) which is expressed in slow muscle and plays a role in molecular motor of muscle contraction^47^. Therefore, the increase of m/z values in tam region of MSC-TX muscle may indicate an advanced regeneration process in this region compared to the control group. Presently, these potential candidates have not been evaluated by IHC and require further elucidation efforts.

In summary, we show that MALDI-IMS is a suitable technique to identify proteomic signatures and allows distinguishing changes of molecular architecture in pathophysiological regions (trauma and trauma adjacent) due to MSC-TX in injured muscle tissue. Furthermore, the results of complementary unsupervised multivariate classification methods (PLSA, PCA, segmentation map) suggest that MSC-TX induces greater changes in the trauma adjacent tissue region. The uniqueness of our approach is the representation and quantification of the entire skeletal muscle and this beyond the primary traumatized region. To the best of our knowledge, there are no comparable studies, which include the trauma-adjacent regions and investigate early molecular alterations in response to a cell-based intervention. Most other studies primarily focusing on the actual injury region at very late time points or on the transplanted cells themselves. Our research shows that the very early molecular alterations occur largely in the region adjacent to the trauma and only to a small extent in the actual trauma region. These data contribute to our current understanding of the early stages of muscle healing and support the identification of new targets for direct (e.g. pharmacological) intervention.

## Materials and Methods

### Study design

Our main objective was to assess local changes of peptide distribution within injured skeletal muscles in response to the MSC-TX treatment by using the MALDI-IMS technique. Muscle sections were obtained from an unrelated study using our established clinically relevant injury model that consists of multiple severe crush trauma in the soleus muscle of female Sprague Dawley rats. The animal experiments were approved by local legal representative (Landesamt für Gesundheit und Soziales, Berlin: Registration number: G0119/12). In this model, animals exhibited a permanent loss of muscle force approximately 40–50%, which was significantly improved by the transplantation of autologous MSCs (Supplementary Fig. 7). To investigate early molecular changes in response to the intervention, we used tissue samples obtained seven days after trauma from n = 3 with MSC-TX and n = 3 corresponding controls. The muscle sections were analyzed by MALDI-IMS as described below. In order to match the obtained m/z values with the corresponding proteins, we used a “bottom-up”-nano LC - MS/MS approach on the adjacent tissue sections from control groups. Identified proteins were subsequently validated by immunohistochemical staining of whole muscle sections.

### Animal experiments

Biological samples were obtained during a previous study (Registration number: G0119/12) and carried out in compliance with the policies and principles established by the Animal Welfare Act, the NIH Guide for Care and Use of Laboratory Animals, and the national animal welfare guidelines. Briefly, female Sprague Dawley rat were used (4 month old, weighing 200–250 g, Charles River, Sulzbach, Germany). Bone marrow aspiration, MSC isolation, MSC phenotype and differentiation, and animal surgeries were performed as previously described^5,48^. Bone marrow aspiration was conducted on both tibiae of all animals and MSCs were isolated by the plastic adherence method and cultured under normal conditions. Three weeks later, a standardized crush injury was performed on the left soleus muscle (unilateral surgery) as described earlier^6^. For this purpose, the soleus muscle was mobilized and crushed bluntly through a 3 cm posterolateral longitudinal incision of the skin and underlying fascia from the lateral gastrocnemius head to the achilles tendon. A curved artery clamp with tips surrounded by polyethylene tubes was used to avoid lesions of the muscle fascia. The muscle was manually clamped for 20 seconds. Standardized pressure on the muscle was ensured by closing the forceps to its third stage, resulting in a closing pressure of 112 ± 5.1 N^48^. After thorough rinsing with saline solution, the superficial muscles and the skin were sutured. One hour after trauma, 2.5 × 106 MSCs were washed and suspended in 50 µl of a 0.9% saline solution and locally injected into the soleus muscle using a 25-gauge cannula. The control group received an injection of 0.9% saline solution in the same manner. At day 7 after injury, animals were sacrificed and the muscles were harvested for subsequent analysis.

### Tissue Preparation and MALDI-IMS data acquisition

Tissue preparation and MALDI-IMS data acquisition were performed as previously described^13^. Briefly, paraformaldehyde (PFA)-fixed specimens were dehydrated by washing sequentially with increasing concentrations of ethanol, subsequently cleared in xylene, and embedded in paraffin. Formalin-fixed, paraffin-embedded sections (FFPE,10 µm) were prepared from paraffin blocks on a microtome (Leica RM 2125). In total six tissue sections were analyzed by MALDI Imaging (three MSC-TX and three control animals). Two muscle tissue sections, one from MSC-TX and one from control group, respectively, were transferred on the same Indium-Tin-Oxide slides (Bruker Daltonik, Bremen, Germany). Sections were dewaxed and passed through decreasing concentrations of ethanol according to an adapted protocol from Casadonte R *et al*.ii^49^. Using an automated spraying device (ImagePrep; Bruker Daltonik, nine spray cycle), 200 µl trypsin solution (20 µg, 20 mM ammonium bicarbonate/acetonitrile 9:1) was applied onto the section. After tissue incubation (3 h at 37 °C; moist chamber), matrix solution (1 ml [7 g/L] a-cyano-4-hydroxycinnamic acid in 50% acetonitrile and 1% trifluoroacetic acid) was applied using ImagePrep (15% power ± 40% modulation, 60 spray cycle). MALDI-IMS data acquisition was performed in positive ion reflector mode on an Autoflex III MALDI-TOF/TOF using flexControl 3.0 and flexImaging 3.0 software (Bruker Daltonik). Measurement settings were as follows: detection range of m/z 800–3500, 200 laser shots per spot, sampling rate of 0.5 GS/s, and raster width = 80 µm. External calibration was performed using a peptide calibration standard (Bruker Daltonik), which was separately spotted close to the tissue section area. Spectra processing was performed in flexAnalysis 3.0 (Bruker Daltonik) with spectra smoothing and baseline subtraction. After MALDI-IMS experiments, the matrix was removed with 70% ethanol and the tissue sections were stained with hematoxylin and eosin (HE) as histological overview staining^49^.

### Statistical data analysis

Statistical data analysis was perform using SCiLS Lab software (Version2015b, SCiLS GmbH, Bremen, Germany). MALDI-IMS raw data was imported into the SCiLS Lab software and converted to the SCiLS Lab file format. Simultaneous preprocessing of all data sets was perform to ensure better comparability between the sample sets. Imported data were pre-processed by convolution baseline removal (width: 20) and total ion count (TIC) normalization. Peak finding and alignment were created using a standard pipeline with the following settings: ± 0.156 Da interval width, mean interval processing, medium smoothing strength^50–52^. The primary trauma (tm) and the trauma adjacent tissue (tam) area of both groups were defined as regions of interest (ROI) and annotated by using the m/z value 976 Da, a previously published discrimination marker (identified as alpha skeletal muscle actin [Acts])^13^. To confirm the annotation another m/z value for Acts (m/z 1198) was used (AUC <0.35; p < 0.001; m/z correlation value >0.65).

In order to assess the spectral diversity among the sample sets, different multivariate clustering algorithms were applied. Based on the peptide signatures, data mining was perform by using a standard segmentation pipeline as published previously^13,50,51^. Peaks were selected using the Orthogonal Matching Pursuit (OMP) algorithm^52^ and top down segmentation were performed by bisecting k-means clustering, which performs a top-to-bottom splitting of mass spectra according to their similarities. Common spatial identities of molecular patterns in the sample were subsequently visualized by colour coding the resulting segmentation map.

To define common molecular features among the sample sets different unsupervised multivariate classification methods for mass spectra representing the tm and tam area of MSC-TX and control tissue were applied: Probabilistic Latent Semantic Analysis (PLSA) and principal component analysis (PCA) were performed as previously described^53,54^.

Theoretically, four principal components can be assumed: (i) tm of MSC-TX group, (ii) tm of control group, (iii) tam of MSC-TX group, and (iv) tam of control group. To ensure that no further sub-structures (matrix cluster and noise spectra) were included into the ROI, PLSA and PCA were performed with five components and the following settings: (i) Interval width of ± 0.156 Da, and (iii) individual spectra. In particular, PLSA was performed with deterministic initialization and PCA with unit variant scaling.

Receiver operating characteristic analysis (ROC) was used to assess the quality of all m/z values within specific ROIs to discriminate between MSC-TX and control muscles. For this method, the number of spectra in the ROIs of both groups should be approximately the same. If that was not the case, 6000 randomly select spectra per ROI/group were used. To determine statistical significance, discriminating m/z values (peaks) with a AUC <0.35 or >0.65 were subsequently analyzed using Wilcoxon rank sum test. M/Z values with a delta peak intensity of >0.3 and P < 0.001 were assumed as potential markers.

### Identification of m/z values by “bottom-up”-nUPLC mass spectrometry

To identify m/z values, complementary protein identification was performed on adjacent muscle sections by a “bottom-up”-nano LC - MS/MS approach as published previously^13^. Briefly, tissue digestion (20 µg trypsin, 20 mM ammonium bicarbonate /acetonitrile 9:1) was performed via ImagePrep. Peptides for nUPLC -MS/MS analysis were extracted directly from adjacent tissue sections into 40 µl of 0.1% triflouroaceticacid (TFA; 15 min incubation at room temperature). Peptides were separated 2–60% acetonitrile/in 0.1% formic acid) using an analytical UPLC System (Waters, PST C18 nanoACQUITY column; flow rate 400 nl/min, 70 min) and analyzed via Orbitrap XL mass spectrometer (Thermo). All raw spectra from the MS/MS measurement were converted to mascot generic files (.mgf) using the ProteoWizard software^55^. Mass spectra were analyzed using Mascot search engine (version 2.4, MatrixScience; UK) searching the UniProt database. Search was performed with the follwing set of parameters:: (i) taxonomy: Rattus norvegicus, (ii) proteolytic enzyme: trypsin, (iii) peptide tolerance: 10ppm, (iv) maximum of accepted missed cleavages: 1, (v) peptide charge: 2+, 3+, 4+; (vi) variable modification: oxidation (M); (vii) MS/MS tolerance: 0.8 Da, and (viii) MOWSE score >25. The results were exported as.csv files from Mascot. Identification of MALDI-IMS m/z values by using an LC-MS/MS reference list requires the accordance of more than one peptide (mass differences <0.9 Da) to subsequently correctly assign the corresponding protein^34^. Peptides with lowest mass difference to the LC-MS/MS reference list value were assumed as a match.

### Data availability

The datasets generated during and/or analyzed during the current study are available from the corresponding author on reasonable request.

## Electronic supplementary material

Supplementary Information
