# Supplementary material for: Unraveling local tissue changes within severely injured skeletal muscles in response to MSC-based intervention using MALDI Imaging mass spectrometry
Source: Sci Rep. 2018 Aug 23;8:12677. doi: 10.1038/s41598-018-30990-w (PMC6107672; doi:10.1038/s41598-018-30990-w)
Supplement: Supplementary file 1 — Supplementary Information [file 41598_2018_30990_MOESM1_ESM.pdf]

Supplementary Data & Materials

Unraveling local tissue changes within severely injured skeletal muscles in response to MSC-based intervention using MALDI Imaging mass spectrometry

Oliver Klein[1], Kristin Strohschein[1,2], Grit Nebrich[2], Michael Fuchs[2], Herbert Thiele[4], Patrick Giavalisco[3], Georg N. Duda[1,2], Tobias Winkler[1,2], Jan Hendrik Kobarg[5], Dennis Trede[5], Sven Geissler[1,2]

[1]Berlin-Brandenburg Center for Regenerative Therapies, Charité – Universitätsmedizin Berlin, Augustenburger Platz 1, 13353 Berlin, Germany

[2]Julius Wolff Institute & Center for Musculoskeletal Surgery, Charité – Universitätsmedizin Berlin, Augustenburger Platz 1, 13353 Berlin, Germany

[3] Experimental Systems Biology Max-Planck-Institute of Molecular Plant Physiology, Am Mühlenberg 14476 Golm, Germany

[4] Fraunhofer - Inst. Medical Image Computing MEVIS, Maria-Goeppert-Straße 3, 23562 Lübeck, Germany

[5] SCiLS, Zweigniederlassung Bremen der Bruker Daltonik, Fahrenheitstr. 1, 28359 Bremen, Germany

Correspondence:

Dr. Oliver Klein

E-mail: Oliver.klein@charite.de,

Fax: +49-30-450566904,

Telephone: +49-30-450566143

Supplementary material & methods

Chemicals

α-cyano-4-hydroxycinnamic acid (HCCA) was obtained from Bruker Daltonik (Bremen, Germany), ammonium bicarbonate, acetonitril (ACN), 0.1% trifluoroacetic acid from Fluka (St. Louis, USA) and trypsin from Promega (Madison, WI, USA). 100% trifluoroacetic acid (TFA) was purchased from Merck (spectroscopy purity, Darmstadt, Germany) and ammonium hydrogen phosphate from Sigma (Sigma St. Louis, USA).

Immunohistochemistry

Immunohistochemical staining of carbonic anhydrase III and tenascin-C was performed as described earlier.[12] Briefly, 3µm tissue sections were dewaxed and rehydrated. Antigen retrieval was done with 20mmol citrate buffer (pH 6) in a water bath for 20min. Rabbit anti-rat carbonic anhydrase III and rabbit anti-rat tenascin-C antibody (Abcam, Cambridge, UK) was used at 1:2000 dilution and 1:200 dilution, respectively. Visualization was done using biotinylated goat anti-rabbit IgG antibody (VectorLabs, Peterborough, UK) as secondary antibody and the Vectastain ABC-AP-Kit (Vector Labs) according to manufactures instructions. Specimens were counterstained with Hematoxylin. Images of whole muscle sections were collected using a Leica DMRB light microscope (Leica, Wetzlar, Germany) and digital camera (AxioCam MRc, Carl Zeiss, Göttingen, Germany) and merged with the AxioVision program (Rel. 4.4, Carl Zeiss).

Supplementary Figures

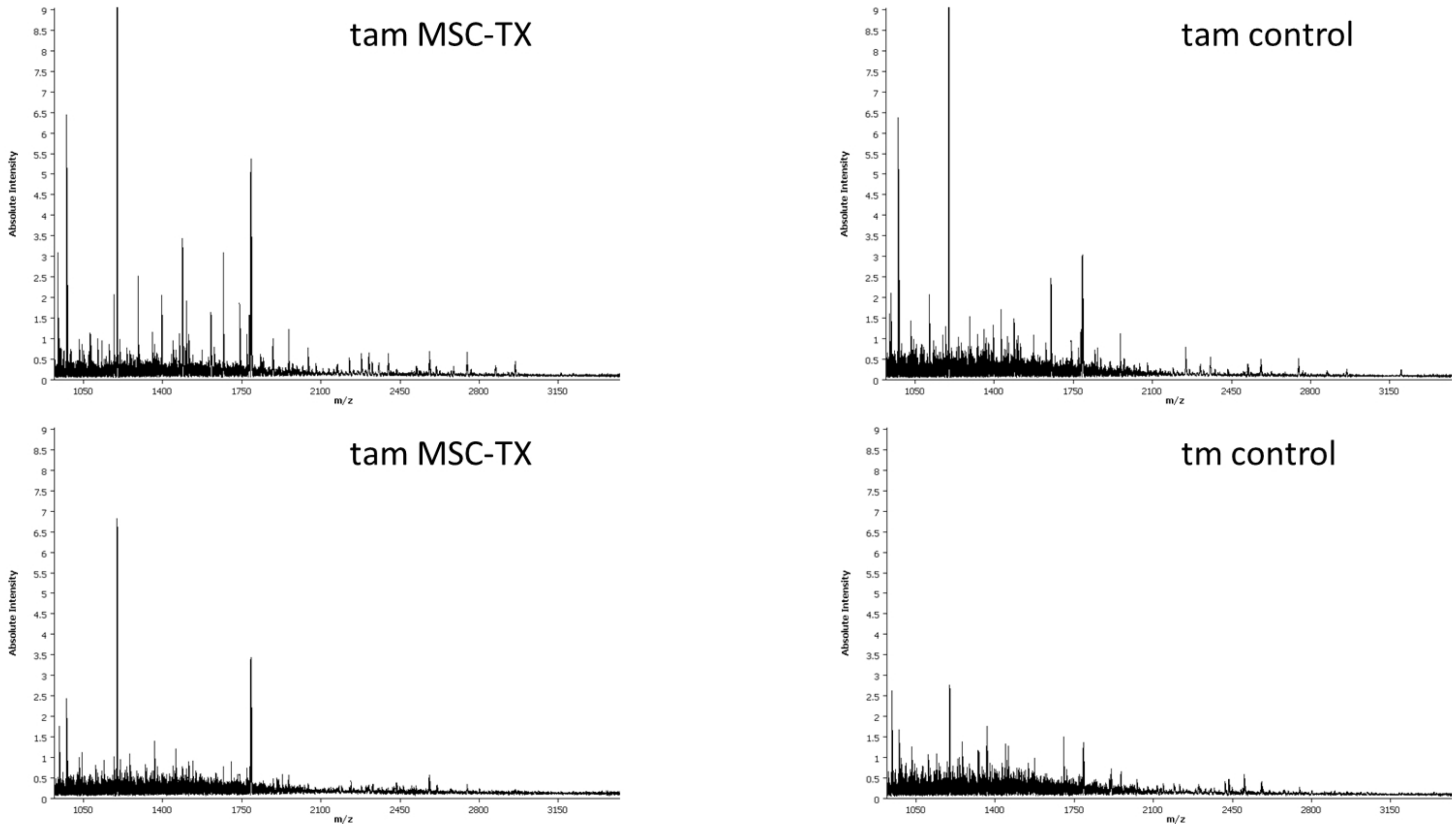

Supplementary figure 1 Representative average spectra of tam and tm regions of MSC-TX and control muscle tissues, respectively.

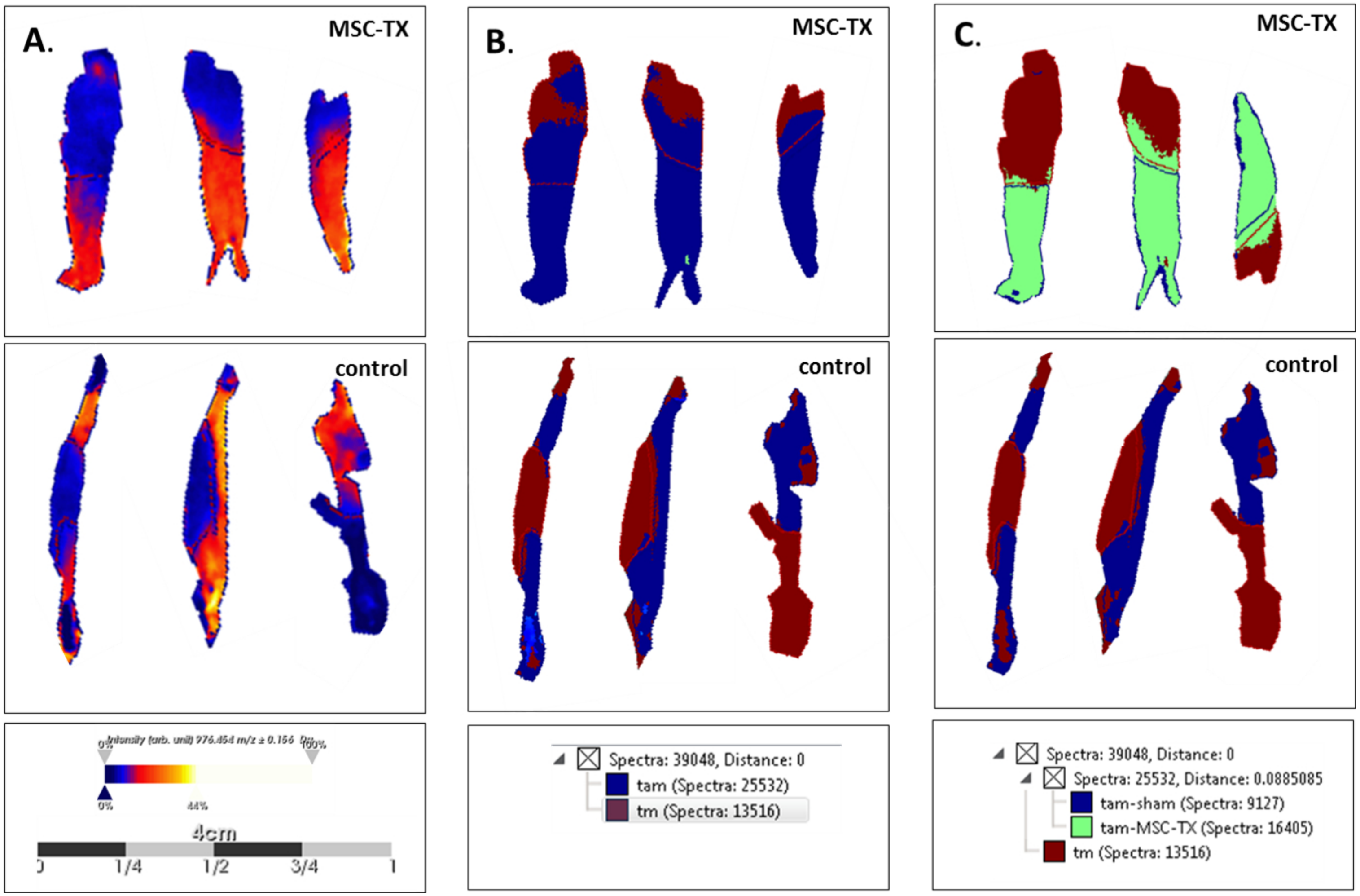

Supplementary figure 2: Discrimination of pathophysiological regions in muscle with and control for all biological replicates A) *Ion density distribution of m/z value 976 (ACTS) clearly discriminate the primary trauma (low intensity) and trauma adjacent region (high intensity) in muscle with and control.* ***B)*** *Two cluster results in a clear discrimination of trauma adjacent (tam) tissue area (blue region) and primary trauma (tm, red) region of all biological replicate.* ***C)*** *Three cluster results in a further clear discrimination between the trauma adjacent area of MSC-TX and control control muscle.*

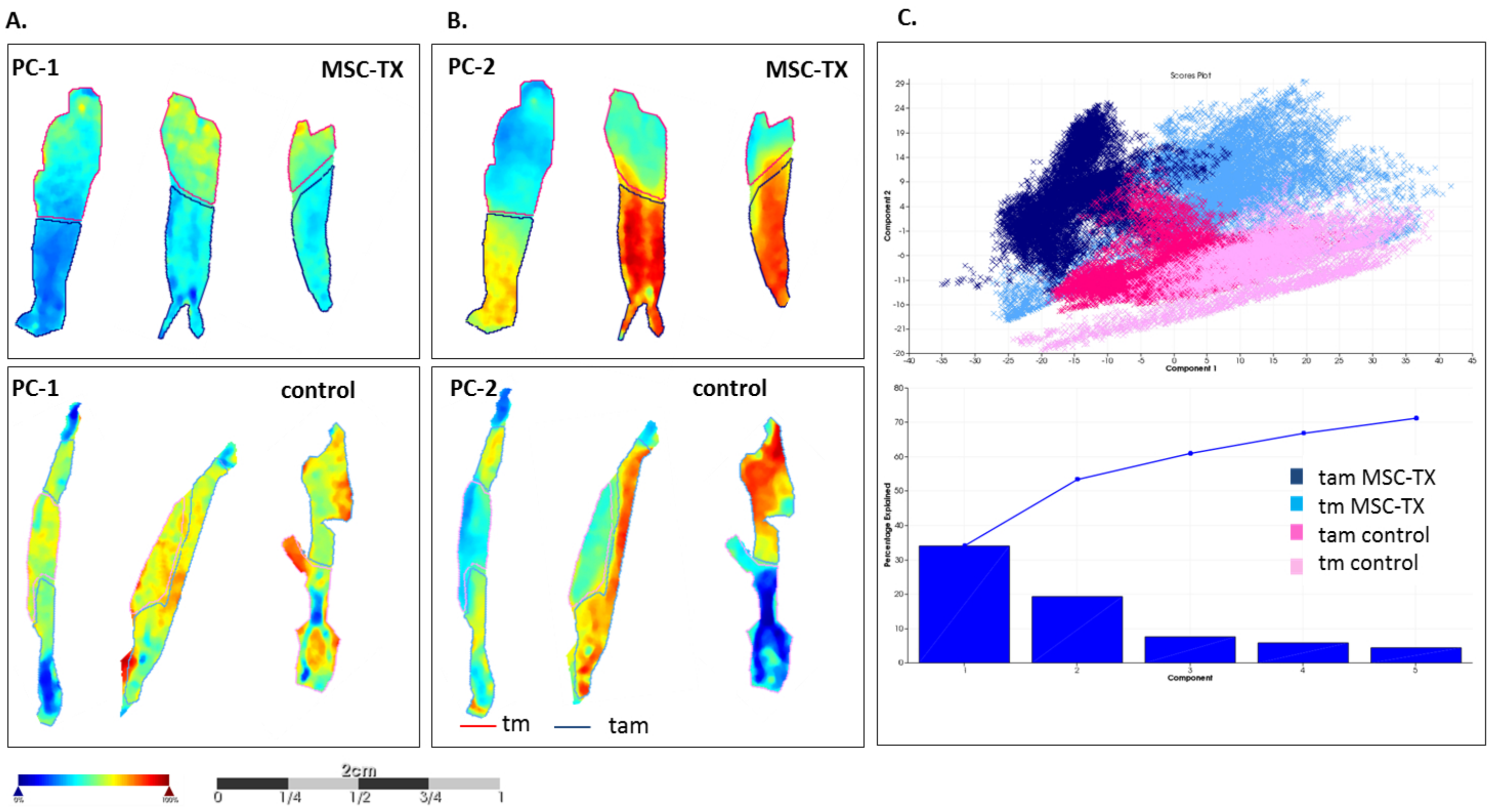

Supplementary figure 3: Principal component analysis (PCA) of mass spectra from traumatized (tm), trauma adjacent (tam) muscle region with and control for all biological replicates. A) *Intensity distribution of the first principal component PC1 for the considered tissue sections which clearly distinguish the trauma adjacent region-MSC-TX, low intensity of PC-1 ,to trauma region MSC-TX, trauma region and trauma adjacent muscle region control, high intensity of PC-1.* ***B)*** *Intensity distribution of the first principal component PC1 for the considered tissue sections which clearly distinguish the trauma adjacent region-MSC-TX, low intensity of PC-1 to trauma region MSC-TX, trauma region and trauma muscle region control, high intensity of PC-1.* ***C)*** *Score Plot of the spectra in the first 2 principal components in PCA, tm-MSC-TX (violet), tam-MSC-TX (dark blue) , tm- control (pink) and tam- control (light blue) Bottom row: variance explanation by first 5principal components.*

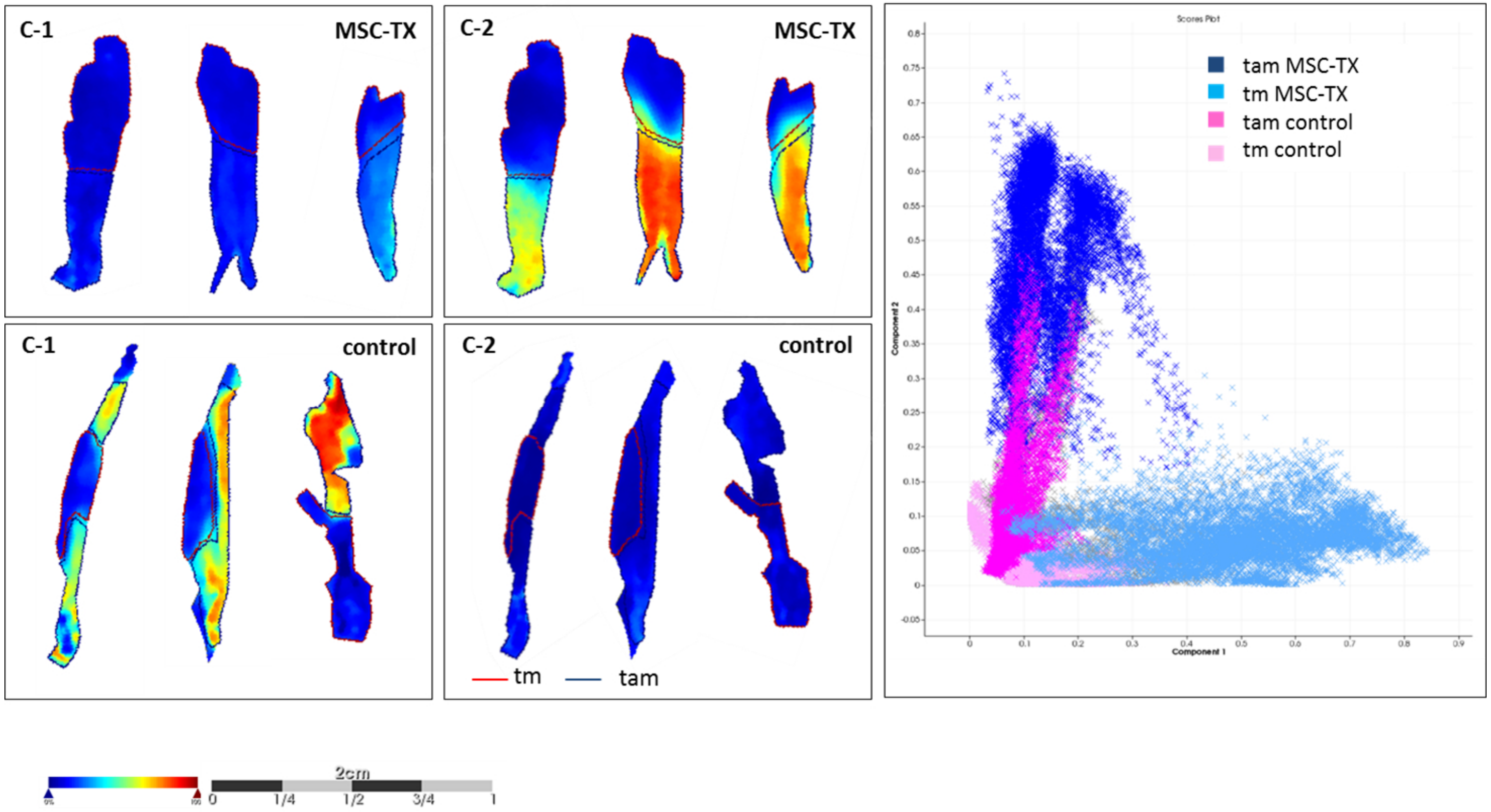

Supplementary figure 4: Probabilistic latent semantic analysis (PLSA) of mass spectra from traumatized (tm), trauma adjacent (tam) region in muscle tissue with MSC-TX and control for all biological replicates A) *Intensity distribution of the first component C1 for the considered tissue sections which shows a clearly high intensity in tam region control and decrease intensity in tm region control , tam-MSC-TX and tm-MSC-TX muscle tissue.* ***B)*** *Intensity distribution of the component 2 results in a high intensity in tam-MSC-TX and low intensity in tm-MSC-TX, tam and tm muscle region control* ***C)*** *Score Plot of the spectra in the first 2 components in pLSA, tm-MSC-TX (violet), tam-MSC-TX (dark blue) , tm (pink) and tam (light blue) muscle region control.*

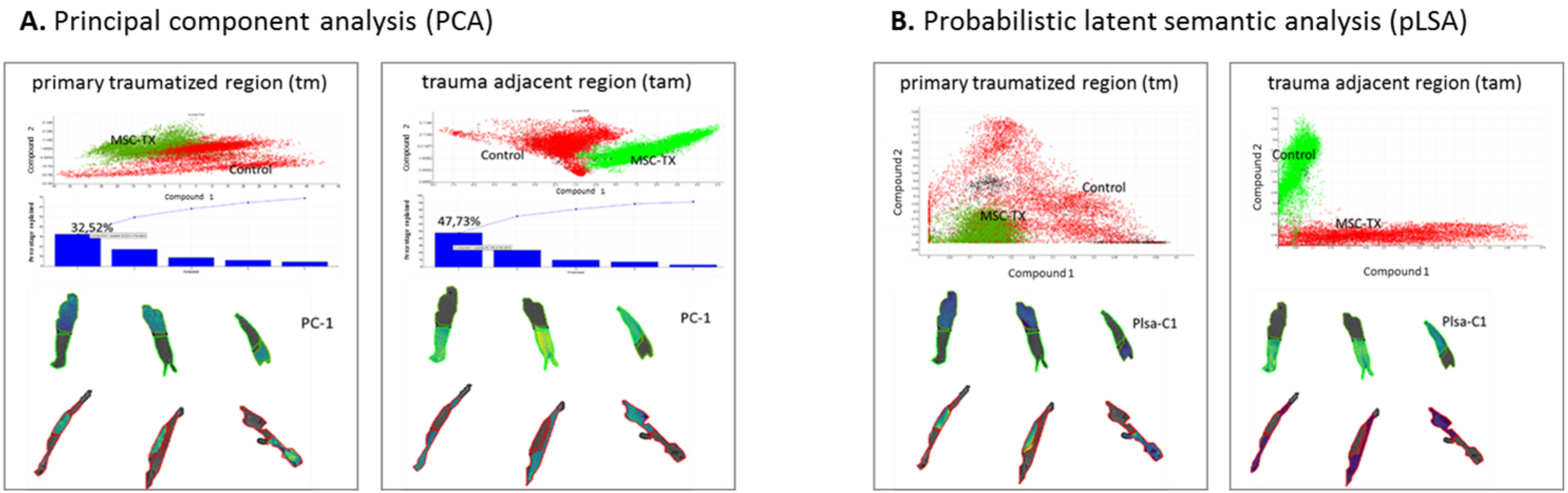

Supplementary figure 5: Probabilistic latent semantic analysis (PLSA) and principle component analysis (PCA) were separately performed for traumatized (tm) or trauma adjacent (tam) region. *PCA and pLSA approaches also discriminate between tm-CTRL and tm-MSC-TX, but to a much lesser extent as the corresponding comparisons between tam-CTRL vs tam-MSC-TX region.* ***A)*** *If the PCA was separately performed for TAM and TM, the PCA Compound 1 (PC1) explained 32% of the total variance between tm-CTRL vs tm-MSC-TX. In contrast, the PC1 increases above 47% of the total variance for the comparisons between tam-CTRL and tam-MSC-TX.* ***B)*** *The PLSA were also separately performed for TM and TAM region, respectively. While the PLSA-Compound 1 (Plsa-C1) only very weakly discriminates between tm-CTRL vs tm-MSC-TX, the plsa-C1 very well separates the tam-CTRL from the tam-MSC-TX region.*

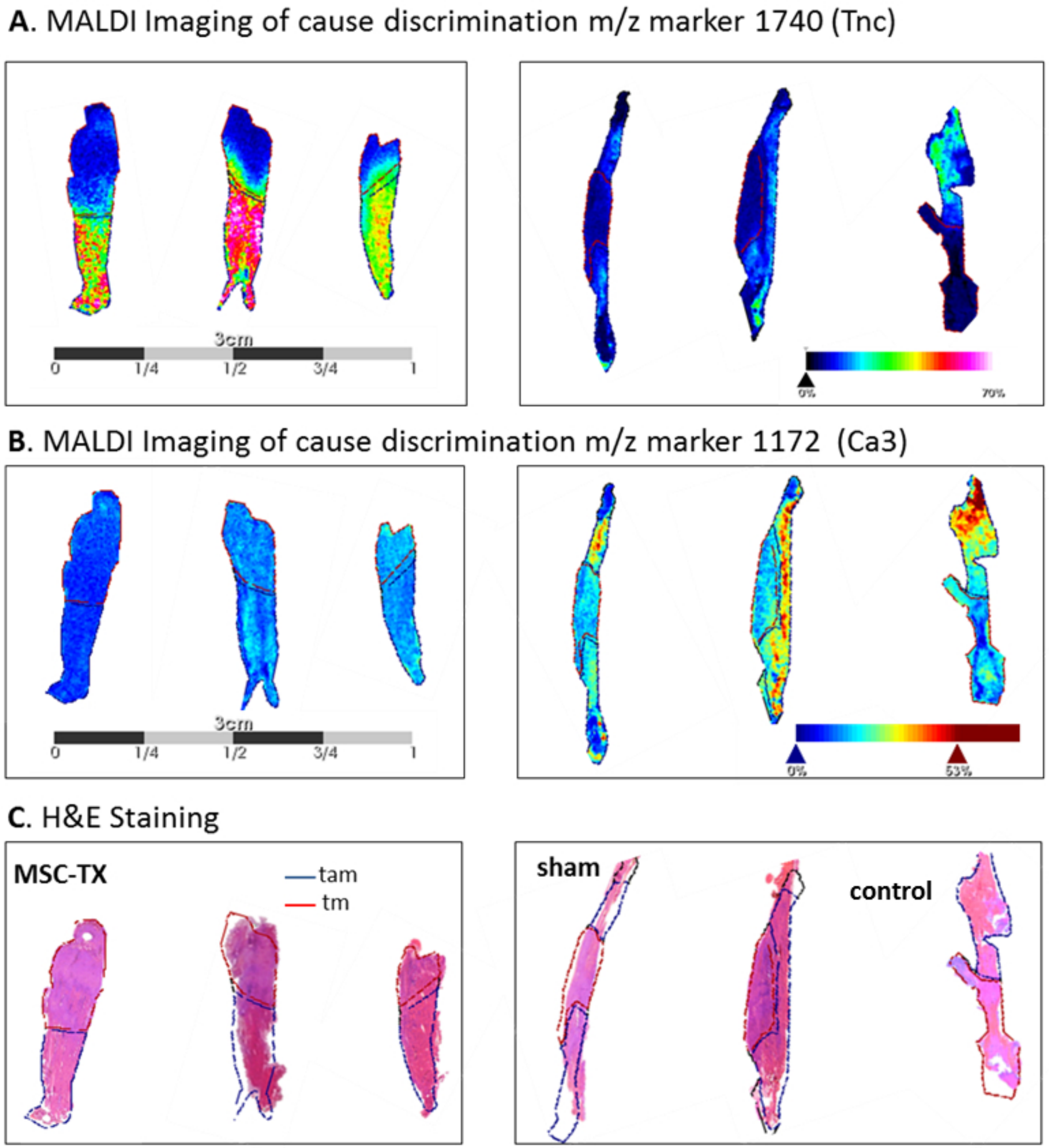

Supplementary figure 6: Spatial distribution of characteristic m/z values for the traumatized, trauma adjacent muscle region with MSC-TX and control for all biological replicates . *Ion density distribution of **(A)** m/z value 1740 (identified as Tnc and **(B)** m/z value 1772 (identified as Ca3) **(C)** Shown are the H&E staining of the soleus muscle in the below subplot and region of interest are indicated by the red (= tm), blue frame.*

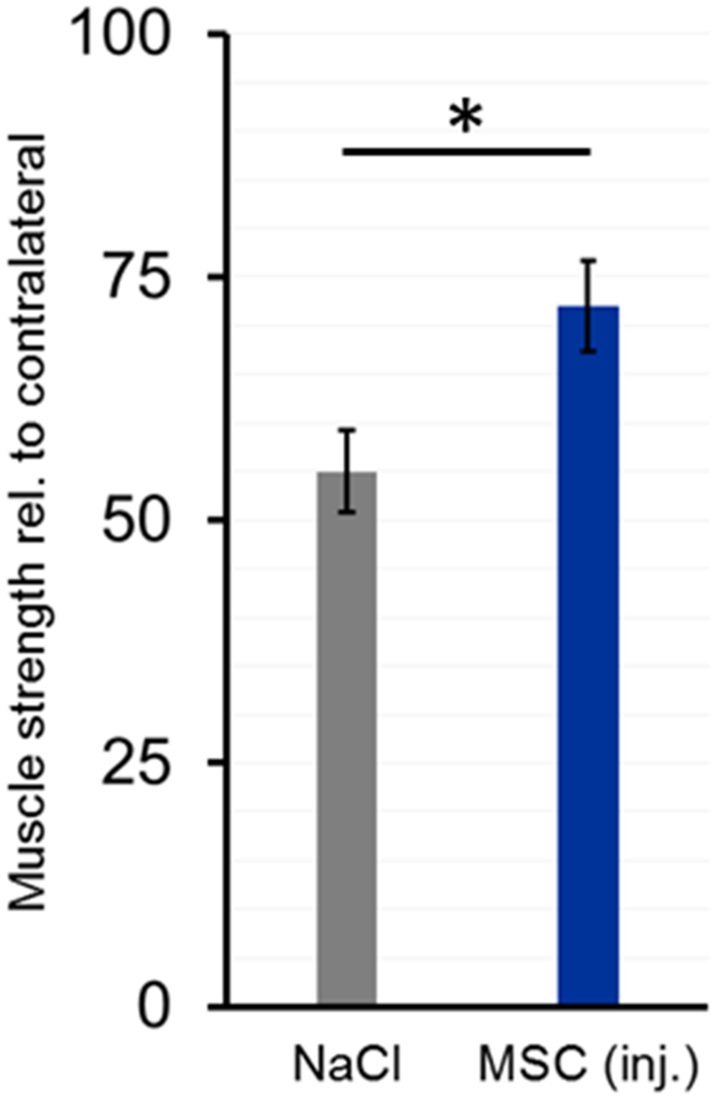

Supplementary figure 7: Recovery of contraction forces in crush trauma model. Functional regeneration was assessed by measuring fast twitch contraction forces at day 28 post injury. *Force is normalized to uninjured (intact) contralateral muscle, and the graphs show the relative fast twitch forces. [n = 8, *p < 0.05]. Error bars represent standard error of the mean.*

Supplementary Tables

Supplementary table 1. Peaks in trauma adjacent and primary trauma muscle tissue with MSC-TX and control

Centroid [m/z]	± [Da]	Trauma adjacent Region					Trauma Region				
		ROC (AUC) TAM-MSC-TX vs TAM-control	Max. intensity TAM-MSC-TX (TIC)	Max. intensity TAM Control (TIC)	Ratio intensity TAM-MSC-TX /TAM-control	Δ[intensity]	ROC (AUC) TM-MSC-TX vs TM-control	Max. intensity TM MSC-TX (TIC)	Max. intensity TM-control (TIC)	Ratio intensity TM-MSC-TX /TM-control	Δ [intensity]
923,519	0,156	0,435	0,482	0,530	0,908	0,049	0,554	0,327	0,307	1,066	0,020
924,540	0,156	0,378	0,407	0,501	0,813	0,093	0,498	0,294	0,293	1,004	0,001
925,483	0,156	0,296	0,338	0,463	0,730	0,125	0,449	0,322	0,368	0,876	0,046
927,533	0,156	0,165	0,283	0,533	0,530	0,251	0,415	0,470	0,551	0,854	0,080
928,481	0,156	0,090	0,280	0,648	0,432	0,368	0,230	0,394	0,642	0,613	0,249
929,509	0,156	0,131	0,283	0,534	0,530	0,251	0,297	0,357	0,493	0,724	0,136
930,540	0,156	0,192	0,265	0,433	0,611	0,168	0,297	0,287	0,407	0,706	0,120
932,525	0,156	0,235	0,367	0,538	0,682	0,171	0,457	0,353	0,371	0,949	0,019
936,595	0,156	0,713	3,082	1,594	1,934	1,488	0,625	0,505	0,286	1,769	0,219
937,556	0,156	0,712	1,920	0,946	2,031	0,975	0,595	0,402	0,284	1,417	0,118
938,598	0,156	0,628	0,671	0,503	1,333	0,168	0,385	0,290	0,346	0,839	0,056
940,527	0,156	0,149	0,378	0,582	0,649	0,204	0,250	0,462	0,624	0,739	0,163
943,512	0,156	0,137	0,414	0,808	0,513	0,393	0,295	0,601	0,818	0,735	0,217
944,564	0,156	0,160	0,989	2,101	0,470	1,113	0,259	1,761	2,621	0,672	0,860
945,536	0,156	0,178	0,744	1,403	0,530	0,659	0,275	1,079	1,536	0,702	0,457
946,510	0,156	0,210	0,363	0,608	0,598	0,245	0,336	0,449	0,622	0,721	0,173
948,543	0,156	0,724	0,764	0,535	1,428	0,229	0,551	0,408	0,353	1,155	0,055
949,521	0,156	0,572	0,525	0,466	1,126	0,059	0,469	0,351	0,370	0,949	0,019
950,582	0,156	0,595	0,742	0,619	1,199	0,123	0,441	0,380	0,397	0,956	0,017

951,563	0,156	0,507	0,484	0,456	1,062	0,028	0,433	0,283	0,306	0,925	0,023
955,582	0,156	0,206	0,303	0,472	0,642	0,169	0,341	0,357	0,462	0,773	0,105
957,558	0,156	0,153	0,312	0,566	0,551	0,254	0,276	0,440	0,613	0,718	0,173
958,549	0,156	0,259	0,492	0,692	0,710	0,201	0,392	0,626	0,692	0,904	0,067
959,541	0,156	0,319	0,437	0,543	0,805	0,106	0,458	0,433	0,445	0,972	0,013
961,528	0,156	0,555	0,690	0,620	1,113	0,070	0,543	0,341	0,324	1,053	0,017
962,524	0,156	0,542	0,492	0,447	1,101	0,045	0,492	0,280	0,280	1,002	0,001
964,520	0,156	0,743	0,709	0,451	1,572	0,258	0,562	0,363	0,341	1,065	0,022
965,520	0,156	0,589	0,463	0,409	1,134	0,055	0,437	0,282	0,318	0,885	0,036
971,549	0,156	0,139	0,333	0,663	0,502	0,330	0,276	0,357	0,513	0,695	0,157
972,558	0,156	0,211	0,445	0,684	0,651	0,239	0,328	0,471	0,603	0,781	0,132
973,569	0,156	0,223	0,347	0,510	0,680	0,163	0,369	0,441	0,510	0,865	0,069
976,441	0,156	0,541	6,437	6,378	1,009	0,058	0,734	2,429	1,671	1,454	0,759
977,457	0,156	0,514	3,528	3,744	0,942	0,217	0,705	1,390	1,002	1,388	0,388
978,475	0,156	0,453	1,369	1,508	0,908	0,139	0,389	0,661	1,098	0,602	0,437
979,579	0,156	0,361	0,621	0,770	0,807	0,149	0,172	0,432	0,963	0,449	0,530
980,514	0,156	0,466	0,815	0,830	0,982	0,015	0,266	0,372	0,570	0,653	0,198
981,536	0,156	0,452	0,494	0,519	0,951	0,026	0,347	0,306	0,384	0,799	0,077
984,609	0,156	0,188	0,298	0,527	0,565	0,229	0,243	0,514	0,976	0,527	0,462
985,550	0,156	0,168	0,397	0,707	0,561	0,311	0,303	0,529	0,822	0,644	0,292
986,578	0,156	0,169	0,351	0,558	0,630	0,206	0,308	0,407	0,541	0,752	0,134
987,522	0,156	0,203	0,409	0,631	0,647	0,223	0,431	0,433	0,469	0,923	0,036
988,553	0,156	0,180	0,443	0,695	0,637	0,252	0,255	0,468	0,621	0,753	0,153
989,500	0,156	0,299	0,620	0,875	0,709	0,255	0,359	0,400	0,494	0,810	0,094
990,533	0,156	0,416	0,592	0,676	0,876	0,084	0,442	0,365	0,386	0,947	0,020
995,548	0,156	0,633	0,727	0,583	1,246	0,144	0,548	0,569	0,508	1,121	0,061
996,590	0,156	0,579	0,454	0,410	1,109	0,045	0,535	0,403	0,386	1,043	0,016
998,503	0,156	0,249	0,327	0,494	0,663	0,167	0,378	0,355	0,440	0,808	0,084
999,548	0,156	0,168	0,321	0,533	0,602	0,212	0,285	0,406	0,543	0,748	0,137
1.000,508	0,156	0,192	0,324	0,517	0,626	0,194	0,262	0,355	0,519	0,683	0,164

1.001,556	0,156	0,233	0,403	0,580	0,696	0,176	0,289	0,438	0,591	0,741	0,153
1.002,518	0,156	0,215	0,415	0,584	0,711	0,169	0,311	0,410	0,498	0,823	0,088
1.003,569	0,156	0,161	0,392	0,630	0,622	0,238	0,238	0,512	0,784	0,653	0,272
1.004,533	0,156	0,181	0,357	0,543	0,657	0,186	0,281	0,436	0,602	0,724	0,166
1.007,609	0,156	0,217	0,420	0,627	0,669	0,207	0,218	0,570	0,861	0,661	0,292
1.008,578	0,156	0,282	0,389	0,522	0,745	0,133	0,260	0,439	0,598	0,734	0,159
1.014,506	0,156	0,291	0,435	0,552	0,788	0,117	0,407	0,394	0,452	0,870	0,059
1.015,572	0,156	0,223	0,322	0,459	0,700	0,138	0,355	0,341	0,403	0,846	0,062
1.018,511	0,156	0,192	0,380	0,573	0,663	0,193	0,404	0,447	0,534	0,837	0,087
1.019,582	0,156	0,321	0,463	0,575	0,806	0,112	0,527	0,571	0,525	1,088	0,046
1.025,589	0,156	0,224	0,464	0,663	0,699	0,200	0,379	0,632	0,696	0,908	0,064
1.026,579	0,156	0,231	0,435	0,610	0,714	0,175	0,385	0,539	0,601	0,897	0,062
1.028,563	0,156	0,302	0,454	0,609	0,746	0,155	0,396	0,519	0,605	0,858	0,086
1.029,557	0,156	0,246	0,363	0,508	0,715	0,145	0,381	0,417	0,494	0,844	0,077
1.032,635	0,156	0,213	0,977	1,423	0,686	0,446	0,314	0,991	1,254	0,790	0,263
1.033,543	0,156	0,204	0,704	1,035	0,681	0,330	0,362	0,668	0,832	0,803	0,163
1.034,542	0,156	0,251	0,499	0,658	0,758	0,159	0,373	0,441	0,518	0,852	0,077
1.039,556	0,156	0,176	0,448	0,722	0,621	0,274	0,314	0,505	0,640	0,789	0,135
1.040,563	0,156	0,190	0,383	0,619	0,619	0,236	0,333	0,468	0,580	0,808	0,111
1.042,579	0,156	0,368	0,849	0,979	0,867	0,130	0,663	1,117	0,838	1,333	0,279
1.043,588	0,156	0,338	0,582	0,704	0,826	0,122	0,596	0,785	0,668	1,176	0,118
1.044,599	0,156	0,231	0,456	0,698	0,653	0,242	0,239	0,531	0,828	0,642	0,297
1.045,519	0,156	0,237	0,405	0,614	0,660	0,209	0,238	0,401	0,658	0,609	0,257
1.046,532	0,156	0,234	0,338	0,510	0,663	0,172	0,246	0,334	0,517	0,646	0,183
1.050,504	0,156	0,454	0,708	0,721	0,982	0,013	0,384	0,328	0,398	0,826	0,069
1.051,522	0,156	0,388	0,599	0,670	0,895	0,070	0,381	0,431	0,474	0,910	0,043
1.052,542	0,156	0,210	0,516	0,746	0,691	0,230	0,245	0,549	0,738	0,744	0,189
1.053,563	0,156	0,327	0,498	0,605	0,824	0,107	0,386	0,540	0,586	0,921	0,046
1.054,585	0,156	0,188	0,460	0,708	0,650	0,248	0,438	0,531	0,542	0,980	0,011
1.055,516	0,156	0,177	0,493	0,849	0,581	0,356	0,334	0,453	0,573	0,791	0,120

1.056,540	0,156	0,160	0,386	0,640	0,602	0,255	0,316	0,376	0,476	0,789	0,100
1.059,994	0,156	0,504	0,239	0,279	0,859	0,039	0,506	0,242	0,212	1,143	0,030
1.070,623	0,156	0,673	0,601	0,443	1,359	0,159	0,452	0,379	0,399	0,950	0,020
1.071,570	0,156	0,458	0,589	0,613	0,960	0,025	0,323	0,462	0,587	0,788	0,124
1.072,517	0,156	0,388	0,691	0,771	0,896	0,080	0,348	0,530	0,624	0,849	0,094
1.073,560	0,156	0,306	0,578	0,718	0,805	0,140	0,267	0,499	0,650	0,767	0,151
1.078,603	0,156	0,068	0,355	0,696	0,510	0,341	0,116	0,484	0,795	0,608	0,311
1.079,557	0,156	0,100	0,318	0,597	0,534	0,278	0,187	0,362	0,561	0,645	0,199
1.081,565	0,156	0,682	1,128	0,862	1,308	0,266	0,419	0,572	0,593	0,964	0,022
1.082,618	0,156	0,666	0,803	0,617	1,301	0,186	0,345	0,445	0,503	0,885	0,058
1.088,576	0,156	0,186	0,462	0,784	0,590	0,321	0,438	0,503	0,507	0,991	0,004
1.089,540	0,156	0,143	0,388	0,655	0,592	0,267	0,455	0,428	0,448	0,954	0,020
1.092,632	0,156	0,208	0,307	0,459	0,668	0,153	0,217	0,395	0,619	0,639	0,223
1.093,600	0,156	0,214	0,280	0,423	0,662	0,143	0,195	0,344	0,548	0,629	0,203
1.094,570	0,156	0,493	0,384	0,411	0,935	0,027	0,363	0,402	0,480	0,837	0,078
1.103,531	0,156	0,160	0,310	0,494	0,629	0,183	0,186	0,421	0,635	0,662	0,215
1.104,510	0,156	0,169	0,354	0,529	0,670	0,175	0,285	0,471	0,583	0,807	0,112
1.105,588	0,156	0,110	0,312	0,632	0,494	0,319	0,282	0,804	1,061	0,758	0,257
1.106,569	0,156	0,176	0,280	0,478	0,586	0,198	0,287	0,564	0,741	0,762	0,177
1.111,587	0,156	0,187	0,302	0,486	0,621	0,184	0,292	0,511	0,670	0,762	0,159
1.113,561	0,156	0,195	0,990	2,064	0,480	1,074	0,444	0,608	0,730	0,832	0,123
1.114,550	0,156	0,172	0,720	1,356	0,531	0,636	0,482	0,516	0,569	0,907	0,053
1.115,539	0,156	0,176	0,477	0,786	0,606	0,310	0,455	0,434	0,457	0,949	0,023
1.127,588	0,156	0,187	0,286	0,465	0,615	0,179	0,375	0,547	0,655	0,835	0,108
1.128,590	0,156	0,213	0,294	0,441	0,666	0,147	0,360	0,462	0,544	0,848	0,083
1.129,593	0,156	0,156	0,418	0,647	0,645	0,230	0,262	0,486	0,645	0,754	0,158
1.130,597	0,156	0,418	0,939	0,951	0,987	0,012	0,506	0,660	0,640	1,031	0,020
1.131,602	0,156	0,362	0,579	0,672	0,862	0,093	0,512	0,486	0,467	1,040	0,019
1.132,608	0,156	0,248	0,387	0,550	0,704	0,163	0,379	0,581	0,658	0,884	0,076
1.133,614	0,156	0,271	0,341	0,441	0,773	0,100	0,349	0,458	0,540	0,849	0,081

1.134,622	0,156	0,233	0,339	0,459	0,739	0,120	0,294	0,470	0,585	0,803	0,115
1.135,631	0,156	0,158	0,314	0,724	0,434	0,410	0,276	0,344	0,492	0,699	0,148
1.136,640	0,156	0,136	0,282	0,580	0,486	0,298	0,339	0,312	0,407	0,766	0,095
1.139,573	0,156	0,110	0,307	0,569	0,540	0,262	0,215	0,456	0,678	0,673	0,222
1.140,587	0,156	0,198	0,387	0,609	0,636	0,222	0,277	0,501	0,680	0,736	0,179
1.141,601	0,156	0,202	0,496	0,802	0,619	0,306	0,382	0,929	1,082	0,858	0,153
1.142,616	0,156	0,184	0,406	0,669	0,607	0,263	0,400	0,749	0,856	0,876	0,106
1.143,632	0,156	0,145	0,290	0,489	0,594	0,198	0,254	0,439	0,591	0,743	0,152
1.144,649	0,156	0,222	0,351	0,504	0,696	0,153	0,345	0,581	0,685	0,849	0,103
1.145,667	0,156	0,264	0,360	0,457	0,788	0,097	0,470	0,519	0,534	0,971	0,015
1.149,545	0,156	0,357	0,500	0,589	0,849	0,089	0,546	0,370	0,339	1,089	0,030
1.150,567	0,156	0,536	0,558	0,532	1,049	0,026	0,616	0,418	0,360	1,161	0,058
1.151,591	0,156	0,264	0,440	0,583	0,756	0,142	0,059	0,362	0,851	0,425	0,490
1.152,615	0,156	0,244	0,352	0,469	0,749	0,118	0,078	0,351	0,672	0,521	0,322
1.153,641	0,156	0,153	0,299	0,459	0,651	0,160	0,148	0,357	0,581	0,615	0,224
1.154,667	0,156	0,195	0,345	0,500	0,690	0,155	0,209	0,398	0,562	0,708	0,164
1.156,620	0,156	0,168	0,420	0,673	0,625	0,253	0,248	0,536	0,763	0,702	0,227
1.157,649	0,156	0,152	0,364	0,558	0,652	0,194	0,299	0,518	0,670	0,773	0,152
1.158,576	0,156	0,178	0,328	0,501	0,655	0,173	0,290	0,438	0,572	0,766	0,134
1.159,607	0,156	0,283	0,370	0,489	0,755	0,120	0,352	0,455	0,520	0,876	0,064
1.163,740	0,156	0,699	0,861	0,574	1,499	0,287	0,473	0,435	0,447	0,973	0,012
1.164,672	0,156	0,562	0,773	0,663	1,166	0,110	0,508	0,456	0,444	1,028	0,013
1.167,577	0,156	0,191	0,294	0,463	0,636	0,169	0,475	0,494	0,481	1,026	0,012
1.168,616	0,156	0,220	0,338	0,477	0,707	0,140	0,426	0,451	0,480	0,940	0,029
1.171,531	0,156	0,069	0,354	1,080	0,328	0,726	0,281	0,414	0,578	0,717	0,164
1.172,574	0,156	0,097	0,464	1,063	0,436	0,599	0,251	0,441	0,608	0,725	0,167
1.173,617	0,156	0,119	0,428	0,810	0,529	0,382	0,239	0,436	0,632	0,690	0,196
1.174,662	0,156	0,142	0,333	0,540	0,616	0,207	0,325	0,430	0,544	0,791	0,114
1.177,592	0,156	0,229	0,399	0,573	0,695	0,175	0,350	0,416	0,494	0,842	0,078
1.178,641	0,156	0,201	0,360	0,522	0,690	0,162	0,395	0,557	0,624	0,893	0,067

1.179,585	0,156	0,191	0,277	0,425	0,652	0,148	0,410	0,431	0,480	0,897	0,049
1.183,580	0,156	0,518	0,608	0,588	1,034	0,020	0,570	0,476	0,418	1,137	0,057
1.184,529	0,156	0,764	2,072	1,284	1,614	0,788	0,785	1,017	0,609	1,672	0,409
1.185,583	0,156	0,723	1,485	0,975	1,522	0,509	0,665	0,764	0,578	1,321	0,186
1.186,639	0,156	0,604	0,735	0,603	1,219	0,132	0,560	0,489	0,464	1,055	0,025
1.187,590	0,156	0,230	0,604	0,824	0,733	0,220	0,285	0,658	0,824	0,799	0,165
1.188,647	0,156	0,194	0,544	0,787	0,691	0,243	0,267	0,692	0,897	0,771	0,205
1.189,599	0,156	0,145	0,469	0,857	0,547	0,388	0,279	0,558	0,712	0,784	0,154
1.190,659	0,156	0,155	0,334	0,572	0,584	0,238	0,301	0,373	0,485	0,770	0,112
1.192,673	0,156	0,277	0,419	0,513	0,817	0,094	0,475	0,464	0,472	0,984	0,008
1.194,692	0,156	0,198	0,404	0,602	0,672	0,198	0,347	0,548	0,657	0,833	0,110
1.195,649	0,156	0,263	0,545	0,706	0,772	0,161	0,446	0,713	0,758	0,940	0,045
1.196,607	0,156	0,325	0,513	0,610	0,841	0,097	0,424	0,555	0,602	0,921	0,048
1.197,673	0,156	0,305	0,436	0,518	0,843	0,081	0,345	0,436	0,510	0,854	0,074
1.198,632	0,156	0,786	17,398	9,604	1,812	7,794	0,869	6,823	2,752	2,479	4,071
1.199,699	0,156	0,773	11,717	6,739	1,739	4,978	0,857	4,966	2,159	2,300	2,807
1.200,661	0,156	0,753	4,082	2,556	1,597	1,526	0,822	1,881	1,000	1,882	0,881
1.201,730	0,156	0,654	1,419	1,082	1,311	0,337	0,708	0,783	0,546	1,432	0,236
1.202,693	0,156	0,403	0,596	0,637	0,936	0,041	0,441	0,442	0,456	0,969	0,014
1.203,656	0,156	0,488	0,584	0,562	1,040	0,023	0,333	0,532	0,633	0,840	0,101
1.210,639	0,156	0,684	0,980	0,767	1,278	0,213	0,570	0,675	0,612	1,103	0,063
1.211,717	0,156	0,609	0,860	0,747	1,152	0,113	0,385	0,604	0,651	0,927	0,048
1.212,580	0,156	0,436	0,674	0,691	0,974	0,018	0,572	0,944	0,789	1,196	0,155
1.213,659	0,156	0,321	0,466	0,555	0,840	0,089	0,537	0,650	0,611	1,063	0,038
1.215,605	0,156	0,218	0,409	0,585	0,699	0,176	0,298	0,474	0,585	0,810	0,111
1.219,614	0,156	0,222	0,412	0,568	0,725	0,156	0,461	0,616	0,611	1,008	0,005
1.220,591	0,156	0,333	0,470	0,564	0,835	0,093	0,440	0,564	0,571	0,988	0,007
1.221,569	0,156	0,251	0,383	0,520	0,736	0,137	0,355	0,421	0,507	0,829	0,087
1.225,597	0,156	0,305	0,428	0,524	0,816	0,097	0,515	0,487	0,448	1,086	0,038
1.226,579	0,156	0,295	0,368	0,455	0,810	0,087	0,496	0,440	0,456	0,966	0,015

1.228,654	0,156	0,333	0,448	0,525	0,853	0,077	0,521	0,489	0,470	1,040	0,019
1.231,609	0,156	0,177	0,450	0,685	0,657	0,235	0,302	0,606	0,764	0,794	0,157
1.232,595	0,156	0,166	0,409	0,623	0,657	0,214	0,316	0,526	0,671	0,784	0,145
1.233,583	0,156	0,115	0,436	0,780	0,559	0,344	0,263	0,688	0,966	0,713	0,277
1.234,680	0,156	0,111	0,421	0,691	0,609	0,270	0,249	0,611	0,830	0,736	0,220
1.235,669	0,156	0,198	0,391	0,541	0,722	0,150	0,321	0,460	0,559	0,822	0,099
1.242,613	0,156	0,322	0,581	0,692	0,839	0,111	0,368	0,545	0,601	0,907	0,056
1.243,608	0,156	0,240	0,763	1,021	0,747	0,259	0,259	0,775	0,998	0,776	0,223
1.244,604	0,156	0,258	0,620	0,798	0,777	0,178	0,269	0,618	0,796	0,776	0,178
1.245,600	0,156	0,222	0,410	0,549	0,748	0,138	0,379	0,450	0,520	0,866	0,070
1.247,707	0,156	0,191	0,339	0,511	0,662	0,173	0,390	0,522	0,602	0,867	0,080
1.248,706	0,156	0,179	0,303	0,445	0,682	0,141	0,376	0,496	0,551	0,900	0,055
1.252,598	0,156	0,254	0,381	0,502	0,759	0,121	0,291	0,385	0,519	0,743	0,134
1.253,601	0,156	0,248	0,413	0,524	0,789	0,111	0,379	0,445	0,524	0,849	0,079
1.254,716	0,156	0,324	0,703	0,897	0,783	0,195	0,340	1,086	1,380	0,787	0,294
1.255,720	0,156	0,341	0,590	0,729	0,810	0,139	0,372	0,896	1,093	0,819	0,198
1.256,725	0,156	0,341	0,440	0,526	0,838	0,085	0,390	0,594	0,691	0,860	0,097
1.257,619	0,156	0,440	0,606	0,626	0,969	0,019	0,378	0,466	0,539	0,864	0,073
1.258,626	0,156	0,341	0,577	0,702	0,821	0,126	0,334	0,676	0,787	0,858	0,112
1.259,633	0,156	0,276	0,480	0,614	0,782	0,134	0,370	0,642	0,712	0,902	0,069
1.260,641	0,156	0,391	0,605	0,679	0,891	0,074	0,482	0,565	0,555	1,017	0,010
1.261,650	0,156	0,353	0,467	0,574	0,815	0,106	0,488	0,414	0,420	0,985	0,006
1.262,660	0,156	0,312	0,510	0,638	0,800	0,128	0,342	0,487	0,567	0,858	0,080
1.263,670	0,156	0,269	0,487	0,606	0,803	0,119	0,425	0,510	0,541	0,943	0,031
1.268,621	0,156	0,165	0,346	0,523	0,661	0,178	0,267	0,410	0,552	0,742	0,142
1.269,636	0,156	0,334	0,598	0,697	0,859	0,099	0,355	0,523	0,605	0,864	0,082
1.270,652	0,156	0,356	0,494	0,564	0,875	0,070	0,383	0,471	0,515	0,913	0,045
1.271,668	0,156	0,251	0,445	0,567	0,785	0,122	0,370	0,445	0,496	0,897	0,051
1.274,722	0,156	0,265	0,477	0,664	0,718	0,188	0,243	0,498	0,759	0,655	0,262
1.275,628	0,156	0,244	0,428	0,616	0,695	0,188	0,232	0,461	0,715	0,645	0,254

1.276,649	0,156	0,232	0,367	0,518	0,709	0,151	0,287	0,486	0,623	0,780	0,137
1.286,665	0,156	0,202	0,539	0,806	0,669	0,267	0,303	0,661	0,836	0,790	0,175
1.287,693	0,156	0,200	0,413	0,612	0,674	0,199	0,249	0,527	0,716	0,736	0,189
1.288,723	0,156	0,455	0,509	0,486	1,049	0,024	0,353	0,431	0,494	0,873	0,063
1.291,700	0,156	0,718	2,521	1,528	1,650	0,993	0,642	0,596	0,460	1,297	0,137
1.292,733	0,156	0,696	1,755	1,133	1,549	0,622	0,602	0,458	0,373	1,230	0,086
1.293,651	0,156	0,592	0,847	0,728	1,164	0,120	0,354	0,441	0,508	0,867	0,068
1.297,675	0,156	0,169	0,309	0,474	0,653	0,165	0,175	0,409	0,630	0,649	0,221
1.298,712	0,156	0,265	0,608	0,806	0,754	0,198	0,221	0,491	0,684	0,717	0,193
1.299,634	0,156	0,241	0,516	0,717	0,719	0,202	0,228	0,474	0,689	0,687	0,216
1.300,672	0,156	0,236	0,470	0,632	0,743	0,162	0,255	0,467	0,631	0,739	0,165
1.301,711	0,156	0,239	0,362	0,485	0,746	0,123	0,342	0,446	0,526	0,847	0,081
1.303,676	0,156	0,187	0,423	0,616	0,687	0,193	0,308	0,520	0,659	0,790	0,138
1.304,717	0,156	0,224	0,367	0,490	0,749	0,123	0,343	0,491	0,574	0,855	0,083
1.307,729	0,156	0,310	0,424	0,501	0,846	0,077	0,335	0,489	0,571	0,857	0,082
1.308,657	0,156	0,278	0,455	0,558	0,815	0,103	0,365	0,534	0,603	0,886	0,069
1.323,707	0,156	0,178	0,470	0,768	0,613	0,297	0,252	0,821	1,169	0,703	0,348
1.324,762	0,156	0,165	0,366	0,602	0,607	0,237	0,215	0,592	0,923	0,642	0,330
1.325,702	0,156	0,088	0,444	1,105	0,402	0,660	0,164	0,643	1,148	0,560	0,505
1.326,759	0,156	0,090	0,420	0,935	0,449	0,516	0,170	0,550	0,910	0,605	0,360
1.327,699	0,156	0,116	0,390	0,668	0,584	0,278	0,258	0,543	0,694	0,783	0,151
1.328,641	0,156	0,125	0,434	0,809	0,537	0,375	0,232	0,741	1,110	0,668	0,369
1.329,700	0,156	0,163	0,414	0,711	0,583	0,297	0,210	0,702	1,115	0,629	0,413
1.330,642	0,156	0,204	0,350	0,511	0,685	0,161	0,243	0,485	0,688	0,705	0,203
1.332,647	0,156	0,320	0,485	0,575	0,844	0,090	0,526	0,452	0,426	1,062	0,027
1.333,709	0,156	0,251	0,389	0,507	0,768	0,118	0,465	0,419	0,427	0,983	0,007
1.338,677	0,156	0,190	0,419	0,616	0,679	0,197	0,306	0,389	0,483	0,805	0,094
1.341,642	0,156	0,110	0,384	0,703	0,546	0,319	0,176	0,488	0,773	0,632	0,285
1.342,711	0,156	0,146	0,442	0,703	0,629	0,261	0,204	0,456	0,682	0,668	0,227
1.345,684	0,156	0,235	0,442	0,568	0,777	0,127	0,329	0,495	0,573	0,864	0,078

1.346,636	0,156	0,228	0,368	0,485	0,759	0,117	0,420	0,548	0,565	0,970	0,017
1.347,709	0,156	0,158	0,359	0,525	0,683	0,167	0,357	0,610	0,680	0,897	0,070
1.348,663	0,156	0,178	0,390	0,728	0,536	0,338	0,343	0,484	0,568	0,854	0,083
1.349,737	0,156	0,202	0,418	0,724	0,578	0,305	0,354	0,491	0,593	0,827	0,103
1.351,648	0,156	0,263	0,335	0,426	0,787	0,091	0,388	0,442	0,504	0,878	0,062
1.352,724	0,156	0,238	0,343	0,436	0,787	0,093	0,331	0,441	0,506	0,872	0,065
1.353,681	0,156	0,259	0,377	0,480	0,785	0,103	0,348	0,444	0,521	0,853	0,077
1.354,638	0,156	0,449	1,158	1,218	0,951	0,060	0,520	0,674	0,617	1,092	0,057
1.355,597	0,156	0,446	0,928	0,981	0,946	0,053	0,586	0,539	0,468	1,150	0,070
1.356,676	0,156	0,370	0,515	0,599	0,860	0,084	0,532	0,365	0,340	1,075	0,025
1.360,758	0,156	0,234	0,509	0,735	0,692	0,226	0,483	0,483	0,496	0,974	0,013
1.361,720	0,156	0,169	0,493	0,853	0,578	0,360	0,168	0,605	1,278	0,473	0,673
1.362,683	0,156	0,138	0,481	0,879	0,547	0,398	0,160	0,668	1,278	0,522	0,610
1.363,767	0,156	0,340	0,856	1,013	0,845	0,158	0,325	1,385	1,749	0,792	0,363
1.364,731	0,156	0,346	0,711	0,891	0,797	0,180	0,348	1,195	1,424	0,839	0,230
1.365,696	0,156	0,293	0,472	0,586	0,805	0,114	0,364	0,693	0,793	0,874	0,100
1.366,661	0,156	0,208	0,502	0,684	0,735	0,181	0,387	0,543	0,595	0,912	0,052
1.374,648	0,156	0,310	0,607	0,898	0,676	0,291	0,419	0,385	0,424	0,909	0,039
1.375,740	0,156	0,310	0,643	0,898	0,717	0,254	0,454	0,471	0,505	0,932	0,034
1.376,712	0,156	0,236	0,427	0,598	0,715	0,170	0,415	0,416	0,439	0,949	0,022
1.377,684	0,156	0,278	0,617	0,777	0,795	0,159	0,414	0,765	0,858	0,891	0,093
1.378,657	0,156	0,284	0,466	0,590	0,790	0,124	0,342	0,555	0,646	0,859	0,091
1.384,751	0,156	0,263	0,438	0,548	0,800	0,109	0,279	0,439	0,541	0,812	0,102
1.385,728	0,156	0,125	0,419	0,718	0,584	0,299	0,179	0,522	0,799	0,654	0,276
1.386,706	0,156	0,106	0,361	0,616	0,586	0,255	0,191	0,483	0,676	0,714	0,193
1.390,746	0,156	0,111	0,352	0,690	0,510	0,338	0,310	0,388	0,490	0,792	0,102
1.393,691	0,156	0,553	0,522	0,468	1,115	0,054	0,528	0,377	0,357	1,057	0,020
1.394,673	0,156	0,601	0,563	0,487	1,157	0,077	0,537	0,456	0,447	1,022	0,010
1.396,764	0,156	0,708	2,056	1,322	1,556	0,735	0,335	0,592	0,723	0,819	0,131
1.397,748	0,156	0,660	1,406	1,053	1,336	0,353	0,269	0,541	0,689	0,785	0,148

1.398,734	0,156	0,523	0,893	0,790	1,131	0,103	0,403	0,554	0,622	0,892	0,067
1.399,719	0,156	0,253	0,451	0,571	0,791	0,119	0,266	0,462	0,577	0,800	0,116
1.403,669	0,156	0,221	0,330	0,455	0,724	0,125	0,359	0,619	0,704	0,878	0,086
1.404,782	0,156	0,231	0,308	0,417	0,738	0,109	0,354	0,514	0,592	0,869	0,078
1.408,744	0,156	0,255	0,394	0,526	0,748	0,133	0,314	0,520	0,643	0,808	0,123
1.409,736	0,156	0,269	0,365	0,474	0,770	0,109	0,357	0,454	0,554	0,819	0,100
1.410,728	0,156	0,152	0,346	0,545	0,634	0,200	0,297	0,456	0,593	0,769	0,137
1.411,722	0,156	0,137	0,338	0,539	0,627	0,201	0,224	0,424	0,593	0,715	0,169
1.412,715	0,156	0,223	0,386	0,525	0,736	0,139	0,403	0,481	0,562	0,855	0,082
1.416,822	0,156	0,234	0,296	0,410	0,721	0,114	0,411	0,582	0,650	0,896	0,068
1.417,694	0,156	0,296	0,328	0,410	0,801	0,081	0,391	0,494	0,581	0,851	0,087
1.430,709	0,156	0,082	0,385	1,705	0,226	1,319	0,330	0,368	0,536	0,687	0,168
1.431,714	0,156	0,057	0,374	1,483	0,252	1,109	0,231	0,595	0,856	0,695	0,261
1.432,720	0,156	0,054	0,285	0,970	0,293	0,685	0,260	0,527	0,747	0,706	0,219
1.433,727	0,156	0,069	0,288	0,609	0,472	0,322	0,313	0,425	0,509	0,834	0,084
1.438,769	0,156	0,577	0,615	0,547	1,125	0,068	0,362	0,442	0,508	0,869	0,066
1.439,779	0,156	0,515	0,604	0,571	1,057	0,033	0,409	0,506	0,545	0,928	0,039
1.440,790	0,156	0,376	0,483	0,542	0,892	0,058	0,473	0,485	0,508	0,956	0,022
1.443,699	0,156	0,175	0,350	0,559	0,626	0,209	0,232	0,511	0,744	0,688	0,232
1.444,713	0,156	0,133	0,381	0,729	0,523	0,347	0,209	0,786	1,325	0,593	0,539
1.445,727	0,156	0,189	0,425	0,644	0,660	0,219	0,223	0,726	1,092	0,664	0,367
1.446,741	0,156	0,655	0,942	0,695	1,355	0,247	0,398	0,599	0,666	0,898	0,068
1.447,756	0,156	0,677	0,682	0,515	1,325	0,167	0,482	0,434	0,424	1,022	0,009
1.452,713	0,156	0,249	0,520	0,716	0,726	0,196	0,229	0,670	0,932	0,719	0,262
1.453,732	0,156	0,242	0,462	0,648	0,713	0,186	0,226	0,628	0,901	0,697	0,273
1.454,751	0,156	0,313	0,479	0,556	0,861	0,077	0,303	0,589	0,701	0,841	0,111
1.455,771	0,156	0,432	0,459	0,483	0,951	0,024	0,409	0,511	0,553	0,923	0,042
1.456,791	0,156	0,298	0,341	0,436	0,781	0,095	0,344	0,497	0,592	0,840	0,095
1.457,812	0,156	0,309	0,450	0,527	0,855	0,076	0,392	0,604	0,673	0,898	0,068
1.458,834	0,156	0,278	0,716	1,044	0,685	0,328	0,455	1,201	1,279	0,939	0,078

1.459,856	0,156	0,321	0,647	0,795	0,814	0,148	0,458	0,908	0,949	0,956	0,041
1.460,751	0,156	0,339	0,527	0,598	0,881	0,071	0,498	0,625	0,634	0,987	0,009
1.461,774	0,156	0,281	0,415	0,518	0,801	0,103	0,433	0,480	0,523	0,919	0,043
1.462,798	0,156	0,101	0,376	0,885	0,425	0,509	0,374	0,375	0,433	0,867	0,058
1.463,823	0,156	0,095	0,333	0,738	0,451	0,405	0,405	0,434	0,462	0,940	0,028
1.464,720	0,156	0,113	0,312	0,583	0,535	0,271	0,530	0,506	0,476	1,063	0,030
1.467,799	0,156	0,254	0,285	0,392	0,727	0,107	0,283	0,397	0,565	0,702	0,168
1.468,827	0,156	0,256	0,334	0,442	0,756	0,108	0,273	0,419	0,602	0,696	0,183
1.472,814	0,156	0,478	0,477	0,467	1,021	0,010	0,399	0,446	0,482	0,925	0,036
1.473,845	0,156	0,801	1,115	0,578	1,929	0,537	0,555	0,494	0,419	1,178	0,075
1.474,747	0,156	0,774	0,894	0,546	1,636	0,347	0,565	0,441	0,404	1,093	0,037
1.475,779	0,156	0,511	0,562	0,524	1,074	0,039	0,362	0,408	0,469	0,869	0,061
1.476,811	0,156	0,223	0,448	0,618	0,725	0,170	0,295	0,387	0,509	0,759	0,122
1.479,782	0,156	0,334	0,386	0,450	0,858	0,064	0,436	0,449	0,471	0,952	0,023
1.480,817	0,156	0,321	0,357	0,424	0,842	0,067	0,471	0,537	0,532	1,010	0,005
1.481,852	0,156	0,206	0,359	0,576	0,624	0,217	0,349	0,534	0,652	0,818	0,119
1.482,758	0,156	0,225	0,361	0,525	0,687	0,165	0,353	0,457	0,530	0,862	0,073
1.487,816	0,156	0,815	3,193	1,483	2,153	1,709	0,672	0,770	0,468	1,645	0,302
1.488,856	0,156	0,822	3,431	1,428	2,402	2,003	0,698	0,756	0,422	1,792	0,334
1.489,896	0,156	0,728	1,740	1,118	1,556	0,621	0,620	0,688	0,537	1,281	0,151
1.490,806	0,156	0,664	1,010	0,799	1,265	0,212	0,508	0,528	0,500	1,055	0,027
1.491,847	0,156	0,381	0,666	0,732	0,909	0,066	0,512	0,472	0,465	1,015	0,007
1.492,758	0,156	0,253	0,457	0,593	0,771	0,136	0,546	0,424	0,404	1,050	0,020
1.495,756	0,156	0,239	0,337	0,476	0,709	0,139	0,333	0,503	0,640	0,785	0,138
1.496,800	0,156	0,210	0,325	0,494	0,657	0,170	0,291	0,561	0,734	0,765	0,173
1.500,721	0,156	0,373	0,774	0,955	0,811	0,180	0,540	0,425	0,419	1,014	0,006
1.501,768	0,156	0,380	0,727	0,857	0,849	0,129	0,489	0,413	0,416	0,993	0,003
1.502,815	0,156	0,410	0,564	0,603	0,935	0,039	0,469	0,423	0,419	1,009	0,004
1.503,863	0,156	0,319	0,469	0,548	0,857	0,079	0,368	0,563	0,680	0,828	0,117
1.504,781	0,156	0,810	1,912	0,928	2,059	0,983	0,511	0,726	0,742	0,978	0,016

1.505,830	0,156	0,770	1,904	1,069	1,782	0,836	0,454	0,697	0,731	0,955	0,033
1.506,880	0,156	0,596	0,969	0,765	1,267	0,205	0,295	0,541	0,691	0,783	0,150
1.507,799	0,156	0,436	0,676	0,719	0,941	0,043	0,372	0,529	0,620	0,853	0,091
1.508,850	0,156	0,488	0,543	0,543	0,999	0,000	0,509	0,505	0,506	0,999	0,000
1.514,773	0,156	0,308	0,387	0,479	0,809	0,091	0,491	0,477	0,491	0,972	0,014
1.515,828	0,156	0,583	1,000	0,825	1,212	0,175	0,695	0,895	0,657	1,362	0,238
1.516,751	0,156	0,617	1,107	0,874	1,266	0,233	0,659	0,884	0,706	1,251	0,177
1.517,807	0,156	0,504	0,833	0,781	1,066	0,051	0,594	0,676	0,591	1,143	0,085
1.518,864	0,156	0,539	0,663	0,605	1,096	0,058	0,575	0,461	0,426	1,082	0,035
1.524,819	0,156	0,208	0,357	0,507	0,705	0,149	0,310	0,446	0,558	0,800	0,112
1.525,880	0,156	0,268	0,334	0,426	0,784	0,092	0,300	0,402	0,519	0,774	0,117
1.527,738	0,156	0,265	0,359	0,500	0,718	0,141	0,180	0,405	0,647	0,626	0,242
1.528,800	0,156	0,325	0,428	0,517	0,829	0,089	0,227	0,429	0,597	0,719	0,168
1.529,730	0,156	0,343	0,390	0,460	0,849	0,069	0,463	0,478	0,495	0,965	0,017
1.532,790	0,156	0,545	0,578	0,531	1,088	0,047	0,675	0,903	0,692	1,306	0,211
1.533,855	0,156	0,599	0,595	0,521	1,143	0,075	0,618	0,797	0,670	1,190	0,128
1.534,788	0,156	0,542	0,448	0,422	1,061	0,026	0,510	0,491	0,479	1,025	0,012
1.543,872	0,156	0,414	0,409	0,440	0,929	0,031	0,477	0,481	0,512	0,939	0,031
1.547,894	0,156	0,356	0,383	0,443	0,864	0,060	0,260	0,447	0,681	0,657	0,234
1.548,833	0,156	0,292	0,402	0,508	0,790	0,107	0,303	0,664	0,861	0,772	0,196
1.549,773	0,156	0,316	0,387	0,473	0,818	0,086	0,353	0,612	0,720	0,851	0,107
1.550,848	0,156	0,345	0,364	0,440	0,826	0,077	0,369	0,525	0,600	0,875	0,075
1.556,906	0,156	0,382	0,385	0,432	0,891	0,047	0,347	0,394	0,492	0,800	0,098
1.557,850	0,156	0,366	0,366	0,429	0,853	0,063	0,350	0,390	0,496	0,786	0,106
1.561,901	0,156	0,255	0,314	0,400	0,785	0,086	0,360	0,461	0,535	0,862	0,074
1.563,930	0,156	0,295	0,375	0,476	0,789	0,100	0,330	0,507	0,614	0,825	0,107
1.564,878	0,156	0,294	0,466	0,580	0,803	0,114	0,349	0,577	0,673	0,856	0,097
1.565,826	0,156	0,254	0,413	0,538	0,768	0,125	0,372	0,459	0,520	0,882	0,061
1.566,774	0,156	0,278	0,448	0,569	0,787	0,121	0,417	0,522	0,570	0,915	0,048
1.567,859	0,156	0,316	0,445	0,516	0,864	0,070	0,473	0,592	0,599	0,988	0,007

1.568,808	0,156	0,376	0,379	0,422	0,896	0,044	0,581	0,529	0,470	1,126	0,059
1.570,844	0,156	0,318	0,308	0,405	0,760	0,097	0,326	0,487	0,613	0,795	0,126
1.571,795	0,156	0,420	0,386	0,424	0,911	0,038	0,376	0,481	0,626	0,769	0,144
1.572,746	0,156	0,302	0,717	1,078	0,665	0,361	0,220	0,535	0,971	0,551	0,436
1.573,834	0,156	0,293	0,620	0,894	0,693	0,274	0,195	0,486	0,856	0,567	0,371
1.574,787	0,156	0,237	0,449	0,703	0,638	0,255	0,249	0,436	0,656	0,664	0,220
1.575,876	0,156	0,197	0,375	0,546	0,688	0,170	0,295	0,452	0,576	0,784	0,124
1.576,829	0,156	0,137	0,319	0,558	0,572	0,239	0,224	0,437	0,667	0,655	0,230
1.577,783	0,156	0,123	0,332	0,584	0,569	0,252	0,275	0,381	0,533	0,714	0,152
1.583,789	0,156	0,425	0,342	0,371	0,922	0,029	0,630	0,496	0,426	1,165	0,070
1.584,883	0,156	0,381	0,310	0,347	0,891	0,038	0,630	0,453	0,393	1,153	0,060
1.585,841	0,156	0,265	0,349	0,458	0,761	0,109	0,396	0,496	0,551	0,899	0,056
1.592,833	0,156	0,258	0,424	0,599	0,708	0,175	0,418	0,433	0,460	0,941	0,027
1.593,794	0,156	0,216	0,382	0,556	0,687	0,174	0,410	0,385	0,437	0,882	0,052
1.599,987	0,156	0,295	0,412	0,508	0,811	0,096	0,479	0,506	0,531	0,954	0,024
1.600,952	0,156	0,262	0,386	0,529	0,730	0,143	0,447	0,489	0,522	0,937	0,033
1.601,918	0,156	0,211	0,402	0,563	0,714	0,161	0,370	0,467	0,538	0,867	0,072
1.602,884	0,156	0,577	0,551	0,487	1,130	0,064	0,460	0,468	0,484	0,965	0,017
1.603,850	0,156	0,713	0,500	0,385	1,300	0,116	0,527	0,402	0,390	1,030	0,012
1.613,818	0,156	0,968	1,641	0,457	3,593	1,184	0,699	0,511	0,333	1,535	0,178
1.614,790	0,156	0,970	1,563	0,430	3,630	1,132	0,694	0,485	0,343	1,415	0,142
1.615,901	0,156	0,923	0,991	0,451	2,200	0,541	0,382	0,479	0,528	0,906	0,049
1.616,873	0,156	0,821	0,680	0,437	1,556	0,243	0,361	0,393	0,457	0,859	0,064
1.617,847	0,156	0,761	0,574	0,400	1,434	0,173	0,569	0,415	0,375	1,107	0,040
1.620,908	0,156	0,222	0,270	0,394	0,684	0,124	0,314	0,359	0,518	0,693	0,159
1.621,883	0,156	0,216	0,313	0,460	0,681	0,147	0,323	0,449	0,591	0,761	0,142
1.628,862	0,156	0,426	0,789	0,886	0,890	0,097	0,409	0,461	0,515	0,895	0,054
1.629,841	0,156	0,385	0,761	0,881	0,864	0,120	0,343	0,477	0,594	0,804	0,116
1.630,820	0,156	0,387	0,618	0,711	0,869	0,093	0,358	0,404	0,499	0,809	0,095
1.634,882	0,156	0,335	0,381	0,470	0,811	0,089	0,527	0,422	0,418	1,011	0,004

1.636,986	0,156	0,411	0,408	0,435	0,939	0,027	0,736	0,614	0,465	1,321	0,149
1.637,829	0,156	0,487	0,404	0,408	0,991	0,004	0,797	0,601	0,405	1,484	0,196
1.638,952	0,156	0,279	0,305	0,396	0,769	0,091	0,585	0,459	0,413	1,111	0,046
1.639,936	0,156	0,288	0,359	0,460	0,781	0,101	0,552	0,459	0,438	1,049	0,021
1.644,861	0,156	0,366	0,375	0,436	0,861	0,060	0,537	0,504	0,470	1,073	0,034
1.645,848	0,156	0,362	0,320	0,377	0,847	0,058	0,536	0,442	0,431	1,025	0,011
1.650,787	0,156	0,089	0,409	2,463	0,166	2,054	0,414	0,355	0,433	0,820	0,078
1.651,776	0,156	0,098	0,406	2,217	0,183	1,811	0,410	0,286	0,378	0,756	0,092
1.652,907	0,156	0,087	0,483	1,637	0,295	1,153	0,416	0,430	0,485	0,887	0,055
1.653,898	0,156	0,109	0,398	1,009	0,395	0,611	0,338	0,366	0,460	0,797	0,093
1.654,888	0,156	0,169	0,407	0,731	0,557	0,324	0,475	0,410	0,428	0,958	0,018
1.659,849	0,156	0,292	0,271	0,355	0,764	0,084	0,529	0,462	0,439	1,053	0,023
1.660,842	0,156	0,280	0,260	0,355	0,734	0,094	0,524	0,445	0,444	1,002	0,001
1.666,813	0,156	0,980	3,093	0,566	5,461	2,526	0,747	0,759	0,388	1,958	0,371
1.667,810	0,156	0,984	2,908	0,579	5,020	2,328	0,800	0,778	0,398	1,957	0,381
1.668,807	0,156	0,977	1,739	0,420	4,139	1,319	0,696	0,515	0,358	1,439	0,157
1.669,805	0,156	0,959	0,922	0,371	2,481	0,550	0,671	0,406	0,322	1,261	0,084
1.670,945	0,156	0,826	0,494	0,330	1,496	0,164	0,655	0,337	0,276	1,222	0,061
1.692,880	0,156	0,201	0,325	0,484	0,673	0,158	0,295	0,394	0,532	0,740	0,139
1.693,889	0,156	0,176	0,309	0,481	0,641	0,173	0,293	0,362	0,478	0,758	0,116
1.701,976	0,156	0,192	0,284	0,530	0,537	0,245	0,216	0,892	1,504	0,593	0,612
1.702,989	0,156	0,182	0,269	0,500	0,538	0,231	0,205	0,832	1,487	0,560	0,655
1.704,003	0,156	0,155	0,238	0,442	0,538	0,204	0,167	0,548	1,014	0,541	0,465
1.705,016	0,156	0,242	0,265	0,371	0,715	0,106	0,206	0,404	0,618	0,654	0,214
1.715,910	0,156	0,215	0,262	0,405	0,647	0,143	0,278	0,492	0,695	0,708	0,203
1.716,929	0,156	0,222	0,303	0,447	0,679	0,144	0,273	0,470	0,631	0,745	0,161
1.717,949	0,156	0,232	0,356	0,515	0,691	0,159	0,286	0,404	0,541	0,747	0,137
1.728,027	0,156	0,547	0,385	0,366	1,052	0,019	0,577	0,455	0,416	1,092	0,038
1.731,983	0,156	0,393	0,395	0,440	0,897	0,045	0,699	0,576	0,457	1,261	0,119
1.733,009	0,156	0,345	0,321	0,386	0,831	0,065	0,674	0,495	0,402	1,230	0,092

1.738,885	0,156	0,834	0,644	0,343	1,876	0,301	0,690	0,415	0,331	1,255	0,084
1.739,915	0,156	0,876	0,639	0,297	2,149	0,341	0,710	0,359	0,274	1,312	0,085
**1.740,945**	**0,156**	**0,852**	1,841	0,920	2,001	0,921	0,738	0,558	0,346	1,611	0,212
**1.741,976**	**0,156**	**0,853**	1,794	0,942	1,905	0,852	0,752	0,586	0,359	1,632	0,227
1.743,007	0,156	0,822	1,041	0,625	1,664	0,415	0,612	0,416	0,359	1,159	0,057
1.743,892	0,156	0,649	0,818	0,676	1,210	0,142	0,345	0,395	0,491	0,805	0,096
1.744,924	0,156	0,578	0,653	0,583	1,120	0,070	0,314	0,360	0,479	0,751	0,119
1.746,989	0,156	0,473	0,374	0,398	0,941	0,024	0,454	0,386	0,416	0,928	0,030
1.768,939	0,156	0,230	0,277	0,475	0,583	0,198	0,437	0,423	0,492	0,860	0,069
1.769,983	0,156	0,287	0,313	0,450	0,695	0,137	0,430	0,457	0,522	0,877	0,064
1.772,966	0,156	0,735	1,099	0,766	1,433	0,332	0,664	0,731	0,573	1,275	0,158
1.774,011	0,156	0,737	1,034	0,699	1,480	0,335	0,637	0,679	0,527	1,289	0,152
1.775,057	0,156	0,658	0,621	0,523	1,188	0,098	0,620	0,488	0,394	1,239	0,094
1.775,953	0,156	0,626	0,435	0,384	1,131	0,051	0,732	0,460	0,327	1,403	0,132
1.777,000	0,156	0,587	0,348	0,318	1,092	0,029	0,733	0,428	0,314	1,364	0,114
1.779,992	0,156	0,410	0,314	0,368	0,852	0,055	0,466	0,421	0,426	0,989	0,005
1.781,040	0,156	0,413	0,341	0,379	0,901	0,038	0,350	0,436	0,523	0,833	0,087
1.782,988	0,156	0,688	1,566	1,147	1,365	0,419	0,538	0,430	0,399	1,077	0,031
1.784,038	0,156	0,668	1,559	1,216	1,281	0,342	0,580	0,474	0,432	1,097	0,042
1.784,938	0,156	0,635	1,005	0,895	1,123	0,110	0,795	0,763	0,478	1,598	0,286
1.785,988	0,156	0,447	0,617	0,668	0,924	0,051	0,736	0,709	0,509	1,393	0,200
1.787,039	0,156	0,311	0,379	0,467	0,811	0,088	0,692	0,529	0,424	1,248	0,105
1.790,947	0,156	0,793	5,047	3,005	1,680	2,042	0,860	3,376	1,211	2,787	2,165
1.792,000	0,156	0,808	5,368	3,042	1,765	2,326	0,864	3,428	1,365	2,510	2,062
1.793,053	0,156	0,785	2,969	1,718	1,728	1,251	0,840	2,121	0,898	2,361	1,223
1.793,957	0,156	0,758	1,246	0,925	1,348	0,321	0,813	0,951	0,498	1,911	0,453
1.795,011	0,156	0,795	0,829	0,593	1,398	0,236	0,756	0,491	0,333	1,475	0,158
1.796,066	0,156	0,750	0,587	0,438	1,341	0,149	0,641	0,335	0,290	1,154	0,045
1.834,045	0,156	0,681	0,530	0,435	1,219	0,095	0,518	0,347	0,357	0,973	0,010
1.834,964	0,156	0,774	0,610	0,409	1,492	0,201	0,590	0,378	0,345	1,094	0,032

1.836,037	0,156	0,763	0,483	0,343	1,407	0,140	0,590	0,313	0,293	1,069	0,020
1.846,170	0,156	0,285	0,483	0,675	0,715	0,193	0,460	0,423	0,450	0,940	0,027
1.847,094	0,156	0,320	0,522	0,726	0,719	0,204	0,558	0,404	0,403	1,004	0,002
1.848,017	0,156	0,341	0,375	0,480	0,781	0,105	0,560	0,318	0,311	1,024	0,007
1.855,110	0,156	0,099	0,248	0,772	0,321	0,524	0,534	0,295	0,308	0,957	0,013
1.856,036	0,156	0,080	0,214	0,750	0,285	0,537	0,514	0,254	0,264	0,960	0,011
1.857,118	0,156	0,114	0,203	0,541	0,374	0,339	0,492	0,222	0,244	0,912	0,022
1.872,152	0,156	0,204	0,308	0,531	0,581	0,223	0,578	0,282	0,263	1,072	0,019
1.873,084	0,156	0,203	0,304	0,488	0,624	0,183	0,591	0,285	0,262	1,088	0,023
1.875,107	0,156	0,317	0,235	0,304	0,774	0,069	0,615	0,352	0,294	1,200	0,059
1.876,041	0,156	0,386	0,232	0,279	0,831	0,047	0,688	0,347	0,267	1,300	0,080
1.887,118	0,156	0,881	0,801	0,288	2,780	0,513	0,621	0,306	0,251	1,221	0,055
1.888,057	0,156	0,932	0,997	0,347	2,868	0,649	0,683	0,428	0,314	1,366	0,115
1.889,152	0,156	0,846	0,691	0,326	2,120	0,365	0,634	0,464	0,369	1,257	0,095
1.890,091	0,156	0,761	0,482	0,318	1,518	0,165	0,568	0,362	0,330	1,098	0,032
1.906,265	0,156	0,425	0,193	0,234	0,826	0,041	0,471	0,414	0,423	0,979	0,009
1.907,210	0,156	0,431	0,226	0,259	0,873	0,033	0,396	0,391	0,470	0,833	0,078
1.908,156	0,156	0,385	0,211	0,253	0,834	0,042	0,381	0,293	0,353	0,828	0,061
1.911,152	0,156	0,300	0,276	0,382	0,722	0,106	0,364	0,422	0,546	0,772	0,124
1.912,100	0,156	0,290	0,287	0,416	0,689	0,129	0,322	0,410	0,577	0,711	0,167
1.913,047	0,156	0,334	0,312	0,437	0,713	0,125	0,276	0,428	0,669	0,639	0,242
1.914,153	0,156	0,362	0,252	0,331	0,763	0,078	0,250	0,393	0,632	0,622	0,239
1.928,251	0,156	0,500	0,241	0,231	1,043	0,010	0,705	0,467	0,259	1,802	0,208
1.929,205	0,156	0,502	0,293	0,276	1,063	0,017	0,730	0,582	0,307	1,898	0,275
1.930,158	0,156	0,473	0,238	0,244	0,978	0,005	0,726	0,405	0,245	1,652	0,160
1.954,268	0,156	0,413	0,215	0,248	0,867	0,033	0,449	0,347	0,378	0,918	0,031
1.955,391	0,156	0,543	0,256	0,231	1,105	0,024	0,407	0,444	0,579	0,767	0,135
1.956,193	0,156	0,533	1,052	1,012	1,040	0,041	0,392	0,465	0,587	0,792	0,122
1.957,156	0,156	0,554	1,220	1,113	1,096	0,107	0,428	0,437	0,482	0,906	0,045
1.958,281	0,156	0,572	0,831	0,725	1,147	0,107	0,497	0,379	0,371	1,020	0,007

1.959,244	0,156	0,525	0,451	0,441	1,023	0,010	0,498	0,275	0,277	0,991	0,002
1.970,193	0,156	0,466	0,279	0,293	0,953	0,014	0,510	0,302	0,293	1,031	0,009
1.974,229	0,156	0,404	0,378	0,481	0,787	0,102	0,523	0,219	0,208	1,052	0,011
1.975,360	0,156	0,429	0,394	0,472	0,835	0,078	0,492	0,254	0,260	0,976	0,006
2.019,181	0,156	0,524	0,345	0,354	0,972	0,010	0,564	0,243	0,216	1,126	0,027
2.028,390	0,156	0,423	0,221	0,258	0,855	0,037	0,320	0,323	0,455	0,710	0,132
2.043,252	0,156	0,599	0,654	0,372	1,759	0,282	0,516	0,227	0,210	1,080	0,017
2.044,246	0,156	0,686	0,764	0,381	2,003	0,383	0,714	0,301	0,186	1,621	0,115
2.045,405	0,156	0,792	0,506	0,301	1,681	0,205	0,733	0,320	0,181	1,773	0,140
2.078,398	0,156	0,541	0,349	0,369	0,945	0,020	0,409	0,222	0,259	0,855	0,038
2.079,404	0,156	0,529	0,397	0,413	0,960	0,016	0,472	0,223	0,244	0,914	0,021
2.080,409	0,156	0,519	0,309	0,335	0,924	0,025	0,452	0,200	0,224	0,894	0,024
2.191,582	0,156	0,438	0,217	0,269	0,804	0,053	0,440	0,292	0,358	0,814	0,067
2.225,314	0,156	0,953	0,464	0,130	3,578	0,335	0,770	0,243	0,128	1,897	0,115
2.225,841	0,156	0,968	0,520	0,115	4,508	0,405	0,742	0,261	0,152	1,717	0,109
2.232,701	0,156	0,497	0,177	0,171	1,035	0,006	0,698	0,401	0,212	1,886	0,188
2.246,112	0,156	0,363	0,204	0,583	0,349	0,380	0,593	0,200	0,183	1,095	0,017
2.246,642	0,156	0,236	0,198	0,589	0,336	0,391	0,583	0,205	0,183	1,117	0,021
2.247,173	0,156	0,193	0,166	0,789	0,211	0,623	0,512	0,160	0,194	0,828	0,033
2.247,527	0,156	0,120	0,166	0,731	0,227	0,565	0,480	0,171	0,190	0,901	0,019
2.248,234	0,156	0,139	0,153	0,673	0,227	0,520	0,498	0,170	0,196	0,866	0,026
2.248,588	0,156	0,118	0,143	0,593	0,241	0,450	0,437	0,163	0,196	0,835	0,032
2.249,119	0,156	0,148	0,127	0,454	0,279	0,327	0,512	0,162	0,168	0,965	0,006
2.278,092	0,156	0,745	0,459	0,157	2,928	0,302	0,704	0,218	0,118	1,840	0,099
2.279,163	0,156	0,902	0,603	0,151	3,997	0,452	0,764	0,248	0,113	2,193	0,135
2.279,699	0,156	0,897	0,625	0,144	4,349	0,481	0,801	0,304	0,102	2,971	0,201
2.280,771	0,156	0,880	0,478	0,141	3,400	0,337	0,764	0,263	0,124	2,127	0,139
2.311,281	0,156	0,651	0,549	0,324	1,692	0,224	0,647	0,278	0,182	1,531	0,097
2.312,363	0,156	0,691	0,655	0,368	1,779	0,287	0,697	0,292	0,166	1,763	0,126
2.313,084	0,156	0,792	0,526	0,286	1,839	0,240	0,726	0,298	0,173	1,719	0,125

2.313,986	0,156	0,717	0,412	0,241	1,705	0,170	0,712	0,276	0,170	1,622	0,106
2.327,001	0,156	0,753	0,393	0,198	1,985	0,195	0,714	0,302	0,168	1,804	0,135
2.355,186	0,156	0,569	0,329	0,334	0,985	0,005	0,541	0,241	0,230	1,049	0,011
2.356,099	0,156	0,461	0,403	0,471	0,857	0,067	0,520	0,235	0,233	1,010	0,002
2.357,195	0,156	0,428	0,400	0,521	0,768	0,121	0,552	0,239	0,228	1,049	0,011
2.357,926	0,156	0,461	0,336	0,422	0,798	0,085	0,528	0,208	0,193	1,076	0,015
2.396,321	0,156	0,694	0,339	0,134	2,520	0,204	0,636	0,169	0,118	1,426	0,050
2.397,244	0,156	0,908	0,558	0,169	3,293	0,389	0,714	0,222	0,130	1,706	0,092
2.398,168	0,156	0,903	0,635	0,188	3,379	0,447	0,728	0,257	0,144	1,790	0,114
2.399,092	0,156	0,831	0,442	0,161	2,755	0,282	0,684	0,213	0,136	1,568	0,077
2.417,067	0,156	0,502	0,169	0,164	1,031	0,005	0,383	0,261	0,406	0,642	0,145
2.434,010	0,156	0,487	0,215	0,244	0,881	0,029	0,404	0,380	0,464	0,819	0,084
2.435,130	0,156	0,586	0,238	0,213	1,116	0,025	0,402	0,339	0,414	0,820	0,074
2.501,241	0,156	0,545	0,209	0,203	1,028	0,006	0,278	0,288	0,461	0,625	0,173
2.502,381	0,156	0,511	0,159	0,175	0,909	0,016	0,149	0,232	0,587	0,395	0,355
2.503,332	0,156	0,490	0,162	0,183	0,885	0,021	0,165	0,223	0,569	0,392	0,346
2.504,283	0,156	0,493	0,173	0,182	0,950	0,009	0,253	0,229	0,422	0,541	0,194
2.522,200	0,156	0,425	0,325	0,377	0,864	0,051	0,625	0,241	0,189	1,270	0,051
2.523,347	0,156	0,412	0,297	0,353	0,843	0,056	0,523	0,185	0,181	1,023	0,004
2.578,394	0,156	0,572	0,391	0,295	1,324	0,096	0,652	0,501	0,377	1,328	0,124
2.579,557	0,156	0,637	0,620	0,468	1,323	0,151	0,667	0,547	0,393	1,394	0,155
2.580,527	0,156	0,679	0,675	0,490	1,378	0,185	0,667	0,490	0,350	1,400	0,140
2.581,497	0,156	0,721	0,495	0,351	1,412	0,144	0,680	0,359	0,259	1,385	0,100
2.614,611	0,156	0,618	0,157	0,122	1,291	0,035	0,646	0,326	0,202	1,610	0,123
2.615,589	0,156	0,549	0,136	0,120	1,135	0,016	0,617	0,300	0,198	1,512	0,102
2.744,561	0,156	0,565	0,510	0,387	1,319	0,123	0,626	0,262	0,203	1,295	0,060
2.745,772	0,156	0,624	0,667	0,505	1,322	0,162	0,644	0,334	0,256	1,305	0,078
2.746,781	0,156	0,606	0,585	0,471	1,241	0,114	0,615	0,316	0,262	1,210	0,055
2.747,791	0,156	0,585	0,362	0,314	1,155	0,049	0,578	0,225	0,196	1,149	0,029
2.764,586	0,156	0,517	0,229	0,212	1,082	0,017	0,592	0,157	0,127	1,238	0,030

2.765,802	0,156	0,536	0,238	0,147	1,619	0,091	0,652	0,158	0,095	1,662	0,063
2.872,334	0,156	0,696	0,320	0,202	1,587	0,118	0,657	0,177	0,129	1,374	0,048
2.872,958	0,156	0,672	0,337	0,208	1,618	0,129	0,658	0,144	0,094	1,543	0,051
2.959,358	0,156	0,668	0,422	0,245	1,726	0,178	0,677	0,208	0,139	1,495	0,069
2.960,416	0,156	0,663	0,381	0,220	1,735	0,162	0,637	0,183	0,135	1,360	0,048

Supplementary table 2. Identified discriminative peaks in **primary trauma** muscle tissue between with and control

Centroid [m/z]	± [Da]	ROC [AUC] TM MSC-TX vs TM-controll	Max. intensity TM-MSC-TX	Max. intensity TM-control	Ratio intensity TM MSC-TX vs TM-control	Δ[intensity]	p-Value - WKS	Significance rating - WKS	IMS [Mr]	LC-MS/MS [Mr]	Δ[Da]	Protein
1.151,591	0,156	0,059	0,362	0,851	0,425	0,490	0,000	<0,001	1.150,584	1150,54155	0,042	Fibronectin OS=Rattus norvegicus GN=Fn1 PE=4 SV=2
1.152,615	0,156	0,078	0,351	0,672	0,521	0,322	0,000	<0,001	1.151,608	1151,66333	-0,056	Protein RGD1562402 OS=Rattus norvegicus GN=RGD1562402 PE=3 SV=1
1.078,603	0,156	0,116	0,484	0,795	0,608	0,311	0,000	<0,001	1.077,596	1077,54451	0,051	Protein LOC100910721 OS=Rattus norvegicus GN=LOC100910721 PE=3 SV=1
2.502,381	0,156	0,149	0,232	0,587	0,395	0,355	0,000	<0,001	2.501,374			
1.362,683	0,156	0,160	0,668	1,278	0,522	0,610	0,000	<0,001	1.361,676	1361,70262	-0,027	Protein Naca OS=Rattus norvegicus

												GN=Naca PE=4 SV=1
1.325,702	0,156	0,164	0,643	1,148	0,560	0,505	0,000	<0,001	1.324,695	1324,74326	-0,049	Histone H4 OS=Rattus norvegicus GN=Hist1h4b PE=1 SV=2
2.503,332	0,156	0,165	0,223	0,569	0,392	0,346	0,000	<0,001	2.502,325			
1.704,003	0,156	0,167	0,548	1,014	0,541	0,465	0,000	<0,001	1.702,996			
1.361,720	0,156	0,168	0,605	1,278	0,473	0,673	0,000	<0,001	1.360,713	1360,68638	0,026	60S ribosomal protein L13 OS=Rattus norvegicus GN=Rpl13 PE=1 SV=2
1.326,759	0,156	0,170	0,550	0,910	0,605	0,360	0,000	<0,001	1.325,752	1325,75335	-0,002	Histone H4 OS=Rattus norvegicus GN=Hist1h4b PE=1 SV=2
979,579	0,156	0,172	0,432	0,963	0,449	0,530	0,000	<0,001	978,572	978,492975	0,079	Protein Col6a3 OS=Rattus norvegicus GN=Col6a3 PE=4 SV=2
1.341,642	0,156	0,176	0,488	0,773	0,632	0,285	0,000	<0,001	1.340,635	1340,65209	-0,017	60S ribosomal protein L26 OS=Rattus norvegicus

												GN=Rpl26 PE=3 SV=1
1.573,834	0,156	0,195	0,486	0,856	0,567	0,371	0,000	<0,001	1.572,827	1572,79515	0,032	40S ribosomal protein S3 OS=Rattus norvegicus GN=Rps3 PE=1 SV=1
1.702,989	0,156	0,205	0,832	1,487	0,560	0,655	0,000	<0,001	1.701,982	1701,88609	0,096	60S acidic ribosomal protein P1 OS=Rattus norvegicus GN=Rplp1 PE=3 SV=1
1.444,713	0,156	0,209	0,786	1,325	0,593	0,539	0,000	<0,001	1.443,706	1443,6982	0,007	Vimentin OS=Rattus norvegicus GN=Vim PE=1 SV=2
1.329,700	0,156	0,210	0,702	1,115	0,629	0,413	0,000	<0,001	1.328,693			
1.324,762	0,156	0,215	0,592	0,923	0,642	0,330	0,000	<0,001	1.323,755			
1.701,976	0,156	0,216	0,892	1,504	0,593	0,612	0,000	<0,001	1.700,969	1700,85704	0,112	Protein Col6a1 OS=Rattus norvegicus GN=Col6a1 PE=4 SV=1
1.007,609	0,156	0,218	0,570	0,861	0,661	0,292	0,000	<0,001	1.006,602	1006,58941	0,012	Creatine kinase M-type OS=Rattus norvegicus GN=Ckm PE=1 SV=2

1.572,746	0,156	0,220	0,535	0,971	0,551	0,436	0,000	<0,001	1.571,739	1571,72774	0,011	Hemoglobin alpha, adult chain 2 OS=Rattus norvegicus GN=Hba1 PE=2 SV=1
1.445,727	0,156	0,223	0,726	1,092	0,664	0,367	0,000	<0,001	1.444,720			
1.328,641	0,156	0,232	0,741	1,110	0,668	0,369	0,000	<0,001	1.327,634	1327,70732	-0,074	Protein Col6a3 OS=Rattus norvegicus GN=Col6a3 PE=4 SV=2
1.044,599	0,156	0,239	0,531	0,828	0,642	0,297	0,000	<0,001	1.043,592	1042,60994	0,982	40S ribosomal protein S3 OS=Rattus norvegicus GN=Rps3 PE=1 SV=1
984,609	0,156	0,243	0,514	0,976	0,527	0,462	0,000	<0,001	983,602			
1.323,707	0,156	0,252	0,821	1,169	0,703	0,348	0,000	<0,001	1.322,700	1322,70257	-0,003	Protein Col6a1 OS=Rattus norvegicus GN=Col6a1 PE=4 SV=1
944,564	0,156	0,259	1,761	2,621	0,672	0,860	0,000	<0,001	943,557	943,521983	0,035	Histone H2A OS=Rattus norvegicus GN=LOC680322 PE=3 SV=1
945,536	0,156	0,275	1,079	1,536	0,702	0,457	0,000	<0,001	944,529			
985,550	0,156	0,303	0,529	0,822	0,644	0,292	0,000	<0,001	984,543			

1.363,767	0,156	0,325	1,385	1,749	0,792	0,363	0,000	<0,001	1.362,760			
1.254,716	0,156	0,340	1,086	1,380	0,787	0,294	0,000	<0,001	1.253,709	1253,63258	0,076	60S ribosomal protein L34 OS=Rattus norvegicus GN=Rpl34 PE=2 SV=1
1.487,816	0,156	0,672	0,770	0,468	1,645	0,302	0,000	<0,001	1.486,809	1486,69307	0,116	Heat shock cognate 71 kDa protein OS=Rattus norvegicus GN=Hspa8 PE=1 SV=1
1.488,856	0,156	0,698	0,756	0,422	1,792	0,334	0,000	<0,001	1.487,849	1487,73013	0,119	Tropomyosin alpha-1 chain OS=Rattus norvegicus GN=Tpm1 PE=1 SV=3
977,457	0,156	0,705	1,390	1,002	1,388	0,388	0,000	<0,001	976,450			
976,441	0,156	0,734	2,429	1,671	1,454	0,759	0,000	<0,001	975,434	975,43825	-0,005	Actin, alpha skeletal muscle OS=Rattus norvegicus GN=Acta1 PE=1 SV=1
1.666,813	0,156	0,747	0,759	0,388	1,958	0,371	0,000	<0,001	1.665,806	1665,96536	-0,160	RCG45615, isoform CRA_a OS=Rattus norvegicus GN=Rpl12 PE=2 SV=1
1.184,529	0,156	0,785	1,017	0,609	1,672	0,409	0,000	<0,001	1.183,522			

1.784,938	0,156	0,795	0,763	0,478	1,598	0,286	0,000	<0,001	1.783,931	1783,85584	0,075	Serum albumin OS=Rattus norvegicus GN=Alb PE=1 SV=2
1.667,810	0,156	0,800	0,778	0,398	1,957	0,381	0,000	<0,001	1.666,803			
1.793,957	0,156	0,813	0,951	0,498	1,911	0,453	0,000	<0,001	1.792,950			
1.200,661	0,156	0,822	1,881	1,000	1,882	0,881	0,000	<0,001	1.199,654	1200,65004	-0,996	Myosin-6 OS=Rattus norvegicus GN=Myh6 PE=4 SV=1
1.793,053	0,156	0,840	2,121	0,898	2,361	1,223	0,000	<0,001	1.792,046			
1.199,699	0,156	0,857	4,966	2,159	2,300	2,807	0,000	<0,001	1.198,692	1198,66534	0,026	Heat shock cognate 71 kDa protein OS=Rattus norvegicus GN=Hspa8 PE=1 SV=1
1.790,947	0,156	0,860	3,376	1,211	2,787	2,165	0,000	<0,001	1.789,940	1789,88605	0,054	Protein Actbl2 OS=Rattus norvegicus GN=Actbl2 PE=3 SV=1
1.792,000	0,156	0,864	3,428	1,365	2,510	2,062	0,000	<0,001	1.790,993			
1.198,632	0,156	0,869	6,823	2,752	2,479	4,071	0,000	<0,001	1.197,625	1197,65841	-0,034	Histone H1.4 OS=Rat. nor. GN=Hist1h1e PE=1 SV=3

Supplementary table 3 Identified discriminative peaks in **trauma adjacent** muscle tissue between with and control

Centroid [m/z]	± [Da]	ROC [AUC] TAM MSC-TX vs TAM-controll	Max. intensity TAM-MSC-TX	Max. intensity TM-control	Ratio intensity TAM MSC-TX vs TAM-control	Δ[intensity]	p-Value - WKS	Significance rating - WKS	IMS [Mr]	LC-MS/MS [Mr]	Δ[Da]	Protein
1.432,720	0,156	0,054	0,285	0,970	0,293	0,685	0,000	<0,001				
1.431,714	0,156	0,057	0,374	1,483	0,252	1,109	0,000	<0,001	1.430,707	1430,7395	0,033	Fibronectin OS=Rattus norvegicus GN=Fn1 PE=4 SV=2
1.078,603	0,156	0,068	0,355	0,696	0,510	0,341	0,000	<0,001	1.077,596	1077,5091	0,087	Myosin regulatory light chain 2, ventricular/cardiac muscle isoform OS=Rattus norvegicus GN=Myl2 PE=1 SV=2
1.433,727	0,156	0,069	0,288	0,609	0,472	0,322	0,000	<0,001				
1.171,531	0,156	0,069	0,354	1,080	0,328	0,726	0,000	<0,001	1.170,524	1170,5606	0,037	Actin, alpha skeletal muscle OS=Rattus norvegicus GN=Acta1 PE=1 SV=1
1.856,036	0,156	0,080	0,214	0,750	0,285	0,537	0,000	<0,001	1.855,029	1855,8059	0,777	Y-box-binding protein 3 OS=Rattus norvegicus GN=Ybx3 PE=2 SV=1
1.430,709	0,156	0,082	0,385	1,705	0,226	1,319	0,000	<0,001	1.429,702	1430,6979	0,996	Synaptic vesicle membrane protein VAT-

												1 homolog OS=Rattus norvegicus GN=Vat1 PE=1 SV=1
1.652,907	0,156	0,087	0,483	1,637	0,295	1,153	0,000	<0,001	1.651,900	1651,7572	0,142	Protein disulfide-isomerase A3 OS=Rattus norvegicus GN=Pdia3 PE=1 SV=2
1.325,702	0,156	0,088	0,444	1,105	0,402	0,660	0,000	<0,001				
1.650,787	0,156	0,089	0,409	2,463	0,166	2,054	0,000	<0,001				
928,481	0,156	0,090	0,280	0,648	0,432	0,368	0,000	<0,001	927,474	926,50635	0,967	Periostin, osteoblast specific factor (Predicted), isoform CRA_a OS=Rattus norvegicus GN=Postn PE=4 SV=1
1.326,759	0,156	0,090	0,420	0,935	0,449	0,516	0,000	<0,001	1.325,752	1325,7533	0,002	Histone H1.4 OS=Rattus norvegicus GN=Hist1h1e PE=1 SV=3
1.463,823	0,156	0,095	0,333	0,738	0,451	0,405	0,000	<0,001	1.462,816	1462,7395	0,076	Protein Tln1 OS=Rattus norvegicus GN=Tln1 PE=4 SV=1
1.172,574	0,156	0,097	0,464	1,063	0,436	0,599	0,000	<0,001	1.171,567	1171,6433	0,077	Carbonic anhydrase 3 OS=Rattus norvegicus GN=Ca3 PE=1 SV=3
1.651,776	0,156	0,098	0,406	2,217	0,183	1,811	0,000	<0,001	1.650,769	1651,7395	0,971	Protein disulfide-isomerase A3 OS=Rattus norvegicus GN=Pdia3 PE=1 SV=2
1.855,110	0,156	0,099	0,248	0,772	0,321	0,524	0,000	<0,001				

1.462,798	0,156	0,101	0,376	0,885	0,425	0,509	0,000	<0,001				
1.653,898	0,156	0,109	0,398	1,009	0,395	0,611	0,000	<0,001				
1.390,746	0,156	0,111	0,352	0,690	0,510	0,338	0,000	<0,001	1.389,739	1389,7155	0,023	Carbonic anhydrase 3 OS=Rattus norvegicus GN=Ca3 PE=1 SV=3
1.857,118	0,156	0,114	0,203	0,541	0,374	0,339	0,000	<0,001				
1.233,583	0,156	0,115	0,436	0,780	0,559	0,344	0,000	<0,001				
2.248,588	0,156	0,118	0,143	0,593	0,241	0,450	0,000	<0,001				
1.173,617	0,156	0,119	0,428	0,810	0,529	0,382	0,000	<0,001				
2.247,527	0,156	0,120	0,166	0,731	0,227	0,565	0,000	<0,001				
1.328,641	0,156	0,125	0,434	0,809	0,537	0,375	0,000	<0,001	1.327,634	1327,6505	0,017	Procollagen, type VI, alpha 2, isoform CRA_a OS=Rattus norvegicus GN=Col6a2 PE=4 SV=2
1.444,713	0,156	0,133	0,381	0,729	0,523	0,347	0,000	<0,001	1.443,706	1443,703	0,003	Elongation factor 2 OS=Rattus norvegicus GN=Eef2 PE=1 SV=4
943,512	0,156	0,137	0,414	0,808	0,513	0,393	0,000	<0,001	942,505	942,49642	0,008	60S ribosomal protein L18a OS=Rattus norvegicus GN=Rpl18a PE=2 SV=1
1.362,683	0,156	0,138	0,481	0,879	0,547	0,398	0,000	<0,001	1.361,676	1361,7026	0,027	Protein Naca OS=Rattus norvegicus GN=Naca PE=4 SV=1

2.248,234	0,156	0,139	0,153	0,673	0,227	0,520	0,000	<0,001				
971,549	0,156	0,139	0,333	0,663	0,502	0,330	0,000	<0,001				
2.249,119	0,156	0,148	0,127	0,454	0,279	0,327	0,000	<0,001				
1.135,631	0,156	0,158	0,314	0,724	0,434	0,410	0,000	<0,001	1.134,624	1135,6199	0,996	Myosin-3 OS=Rattus norvegicus GN=Myh3 PE=4 SV=1
944,564	0,156	0,160	0,989	2,101	0,470	1,113	0,000	<0,001	943,557	943,52198	0,035	Histone H2A OS=Rattus norvegicus GN=LOC680322 PE=3 SV=1
1.361,720	0,156	0,169	0,493	0,853	0,578	0,360	0,000	<0,001				
1.654,888	0,156	0,169	0,407	0,731	0,557	0,324	0,000	<0,001	1.653,881	1653,9188	0,038	Uncharacterized protein OS=Rattus norvegicus GN=RGD1560414 PE=4 SV=1
1.114,550	0,156	0,172	0,720	1,356	0,531	0,636	0,000	<0,001	1.113,543	1113,5741	0,031	Protein Rrbp1 OS=Rattus norvegicus GN=Rrbp1 PE=4 SV=2
1.055,516	0,156	0,177	0,493	0,849	0,581	0,356	0,000	<0,001	1.054,509	1053,5518	0,957	40S ribosomal protein S14 OS=Rattus norvegicus GN=Rps14 PE=2 SV=1
1.348,663	0,156	0,178	0,390	0,728	0,536	0,338	0,000	<0,001	1.347,656	1346,726	0,930	40S ribosomal protein S8 OS=Rattus norvegicus GN=Rps8 PE=2 SV=1
945,536	0,156	0,178	0,744	1,403	0,530	0,659	0,000	<0,001				
1.088,576	0,156	0,186	0,462	0,784	0,590	0,321	0,000	<0,001				

2.247,173	0,156	0,193	0,166	0,789	0,211	0,623	0,000	<0,001				
1.033,543	0,156	0,204	0,704	1,035	0,681	0,330	0,000	<0,001	1.032,536	1032,4957	0,040	Sarcoplasmic/endoplasmic reticulum calcium ATPase 1 OS=Rattus norvegicus GN=Atp2a1 PE=3 SV=1
1.032,635	0,156	0,213	0,977	1,423	0,686	0,446	0,000	<0,001	1.031,628	1031,5837	0,044	Histone H3 OS=Rattus norvegicus GN=H3f3b PE=2 SV=1
2.246,642	0,156	0,236	0,198	0,589	0,336	0,391	0,000	<0,001				
1.458,834	0,156	0,278	0,716	1,044	0,685	0,328	0,000	<0,001	1.457,827	1458,6939	0,867	Heterogeneous nuclear ribonucleoprotein M OS=Rattus norvegicus GN=Hnrnpm PE=2 SV=1
1.572,746	0,156	0,302	0,717	1,078	0,665	0,361	0,000	<0,001	1.571,739	1571,7277	0,011	Hemoglobin alpha, adult chain 2 OS=Rattus norvegicus GN=Hba1 PE=2 SV=1
1.201,730	0,156	0,654	1,419	1,082	1,311	0,337	0,000	<0,001				
1.397,748	0,156	0,660	1,406	1,053	1,336	0,353	0,000	<0,001	1.396,741	1396,6964	0,044	Protein disulfide-isomerase A3 OS=Rattus norvegicus GN=Pdia3 PE=1 SV=2
1.784,038	0,156	0,668	1,559	1,216	1,281	0,342	0,000	<0,001	1.783,031	1783,8319	0,801	Calreticulin OS=Rattus norvegicus GN=Calr PE=1 SV=1
2.044,246	0,156	0,686	0,764	0,381	2,003	0,383	0,000	<0,001				

1.782,988	0,156	0,688	1,566	1,147	1,365	0,419	0,000	<0,001	1.781,981	1781,9404	0,040	Myosin light chain 3 OS=Rattus norvegicus GN=Myl3 PE=2 SV=2
1.292,733	0,156	0,696	1,755	1,133	1,549	0,622	0,000	<0,001				
1.396,764	0,156	0,708	2,056	1,322	1,556	0,735	0,000	<0,001	1.395,757	1395,7462	0,011	Myosin light chain 3 OS=Rattus norvegicus GN=Myl3 PE=2 SV=2
937,556	0,156	0,712	1,920	0,946	2,031	0,975	0,000	<0,001	936,549	936,5006	0,048	Protein Flnc OS=Rattus norvegicus GN=Flnc PE=4 SV=1
936,595	0,156	0,713	3,082	1,594	1,934	1,488	0,000	<0,001	935,588	935,57761	0,010	Acyl-CoA dehydrogenase family member 11 OS=Rattus norvegicus GN=Acad11 PE=1 SV=1
1.291,700	0,156	0,718	2,521	1,528	1,650	0,993	0,000	<0,001				
1.185,583	0,156	0,723	1,485	0,975	1,522	0,509	0,000	<0,001	1.184,576	1187,6364	3,061	60S ribosomal protein L10 OS=Rattus norvegicus GN=Rpl10 PE=1 SV=3
1.489,896	0,156	0,728	1,740	1,118	1,556	0,621	0,000	<0,001	1.488,889	1488,7723	0,116	Protein Flnc OS=Rattus norvegicus GN=Flnc PE=4 SV=1
1.772,966	0,156	0,735	1,099	0,766	1,433	0,332	0,000	<0,001	1.771,959	1772,8912	0,932	Protein Myom3 OS=Rattus norvegicus GN=Myom3 PE=4 SV=2
1.774,011	0,156	0,737	1,034	0,699	1,480	0,335	0,000	<0,001				

1.200,661	0,156	0,753	4,082	2,556	1,597	1,526	0,000	<0,001	1.199,654	1200,65	0,996	Myosin-6 OS=Rattus norvegicus GN=Myh6 PE=4 SV=1
1.793,957	0,156	0,758	1,246	0,925	1,348	0,321	0,000	<0,001				
1.184,529	0,156	0,764	2,072	1,284	1,614	0,788	0,000	<0,001				
1.505,830	0,156	0,770	1,904	1,069	1,782	0,836	0,000	<0,001				
1.199,699	0,156	0,773	11,717	6,739	1,739	4,978	0,000	<0,001	1.198,692	1198,6653	0,026	Heat shock cognate 71 kDa protein OS=Rattus norvegicus GN=Hspa8 PE=1 SV=1
1.199,699	0,156	0,773	11,717	6,739	1,739	4,978	0,000	<0,001				
1.474,747	0,156	0,774	0,894	0,546	1,636	0,347	0,000	<0,001	1.473,740	1473,7502	0,011	60S ribosomal protein L13 OS=Rattus norvegicus GN=RGD1563145 PE=3 SV=1
1.793,053	0,156	0,785	2,969	1,718	1,728	1,251	0,000	<0,001				
1.198,632	0,156	0,786	17,398	9,604	1,812	7,794	0,000	<0,001	1.197,625	1197,5118	0,113	Actin, alpha skeletal muscle OS=Rattus norvegicus GN=Acta1 PE=1 SV=1
1.790,947	0,156	0,793	5,047	3,005	1,680	2,042	0,000	<0,001	1.789,940	1789,8861	0,054	Actin, alpha skeletal muscle OS=Rattus norvegicus GN=Acta1 PE=1 SV=1
1.473,845	0,156	0,801	1,115	0,578	1,929	0,537	0,000	<0,001	1.472,838	1473,7502	0,913	60S ribosomal protein L13 OS=Rattus

												norvegicus GN=RGD1563145 PE=3 SV=1
1.792,000	0,156	0,808	5,368	3,042	1,765	2,326	0,000	<0,001				
1.504,781	0,156	0,810	1,912	0,928	2,059	0,983	0,000	<0,001				
1.487,816	0,156	0,815	3,193	1,483	2,153	1,709	0,000	<0,001	1.486,809	1486,6931	0,116	Heat shock cognate 71 kDa protein OS=Rattus norvegicus GN=Hspa8 PE=1 SV=1
1.743,007	0,156	0,822	1,041	0,625	1,664	0,415	0,000	<0,001				
1.488,856	0,156	0,822	3,431	1,428	2,402	2,003	0,000	<0,001	1.487,849	1487,7301	0,119	Tropomyosin alpha-1 chain OS=Rattus norvegicus GN=Tpm1 PE=1 SV=3
1.889,152	0,156	0,846	0,691	0,326	2,120	0,365	0,000	<0,001	1.888,145	1889,0826	0,938	Myosin light chain 1/3, skeletal muscle isoform OS=Rattus norvegicus GN=Myl1 PE=1 SV=2
1.740,945	0,156	0,852	1,841	0,920	2,001	0,921	0,000	<0,001	1.739,938	1739,8326	0,105	Protein Tnc OS= Rat.nor. GN=Tnc PE=4 SV=1
1.741,976	0,156	0,853	1,794	0,942	1,905	0,852	0,000	<0,001				
1.739,915	0,156	0,876	0,639	0,297	2,149	0,341	0,000	<0,001				
2.280,771	0,156	0,880	0,478	0,141	3,400	0,337	0,000	<0,001				
1.887,118	0,156	0,881	0,801	0,288	2,780	0,513	0,000	<0,001				

2.279,699	0,156	0,897	0,625	0,144	4,349	0,481	0,000	<0,001				
2.279,163	0,156	0,902	0,603	0,151	3,997	0,452	0,000	<0,001				
2.398,168	0,156	0,903	0,635	0,188	3,379	0,447	0,000	<0,001				
2.397,244	0,156	0,908	0,558	0,169	3,293	0,389	0,000	<0,001				
1.615,901	0,156	0,923	0,991	0,451	2,200	0,541	0,000	<0,001				
1.888,057	0,156	0,932	0,997	0,347	2,868	0,649	0,000	<0,001	1.887,050	1886,9906	0,059	Myl6 protein OS= Rat.nor. GN=Myl6 PE=2 SV=1
2.225,314	0,156	0,953	0,464	0,130	3,578	0,335	0,000	<0,001				
1.669,805	0,156	0,959	0,922	0,371	2,481	0,550	0,000	<0,001				
2.225,841	0,156	0,968	0,520	0,115	4,508	0,405	0,000	<0,001				
1.613,818	0,156	0,968	1,641	0,457	3,593	1,184	0,000	<0,001	1.612,811	1612,7687	0,042	Heterogeneous nuclear ribonucleoprotein M OS=Rat.nor. N=Hnrnpm PE=2 SV=1
1.614,790	0,156	0,970	1,563	0,430	3,630	1,132	0,000	<0,001				
1.668,807	0,156	0,977	1,739	0,420	4,139	1,319	0,000	<0,001	1.667,800	1667,8123	0,013	Protein Tnc OS= Rat.nor. GN=Tnc PE=4 SV=1
1.666,813	0,156	0,980	3,093	0,566	5,461	2,526	0,000	<0,001	1.665,806	1665,9654	0,160	RCG45615, isoform CRA_a OS= Rat.nor. GN=Rpl12 PE=2 SV=1
1.667,810	0,156	0,984	2,908	0,579	5,020	2,328	0,000	<0,001				

Supplementary table 4: Identified proteins by corresponding bottom up LC-MS/MS approach with adjacent tissue sections

1) Retention time 2) mass measured 3) Mass error

Time1) (min)	Charge	m/z	Mass2	Error3 (ppm)	Score	Sequence	Modifications	Accession	Description
6.24	2	309.17	616.33	-1.92	29	ENRAK		Q9ER30	Kelch-like protein 41 OS=Rattus norvegicus GN=Klhl41 PE=1 SV=1
8.86	2	309.68	617.34	-2.54	27	SKEALA		P02770	Serum albumin OS=Rattus norvegicus GN=Alb PE=1 SV=2
22.87	2	310.69	619.37	-2.45	30	FNVLK		Q6PDV9	Ribosomal protein S11 OS=Rattus norvegicus GN=Rps11 PE=2 SV=1
13.40	2	310.71	619.40	-3.01	28	FKVVK		P62268	40S ribosomal protein S23 OS=Rattus norvegicus GN=Rps23 PE=1 SV=3
12.40	2	317.19	632.37	-3.50	27	LVMVR	[3] Oxidation (M)	P16290	Phosphoglycerate mutase 2 OS=Rattus norvegicus GN=Pgam2 PE=2 SV=2
16.78	2	322.69	643.36	-2.61	46	LDLAGR		P60711	Actin. cytoplasmic 1 OS=Rattus norvegicus GN=Actb PE=1 SV=1
16.78	2	322.69	643.36	-2.61	46	LDLAGR		P68035	Actin. alpha cardiac muscle 1 OS=Rattus norvegicus GN=Actc1 PE=2 SV=1
16.78	2	322.69	643.36	-2.61	46	LDLAGR		P68136	Actin. alpha skeletal muscle OS=Rattus norvegicus GN=Acta1 PE=1 SV=1
16.78	2	322.69	643.36	-2.61	46	LDLAGR		D3ZRN3	Protein Actbl2 OS=Rattus norvegicus GN=Actbl2 PE=3 SV=1
29.86	2	322.72	643.43	-2.53	26	GILTLK		P68136	Actin. alpha skeletal muscle OS=Rattus norvegicus GN=Acta1 PE=1 SV=1
29.86	2	322.72	643.43	-2.53	26	GILTLK		P68035	Actin. alpha cardiac muscle 1 OS=Rattus norvegicus GN=Actc1 PE=2 SV=1
29.86	2	322.72	643.43	-2.53	26	GILTLK		P60711	Actin. cytoplasmic 1 OS=Rattus norvegicus GN=Actb PE=1 SV=1
29.86	2	322.72	643.43	-2.53	26	GILTLK		D3ZRN3	Protein Actbl2 OS=Rattus norvegicus GN=Actbl2 PE=3 SV=1
11.03	2	323.20	644.38	-2.90	28	LANLSK		P00564	Creatine kinase M-type OS=Rattus norvegicus GN=Ckm PE=1 SV=2

23.76	2	324.71	647.40	-2.60	25	LIFAGK		F1LML2	Polyubiquitin-C OS=Rattus norvegicus GN=Ubc PE=2 SV=1
20.31	2	332.69	663.37	-2.66	25	AFASLR		P41123	60S ribosomal protein L13 OS=Rattus norvegicus GN=Rpl13 PE=1 SV=2
11.39	2	333.68	665.35	-3.10	33	YGASLR		D4A7B1	60S ribosomal protein L37a OS=Rattus norvegicus GN=Rpl37a PE=3 SV=1
17.55	2	339.70	677.38	-2.30	47	SLMLAK	[3] Oxidation (M)	F1LMC6	Troponin I. slow skeletal muscle (Fragment) OS=Rattus norvegicus GN=Tnni1 PE=4 SV=1
11.03	2	341.69	681.36	-3.24	26	GMLPHK		Q5RK10	60S ribosomal protein L13a OS=Rattus norvegicus GN=Rpl13a PE=2 SV=1
25.84	2	343.71	685.41	-2.39	33	DGLIIR		P84100	60S ribosomal protein L19 OS=Rattus norvegicus GN=Rpl19 PE=1 SV=1
10.05	2	344.20	686.39	-2.68	27	VADALAK		B1H216	Hemoglobin alpha. adult chain 2 OS=Rattus norvegicus GN=Hba1 PE=2 SV=1
7.80	2	349.66	697.31	-2.94	27	MFDAAK	[1] Oxidation (M)	F1LMC6	Troponin I. slow skeletal muscle (Fragment) OS=Rattus norvegicus GN=Tnni1 PE=4 SV=1
18.25	2	350.23	698.44	-2.71	25	ALVAAVR		P02770	Serum albumin OS=Rattus norvegicus GN=Alb PE=1 SV=2
20.56	2	350.23	698.44	-2.62	44	VGLIAAR		P62919	60S ribosomal protein L8 OS=Rattus norvegicus GN=Rpl8 PE=2 SV=2
9.31	2	361.68	721.34	-2.26	32	AEFAER		P04692	Tropomyosin alpha-1 chain OS=Rattus norvegicus GN=Tpm1 PE=1 SV=3
9.31	2	361.68	721.34	-2.26	32	AEFAER		Q5FVG5	Similar to tropomyosin 1. embryonic fibroblast-rat. isoform CRA_c OS=Rattus norvegicus GN=Tpm2 PE=2 SV=1
9.31	2	361.68	721.34	-2.26	32	AEFAER		Q6AZ25	Tropomyosin 1. alpha OS=Rattus norvegicus GN=Tpm1 PE=2 SV=1
9.31	2	361.68	721.34	-2.26	32	AEFAER		Q63610	Tropomyosin alpha-3 chain OS=Rattus norvegicus GN=Tpm3 PE=1 SV=2
9.31	2	361.68	721.34	-2.26	32	AEFAER		P09495	Tropomyosin alpha-4 chain OS=Rattus norvegicus GN=Tpm4 PE=1 SV=3
23.28	2	363.70	725.38	-2.34	27	FAYLGR		Q5RK10	60S ribosomal protein L13a OS=Rattus norvegicus GN=Rpl13a PE=2 SV=1

11.39	2	365.23	728.45	-3.18	36	ALKEIR		D4ABZ9	Protein Rpl31-ps8 (Fragment) OS=Rattus norvegicus GN=Rpl31l4 PE=4 SV=2
24.70	2	365.73	729.44	-2.47	29	LSELLR		P34058	Heat shock protein HSP 90-beta OS=Rattus norvegicus GN=Hsp90ab1 PE=1 SV=4
24.70	2	365.73	729.44	-2.47	29	LSELLR		P82995	Heat shock protein HSP 90-alpha OS=Rattus norvegicus GN=Hsp90aa1 PE=1 SV=3
11.76	2	367.19	732.36	-2.62	35	SSMPGVR		P31000	Vimentin OS=Rattus norvegicus GN=Vim PE=1 SV=2
8.19	2	368.69	735.36	-2.42	40	VSMDLR	[3] Oxidation (M)	F1LMC6	Troponin I. slow skeletal muscle (Fragment) OS=Rattus norvegicus GN=Tnni1 PE=4 SV=1
8.78	2	369.18	736.35	-2.24	39	TAENFR		P10111	Peptidyl-prolyl cis-trans isomerase A OS=Rattus norvegicus GN=Ppia PE=1 SV=2
18.88	2	371.73	741.45	-2.38	41	AAQLAIR		P62912	60S ribosomal protein L32 OS=Rattus norvegicus GN=Rpl32 PE=1 SV=2
9.81	2	372.70	743.38	-2.54	45	TPEELR		Q66HH8	Annexin OS=Rattus norvegicus GN=Anxa5 PE=2 SV=1
13.02	2	372.73	743.45	-2.72	35	LATALQK		P04692	Tropomyosin alpha-1 chain OS=Rattus norvegicus GN=Tpm1 PE=1 SV=3
13.02	2	372.73	743.45	-2.72	35	LATALQK		Q5FVG5	Similar to tropomyosin 1. embryonic fibroblast-rat. isoform CRA_c OS=Rattus norvegicus GN=Tpm2 PE=2 SV=1
13.02	2	372.73	743.45	-2.72	35	LATALQK		Q6AZ25	Tropomyosin 1. alpha OS=Rattus norvegicus GN=Tpm1 PE=2 SV=1
13.02	2	372.73	743.45	-2.72	35	LATALQK		Q63610	Tropomyosin alpha-3 chain OS=Rattus norvegicus GN=Tpm3 PE=1 SV=2
13.02	2	372.73	743.45	-2.72	35	LATALQK		P09495	Tropomyosin alpha-4 chain OS=Rattus norvegicus GN=Tpm4 PE=1 SV=3
13.02	2	372.73	743.45	-2.72	35	LATALQK		M0R4A2	Uncharacterized protein (Fragment) OS=Rattus norvegicus PE=4 SV=1
18.41	2	373.23	744.45	-2.34	51	LASSVLR		P84100	60S ribosomal protein L19 OS=Rattus norvegicus GN=Rpl19 PE=1 SV=1
33.73	2	374.72	747.43	-2.46	25	ALELFR		Q9QZ76	Myoglobin OS=Rattus norvegicus GN=Mb PE=1 SV=3
26.21	2	375.21	748.41	-2.65	27	FDVQLK		D3ZLL8	Protein LOC100909878 OS=Rattus norvegicus GN=Rps15al4 PE=3 SV=1

21.04	2	375.22	748.42	-2.32	40	VAFSVAR		P21531	60S ribosomal protein L3 OS=Rattus norvegicus GN=Rpl3 PE=1 SV=3
8.65	2	376.21	750.40	-2.26	33	VYQVSR		Q6P725	Desmin OS=Rattus norvegicus GN=Des PE=2 SV=1
22.57	2	376.22	750.42	-2.64	40	FKFPGR		Q6PDV7	60S ribosomal protein L10 OS=Rattus norvegicus GN=Rpl10 PE=1 SV=3
16.85	2	379.22	756.43	-3.10	30	IHGVGFK		D4ABZ9	Protein Rpl31-ps8 (Fragment) OS=Rattus norvegicus GN=Rpl31l4 PE=4 SV=2
15.26	2	379.25	756.50	12.21	32	TLVGLVR		F1LQM9	Protein Xpo7 OS=Rattus norvegicus GN=Xpo7 PE=4 SV=2
11.54	2	380.22	758.43	-2.74	28	TILNNGK		P14141	Carbonic anhydrase 3 OS=Rattus norvegicus GN=Ca3 PE=1 SV=3
16.06	2	383.22	764.42	-2.95	31	DSFLKR		D3ZPN7	Protein LOC100360604 OS=Rattus norvegicus GN=LOC100364176 PE=4 SV=1
16.06	2	383.22	764.42	-2.95	31	DSFLKR		D3ZEK2	Protein RGD1562923 OS=Rattus norvegicus GN=RGD1562923 PE=4 SV=1
16.06	2	383.22	764.42	-2.95	31	DSFLKR		M0RCS9	Uncharacterized protein (Fragment) OS=Rattus norvegicus PE=4 SV=1
16.06	2	383.22	764.42	-2.95	31	DSFLKR		D3ZRA9	Uncharacterized protein OS=Rattus norvegicus GN=RGD1560414 PE=4 SV=1
28.25	2	383.22	764.42	-3.34	31	MQIFVK		F1LML2	Polyubiquitin-C OS=Rattus norvegicus GN=Ubc PE=2 SV=1
16.06	2	386.74	771.46	-2.83	32	IGVITNR		P62703	40S ribosomal protein S4. X isoform OS=Rattus norvegicus GN=Rps4x PE=2 SV=2
13.62	2	387.20	772.39	-0.05	50	MGPAIER		F1LV13	Heterogeneous nuclear ribonucleoprotein M OS=Rattus norvegicus GN=Hnrnpm PE=2 SV=1
8.70	2	393.71	785.40	-2.76	58	ASGPGLER		D3ZHA0	Protein Flnc OS=Rattus norvegicus GN=Flnc PE=4 SV=1
14.78	2	393.75	785.49	-2.04	25	RASAILR		Q642E2	Protein LOC100362069 OS=Rattus norvegicus GN=Rpl28 PE=2 SV=1
21.45	2	394.74	787.47	-2.36	37	KLPFQR		B0BMY8	Histone H3 OS=Rattus norvegicus GN=H3f3b PE=2 SV=1
19.36	2	395.71	789.41	-2.98	59	ASVNMLR		B0K031	60S ribosomal protein L7 OS=Rattus norvegicus GN=Rpl7 PE=2 SV=1

13.18	2	396.20	790.38	-2.72	30	VFDPEGK		P08733	Myosin regulatory light chain 2. ventricular/cardiac muscle isoform OS=Rattus norvegicus GN=Myl2 PE=1 SV=2
14.94	2	398.24	794.46	-2.11	34	IIAPPER		P60711	Actin. cytoplasmic 1 OS=Rattus norvegicus GN=Actb PE=1 SV=1
14.94	2	398.24	794.46	-2.11	34	IIAPPER		P68035	Actin. alpha cardiac muscle 1 OS=Rattus norvegicus GN=Actc1 PE=2 SV=1
14.94	2	398.24	794.46	-2.11	34	IIAPPER		P68136	Actin. alpha skeletal muscle OS=Rattus norvegicus GN=Acta1 PE=1 SV=1
14.94	2	398.24	794.46	-2.11	34	IIAPPER		D3ZRN3	Protein Actbl2 OS=Rattus norvegicus GN=Actbl2 PE=3 SV=1
26.05	2	400.77	799.53	-2.12	34	RGILTLK		P68035	Actin. alpha cardiac muscle 1 OS=Rattus norvegicus GN=Actc1 PE=2 SV=1
26.05	2	400.77	799.53	-2.12	34	RGILTLK		P68136	Actin. alpha skeletal muscle OS=Rattus norvegicus GN=Acta1 PE=1 SV=1
26.05	2	400.77	799.53	-2.12	34	RGILTLK		P60711	Actin. cytoplasmic 1 OS=Rattus norvegicus GN=Actb PE=1 SV=1
26.05	2	400.77	799.53	-2.12	34	RGILTLK		D3ZRN3	Protein Actbl2 OS=Rattus norvegicus GN=Actbl2 PE=3 SV=1
14.42	2	404.73	807.45	-2.70	32	TLDGFKK		P04785	Protein disulfide-isomerase OS=Rattus norvegicus GN=P4hb PE=1 SV=2
7.72	2	406.20	810.38	-3.62	37	GTGASGSFK		P15865	Histone H1.4 OS=Rattus norvegicus GN=Hist1h1e PE=1 SV=3
7.72	2	406.20	810.38	-3.62	37	GTGASGSFK		D3ZBN0	Histone H1.5 OS=Rattus norvegicus GN=Hist1h1b PE=3 SV=1
7.72	2	406.20	810.38	-3.62	37	GTGASGSFK		P06349	Histone H1t OS=Rattus norvegicus GN=Hist1h1t PE=1 SV=2
7.72	2	406.20	810.38	-3.62	37	GTGASGSFK		M0R7B4	Protein LOC684828 OS=Rattus norvegicus GN=LOC684828 PE=3 SV=1
9.62	2	407.23	812.44	-2.25	35	QAAEGPLK		M0R590	Protein LOC685186 OS=Rattus norvegicus GN=LOC685186 PE=3 SV=1
24.35	2	407.74	813.46	-2.05	62	TADIPGLK		M0RA80	Protein Tnc OS=Rattus norvegicus GN=Tnc PE=4 SV=1

9.00	2	408.73	815.44	-3.14	43	VEEIAAGK		P62718	60S ribosomal protein L18a OS=Rattus norvegicus GN=Rpl18a PE=2 SV=1
13.10	2	410.23	818.44	-3.02	27	YRPDLR		Q642E2	Protein LOC100362069 OS=Rattus norvegicus GN=Rpl28 PE=2 SV=1
19.67	2	410.75	819.48	-2.86	27	FIKIDGK		P62703	40S ribosomal protein S4. X isoform OS=Rattus norvegicus GN=Rps4x PE=2 SV=2
14.42	2	411.23	820.45	-3.02	47	KGAGSVFR		P62919	60S ribosomal protein L8 OS=Rattus norvegicus GN=Rpl8 PE=2 SV=2
16.46	2	413.77	825.53	-3.02	30	IDKPILK		P62919	60S ribosomal protein L8 OS=Rattus norvegicus GN=Rpl8 PE=2 SV=2
12.00	2	414.25	826.49	-2.51	27	VAAAPGTLK		B0K031	60S ribosomal protein L7 OS=Rattus norvegicus GN=Rpl7 PE=2 SV=1
9.89	2	414.75	827.48	-2.61	52	AAIAAAAAAK		F1LSW7	60S ribosomal protein L14 OS=Rattus norvegicus GN=Rpl14 PE=2 SV=1
10.05	2	415.77	829.52	-3.19	30	IIKTEVK		F1LSW7	60S ribosomal protein L14 OS=Rattus norvegicus GN=Rpl14 PE=2 SV=1
27.46	2	416.25	830.48	-2.40	37	STELLIR		B0BMY8	Histone H3 OS=Rattus norvegicus GN=H3f3b PE=2 SV=1
11.92	2	419.74	837.47	-2.74	34	GAQAPVKAP		M0R665	Protein LOC686807 OS=Rattus norvegicus GN=LOC686807 PE=4 SV=1
8.41	2	423.24	844.46	-3.15	28	KVGDDIAK		B2RYU2	RCG45615. isoform CRA_a OS=Rattus norvegicus GN=Rpl12 PE=2 SV=1
29.30	2	424.73	847.45	-2.77	63	IGADFLGR		P13221	Aspartate aminotransferase. cytoplasmic OS=Rattus norvegicus GN=Got1 PE=1 SV=3
25.92	2	425.77	849.52	-2.44	29	HLQLAIR		M0RDM4	Histone H2A OS=Rattus norvegicus GN=LOC680322 PE=3 SV=1
17.94	2	429.25	856.49	-0.75	32	YAHVVLR		D3ZAU6	Protein RGD1561919 OS=Rattus norvegicus GN=RGD1561919 PE=3 SV=1
17.94	2	429.25	856.49	-0.75	32	YAHVVLR		D3ZII2	Protein RGD1562404 OS=Rattus norvegicus GN=RGD1562404 PE=3 SV=1
14.55	2	430.73	859.44	-2.65	26	GEITGEVR		C0JPT7	Filamin alpha OS=Rattus norvegicus GN=Flna PE=2 SV=1
14.55	2	430.73	859.44	-2.65	26	GELTGEVR		D3ZHA0	Protein Flnc OS=Rattus norvegicus GN=Flnc PE=4 SV=1

20.88	2	431.73	861.44	-1.54	46	VSADAMLR		F1LMC6	Troponin I. slow skeletal muscle (Fragment) OS=Rattus norvegicus GN=Tnni1 PE=4 SV=1
8.92	2	432.71	863.41	-3.11	51	GGAGVGSMTK		D4A6G6	Protein LOC100362339 OS=Rattus norvegicus GN=LOC100362339 PE=4 SV=1
23.20	2	432.75	863.48	-4.11	28	VSIYGVAR		M0RA80	Protein Tnc OS=Rattus norvegicus GN=Tnc PE=4 SV=1
13.34	2	433.22	864.43	-3.13	27	FVEEVSR		F1LNH3	Procollagen. type VI. alpha 2. isoform CRA_a OS=Rattus norvegicus GN=Col6a2 PE=4 SV=2
7.23	2	434.26	866.51	-3.52	30	KVHGSLAR		P62864	40S ribosomal protein S30 OS=Rattus norvegicus GN=Fau PE=1 SV=1
12.55	2	435.28	868.55	-3.83	42	TVIVKGPR		Q6P9U5	Ribosomal protein L9 OS=Rattus norvegicus GN=Rpl9 PE=2 SV=1
6.55	2	436.73	871.45	-3.18	33	KQASGPER		P23928	Alpha-crystallin B chain OS=Rattus norvegicus GN=Cryab PE=1 SV=1
14.49	2	436.76	871.51	-3.47	44	EIVRDIK		P60711	Actin. cytoplasmic 1 OS=Rattus norvegicus GN=Actb PE=1 SV=1
14.49	2	436.76	871.51	-3.47	44	EIVRDIK		P68035	Actin. alpha cardiac muscle 1 OS=Rattus norvegicus GN=Actc1 PE=2 SV=1
14.49	2	436.76	871.51	-3.47	44	EIVRDIK		P68136	Actin. alpha skeletal muscle OS=Rattus norvegicus GN=Acta1 PE=1 SV=1
14.49	2	436.76	871.51	9.50	36	EDLLAAIK		D3Z9F8	Collagen alpha-1(XII) chain OS=Rattus norvegicus GN=Col12a1 PE=4 SV=2
14.49	2	436.76	871.51	9.50	30	ELQELLK		G3V6A8	Golgi autoantigen. golgin subfamily b. macrogolgin 1. isoform CRA_c OS=Rattus norvegicus GN=Golgb1 PE=4 SV=1
14.49	2	436.76	871.51	-3.47	44	KQVQELK		Q5PQT2	Myotubularin-related protein 3 OS=Rattus norvegicus GN=Mtmr3 PE=2 SV=1
14.49	2	436.76	871.51	-3.47	36	QLLSGNLK		B2GV13	Protein LOC100910121 OS=Rattus norvegicus GN=Rrn3 PE=2 SV=1
14.49	2	436.76	871.51	-3.47	41	KNQIEIK		Q5U303	TGF-beta-activated kinase 1 and MAP3K7-binding protein 2 OS=Rattus norvegicus GN=Tab2 PE=2 SV=2
14.49	2	436.76	871.51	-3.47	44	EIVRDIK		F1M648	Uncharacterized protein (Fragment) OS=Rattus norvegicus PE=3 SV=1

14.49	2	436.76	871.51	-3.47	44	EIVRDIK		M0R8W7	Uncharacterized protein OS=Rattus norvegicus PE=3 SV=1
8.70	2	437.25	872.49	-2.86	34	IKELENK		Q811A2	Bone marrow stromal antigen 2 OS=Rattus norvegicus GN=Bst2 PE=1 SV=1
11.76	2	439.21	876.41	-3.16	44	MGANSLER		F1LV13	Heterogeneous nuclear ribonucleoprotein M OS=Rattus norvegicus GN=Hnrnpm PE=2 SV=1
11.39	2	439.72	877.43	-3.30	51	VSADAMLR	[6] Oxidation (M)	F1LMC6	Troponin I. slow skeletal muscle (Fragment) OS=Rattus norvegicus GN=Tnni1 PE=4 SV=1
27.92	2	442.24	882.46	-2.42	32	ILYGDFR		G3V885	Myosin-6 OS=Rattus norvegicus GN=Myh6 PE=4 SV=1
27.92	2	442.24	882.46	-2.42	32	ILYGDFR		P02564	Myosin-7 OS=Rattus norvegicus GN=Myh7 PE=2 SV=2
27.92	2	442.24	882.46	-2.42	32	ILYGDFR		G3V8B0	Myosin-7 OS=Rattus norvegicus GN=Myh7 PE=4 SV=1
11.84	2	442.74	883.47	-3.41	26	MQHLIAR	[1] Oxidation (M)	P11980	Pyruvate kinase PKM OS=Rattus norvegicus GN=Pkm PE=1 SV=3
14.42	2	442.74	883.47	-3.06	56	AGMTHIVR		P21531	60S ribosomal protein L3 OS=Rattus norvegicus GN=Rpl3 PE=1 SV=3
28.64	2	444.24	886.46	-2.89	31	HIDFSLR		P29314	40S ribosomal protein S9 OS=Rattus norvegicus GN=Rps9 PE=1 SV=4
19.28	2	444.26	886.51	-2.95	35	KGDIVDIK		D3ZPN7	Protein LOC100360604 OS=Rattus norvegicus GN=LOC100364176 PE=4 SV=1
19.28	2	444.26	886.51	-2.95	35	KGDIVDIK		D3ZEK2	Protein RGD1562923 OS=Rattus norvegicus GN=RGD1562923 PE=4 SV=1
19.28	2	444.26	886.51	-2.95	35	KGDIVDIK		D3ZRA9	Uncharacterized protein OS=Rattus norvegicus GN=RGD1560414 PE=4 SV=1
19.28	2	444.26	886.51	-2.95	35	KGDIVDIK		D3ZKL4	Uncharacterized protein OS=Rattus norvegicus GN=RGD1561957 PE=4 SV=1
8.41	2	450.74	899.46	-5.17	30	AGMTHIVR	[3] Oxidation (M)	P21531	60S ribosomal protein L3 OS=Rattus norvegicus GN=Rpl3 PE=1 SV=3
19.44	2	451.75	901.48	-3.30	37	LTVDEAVR		P30427	Plectin OS=Rattus norvegicus GN=Plec PE=1 SV=2
17.94	2	452.24	902.46	-2.60	41	GGPLSGPYR		P14141	Carbonic anhydrase 3 OS=Rattus norvegicus GN=Ca3 PE=1 SV=3
10.44	2	452.72	903.42	-4.80	58	MGANNLER		F1LV13	Heterogeneous nuclear ribonucleoprotein M OS=Rattus norvegicus GN=Hnrnpm PE=2 SV=1

14.55	2	453.24	904.46	-3.17	36	SLESINSR		P62890	60S ribosomal protein L30 OS=Rattus norvegicus GN=Rpl30 PE=3 SV=2
25.84	2	454.25	906.48	-2.17	51	FEEILTR		P00564	Creatine kinase M-type OS=Rattus norvegicus GN=Ckm PE=1 SV=2
8.86	2	455.24	908.46	-3.16	53	VGNEYVTK		G3V885	Myosin-6 OS=Rattus norvegicus GN=Myh6 PE=4 SV=1
8.86	2	455.24	908.46	-3.16	53	VGNEYVTK		B6RK61	Myosin heavy chain 7B OS=Rattus norvegicus GN=Myh7b PE=2 SV=1
8.86	2	455.24	908.46	-3.16	53	VGNEYVTK		G3V6D8	Myosin-3 OS=Rattus norvegicus GN=Myh3 PE=4 SV=1
8.86	2	455.24	908.46	-3.16	53	VGNEYVTK		F1LMU0	Myosin-4 OS=Rattus norvegicus GN=Myh4 PE=2 SV=1
8.86	2	455.24	908.46	-3.16	53	VGNEYVTK		P02564	Myosin-7 OS=Rattus norvegicus GN=Myh7 PE=2 SV=2
8.86	2	455.24	908.46	-3.16	53	VGNEYVTK		G3V8B0	Myosin-7 OS=Rattus norvegicus GN=Myh7 PE=4 SV=1
8.86	2	455.24	908.46	-3.16	53	VGNEYVTK		F1M8F6	Myosin-8 (Fragment) OS=Rattus norvegicus GN=Myh8 PE=4 SV=2
8.86	2	455.24	908.46	-3.16	53	VGNEYVTK		F1LRV9	Protein Myh1 OS=Rattus norvegicus GN=Myh1 PE=2 SV=2
8.86	2	455.24	908.46	-3.16	53	VGNEYVTK		F1M789	Protein Myh13 OS=Rattus norvegicus GN=Myh13 PE=4 SV=2
8.86	2	455.24	908.46	-3.16	53	VGNEYVTK		G3V6E1	Uncharacterized protein OS=Rattus norvegicus GN=Myh2 PE=4 SV=2
9.62	2	456.21	910.41	-2.77	26	LMVEMEK	[2] Oxidation (M)|[5] Oxidation (M)	P00564	Creatine kinase M-type OS=Rattus norvegicus GN=Ckm PE=1 SV=2
9.39	2	456.26	910.50	-2.80	32	GMLPHKTK		Q5RK10	60S ribosomal protein L13a OS=Rattus norvegicus GN=Rpl13a PE=2 SV=1
9.89	2	456.74	911.47	-3.32	35	VHLTDAEK		P02091	Hemoglobin subunit beta-1 OS=Rattus norvegicus GN=Hbb PE=1 SV=3
9.89	2	456.74	911.47	-3.32	35	VHLTDAEK		M3ZCQ3	Protein LOC100910765 OS=Rattus norvegicus GN=LOC100910765 PE=3 SV=1
14.42	2	457.24	912.46	-2.79	61	GAPEGVIDR		E9PSX6	Sarcoplasmic/endoplasmic reticulum calcium ATPase 2 OS=Rattus norvegicus GN=Atp2a2 PE=3 SV=2
14.42	2	457.24	912.46	-2.79	61	GAPEGVIDR		B4F7E5	ATPase. Ca++ transporting. cardiac muscle. fast twitch 1 OS=Rattus norvegicus GN=Atp2a1 PE=2 SV=1

23.04	2	457.79	913.56	-2.27	30	QTVAVGVIK		P62632	Elongation factor 1-alpha 2 OS=Rattus norvegicus GN=Eef1a2 PE=2 SV=1
23.04	2	457.79	913.56	-2.27	30	QTVAVGVIK		F1M6C2	Elongation factor 1-alpha (Fragment) OS=Rattus norvegicus GN=LOC100360150 PE=3 SV=1
31.12	2	460.24	918.47	-2.98	37	APAMFNIR		P49242	40S ribosomal protein S3a OS=Rattus norvegicus GN=Rps3a PE=1 SV=2
31.12	2	460.24	918.47	-2.98	37	APAMFNIR		Q6TXJ6	LRRGT00003 OS=Rattus norvegicus GN=LOC100362727 PE=2 SV=1
38.59	2	460.76	919.50	-2.66	33	DIGFIKLD		P62275	40S ribosomal protein S29 OS=Rattus norvegicus GN=Rps29 PE=1 SV=2
11.39	2	462.29	922.56	-3.20	38	IIAPPERK		P60711	Actin. cytoplasmic 1 OS=Rattus norvegicus GN=Actb PE=1 SV=1
11.39	2	462.29	922.56	-3.20	38	IIAPPERK		P68035	Actin. alpha cardiac muscle 1 OS=Rattus norvegicus GN=Actc1 PE=2 SV=1
11.39	2	462.29	922.56	-3.20	38	IIAPPERK		P68136	Actin. alpha skeletal muscle OS=Rattus norvegicus GN=Acta1 PE=1 SV=1
11.39	2	462.29	922.56	-3.20	38	IIAPPERK		D3ZRN3	Protein Actbl2 OS=Rattus norvegicus GN=Actbl2 PE=3 SV=1
15.75	2	462.75	923.48	-3.10	55	ELADIAHR		P05065	Fructose-bisphosphate aldolase A OS=Rattus norvegicus GN=Aldoa PE=1 SV=2
10.00	2	463.26	924.50	-3.64	31	IEISQHAK		D4A7B1	60S ribosomal protein L37a OS=Rattus norvegicus GN=Rpl37a PE=3 SV=1
19.60	2	463.26	924.52	-3.05	26	AIFAGYKR		D4A771	Protein LOC100362049 OS=Rattus norvegicus GN=LOC100362049 PE=4 SV=1
18.88	2	464.26	926.50	-2.64	32	AHSIQIMK		P62718	60S ribosomal protein L18a OS=Rattus norvegicus GN=Rpl18a PE=2 SV=1
25.59	2	464.26	926.51	-2.42	54	NGAIHIFR		D3ZAF5	Periostin. osteoblast specific factor (Predicted). isoform CRA_a OS=Rattus norvegicus GN=Postn PE=4 SV=1
11.84	2	465.75	929.49	-3.28	29	LREDLER		D3ZAU6	Protein RGD1561919 OS=Rattus norvegicus GN=RGD1561919 PE=3 SV=1
22.16	2	466.77	931.52	-2.37	26	FRSNLPAK		D4A771	Protein LOC100362049 OS=Rattus norvegicus GN=LOC100362049 PE=4 SV=1

22.16	2	466.77	931.52	-2.37	29	SFRNPLAK		M0R5J4	Uncharacterized protein OS=Rattus norvegicus PE=3 SV=1
23.36	2	468.24	934.47	-2.11	48	APAMFNIR	[4] Oxidation (M)	P49242	40S ribosomal protein S3a OS=Rattus norvegicus GN=Rps3a PE=1 SV=2
23.36	2	468.24	934.47	-2.11	48	APAMFNIR	[4] Oxidation (M)	Q6TXJ6	LRRGT00003 OS=Rattus norvegicus GN=LOC100362727 PE=2 SV=1
6.58	2	468.80	935.58	-2.98	30	KPPGSLLPK		B3DMA2	Acyl-CoA dehydrogenase family member 11 OS=Rattus norvegicus GN=Acad11 PE=1 SV=1
6.59	3	312.87	935.58	-2.42	27	KPPGSLLPK		B3DMA2	Acyl-CoA dehydrogenase family member 11 OS=Rattus norvegicus GN=Acad11 PE=1 SV=1
26.37	2	469.26	936.50	-2.46	42	LGSFGSITR		D3ZHA0	Protein Flnc OS=Rattus norvegicus GN=Flnc PE=4 SV=1
13.34	2	469.73	937.45	-2.91	29	YEITEQR		F1LQS3	60S ribosomal protein L6 OS=Rattus norvegicus GN=Rpl6 PE=3 SV=1
13.48	2	469.79	937.57	-3.24	36	KAHLGTALK		P62268	40S ribosomal protein S23 OS=Rattus norvegicus GN=Rps23 PE=1 SV=3
24.01	2	471.24	940.46	-2.14	33	YKELGFQG		Q9QZ76	Myoglobin OS=Rattus norvegicus GN=Mb PE=1 SV=3
16.46	2	471.28	940.54	-2.82	64	AVVGVVAGGGR		P62919	60S ribosomal protein L8 OS=Rattus norvegicus GN=Rpl8 PE=2 SV=2
19.91	2	471.77	941.53	-2.68	57	NIVEAAAVR		P62856	40S ribosomal protein S26 OS=Rattus norvegicus GN=Rps26 PE=3 SV=3
12.94	2	472.26	942.50	0.77	30	AHSIQIMK	[7] Oxidation (M)	P62718	60S ribosomal protein L18a OS=Rattus norvegicus GN=Rpl18a PE=2 SV=1
30.69	2	472.77	943.52	-2.14	56	AGLQFPVGR		M0RDM4	Histone H2A OS=Rattus norvegicus GN=LOC680322 PE=3 SV=1
9.67	2	476.82	951.62	8.49	26	KPLIVASPK		Q4KLP0	Probable 2-oxoglutarate dehydrogenase E1 component DHKTD1. mitochondrial OS=Rattus norvegicus GN=Dhtkd1 PE=2 SV=1
34.16	2	477.30	952.59	-2.44	43	LLLPGELAK		G3V8B3	Histone H2B OS=Rattus norvegicus GN=LOC684797 PE=3 SV=1
8.86	2	478.30	954.58	-3.08	30	VAAAPGTLKK		B0K031	60S ribosomal protein L7 OS=Rattus norvegicus GN=Rpl7 PE=2 SV=1

25.59	2	479.27	956.53	-2.32	38	TIGISVDPR		P41123	60S ribosomal protein L13 OS=Rattus norvegicus GN=Rpl13 PE=1 SV=2
25.59	2	479.27	956.53	-2.32	38	TIGISVDPR		F1M2E9	60S ribosomal protein L13 OS=Rattus norvegicus PE=3 SV=2
15.02	2	480.24	958.47	-2.93	48	DDEVQVVR		G3V6I9	60S ribosomal protein L26 OS=Rattus norvegicus GN=Rpl26 PE=3 SV=1
15.02	2	480.24	958.47	-2.93	48	DDEVQVVR		M0RA61	Uncharacterized protein OS=Rattus norvegicus PE=4 SV=1
11.92	2	480.25	958.48	-2.47	41	LYDNHLGK		G3V885	Myosin-6 OS=Rattus norvegicus GN=Myh6 PE=4 SV=1
11.92	2	480.25	958.48	-2.47	41	LYDNHLGK		P02564	Myosin-7 OS=Rattus norvegicus GN=Myh7 PE=2 SV=2
11.92	2	480.25	958.48	-2.47	41	LYDNHLGK		G3V8B0	Myosin-7 OS=Rattus norvegicus GN=Myh7 PE=4 SV=1
9.23	2	481.74	961.46	-2.77	39	LTEDEVEK		P16409	Myosin light chain 3 OS=Rattus norvegicus GN=Myl3 PE=2 SV=2
16.14	2	484.22	966.42	-2.98	49	MMNTDLSR		Q6P3V9	60S ribosomal protein L4 OS=Rattus norvegicus GN=Rpl4 PE=2 SV=1
18.63	2	484.28	966.55	-2.80	45	HVLATLGEK		P02600	Myosin light chain 1/3. skeletal muscle isoform OS=Rattus norvegicus GN=Myl1 PE=1 SV=2
18.63	2	484.28	966.55	-2.80	45	HVLATLGEK		M0R4E1	Myosin light chain 4 OS=Rattus norvegicus GN=Myl4 PE=4 SV=1
10.56	2	485.27	968.52	-3.83	33	IIAHTQMR		P21531	60S ribosomal protein L3 OS=Rattus norvegicus GN=Rpl3 PE=1 SV=3
31.65	2	486.79	971.56	-2.26	44	QTALAELVK		P02770	Serum albumin OS=Rattus norvegicus GN=Alb PE=1 SV=2
10.32	2	487.27	972.52	-3.80	37	LREEIEGK		D3ZAF5	Periostin. osteoblast specific factor (Predicted). isoform CRA_a OS=Rattus norvegicus GN=Postn PE=4 SV=1
23.52	2	487.30	972.59	-2.80	60	SGVSLAALKK		P15865	Histone H1.4 OS=Rattus norvegicus GN=Hist1h1e PE=1 SV=3
23.52	2	487.30	972.59	-2.80	60	SGVSLAALKK		M0R7B4	Protein LOC684828 OS=Rattus norvegicus GN=LOC684828 PE=3 SV=1
52.64	2	488.73	975.44	-3.31	41	AGFAGDDAPR		P68035	Actin. alpha cardiac muscle 1 OS=Rattus norvegicus GN=Actc1 PE=2 SV=1

52.64	2	488.73	975.44	-3.31	41	AGFAGDDAPR		P68136	Actin. alpha skeletal muscle OS=Rattus norvegicus GN=Acta1 PE=1 SV=1
52.64	2	488.73	975.44	-3.31	41	AGFAGDDAPR		P60711	Actin. cytoplasmic 1 OS=Rattus norvegicus GN=Actb PE=1 SV=1
2.40	2	488.73	975.44	-2.82	31	AGFAGDDAPR		P68035	Actin. alpha cardiac muscle 1 OS=Rattus norvegicus GN=Actc1 PE=2 SV=1
2.40	2	488.73	975.44	-2.82	31	AGFAGDDAPR		P68136	Actin. alpha skeletal muscle OS=Rattus norvegicus GN=Acta1 PE=1 SV=1
2.40	2	488.73	975.44	-2.82	31	AGFAGDDAPR		P60711	Actin. cytoplasmic 1 OS=Rattus norvegicus GN=Actb PE=1 SV=1
12.40	2	488.73	975.44	-1.77	70	AGFAGDDAPR		P68035	Actin. alpha cardiac muscle 1 OS=Rattus norvegicus GN=Actc1 PE=2 SV=1
12.40	2	488.73	975.44	-1.77	70	AGFAGDDAPR		P68136	Actin. alpha skeletal muscle OS=Rattus norvegicus GN=Acta1 PE=1 SV=1
12.40	2	488.73	975.44	-1.77	70	AGFAGDDAPR		P60711	Actin. cytoplasmic 1 OS=Rattus norvegicus GN=Actb PE=1 SV=1
29.22	2	490.25	978.49	-2.78	33	LMQVEFGR		D4A111	Protein Col6a3 OS=Rattus norvegicus GN=Col6a3 PE=4 SV=2
11.39	2	490.75	979.50	-3.69	26	STHPHFVR		F1LMU0	Myosin-4 OS=Rattus norvegicus GN=Myh4 PE=2 SV=1
11.39	2	490.75	979.50	-3.69	26	STHPHFVR		P02564	Myosin-7 OS=Rattus norvegicus GN=Myh7 PE=2 SV=2
11.39	2	490.75	979.50	-3.69	26	STHPHFVR		G3V8B0	Myosin-7 OS=Rattus norvegicus GN=Myh7 PE=4 SV=1
11.39	2	490.75	979.50	-3.69	26	STHPHFVR		F1M8F6	Myosin-8 (Fragment) OS=Rattus norvegicus GN=Myh8 PE=4 SV=2
11.39	2	490.75	979.50	-3.69	26	STHPHFVR		F1LRV9	Protein Myh1 OS=Rattus norvegicus GN=Myh1 PE=2 SV=2
11.39	2	490.75	979.50	-3.69	26	STHPHFVR		F1M789	Protein Myh13 OS=Rattus norvegicus GN=Myh13 PE=4 SV=2
11.39	2	490.75	979.50	-3.69	26	STHPHFVR		G3V6E1	Uncharacterized protein OS=Rattus norvegicus GN=Myh2 PE=4 SV=2
9.00	2	492.22	982.42	-3.39	43	MMNTDLSR	[2] Oxidation (M)	Q6P3V9	60S ribosomal protein L4 OS=Rattus norvegicus GN=Rpl4 PE=2 SV=1

33.64	2	494.31	986.60	-3.19	35	RVPFSLLR		G3V913	Heat shock 27kDa protein 1 OS=Rattus norvegicus GN=Hspb1 PE=3 SV=1
6.58	2	495.25	988.49	-3.01	33	EAAGAGSGGGKK		G3V7Q2	Hepatocyte nuclear factor 3-beta OS=Rattus norvegicus GN=Foxa2 PE=4 SV=1
10.32	2	495.27	988.52	-3.20	73	LSKEDIER		P63018	Heat shock cognate 71 kDa protein OS=Rattus norvegicus GN=Hspa8 PE=1 SV=1
36.26	2	495.80	989.59	-2.58	68	LSNIFVIGK		P62703	40S ribosomal protein S4. X isoform OS=Rattus norvegicus GN=Rps4x PE=2 SV=2
19.44	2	498.28	994.55	-3.09	54	HVLATLGER		P16409	Myosin light chain 3 OS=Rattus norvegicus GN=Myl3 PE=2 SV=2
8.86	2	499.25	996.48	-3.48	36	LSYNTASNK		F1LQ14	60S ribosomal protein L34 OS=Rattus norvegicus GN=Rpl34 PE=2 SV=1
34.76	2	499.75	997.48	-3.34	38	DLTDYLMK		P60711	Actin. cytoplasmic 1 OS=Rattus norvegicus GN=Actb PE=1 SV=1
34.76	2	499.75	997.48	-3.34	38	DLTDYLMK		P68035	Actin. alpha cardiac muscle 1 OS=Rattus norvegicus GN=Actc1 PE=2 SV=1
34.76	2	499.75	997.48	-3.34	38	DLTDYLMK		P68136	Actin. alpha skeletal muscle OS=Rattus norvegicus GN=Acta1 PE=1 SV=1
34.76	2	499.75	997.48	-3.34	38	DLTDYLMK		D3ZRN3	Protein Actbl2 OS=Rattus norvegicus GN=Actbl2 PE=3 SV=1
22.49	2	500.26	998.50	-2.58	27	ELPNIEER		D4A111	Protein Col6a3 OS=Rattus norvegicus GN=Col6a3 PE=4 SV=2
21.76	2	500.29	998.57	-2.94	72	LEAAAAALAAK		Q6P3V9	60S ribosomal protein L4 OS=Rattus norvegicus GN=Rpl4 PE=2 SV=1
7.87	2	501.26	1000.51	-3.18	33	HKVSMDLR	[5] Oxidation (M)	F1LMC6	Troponin I. slow skeletal muscle (Fragment) OS=Rattus norvegicus GN=Tnni1 PE=4 SV=1
17.62	2	501.76	1001.51	-3.56	81	ADVDAATLAR		Q6P725	Desmin OS=Rattus norvegicus GN=Des PE=2 SV=1
10.63	2	501.78	1001.55	-2.75	48	LKAEGSEIR		P04785	Protein disulfide-isomerase OS=Rattus norvegicus GN=P4hb PE=1 SV=2
10.63	2	501.78	1001.55	-14.03	36	IKANTVSGGR		D3ZTL8	Protein Rapgef6 OS=Rattus norvegicus GN=Rapgef6 PE=4 SV=1
31.65	2	502.30	1002.58	-2.79	25	APITGYIIR		F1LST1	Fibronectin OS=Rattus norvegicus GN=Fn1 PE=4 SV=2

9.31	2	504.30	1006.59	-2.88	44	LANLSKHPK		P00564	Creatine kinase M-type OS=Rattus norvegicus GN=Ckm PE=1 SV=2
26.45	2	504.31	1006.60	-2.66	28	IKFPLPHR		P62718	60S ribosomal protein L18a OS=Rattus norvegicus GN=Rpl18a PE=2 SV=1
15.42	2	504.78	1007.54	-3.47	38	GSLKADYVR		P08733	Myosin regulatory light chain 2. ventricular/cardiac muscle isoform OS=Rattus norvegicus GN=Myl2 PE=1 SV=2
22.00	2	507.22	1012.43	-2.54	32	GGNFGFGDSR		M0R6J9	Heterogeneous nuclear ribonucleoproteins A2/B1 OS=Rattus norvegicus GN=Hnrnpa2b1 PE=4 SV=1
30.87	2	507.26	1012.50	-2.36	37	MFAAFPTTK		B1H216	Hemoglobin alpha. adult chain 2 OS=Rattus norvegicus GN=Hba1 PE=2 SV=1
20.48	2	508.74	1015.47	-2.80	62	VDFDDIHR		M0R799	Troponin T. slow skeletal muscle OS=Rattus norvegicus GN=Tnnt1 PE=4 SV=1
10.85	2	509.29	1016.56	-3.03	41	ITITNDKGR		P63018	Heat shock cognate 71 kDa protein OS=Rattus norvegicus GN=Hspa8 PE=1 SV=1
10.85	2	509.29	1016.56	-3.03	41	ITITNDKGR		Q07439	Heat shock 70 kDa protein 1A/1B OS=Rattus norvegicus GN=Hspa1a PE=2 SV=2
11.31	2	510.26	1018.50	-3.67	43	SVNDLTSQR		G3V8B0	Myosin-7 OS=Rattus norvegicus GN=Myh7 PE=4 SV=1
25.51	2	510.29	1018.57	-1.99	63	AFKAWAVAR		P02770	Serum albumin OS=Rattus norvegicus GN=Alb PE=1 SV=2
15.11	2	511.27	1020.53	-3.23	40	RFVEEVSR		F1LNH3	Procollagen. type VI. alpha 2. isoform CRA_a OS=Rattus norvegicus GN=Col6a2 PE=4 SV=2
24.86	2	513.30	1024.59	-2.56	29	AVAEPGIQLK		M0RBU0	Cartilage oligomeric matrix protein OS=Rattus norvegicus GN=Comp PE=4 SV=1
25.59	2	513.31	1024.60	-2.23	70	IGGIGTVPVGR		P62632	Elongation factor 1-alpha 2 OS=Rattus norvegicus GN=Eef1a2 PE=2 SV=1
25.59	2	513.31	1024.60	-2.23	70	IGGIGTVPVGR		F1M6C2	Elongation factor 1-alpha (Fragment) OS=Rattus norvegicus GN=LOC100360150 PE=3 SV=1
20.88	2	516.78	1031.54	-0.41	70	TSGGAGGLGSLR		Q6P725	Desmin OS=Rattus norvegicus GN=Des PE=2 SV=1
33.31	2	516.79	1031.57	-3.05	59	LFEGNALLR		P29314	40S ribosomal protein S9 OS=Rattus norvegicus GN=Rps9 PE=1 SV=4

18.02	2	516.80	1031.58	-3.75	44	YRPGTVALR		B0BMY8	Histone H3 OS=Rattus norvegicus GN=H3f3b PE=2 SV=1
20.80	3	344.87	1031.59	-2.02	52	VKVGVNGFGR		M0R590	Protein LOC685186 OS=Rattus norvegicus GN=LOC685186 PE=3 SV=1
10.48	2	517.26	1032.50	-3.13	46	HTDPVPDPR		E9PSX6	Sarcoplasmic/endoplasmic reticulum calcium ATPase 2 OS=Rattus norvegicus GN=Atp2a2 PE=3 SV=2
10.48	2	517.26	1032.50	-3.13	46	HTDPVPDPR		B4F7E5	ATPase. Ca++ transporting. cardiac muscle. fast twitch 1 OS=Rattus norvegicus GN=Atp2a1 PE=2 SV=1
10.48	2	517.26	1032.50	-3.13	46	HTDPVPDPR		M0RCD2	Sarcoplasmic/endoplasmic reticulum calcium ATPase 1 OS=Rattus norvegicus GN=Atp2a1 PE=3 SV=1
15.02	2	517.72	1033.43	-1.88	27	DHFEEAMR		P46462	Transitional endoplasmic reticulum ATPase OS=Rattus norvegicus GN=Vcp PE=1 SV=3
12.40	2	518.78	1035.55	-4.67	25	ILMEHIHK	[3] Oxidation (M)	P84100	60S ribosomal protein L19 OS=Rattus norvegicus GN=Rpl19 PE=1 SV=1
23.12	2	518.83	1035.64	-2.99	36	IKIIAPPER		P60711	Actin. cytoplasmic 1 OS=Rattus norvegicus GN=Actb PE=1 SV=1
23.12	2	518.83	1035.64	-2.99	36	IKIIAPPER		P68035	Actin. alpha cardiac muscle 1 OS=Rattus norvegicus GN=Actc1 PE=2 SV=1
23.12	2	518.83	1035.64	-2.99	36	IKIIAPPER		P68136	Actin. alpha skeletal muscle OS=Rattus norvegicus GN=Acta1 PE=1 SV=1
23.12	2	518.83	1035.64	-2.99	36	IKIIAPPER		D3ZRN3	Protein Actbl2 OS=Rattus norvegicus GN=Actbl2 PE=3 SV=1
23.12	2	518.83	1035.64	-2.99	32	IEIIAPPKR		M0R8W7	Uncharacterized protein OS=Rattus norvegicus PE=3 SV=1
15.91	2	519.27	1036.53	-3.80	70	IASNSATAFR		D4A111	Protein Col6a3 OS=Rattus norvegicus GN=Col6a3 PE=4 SV=2
25.67	2	521.80	1041.58	-2.91	67	LAAEQELIR		P30427	Plectin OS=Rattus norvegicus GN=Plec PE=1 SV=2
20.23	2	522.27	1042.52	-2.61	43	VSITEAMHR		P30427	Plectin OS=Rattus norvegicus GN=Plec PE=1 SV=2
15.26	2	522.31	1042.61	-3.42	40	ELTAVVQKR		P62909	40S ribosomal protein S3 OS=Rattus norvegicus GN=Rps3 PE=1 SV=1
13.86	2	523.76	1045.50	-2.78	52	SLAAEEEAAR		P30427	Plectin OS=Rattus norvegicus GN=Plec PE=1 SV=2

14.10	2	524.80	1047.58	-3.26	35	LLPAAEAHAR		A0JPQ4	Tripartite motif-containing protein 72 OS=Rattus norvegicus GN=Trim72 PE=2 SV=1
11.76	2	525.24	1048.46	-3.32	64	AMNGESLDGR		G3V6P6	Putative RNA-binding protein 3 OS=Rattus norvegicus GN=Rbm3 PE=4 SV=1
9.00	2	526.74	1051.46	-3.55	74	DANGNSFATR		P62703	40S ribosomal protein S4. X isoform OS=Rattus norvegicus GN=Rps4x PE=2 SV=2
10.63	2	527.30	1052.59	-3.31	40	KIEISQHAK		D4A7B1	60S ribosomal protein L37a OS=Rattus norvegicus GN=Rpl37a PE=3 SV=1
12.40	2	527.78	1053.55	-4.62	66	TPGPGAQSALR		Q6PDV6	40S ribosomal protein S14 OS=Rattus norvegicus GN=Rps14 PE=2 SV=1
12.87	2	528.76	1055.51	-4.37	30	ANPFGGASHAK		P62268	40S ribosomal protein S23 OS=Rattus norvegicus GN=Rps23 PE=1 SV=3
37.81	2	531.28	1060.54	6.88	48	IPDWFLDR		D3ZII2	Protein RGD1562404 OS=Rattus norvegicus GN=RGD1562404 PE=3 SV=1
25.35	2	531.79	1061.57	-2.62	32	VLTPDLYNK		P00564	Creatine kinase M-type OS=Rattus norvegicus GN=Ckm PE=1 SV=2
7.80	2	532.26	1062.52	-4.01	49	KGDSSAEELK		P41123	60S ribosomal protein L13 OS=Rattus norvegicus GN=Rpl13 PE=1 SV=2
7.80	2	532.26	1062.52	-4.01	49	KGDSSAEELK		F1M2E9	60S ribosomal protein L13 OS=Rattus norvegicus PE=3 SV=2
21.60	2	533.26	1064.51	-2.33	32	FLGDEETVR		P16290	Phosphoglycerate mutase 2 OS=Rattus norvegicus GN=Pgam2 PE=2 SV=2
16.78	2	533.76	1065.51	-3.07	52	TVIDYNGER		P04785	Protein disulfide-isomerase OS=Rattus norvegicus GN=P4hb PE=1 SV=2
9.23	2	533.78	1065.54	-3.26	29	GKDSLYAQGK		B2RYQ8	Large subunit ribosomal protein L36a. isoform CRA_a OS=Rattus norvegicus GN=Rpl36al PE=3 SV=1
13.40	2	536.26	1070.51	-3.16	38	RDDDPLNAR		F1LNH3	Procollagen. type VI. alpha 2. isoform CRA_a OS=Rattus norvegicus GN=Col6a2 PE=4 SV=2
13.40	2	536.26	1070.51	7.30	39	GVDNGEDIPR		D3ZV71	Protein Iqsec2 OS=Rattus norvegicus GN=Iqsec2 PE=4 SV=1
25.35	2	536.30	1070.59	-2.36	41	VITIMQNPR		D3ZAU6	Protein RGD1561919 OS=Rattus norvegicus GN=RGD1561919 PE=3 SV=1

25.35	2	536.30	1070.59	-2.36	41	VITIMQNPR		D3ZII2	Protein RGD1562404 OS=Rattus norvegicus GN=RGD1562404 PE=3 SV=1
8.70	2	537.28	1072.55	-2.94	58	LDKENALDR		P04692	Tropomyosin alpha-1 chain OS=Rattus norvegicus GN=Tpm1 PE=1 SV=3
8.70	2	537.28	1072.55	-2.94	58	LDKENAIDR		Q5FVG5	Similar to tropomyosin 1. embryonic fibroblast-rat. isoform CRA_c OS=Rattus norvegicus GN=Tpm2 PE=2 SV=1
26.00	2	537.32	1072.63	-2.49	32	KVVNPLFEK		D3ZPL5	Protein LOC100361311 OS=Rattus norvegicus GN=RGD1562953 PE=4 SV=1
19.60	2	538.29	1074.56	-2.93	39	QLSSGVSEIR		G3V913	Heat shock 27kDa protein 1 OS=Rattus norvegicus GN=Hspb1 PE=3 SV=1
22.33	2	538.79	1075.57	-2.49	46	LRAVDFAER		P62919	60S ribosomal protein L8 OS=Rattus norvegicus GN=Rpl8 PE=2 SV=2
14.02	2	539.76	1077.51	-3.09	45	EMLTTQAER		P08733	Myosin regulatory light chain 2. ventricular/cardiac muscle isoform OS=Rattus norvegicus GN=Myl2 PE=1 SV=2
14.94	2	539.78	1077.54	-3.17	31	HFNAPSHIR		G3V6I9	60S ribosomal protein L26 OS=Rattus norvegicus GN=Rpl26 PE=3 SV=1
14.94	2	539.78	1077.54	-3.17	31	HFNAPSHIR		M0R6L3	Protein LOC100910721 OS=Rattus norvegicus GN=LOC100910721 PE=3 SV=1
14.94	3	360.19	1077.54	-2.12	44	HFNAPSHIR		G3V6I9	60S ribosomal protein L26 OS=Rattus norvegicus GN=Rpl26 PE=3 SV=1
14.94	3	360.19	1077.54	-2.12	44	HFNAPSHIR		M0R6L3	Protein LOC100910721 OS=Rattus norvegicus GN=LOC100910721 PE=3 SV=1
18.79	2	541.28	1080.54	-2.56	38	TLSDYNIQK		F1LML2	Polyubiquitin-C OS=Rattus norvegicus GN=Ubc PE=2 SV=1
21.84	2	541.33	1080.65	-2.57	28	KVPAVPETLK		B0K031	60S ribosomal protein L7 OS=Rattus norvegicus GN=Rpl7 PE=2 SV=1
11.03	2	542.27	1082.53	-3.41	26	TDLNHENLK		P00564	Creatine kinase M-type OS=Rattus norvegicus GN=Ckm PE=1 SV=2
37.98	2	542.30	1082.59	-2.06	51	VLPGVDALSNV		M0R6Y8	Phosphoglycerate kinase OS=Rattus norvegicus GN=RGD1560402 PE=3 SV=1

17.24	2	542.33	1082.64	-3.06	41	ILKSPEIQR		Q6P3V9	60S ribosomal protein L4 OS=Rattus norvegicus GN=Rpl4 PE=2 SV=1
17.17	3	361.89	1082.64	-2.93	33	ILKSPEIQR		Q6P3V9	60S ribosomal protein L4 OS=Rattus norvegicus GN=Rpl4 PE=2 SV=1
16.69	2	543.80	1085.58	-3.13	34	QVEVLTNQR		Q6P725	Desmin OS=Rattus norvegicus GN=Des PE=2 SV=1
14.70	2	544.76	1087.50	-3.19	33	QDEHGFISR		P23928	Alpha-crystallin B chain OS=Rattus norvegicus GN=Cryab PE=1 SV=1
24.17	2	544.81	1087.60	-1.91	42	EKGTWVQLK		D3ZPN7	Protein LOC100360604 OS=Rattus norvegicus GN=LOC100364176 PE=4 SV=1
24.17	2	544.81	1087.60	-1.91	42	EKGTWVQLK		D3ZEK2	Protein RGD1562923 OS=Rattus norvegicus GN=RGD1562923 PE=4 SV=1
24.17	2	544.81	1087.60	-1.91	42	EKGTWVQLK		M0RCS9	Uncharacterized protein (Fragment) OS=Rattus norvegicus PE=4 SV=1
24.17	2	544.81	1087.60	-1.91	42	EKGTWVQLK		D3ZRA9	Uncharacterized protein OS=Rattus norvegicus GN=RGD1560414 PE=4 SV=1
17.17	2	545.28	1088.54	-2.96	35	SLETENAGLR		G3V8L3	Lamin A. isoform CRA_b OS=Rattus norvegicus GN=Lmna PE=3 SV=1
29.38	2	546.29	1090.57	-2.19	47	VNFTVDQIR		P05197	Elongation factor 2 OS=Rattus norvegicus GN=Eef2 PE=1 SV=4
23.61	2	546.29	1090.57	-1.93	34	GLSQSALPYR		M0RBC7	Protein Bco2 (Fragment) OS=Rattus norvegicus GN=Bco2 PE=3 SV=1
23.61	2	546.29	1090.57	-1.93	34	GLSQSALPYR		M0RCY2	Protein LOC683961 OS=Rattus norvegicus GN=LOC683961 PE=3 SV=1
20.07	2	546.79	1091.57	-2.23	42	GLVGPELHDR		P30427	Plectin OS=Rattus norvegicus GN=Plec PE=1 SV=2
20.07	2	547.27	1092.52	-1.99	37	FADLSEAANR		P31000	Vimentin OS=Rattus norvegicus GN=Vim PE=1 SV=2
21.76	2	547.75	1093.48	-2.59	33	DFADMPNLR	[5] Oxidation (M)	D3ZVB7	Osteoglycin (Predicted) OS=Rattus norvegicus GN=LOC100910855 PE=4 SV=1
8.65	2	547.76	1093.50	-2.98	54	EMLTTQAER	[2] Oxidation (M)	P08733	Myosin regulatory light chain 2. ventricular/cardiac muscle isoform OS=Rattus norvegicus GN=Myl2 PE=1 SV=2
42.34	2	547.86	1093.71	-2.79	45	LLEPVLLLGK		B0K038	Rps16 protein (Fragment) OS=Rattus norvegicus GN=Rps16 PE=2 SV=1

20.96	2	550.24	1098.46	-1.84	39	VEFMDDTSR		P62859	40S ribosomal protein S28 OS=Rattus norvegicus GN=Rps28 PE=1 SV=1
27.96	2	550.84	1099.66	-2.49	71	KQTALAELVK		P02770	Serum albumin OS=Rattus norvegicus GN=Alb PE=1 SV=2
7.95	2	551.76	1101.51	-3.39	43	EGKTDYYAR		P09895	60S ribosomal protein L5 OS=Rattus norvegicus GN=Rpl5 PE=1 SV=3
17.70	2	552.27	1102.52	-3.11	32	SNYNLPMHK		Q6P3V9	60S ribosomal protein L4 OS=Rattus norvegicus GN=Rpl4 PE=2 SV=1
19.03	2	553.28	1104.55	-2.86	65	APTAQVESFR		M0RA80	Protein Tnc OS=Rattus norvegicus GN=Tnc PE=4 SV=1
11.76	2	553.79	1105.56	-3.45	44	GTVQTGLDTSK		M0R7S5	Protein Plin4 OS=Rattus norvegicus GN=Plin4 PE=4 SV=1
9.96	2	554.29	1106.57	-3.73	36	AAQEEYIKR		P05065	Fructose-bisphosphate aldolase A OS=Rattus norvegicus GN=Aldoa PE=1 SV=2
9.96	2	554.29	1106.57	-3.73	36	AAQEEYIKR		Q6AY07	Fructose-bisphosphate aldolase OS=Rattus norvegicus GN=Aldoart2 PE=2 SV=1
17.17	2	554.80	1107.59	-3.60	27	YKEVAELTR		G3V6S0	Protein Sptbn1 OS=Rattus norvegicus GN=Sptbn1 PE=4 SV=2
6.97	2	554.80	1107.59	-3.49	43	EAPAPPKAEAK		F1LT35	Protein RGD1564606 (Fragment) OS=Rattus norvegicus GN=RGD1564606 PE=3 SV=1
45.90	2	555.33	1108.65	-2.18	61	IAIYELLFK		F1LT36	Protein RGD1564698 OS=Rattus norvegicus GN=RGD1564698 PE=4 SV=2
19.84	2	555.77	1109.53	-1.86	48	STTPDITGYR		F1LST1	Fibronectin OS=Rattus norvegicus GN=Fn1 PE=4 SV=2
15.83	2	555.80	1109.58	-3.73	46	EVGEHVVSVR		D3ZHA0	Protein Flnc OS=Rattus norvegicus GN=Flnc PE=4 SV=1
29.05	2	556.77	1111.52	-2.30	43	MNVFDTELK	[1] Oxidation (M)	E9PSX6	Sarcoplasmic/endoplasmic reticulum calcium ATPase 2 OS=Rattus norvegicus GN=Atp2a2 PE=3 SV=2
29.54	2	557.26	1112.51	-2.22	59	VVFDDTFDR		P14141	Carbonic anhydrase 3 OS=Rattus norvegicus GN=Ca3 PE=1 SV=3
14.86	2	557.79	1113.57	-3.32	52	EQEIAAVQAR		F1M853	Protein Rrbp1 OS=Rattus norvegicus GN=Rrbp1 PE=4 SV=2
33.39	2	557.81	1113.61	-2.23	34	MIAEAIPELK		P04636	Malate dehydrogenase. mitochondrial OS=Rattus norvegicus GN=Mdh2 PE=1 SV=2

13.18	2	558.24	1114.46	-3.85	47	VEFMDDTSR	[4] Oxidation (M)	P62859	40S ribosomal protein S28 OS=Rattus norvegicus GN=Rps28 PE=1 SV=1
24.78	2	558.32	1114.62	-1.62	42	LATTVSAPDLK		Q5M7W5	Microtubule-associated protein 4 OS=Rattus norvegicus GN=Map4 PE=1 SV=1
22.87	2	559.28	1116.55	-2.67	38	VAAAFPGDVDR		B1WBU9	Phosphorylase OS=Rattus norvegicus GN=Pygm PE=2 SV=1
42.03	2	560.33	1118.65	-2.08	40	GVIPFIFQAK		D4A111	Protein Col6a3 OS=Rattus norvegicus GN=Col6a3 PE=4 SV=2
21.76	2	561.32	1120.62	-2.37	33	SVNELIYKR		B0K031	60S ribosomal protein L7 OS=Rattus norvegicus GN=Rpl7 PE=2 SV=1
25.59	2	563.80	1125.59	-1.92	39	STAELYLTTK		Q7TN00	Cardiac titin N2BA isoform (Fragment) OS=Rattus norvegicus PE=2 SV=2
30.78	2	563.85	1125.68	-2.28	54	RVLQALEGLK		D4A6G6	Protein LOC100362339 OS=Rattus norvegicus GN=LOC100362339 PE=4 SV=1
23.68	2	564.31	1126.60	-2.28	76	LDAPSQIEVR		M0RA80	Protein Tnc OS=Rattus norvegicus GN=Tnc PE=4 SV=1
22.00	2	564.34	1126.67	-2.05	85	KLEAAAAALAAK		Q6P3V9	60S ribosomal protein L4 OS=Rattus norvegicus GN=Rpl4 PE=2 SV=1
27.12	2	565.30	1128.59	-1.95	44	NLVDNITGQR		P30427	Plectin OS=Rattus norvegicus GN=Plec PE=1 SV=2
11.69	2	565.33	1128.65	6.82	34	ELQELLKEK		G3V6A8	Golgi autoantigen. golgin subfamily b. macrogolgin 1. isoform CRA_c OS=Rattus norvegicus GN=Golgb1 PE=4 SV=1
24.62	2	565.78	1129.54	-1.56	57	GYSFVTTAER		P68035	Actin. alpha cardiac muscle 1 OS=Rattus norvegicus GN=Actc1 PE=2 SV=1
24.62	2	565.78	1129.54	-1.56	57	GYSFVTTAER		P68136	Actin. alpha skeletal muscle OS=Rattus norvegicus GN=Acta1 PE=1 SV=1
32.06	2	566.31	1130.60	-2.77	61	MEIQEIQLK		P04692	Tropomyosin alpha-1 chain OS=Rattus norvegicus GN=Tpm1 PE=1 SV=3
32.06	2	566.31	1130.60	-2.77	61	MEIQEIQLK		Q6AZ25	Tropomyosin 1. alpha OS=Rattus norvegicus GN=Tpm1 PE=2 SV=1
32.06	2	566.31	1130.60	-2.77	61	MELQEIQLK		Q63610	Tropomyosin alpha-3 chain OS=Rattus norvegicus GN=Tpm3 PE=1 SV=2

32.73	2	566.31	1130.60	-2.56	39	MEIQEIQLK		P04692	Tropomyosin alpha-1 chain OS=Rattus norvegicus GN=Tpm1 PE=1 SV=3
32.73	2	566.31	1130.60	-2.56	39	MEIQEIQLK		Q6AZ25	Tropomyosin 1. alpha OS=Rattus norvegicus GN=Tpm1 PE=2 SV=1
32.73	2	566.31	1130.60	-2.56	39	MELQEIQLK		Q63610	Tropomyosin alpha-3 chain OS=Rattus norvegicus GN=Tpm3 PE=1 SV=2
20.39	2	566.77	1131.52	-1.96	51	GYSFTTTAER		P60711	Actin. cytoplasmic 1 OS=Rattus norvegicus GN=Actb PE=1 SV=1
8.62	2	567.77	1133.53	-3.53	32	AYSEAHEISK		P61983	14-3-3 protein gamma OS=Rattus norvegicus GN=Ywhag PE=1 SV=2
17.86	2	567.78	1133.54	-3.06	38	DVNQQEFVR		D4A6G6	Protein LOC100362339 OS=Rattus norvegicus GN=LOC100362339 PE=4 SV=1
14.70	2	568.82	1135.62	-3.37	48	VKVGNEYVTK		G3V885	Myosin-6 OS=Rattus norvegicus GN=Myh6 PE=4 SV=1
14.70	2	568.82	1135.62	-3.37	48	VKVGNEYVTK		G3V6D8	Myosin-3 OS=Rattus norvegicus GN=Myh3 PE=4 SV=1
14.70	2	568.82	1135.62	-3.37	48	VKVGNEYVTK		F1LMU0	Myosin-4 OS=Rattus norvegicus GN=Myh4 PE=2 SV=1
14.70	2	568.82	1135.62	-3.37	48	VKVGNEYVTK		P02564	Myosin-7 OS=Rattus norvegicus GN=Myh7 PE=2 SV=2
14.70	2	568.82	1135.62	-3.37	48	VKVGNEYVTK		G3V8B0	Myosin-7 OS=Rattus norvegicus GN=Myh7 PE=4 SV=1
14.70	2	568.82	1135.62	-3.37	48	VKVGNEYVTK		F1M8F6	Myosin-8 (Fragment) OS=Rattus norvegicus GN=Myh8 PE=4 SV=2
14.70	2	568.82	1135.62	-3.37	48	VKVGNEYVTK		F1LRV9	Protein Myh1 OS=Rattus norvegicus GN=Myh1 PE=2 SV=2
14.70	2	568.82	1135.62	-3.37	48	VKVGNEYVTK		F1M789	Protein Myh13 OS=Rattus norvegicus GN=Myh13 PE=4 SV=2
14.70	2	568.82	1135.62	-3.37	48	VKVGNEYVTK		G3V6E1	Uncharacterized protein OS=Rattus norvegicus GN=Myh2 PE=4 SV=2
19.52	2	570.27	1138.53	-2.41	44	YYPTEDVPR		F1LQS3	60S ribosomal protein L6 OS=Rattus norvegicus GN=Rpl6 PE=3 SV=1
7.72	2	570.81	1139.62	-2.90	64	EEKPAVTAAPK		P23928	Alpha-crystallin B chain OS=Rattus norvegicus GN=Cryab PE=1 SV=1
31.97	2	572.83	1143.64	-2.24	51	AVPQLQGYLR		F1LQS3	60S ribosomal protein L6 OS=Rattus norvegicus GN=Rpl6 PE=3 SV=1

31.97	2	572.83	1143.64	-2.24	51	AVPQLQGYLR		F1M2R6	Uncharacterized protein (Fragment) OS=Rattus norvegicus PE=4 SV=1
18.79	2	573.27	1144.53	-2.81	46	NLQTVNVDEN		D4ABZ9	Protein Rpl31-ps8 (Fragment) OS=Rattus norvegicus GN=Rpl31l4 PE=4 SV=2
11.39	2	573.79	1145.56	-2.93	30	LQTENGELSR		P02564	Myosin-7 OS=Rattus norvegicus GN=Myh7 PE=2 SV=2
11.39	2	573.79	1145.56	-2.93	30	LQTENGELSR		G3V8B0	Myosin-7 OS=Rattus norvegicus GN=Myh7 PE=4 SV=1
27.12	2	575.31	1148.61	-1.87	52	LVQEVTDFAK		P02770	Serum albumin OS=Rattus norvegicus GN=Alb PE=1 SV=2
20.23	2	575.84	1149.66	-1.56	75	VLIAAHGNSLR		P16290	Phosphoglycerate mutase 2 OS=Rattus norvegicus GN=Pgam2 PE=2 SV=2
23.52	2	576.28	1150.54	-2.22	41	FTNIGPDTMR		F1LST1	Fibronectin OS=Rattus norvegicus GN=Fn1 PE=4 SV=2
35.27	2	576.84	1151.66	-2.58	37	NGVAPIIDVVR		D3ZF07	Protein RGD1562402 OS=Rattus norvegicus GN=RGD1562402 PE=3 SV=1
17.24	2	577.82	1153.64	-3.12	28	ARAHSIQIMK		P62718	60S ribosomal protein L18a OS=Rattus norvegicus GN=Rpl18a PE=2 SV=1
8.02	2	578.27	1154.53	-3.33	61	EAAEGLGSHER		Q07009	Calpain-2 catalytic subunit OS=Rattus norvegicus GN=Capn2 PE=1 SV=3
9.00	2	578.80	1155.59	-2.58	30	NGVRPSHFSR		D4A6G6	Protein LOC100362339 OS=Rattus norvegicus GN=LOC100362339 PE=4 SV=1
26.05	2	581.31	1160.61	-1.59	50	EITALAPSTMK		P60711	Actin. cytoplasmic 1 OS=Rattus norvegicus GN=Actb PE=1 SV=1
26.05	2	581.31	1160.61	-1.59	50	EITALAPSTMK		P68035	Actin. alpha cardiac muscle 1 OS=Rattus norvegicus GN=Actc1 PE=2 SV=1
26.05	2	581.31	1160.61	-1.59	50	EITALAPSTMK		P68136	Actin. alpha skeletal muscle OS=Rattus norvegicus GN=Acta1 PE=1 SV=1
25.67	2	583.33	1164.65	-2.18	66	VLGDVIEVHGK		P23928	Alpha-crystallin B chain OS=Rattus norvegicus GN=Cryab PE=1 SV=1
17.01	2	584.28	1166.54	-2.46	41	FTNIGPDTMR	[9] Oxidation (M)	F1LST1	Fibronectin OS=Rattus norvegicus GN=Fn1 PE=4 SV=2
8.27	2	584.80	1167.59	-2.55	41	QVHPDTGISSK		G3V8B3	Histone H2B OS=Rattus norvegicus GN=LOC684797 PE=3 SV=1

32.73	2	585.80	1169.59	-1.85	46	FLDGIYVSEK		Q6P9U5	Ribosomal protein L9 OS=Rattus norvegicus GN=Rpl9 PE=2 SV=1
15.99	2	586.29	1170.56	-2.73	60	HQGVMVGMGQK		P68136	Actin. alpha skeletal muscle OS=Rattus norvegicus GN=Acta1 PE=1 SV=1
15.99	2	586.29	1170.56	-2.73	60	HQGVMVGMGQK		P68035	Actin. alpha cardiac muscle 1 OS=Rattus norvegicus GN=Actc1 PE=2 SV=1
15.99	2	586.29	1170.56	-2.73	60	HQGVMVGMGQK		P60711	Actin. cytoplasmic 1 OS=Rattus norvegicus GN=Actb PE=1 SV=1
15.99	2	586.29	1170.56	-2.73	60	HQGVMVGMGQK		D3ZRN3	Protein Actbl2 OS=Rattus norvegicus GN=Actbl2 PE=3 SV=1
27.69	2	586.32	1170.62	-2.58	38	LDIDSAPITAR		P11980	Pyruvate kinase PKM OS=Rattus norvegicus GN=Pkm PE=1 SV=3
27.69	2	586.32	1170.62	-2.58	38	LDIDSAPITAR		D4ADU8	Pyruvate kinase OS=Rattus norvegicus PE=3 SV=2
13.02	2	586.32	1170.63	-3.54	37	LVEIDNGKQR		G3V8L3	Lamin A. isoform CRA_b OS=Rattus norvegicus GN=Lmna PE=3 SV=1
25.43	2	586.83	1171.64	-2.46	35	GGPLSGPYRLR		P14141	Carbonic anhydrase 3 OS=Rattus norvegicus GN=Ca3 PE=1 SV=3
29.38	2	586.83	1171.65	-2.12	75	IALTDNSLVAR		B0K031	60S ribosomal protein L7 OS=Rattus norvegicus GN=Rpl7 PE=2 SV=1
17.24	2	587.32	1172.63	-2.57	61	EYTINIHKR		D4ABZ9	Protein Rpl31-ps8 (Fragment) OS=Rattus norvegicus GN=Rpl31l4 PE=4 SV=2
11.15	2	587.80	1173.60	-3.08	63	VPGDQTSTTIR		M0RA80	Protein Tnc OS=Rattus norvegicus GN=Tnc PE=4 SV=1
17.78	2	588.73	1175.45	-2.63	33	GYFDEEMNR	[7] Oxidation (M)	P30427	Plectin OS=Rattus norvegicus GN=Plec PE=1 SV=2
6.58	2	589.31	1176.60	-0.39	44	EAHKSEIAHR		P02770	Serum albumin OS=Rattus norvegicus GN=Alb PE=1 SV=2
19.91	2	589.31	1176.60	-0.95	72	EITALAPSTMK	[10] Oxidation (M)	P60711	Actin. cytoplasmic 1 OS=Rattus norvegicus GN=Actb PE=1 SV=1
19.91	2	589.31	1176.60	-0.95	72	EITALAPSTMK	[10] Oxidation (M)	P68035	Actin. alpha cardiac muscle 1 OS=Rattus norvegicus GN=Actc1 PE=2 SV=1
19.91	2	589.31	1176.60	-0.95	72	EITALAPSTMK	[10] Oxidation (M)	P68136	Actin. alpha skeletal muscle OS=Rattus norvegicus GN=Acta1 PE=1 SV=1

16.85	2	590.76	1179.50	-2.28	83	NVEAMSGMEGR		F1LMC6	Troponin I. slow skeletal muscle (Fragment) OS=Rattus norvegicus GN=Tnni1 PE=4 SV=1
10.00	2	590.81	1179.61	-3.01	42	VQVEYKGETK		P63018	Heat shock cognate 71 kDa protein OS=Rattus norvegicus GN=Hspa8 PE=1 SV=1
10.00	2	590.81	1179.61	-3.01	42	VQVEYKGETK		D3ZH98	Uncharacterized protein OS=Rattus norvegicus PE=3 SV=1
27.92	2	590.81	1179.61	-1.98	50	ISGLIYEETR		P62804	Histone H4 OS=Rattus norvegicus GN=Hist1h4b PE=1 SV=2
15.18	2	591.28	1180.54	-2.36	54	MELQEMQLK	[1] Oxidation (M)|[6] Oxidation (M)	Q5FVG5	Similar to tropomyosin 1. embryonic fibroblast-rat. isoform CRA_c OS=Rattus norvegicus GN=Tpm2 PE=2 SV=1
15.18	2	591.28	1180.54	-2.36	54	MEIQEMQLK	[1] Oxidation (M)|[6] Oxidation (M)	P09495	Tropomyosin alpha-4 chain OS=Rattus norvegicus GN=Tpm4 PE=1 SV=3
15.83	2	593.28	1184.54	-3.18	46	RFPGYDSESK		P09895	60S ribosomal protein L5 OS=Rattus norvegicus GN=Rpl5 PE=1 SV=3
11.54	2	594.28	1186.56	-3.00	66	HQGVMVGMGQK	[5] Oxidation (M)	P68136	Actin. alpha skeletal muscle OS=Rattus norvegicus GN=Acta1 PE=1 SV=1
11.54	2	594.28	1186.56	-3.00	66	HQGVMVGMGQK	[5] Oxidation (M)	P68035	Actin. alpha cardiac muscle 1 OS=Rattus norvegicus GN=Actc1 PE=2 SV=1
11.54	2	594.28	1186.56	-3.00	66	HQGVMVGMGQK	[5] Oxidation (M)	P60711	Actin. cytoplasmic 1 OS=Rattus norvegicus GN=Actb PE=1 SV=1
11.54	2	594.28	1186.56	-3.00	66	HQGVMVGMGQK	[5] Oxidation (M)	D3ZRN3	Protein Actbl2 OS=Rattus norvegicus GN=Actbl2 PE=3 SV=1
8.92	2	594.29	1186.56	-2.56	42	HQGVMVGMGQK	[8] Oxidation (M)	P68136	Actin. alpha skeletal muscle OS=Rattus norvegicus GN=Acta1 PE=1 SV=1
8.92	2	594.29	1186.56	-2.56	42	HQGVMVGMGQK	[8] Oxidation (M)	P68035	Actin. alpha cardiac muscle 1 OS=Rattus norvegicus GN=Actc1 PE=2 SV=1
8.92	2	594.29	1186.56	-2.56	42	HQGVMVGMGQK	[8] Oxidation (M)	P60711	Actin. cytoplasmic 1 OS=Rattus norvegicus GN=Actb PE=1 SV=1
8.92	2	594.29	1186.56	-2.56	42	HQGVMVGMGQK	[8] Oxidation (M)	D3ZRN3	Protein Actbl2 OS=Rattus norvegicus GN=Actbl2 PE=3 SV=1
25.67	2	594.82	1187.63	-2.53	36	GPGLSQAFVGQK		D3ZHA0	Protein Flnc OS=Rattus norvegicus GN=Flnc PE=4 SV=1

12.63	2	594.83	1187.64	-3.93	47	GAFGKPQGTVAR		Q6PDV7	60S ribosomal protein L10 OS=Rattus norvegicus GN=Rpl10 PE=1 SV=3
18.17	2	596.78	1191.55	-2.60	33	DETNYGIPQR		P63245	Guanine nucleotide-binding protein subunit beta-2-like 1 OS=Rattus norvegicus GN=Gnb2l1 PE=1 SV=3
23.04	2	596.80	1191.59	-2.18	46	DGFIDKNDLR		P08733	Myosin regulatory light chain 2. ventricular/cardiac muscle isoform OS=Rattus norvegicus GN=Myl2 PE=1 SV=2
29.70	2	597.83	1193.64	-2.04	38	IDIIPNPQER		P34058	Heat shock protein HSP 90-beta OS=Rattus norvegicus GN=Hsp90ab1 PE=1 SV=4
16.14	2	598.80	1195.59	-3.40	28	ALPAQDPPMEK		B5DFG4	Heat shock 27kD protein family. member 7 (Cardiovascular) OS=Rattus norvegicus GN=Hspb7 PE=2 SV=1
21.53	2	599.36	1196.71	-0.74	33	LSKDPNIVIAK		P11598	Protein disulfide-isomerase A3 OS=Rattus norvegicus GN=Pdia3 PE=1 SV=2
7.80	2	599.76	1197.51	-2.66	74	DSYVGDEAQSK		P68035	Actin. alpha cardiac muscle 1 OS=Rattus norvegicus GN=Actc1 PE=2 SV=1
7.80	2	599.76	1197.51	-2.66	74	DSYVGDEAQSK		P68136	Actin. alpha skeletal muscle OS=Rattus norvegicus GN=Acta1 PE=1 SV=1
7.80	2	599.76	1197.51	-2.66	74	DSYVGDEAQSK		P60711	Actin. cytoplasmic 1 OS=Rattus norvegicus GN=Actb PE=1 SV=1
9.96	2	599.77	1197.53	-3.11	68	MFDAAKSPTSQ	[1] Oxidation (M)	F1LMC6	Troponin I. slow skeletal muscle (Fragment) OS=Rattus norvegicus GN=Tnni1 PE=4 SV=1
28.89	2	599.84	1197.66	-1.75	75	ASGPPVSELITK		P15865	Histone H1.4 OS=Rattus norvegicus GN=Hist1h1e PE=1 SV=3
28.89	2	599.84	1197.66	-1.75	75	ASGPPVSELITK		M0R7B4	Protein LOC684828 OS=Rattus norvegicus GN=LOC684828 PE=3 SV=1
30.19	3	400.24	1197.70	-1.32	32	AVFPSIVGRPR		P68035	Actin. alpha cardiac muscle 1 OS=Rattus norvegicus GN=Actc1 PE=2 SV=1
30.19	3	400.24	1197.70	-1.32	32	AVFPSIVGRPR		P68136	Actin. alpha skeletal muscle OS=Rattus norvegicus GN=Acta1 PE=1 SV=1
30.19	3	400.24	1197.70	-1.32	32	AVFPSIVGRPR		P60711	Actin. cytoplasmic 1 OS=Rattus norvegicus GN=Actb PE=1 SV=1

30.11	2	599.86	1197.70	-1.18	30	AVFPSIVGRPR		P68035	Actin. alpha cardiac muscle 1 OS=Rattus norvegicus GN=Actc1 PE=2 SV=1
30.11	2	599.86	1197.70	-1.18	30	AVFPSIVGRPR		P68136	Actin. alpha skeletal muscle OS=Rattus norvegicus GN=Acta1 PE=1 SV=1
30.11	2	599.86	1197.70	-1.18	30	AVFPSIVGRPR		P60711	Actin. cytoplasmic 1 OS=Rattus norvegicus GN=Actb PE=1 SV=1
35.51	2	600.34	1198.67	-1.39	47	DAGTIAGLNVLR		P63018	Heat shock cognate 71 kDa protein OS=Rattus norvegicus GN=Hspa8 PE=1 SV=1
47.23	2	601.33	1200.65	-2.96	41	AGLLGLLEEMR		G3V885	Myosin-6 OS=Rattus norvegicus GN=Myh6 PE=4 SV=1
47.23	2	601.33	1200.65	-2.96	41	AGLLGLLEEMR		P02564	Myosin-7 OS=Rattus norvegicus GN=Myh7 PE=2 SV=2
47.23	2	601.33	1200.65	-2.96	41	AGLLGLLEEMR		G3V8B0	Myosin-7 OS=Rattus norvegicus GN=Myh7 PE=4 SV=1
47.23	2	601.33	1200.65	-2.96	41	AGLLGLLEEMR		F1M8F6	Myosin-8 (Fragment) OS=Rattus norvegicus GN=Myh8 PE=4 SV=2
47.23	2	601.33	1200.65	-2.96	41	AGLLGLLEEMR		F1LRV9	Protein Myh1 OS=Rattus norvegicus GN=Myh1 PE=2 SV=2
47.23	2	601.33	1200.65	-2.96	41	AGLLGLLEEMR		F1M789	Protein Myh13 OS=Rattus norvegicus GN=Myh13 PE=4 SV=2
47.23	2	601.33	1200.65	-2.96	41	AGLLGLLEEMR		G3V6E1	Uncharacterized protein OS=Rattus norvegicus GN=Myh2 PE=4 SV=2
7.13	2	602.28	1202.55	-2.59	42	HQGVMVGMGQK	[5] Oxidation (M)|[8] Oxidation (M)	P68136	Actin. alpha skeletal muscle OS=Rattus norvegicus GN=Acta1 PE=1 SV=1
7.13	2	602.28	1202.55	-2.59	42	HQGVMVGMGQK	[5] Oxidation (M)|[8] Oxidation (M)	P68035	Actin. alpha cardiac muscle 1 OS=Rattus norvegicus GN=Actc1 PE=2 SV=1
7.13	2	602.28	1202.55	-2.59	42	HQGVMVGMGQK	[5] Oxidation (M)|[8] Oxidation (M)	P60711	Actin. cytoplasmic 1 OS=Rattus norvegicus GN=Actb PE=1 SV=1
7.13	2	602.28	1202.55	-2.59	42	HQGVMVGMGQK	[5] Oxidation (M)|[8] Oxidation (M)	D3ZRN3	Protein Actbl2 OS=Rattus norvegicus GN=Actbl2 PE=3 SV=1

19.28	2	602.33	1202.64	-2.70	76	FAAATGATPIAGR		P38983	40S ribosomal protein SA OS=Rattus norvegicus GN=Rpsa PE=1 SV=3
18.02	2	603.34	1204.66	-3.04	37	KGHAVGDIPGVR		P62268	40S ribosomal protein S23 OS=Rattus norvegicus GN=Rps23 PE=1 SV=3
15.18	2	604.81	1207.61	-1.71	47	AITEEEKNFK		P41123	60S ribosomal protein L13 OS=Rattus norvegicus GN=Rpl13 PE=1 SV=2
15.18	2	604.81	1207.61	-1.71	47	AITEEEKNFK		D3ZD02	60S ribosomal protein L13 OS=Rattus norvegicus GN=RGD1563145 PE=3 SV=1
17.70	2	605.38	1208.75	15.00	25	KVKPVPWVEK		B4F778	Protein Rfc4 OS=Rattus norvegicus GN=Rfc4 PE=2 SV=1
6.58	2	606.75	1211.49	-2.65	51	NVEAMSGMEGR	[5] Oxidation (M)|[8] Oxidation (M)	F1LMC6	Troponin I. slow skeletal muscle (Fragment) OS=Rattus norvegicus GN=Tnni1 PE=4 SV=1
26.13	2	606.80	1211.59	-1.77	38	IHIEGEPDFR		D3ZAF5	Periostin. osteoblast specific factor (Predicted). isoform CRA_a OS=Rattus norvegicus GN=Postn PE=4 SV=1
29.38	2	606.84	1211.67	-2.97	45	ATGPPVSELITK		D3ZBN0	Histone H1.5 OS=Rattus norvegicus GN=Hist1h1b PE=3 SV=1
21.53	2	607.31	1212.61	-2.30	28	VTQSNFAVGYK		D3Z9A9	Uncharacterized protein (Fragment) OS=Rattus norvegicus PE=4 SV=2
15.02	2	607.82	1213.63	-2.37	41	ALGTNPTNAEVK		P02600	Myosin light chain 1/3. skeletal muscle isoform OS=Rattus norvegicus GN=Myl1 PE=1 SV=2
22.65	2	608.33	1214.64	-2.95	51	TVDGPSGKLWR		M0R590	Protein LOC685186 OS=Rattus norvegicus GN=LOC685186 PE=3 SV=1
18.88	3	405.89	1214.65	-2.63	28	GIPHLVTHDAR		P62703	40S ribosomal protein S4. X isoform OS=Rattus norvegicus GN=Rps4x PE=2 SV=2
18.88	2	608.33	1214.65	-2.14	41	GIPHLVTHDAR		P62703	40S ribosomal protein S4. X isoform OS=Rattus norvegicus GN=Rps4x PE=2 SV=2
27.84	2	608.82	1215.62	-1.79	40	NFGIGQDIQPK		D3ZPL5	Protein LOC100361311 OS=Rattus norvegicus GN=RGD1562953 PE=4 SV=1
32.48	2	609.32	1216.62	-2.04	42	DAGTIAGLNVMR		P06761	78 kDa glucose-regulated protein OS=Rattus norvegicus GN=Hspa5 PE=1 SV=1
41.34	2	609.33	1216.65	-1.69	52	AGLLGLLEEMR	[10] Oxidation (M)	G3V885	Myosin-6 OS=Rattus norvegicus GN=Myh6 PE=4 SV=1

41.34	2	609.33	1216.65	-1.69	52	AGLLGLLEEMR	[10] Oxidation (M)	P02564	Myosin-7 OS=Rattus norvegicus GN=Myh7 PE=2 SV=2
41.34	2	609.33	1216.65	-1.69	52	AGLLGLLEEMR	[10] Oxidation (M)	G3V8B0	Myosin-7 OS=Rattus norvegicus GN=Myh7 PE=4 SV=1
41.34	2	609.33	1216.65	-1.69	52	AGLLGLLEEMR	[10] Oxidation (M)	F1M8F6	Myosin-8 (Fragment) OS=Rattus norvegicus GN=Myh8 PE=4 SV=2
41.34	2	609.33	1216.65	-1.69	52	AGLLGLLEEMR	[10] Oxidation (M)	F1LRV9	Protein Myh1 OS=Rattus norvegicus GN=Myh1 PE=2 SV=2
41.34	2	609.33	1216.65	-1.69	52	AGLLGLLEEMR	[10] Oxidation (M)	F1M789	Protein Myh13 OS=Rattus norvegicus GN=Myh13 PE=4 SV=2
41.34	2	609.33	1216.65	-1.69	52	AGLLGLLEEMR	[10] Oxidation (M)	G3V6E1	Uncharacterized protein OS=Rattus norvegicus GN=Myh2 PE=4 SV=2
25.10	2	609.80	1217.59	-2.35	62	MVPTGMGAGLER		F1LV13	Heterogeneous nuclear ribonucleoprotein M OS=Rattus norvegicus GN=Hnrnpm PE=2 SV=1
14.63	2	610.32	1218.63	-2.93	41	YELGRPAANTK		B2RYR8	40S ribosomal protein S8 OS=Rattus norvegicus GN=Rps8 PE=2 SV=1
12.55	2	611.31	1220.61	-3.40	38	GHLENNPALEK		P19945	60S acidic ribosomal protein P0 OS=Rattus norvegicus GN=Rplp0 PE=1 SV=2
19.91	2	612.83	1223.65	-2.87	47	GVVEVTHDLQK		Q5RJR9	Serine (Or cysteine) proteinase inhibitor. clade H. member 1. isoform CRA_b OS=Rattus norvegicus GN=Serpinh1 PE=2 SV=1
21.20	2	613.29	1224.56	-1.76	48	EATTEFSVDAR		C0JPT7	Filamin alpha OS=Rattus norvegicus GN=Flna PE=2 SV=1
11.31	2	613.29	1224.57	-3.04	54	NNQIDHIDEK		P51886	Lumican OS=Rattus norvegicus GN=Lum PE=2 SV=1
7.04	2	613.33	1224.64	-3.11	45	KAPTTAAPPSER		M0R9L0	Protein Naca OS=Rattus norvegicus GN=Naca PE=4 SV=1
33.31	2	615.35	1228.69	-1.11	39	VRMNVLADALK		D3ZLL8	Protein LOC100909878 OS=Rattus norvegicus GN=Rps15al4 PE=3 SV=1
13.93	2	616.81	1231.61	-2.85	44	STESLQANVQR		P41123	60S ribosomal protein L13 OS=Rattus norvegicus GN=Rpl13 PE=1 SV=2
13.93	2	616.81	1231.61	-2.85	44	STESLQANVQR		D3ZD02	60S ribosomal protein L13 OS=Rattus norvegicus GN=RGD1563145 PE=3 SV=1

26.13	2	617.32	1232.62	-1.51	56	DAGTIAGLNVMR	[11] Oxidation (M)	P06761	78 kDa glucose-regulated protein OS=Rattus norvegicus GN=Hspa5 PE=1 SV=1
31.29	2	618.36	1234.71	-1.72	27	FVNVVPTFGKK		P62864	40S ribosomal protein S30 OS=Rattus norvegicus GN=Fau PE=1 SV=1
31.97	2	618.84	1235.67	-1.88	55	QMVIDVLHPGK		D4ACJ1	40S ribosomal protein S24 OS=Rattus norvegicus GN=LOC100363469 PE=3 SV=1
24.78	2	618.85	1235.69	-1.93	44	TTTGNKVFGALK		P09895	60S ribosomal protein L5 OS=Rattus norvegicus GN=Rpl5 PE=1 SV=3
24.78	2	618.85	1235.69	-1.93	44	TTTGNKVFGALK		D3ZHP8	Protein Rpl5l1 OS=Rattus norvegicus GN=Rpl5l1 PE=3 SV=1
18.95	2	620.32	1238.62	-2.26	36	SVEMLKEMIK	[4] Oxidation (M)|[8] Oxidation (M)	P11980	Pyruvate kinase PKM OS=Rattus norvegicus GN=Pkm PE=1 SV=3
18.95	2	620.32	1238.62	-2.26	36	SVEMLKEMIK	[4] Oxidation (M)|[8] Oxidation (M)	D4ADU8	Pyruvate kinase OS=Rattus norvegicus PE=3 SV=2
20.23	2	620.78	1239.55	-2.45	47	EAFTIMDQNR	[6] Oxidation (M)	P08733	Myosin regulatory light chain 2. ventricular/cardiac muscle isoform OS=Rattus norvegicus GN=Myl2 PE=1 SV=2
29.05	2	620.82	1239.63	-2.15	65	AEGPEVDVSLPK		Q38PG0	AHNAK 1 (Fragment) OS=Rattus norvegicus PE=2 SV=1
33.31	2	621.84	1241.66	-1.81	75	NIEDVIAQGVGK		D4A4D5	Protein LOC100362751 OS=Rattus norvegicus GN=LOC498555 PE=3 SV=1
33.31	2	621.84	1241.66	-1.81	75	NIEDVIAQGVGK		P02401	60S acidic ribosomal protein P2 OS=Rattus norvegicus GN=Rplp2 PE=1 SV=2
20.56	2	621.84	1241.67	-3.16	26	QVQVALETAQR		P30427	Plectin OS=Rattus norvegicus GN=Plec PE=1 SV=2
26.29	2	621.84	1241.67	-1.62	76	AGNLGGGVVTIER		Q6PDV8	RCG31311 OS=Rattus norvegicus GN=LOC100363800 PE=2 SV=1
34.50	2	621.86	1241.70	-1.89	32	ADLINNLGTIAK		P82995	Heat shock protein HSP 90-alpha OS=Rattus norvegicus GN=Hsp90aa1 PE=1 SV=3
34.50	2	621.86	1241.70	-1.89	32	ADLINNLGTIAK		P34058	Heat shock protein HSP 90-beta OS=Rattus norvegicus GN=Hsp90ab1 PE=1 SV=4

34.50	2	621.86	1241.70	-1.89	32	ADLINNLGTIAK		F1LYW7	Uncharacterized protein (Fragment) OS=Rattus norvegicus PE=3 SV=2
30.78	2	622.33	1242.65	-7.37	50	HGVVPLATYMR		D3ZPN7	Protein LOC100360604 OS=Rattus norvegicus GN=LOC100364176 PE=4 SV=1
30.78	2	622.33	1242.65	-0.37	58	IQLVEEELDR		P04692	Tropomyosin alpha-1 chain OS=Rattus norvegicus GN=Tpm1 PE=1 SV=3
30.78	2	622.33	1242.65	-7.37	50	HGVVPLATYMR		D3ZEK2	Protein RGD1562923 OS=Rattus norvegicus GN=RGD1562923 PE=4 SV=1
30.78	2	622.33	1242.65	-0.37	58	IQLVEEELDR		Q5FVG5	Similar to tropomyosin 1. embryonic fibroblast-rat. isoform CRA_c OS=Rattus norvegicus GN=Tpm2 PE=2 SV=1
30.78	2	622.33	1242.65	-0.37	58	IQLVEEELDR		Q6AZ25	Tropomyosin 1. alpha OS=Rattus norvegicus GN=Tpm1 PE=2 SV=1
30.78	2	622.33	1242.65	-0.37	58	IQLVEEELDR		Q63610	Tropomyosin alpha-3 chain OS=Rattus norvegicus GN=Tpm3 PE=1 SV=2
30.78	2	622.33	1242.65	-0.37	58	IQLVEEELDR		P09495	Tropomyosin alpha-4 chain OS=Rattus norvegicus GN=Tpm4 PE=1 SV=3
26.00	2	622.33	1242.65	-1.67	43	SLQDLQLANNK		P51886	Lumican OS=Rattus norvegicus GN=Lum PE=2 SV=1
20.96	2	622.34	1242.67	-1.59	60	GPLPAAPPAAPER		F1LQN3	Reticulon OS=Rattus norvegicus GN=Rtn4 PE=2 SV=1
28.25	2	623.35	1244.69	-2.22	38	VRMNVLADALK	[3] Oxidation (M)	D3ZLL8	Protein LOC100909878 OS=Rattus norvegicus GN=Rps15al4 PE=3 SV=1
16.06	2	624.29	1246.56	-2.45	77	AGELTEDEVER		D3ZAU6	Protein RGD1561919 OS=Rattus norvegicus GN=RGD1561919 PE=3 SV=1
16.06	2	624.29	1246.56	-2.45	77	AGELTEDEVER		D3ZII2	Protein RGD1562404 OS=Rattus norvegicus GN=RGD1562404 PE=3 SV=1
18.10	2	624.82	1247.63	-3.42	33	FKDLGEQHFK		P02770	Serum albumin OS=Rattus norvegicus GN=Alb PE=1 SV=2
13.78	2	625.29	1248.57	-2.47	52	EGNGTVMGAEIR	[7] Oxidation (M)	B2GV99	Myl6 protein OS=Rattus norvegicus GN=Myl6 PE=2 SV=1
13.78	2	625.29	1248.57	-2.47	52	EGNGTVMGAELR	[7] Oxidation (M)	P02600	Myosin light chain 1/3. skeletal muscle isoform OS=Rattus norvegicus GN=Myl1 PE=1 SV=2

13.78	2	625.29	1248.57	-2.47	52	EGNGTVMGAELR	[7] Oxidation (M)	P16409	Myosin light chain 3 OS=Rattus norvegicus GN=Myl3 PE=2 SV=2
18.57	2	625.31	1248.61	-2.14	39	EQVANSAFVER		P34058	Heat shock protein HSP 90-beta OS=Rattus norvegicus GN=Hsp90ab1 PE=1 SV=4
22.41	2	626.82	1251.63	-1.80	26	YQAVTATLEEK		Q5RK10	60S ribosomal protein L13a OS=Rattus norvegicus GN=Rpl13a PE=2 SV=1
34.67	2	626.86	1251.71	-2.38	31	VHIGQVIMSIR		Q6PDV7	60S ribosomal protein L10 OS=Rattus norvegicus GN=Rpl10 PE=1 SV=3
8.78	2	627.82	1253.63	-3.05	41	LSYNTASNKTR		F1LQ14	60S ribosomal protein L34 OS=Rattus norvegicus GN=Rpl34 PE=2 SV=1
24.78	2	629.31	1256.60	-2.43	42	DETEFYLGKR		D4A771	Protein LOC100362049 OS=Rattus norvegicus GN=LOC100362049 PE=4 SV=1
29.14	2	629.33	1256.65	-1.52	53	ASAPLPGFSTPGR		P97541	Heat shock protein beta-6 OS=Rattus norvegicus GN=Hspb6 PE=1 SV=1
28.16	2	629.79	1257.56	-1.75	50	GTVTDFSGFDGR		Q66HH8	Annexin OS=Rattus norvegicus GN=Anxa5 PE=2 SV=1
30.19	2	629.83	1257.65	-2.28	39	FTTKRPNTFF		P62718	60S ribosomal protein L18a OS=Rattus norvegicus GN=Rpl18a PE=2 SV=1
31.21	2	629.85	1257.68	9.10	52	AFGPGLEGGLVNK		D3ZHA0	Protein Flnc OS=Rattus norvegicus GN=Flnc PE=4 SV=1
31.21	2	629.85	1257.68	-10.94	30	VATYLPAPEGLK		M0RA80	Protein Tnc OS=Rattus norvegicus GN=Tnc PE=4 SV=1
22.95	2	630.30	1258.59	-1.76	26	VVDLMAYMASK	[5] Oxidation (M)|[8] Oxidation (M)	M0R590	Protein LOC685186 OS=Rattus norvegicus GN=LOC685186 PE=3 SV=1
22.95	2	630.30	1258.59	-1.76	26	VVDLMAYMASK	[5] Oxidation (M)|[8] Oxidation (M)	M0R451	Uncharacterized protein OS=Rattus norvegicus PE=3 SV=1
22.95	2	630.30	1258.59	-1.76	26	VVDLMAYMASK	[5] Oxidation (M)|[8] Oxidation (M)	D3ZIY0	Uncharacterized protein OS=Rattus norvegicus PE=3 SV=1
23.12	2	630.33	1258.65	-0.52	31	HGVVPLATYMR	[10] Oxidation (M)	D3ZPN7	Protein LOC100360604 OS=Rattus norvegicus GN=LOC100364176 PE=4 SV=1
23.12	2	630.33	1258.65	-5.68	34	DAFAGKLPEPSK		Q9ER30	Kelch-like protein 41 OS=Rattus norvegicus GN=Klhl41 PE=1 SV=1

23.12	2	630.33	1258.65	-0.52	31	HGVVPLATYMR	[10] Oxidation (M)	D3ZEK2	Protein RGD1562923 OS=Rattus norvegicus GN=RGD1562923 PE=4 SV=1
20.15	2	630.88	1259.74	-2.43	64	SLVSKGTLVQTK		P15865	Histone H1.4 OS=Rattus norvegicus GN=Hist1h1e PE=1 SV=3
20.15	2	630.88	1259.74	-2.43	64	SLVSKGTLVQTK		D3ZBN0	Histone H1.5 OS=Rattus norvegicus GN=Hist1h1b PE=3 SV=1
36.32	2	631.34	1260.67	-1.86	85	AITGASLADIMAK		P83732	60S ribosomal protein L24 OS=Rattus norvegicus GN=Rpl24 PE=2 SV=1
24.94	2	632.31	1262.60	5.29	34	EEAENNLAAFR		Q6P725	Desmin OS=Rattus norvegicus GN=Des PE=2 SV=1
32.73	2	633.82	1265.63	-1.68	55	FPNAEFAEITK		P02770	Serum albumin OS=Rattus norvegicus GN=Alb PE=1 SV=2
17.01	2	633.83	1265.65	-2.69	40	EAIEGTYIDKK		Q6PDV9	Ribosomal protein S11 OS=Rattus norvegicus GN=Rps11 PE=2 SV=1
21.36	2	634.83	1267.65	-1.92	61	MKEIAEAYLGK	[1] Oxidation (M)	P63018	Heat shock cognate 71 kDa protein OS=Rattus norvegicus GN=Hspa8 PE=1 SV=1
31.65	2	634.86	1267.71	-0.46	50	VHIGQVIMSIR	[8] Oxidation (M)	Q6PDV7	60S ribosomal protein L10 OS=Rattus norvegicus GN=Rpl10 PE=1 SV=3
7.04	3	423.58	1267.71	-3.26	27	EEKPAVTAAPKK		P23928	Alpha-crystallin B chain OS=Rattus norvegicus GN=Cryab PE=1 SV=1
7.04	2	634.86	1267.71	-2.92	52	EEKPAVTAAPKK		P23928	Alpha-crystallin B chain OS=Rattus norvegicus GN=Cryab PE=1 SV=1
12.00	2	635.34	1268.66	-2.64	34	QEAIPEDVIQK		P35559	Insulin-degrading enzyme OS=Rattus norvegicus GN=Ide PE=1 SV=1
15.02	2	635.86	1269.70	-2.58	40	INATLETKQPR		G3V885	Myosin-6 OS=Rattus norvegicus GN=Myh6 PE=4 SV=1
15.02	2	635.86	1269.70	-2.58	40	INATLETKQPR		P02564	Myosin-7 OS=Rattus norvegicus GN=Myh7 PE=2 SV=2
15.02	2	635.86	1269.70	-2.58	40	INATLETKQPR		G3V8B0	Myosin-7 OS=Rattus norvegicus GN=Myh7 PE=4 SV=1
25.51	2	636.36	1270.70	-1.91	49	VGGVQSLGGTGALR		P13221	Aspartate aminotransferase. cytoplasmic OS=Rattus norvegicus GN=Got1 PE=1 SV=3
13.40	2	636.78	1271.56	-1.05	41	TLEDQMNEHR		P02564	Myosin-7 OS=Rattus norvegicus GN=Myh7 PE=2 SV=2
13.40	2	636.78	1271.56	-1.05	41	TLEDQMNEHR		G3V8B0	Myosin-7 OS=Rattus norvegicus GN=Myh7 PE=4 SV=1
38.67	2	637.87	1273.72	-1.56	32	LLVVYPWTQR		P02091	Hemoglobin subunit beta-1 OS=Rattus norvegicus GN=Hbb PE=1 SV=3

13.93	2	638.83	1275.64	-2.31	67	INENTGSVSVTR		F1M7X3	Protein Cdh13 (Fragment) OS=Rattus norvegicus GN=Cdh13 PE=4 SV=2
18.63	2	638.84	1275.66	-2.22	33	GVLTSTVNSSSPK		M0R9L0	Protein Naca OS=Rattus norvegicus GN=Naca PE=4 SV=1
29.14	2	639.34	1276.67	-1.44	86	AITGASLADIMAK	[11] Oxidation (M)	P83732	60S ribosomal protein L24 OS=Rattus norvegicus GN=Rpl24 PE=2 SV=1
21.60	2	639.80	1277.59	-1.71	51	EFDELSPSAQR		E9PSX6	Sarcoplasmic/endoplasmic reticulum calcium ATPase 2 OS=Rattus norvegicus GN=Atp2a2 PE=3 SV=2
20.96	2	639.81	1277.60	-1.81	40	RMGESDDSILR		P05765	40S ribosomal protein S21 OS=Rattus norvegicus GN=Rps21 PE=1 SV=1
13.48	2	642.28	1282.55	-2.93	28	EGMSIVEAMER	[3] Oxidation (M)|[9] Oxidation (M)	P10111	Peptidyl-prolyl cis-trans isomerase A OS=Rattus norvegicus GN=Ppia PE=1 SV=2
12.00	2	642.36	1282.70	-2.91	44	TKTPGPGAQSALR		Q6PDV6	40S ribosomal protein S14 OS=Rattus norvegicus GN=Rps14 PE=2 SV=1
10.05	2	642.83	1283.65	-2.88	72	SLTATGGNHVTAR		D3ZHA0	Protein Flnc OS=Rattus norvegicus GN=Flnc PE=4 SV=1
6.90	2	644.78	1287.55	-3.41	35	TLEDQMNEHR	[6] Oxidation (M)	P02564	Myosin-7 OS=Rattus norvegicus GN=Myh7 PE=2 SV=2
6.90	2	644.78	1287.55	-3.41	35	TLEDQMNEHR	[6] Oxidation (M)	G3V8B0	Myosin-7 OS=Rattus norvegicus GN=Myh7 PE=4 SV=1
13.56	2	644.83	1287.65	-2.44	46	IEAQNRPFDAK		G3V6E1	Uncharacterized protein OS=Rattus norvegicus GN=Myh2 PE=4 SV=2
20.48	2	649.82	1297.62	-1.67	84	VNPDDVGGEALGR		P02091	Hemoglobin subunit beta-1 OS=Rattus norvegicus GN=Hbb PE=1 SV=3
30.36	2	649.85	1297.69	-1.05	50	KFLDGIYVSEK		Q6P9U5	Ribosomal protein L9 OS=Rattus norvegicus GN=Rpl9 PE=2 SV=1
17.47	3	433.58	1297.71	-3.06	28	HGHLGFLPHKR		D4A9G1	Protein Rpl3l OS=Rattus norvegicus GN=Rpl3l PE=3 SV=2
32.90	2	649.89	1297.76	-1.35	50	KLVILEGELER		Q5FVG5	Similar to tropomyosin 1. embryonic fibroblast-rat. isoform CRA_c OS=Rattus norvegicus GN=Tpm2 PE=2 SV=1
32.90	2	649.89	1297.76	-1.35	50	KLVILEGELER		P09495	Tropomyosin alpha-4 chain OS=Rattus norvegicus GN=Tpm4 PE=1 SV=3

26.95	2	650.34	1298.66	-1.39	43	AWGPGLETGQVGK		D3ZHA0	Protein Flnc OS=Rattus norvegicus GN=Flnc PE=4 SV=1
15.75	2	651.31	1300.61	-0.92	48	LMELHGEGGSSGK		P49242	40S ribosomal protein S3a OS=Rattus norvegicus GN=Rps3a PE=1 SV=2
27.04	2	651.82	1301.63	-0.90	42	GQSIDDMIPAQK		P00564	Creatine kinase M-type OS=Rattus norvegicus GN=Ckm PE=1 SV=2
43.48	2	651.86	1301.71	-1.81	52	SLDLDSIIAEVK		G3V908	Protein Kb15 OS=Rattus norvegicus GN=Kb15 PE=2 SV=2
19.52	2	652.36	1302.70	-2.78	57	VSGPGVEPHGVLR		D3ZHA0	Protein Flnc OS=Rattus norvegicus GN=Flnc PE=4 SV=1
35.92	2	652.37	1302.73	-1.04	30	ATGVFTTLQPLR		F1LST1	Fibronectin OS=Rattus norvegicus GN=Fn1 PE=4 SV=2
17.39	2	652.82	1303.63	-1.97	59	GVQVETISSGDGR		Q62658	Peptidyl-prolyl cis-trans isomerase FKBP1A OS=Rattus norvegicus GN=Fkbp1a PE=1 SV=3
22.74	2	652.84	1303.66	-2.24	41	DRDLEVDTTLK		P02454	Collagen alpha-1(I) chain OS=Rattus norvegicus GN=Col1a1 PE=1 SV=5
14.10	2	654.80	1307.59	-2.40	44	KNVEAMSGMEGR		F1LMC6	Troponin I. slow skeletal muscle (Fragment) OS=Rattus norvegicus GN=Tnni1 PE=4 SV=1
22.00	2	654.88	1307.74	-1.76	42	KTPAEILPSPQK		M0R9L0	Protein Naca OS=Rattus norvegicus GN=Naca PE=4 SV=1
30.95	2	655.36	1308.71	-1.97	35	EVPMVAVPPVGSK		F1M853	Protein Rrbp1 OS=Rattus norvegicus GN=Rrbp1 PE=4 SV=2
10.44	2	655.82	1309.62	-2.29	28	ALVADSHPESER		G3V6S0	Protein Sptbn1 OS=Rattus norvegicus GN=Sptbn1 PE=4 SV=2
11.92	2	656.86	1311.70	-2.77	32	ETSTPSPQKIPK		M0R9L0	Protein Naca OS=Rattus norvegicus GN=Naca PE=4 SV=1
43.08	2	656.88	1311.74	-1.94	37	IQAIELEDLLR		P47853	Biglycan OS=Rattus norvegicus GN=Bgn PE=2 SV=1
14.18	2	657.30	1312.59	-1.96	39	SYTVGVTMMHR	[8] Oxidation (M)|[9] Oxidation (M)	Q5RJR9	Serine (Or cysteine) proteinase inhibitor. clade H. member 1. isoform CRA_b OS=Rattus norvegicus GN=Serpinh1 PE=2 SV=1
9.81	2	657.79	1313.57	-2.09	30	NNASTSYDVTDK		D4A9G1	Protein Rpl3l OS=Rattus norvegicus GN=Rpl3l PE=3 SV=2
22.16	2	658.83	1315.64	-0.73	59	EQAEAEVASLNR		Q63610	Tropomyosin alpha-3 chain OS=Rattus norvegicus GN=Tpm3 PE=1 SV=2

15.35	2	658.86	1315.71	-2.23	42	VGSTKVPMTPGVK	[8] Oxidation (M)	E9PSX6	Sarcoplasmic/endoplasmic reticulum calcium ATPase 2 OS=Rattus norvegicus GN=Atp2a2 PE=3 SV=2
9.00	2	659.31	1316.60	-2.68	60	LMELHGEGGSSGK	[2] Oxidation (M)	P49242	40S ribosomal protein S3a OS=Rattus norvegicus GN=Rps3a PE=1 SV=2
17.62	2	659.82	1317.62	-1.62	54	GQSIDDMIPAQK	[7] Oxidation (M)	P00564	Creatine kinase M-type OS=Rattus norvegicus GN=Ckm PE=1 SV=2
9.67	2	660.32	1318.63	-2.14	77	GASNTLAESSASPK		M0R9L0	Protein Naca OS=Rattus norvegicus GN=Naca PE=4 SV=1
6.58	2	660.32	1318.63	-2.06	47	EGGKVTAETENGK		G3V885	Myosin-6 OS=Rattus norvegicus GN=Myh6 PE=4 SV=1
6.58	2	660.32	1318.63	-2.06	47	EGGKVTAETENGK		P02564	Myosin-7 OS=Rattus norvegicus GN=Myh7 PE=2 SV=2
6.58	2	660.32	1318.63	-2.06	47	EGGKVTAETENGK		G3V8B0	Myosin-7 OS=Rattus norvegicus GN=Myh7 PE=4 SV=1
22.41	2	660.85	1319.69	-1.48	31	KAGNFYVPAEPK		B0K031	60S ribosomal protein L7 OS=Rattus norvegicus GN=Rpl7 PE=2 SV=1
12.79	2	662.32	1322.63	-4.35	62	NMSVIAHVDHGK	[2] Oxidation (M)	P05197	Elongation factor 2 OS=Rattus norvegicus GN=Eef2 PE=1 SV=4
17.24	2	662.36	1322.70	-2.21	40	DRLLPPTQNNR		D3ZUL3	Protein Col6a1 OS=Rattus norvegicus GN=Col6a1 PE=4 SV=1
11.03	2	662.36	1322.70	-1.93	48	LGVRPSQGGEAPR		F1LST1	Fibronectin OS=Rattus norvegicus GN=Fn1 PE=4 SV=2
27.04	2	663.36	1324.70	-1.04	26	EVPMVAVPPVGSK	[4] Oxidation (M)	F1M853	Protein Rrbp1 OS=Rattus norvegicus GN=Rrbp1 PE=4 SV=2
22.33	3	442.59	1324.74	-2.29	47	DNIQGITKPAIR		P62804	Histone H4 OS=Rattus norvegicus GN=Hist1h4b PE=1 SV=2
22.33	2	663.38	1324.74	-1.36	38	DNIQGITKPAIR		P62804	Histone H4 OS=Rattus norvegicus GN=Hist1h4b PE=1 SV=2
25.84	2	663.88	1325.75	-1.55	74	KASGPPVSELITK		P15865	Histone H1.4 OS=Rattus norvegicus GN=Hist1h1e PE=1 SV=3
25.84	2	663.88	1325.75	-1.55	74	KASGPPVSELITK		M0R7B4	Protein LOC684828 OS=Rattus norvegicus GN=LOC684828 PE=3 SV=1
20.88	2	664.83	1327.65	-1.10	66	DIANTPHELYR		F1LNH3	Procollagen. type VI. alpha 2. isoform CRA_a OS=Rattus norvegicus GN=Col6a2 PE=4 SV=2
21.92	2	664.86	1327.71	-1.72	34	SGDDVRGPSVVLK		D4A111	Protein Col6a3 OS=Rattus norvegicus GN=Col6a3 PE=4 SV=2

30.45	3	444.59	1330.76	-2.70	25	VLTPDLYNKLR		P00564	Creatine kinase M-type OS=Rattus norvegicus GN=Ckm PE=1 SV=2
30.45	2	666.39	1330.76	-1.80	53	VLTPDLYNKLR		P00564	Creatine kinase M-type OS=Rattus norvegicus GN=Ckm PE=1 SV=2
19.03	2	666.81	1331.60	-2.27	59	HQEGEIFDTEK		F1LQS3	60S ribosomal protein L6 OS=Rattus norvegicus GN=Rpl6 PE=3 SV=1
20.31	2	666.82	1331.63	-1.57	65	ATDAEADVASLNR		Q5FVG5	Similar to tropomyosin 1. embryonic fibroblast-rat. isoform CRA_c OS=Rattus norvegicus GN=Tpm2 PE=2 SV=1
20.31	2	666.82	1331.63	-1.57	65	ATDAEADVASLNR		P04692	Tropomyosin alpha-1 chain OS=Rattus norvegicus GN=Tpm1 PE=1 SV=3
24.94	2	666.85	1331.69	-1.45	103	GILAADESTGSIAK		P05065	Fructose-bisphosphate aldolase A OS=Rattus norvegicus GN=Aldoa PE=1 SV=2
24.94	2	666.85	1331.69	-1.45	103	GILAADESTGSIAK		Q6AY07	Fructose-bisphosphate aldolase OS=Rattus norvegicus GN=Aldoart2 PE=2 SV=1
29.30	2	666.87	1331.73	-3.94	34	ALRYPMAVGLNK		D3ZZ95	60S ribosomal protein L36 OS=Rattus norvegicus GN=LOC100361060 PE=3 SV=1
25.92	2	668.35	1334.68	-1.39	49	EIAQDFKTDLR		B0BMY8	Histone H3 OS=Rattus norvegicus GN=H3f3b PE=2 SV=1
11.03	2	669.37	1336.73	-2.70	30	TKAEPAAPQTPVK		D3ZHA7	Protein Myl6b OS=Rattus norvegicus GN=Myl6b PE=4 SV=1
18.02	3	446.89	1337.65	-2.12	28	GDNQSPIELHTK		P14141	Carbonic anhydrase 3 OS=Rattus norvegicus GN=Ca3 PE=1 SV=3
18.02	2	669.83	1337.66	-1.66	49	GDNQSPIELHTK		P14141	Carbonic anhydrase 3 OS=Rattus norvegicus GN=Ca3 PE=1 SV=3
28.16	2	670.34	1338.66	-1.39	66	NMLFSGTNIAAGK	[2] Oxidation (M)	E9PSX6	Sarcoplasmic/endoplasmic reticulum calcium ATPase 2 OS=Rattus norvegicus GN=Atp2a2 PE=3 SV=2
28.16	2	670.34	1338.66	-1.39	66	NMLFSGTNIAAGK	[2] Oxidation (M)	B4F7E5	ATPase. Ca++ transporting. cardiac muscle. fast twitch 1 OS=Rattus norvegicus GN=Atp2a1 PE=2 SV=1
28.16	2	670.34	1338.66	-1.39	66	NMLFSGTNIAAGK	[2] Oxidation (M)	M0RCD2	Sarcoplasmic/endoplasmic reticulum calcium ATPase 1 OS=Rattus norvegicus GN=Atp2a1 PE=3 SV=1
26.45	2	670.89	1339.77	-1.48	60	KATGPPVSELITK		D3ZBN0	Histone H1.5 OS=Rattus norvegicus GN=Hist1h1b PE=3 SV=1

31.29	2	671.33	1340.65	-1.95	41	MKFNPFVTSDR		G3V6I9	60S ribosomal protein L26 OS=Rattus norvegicus GN=Rpl26 PE=3 SV=1
14.26	2	671.34	1340.66	-1.98	52	FGQGGAGPVGGQGPR		F1LQW3	Protein Sfpq (Fragment) OS=Rattus norvegicus GN=Sfpq PE=4 SV=1
24.43	2	671.37	1340.73	-1.83	28	VLDGIPPPYDKK		Q5RK10	60S ribosomal protein L13a OS=Rattus norvegicus GN=Rpl13a PE=2 SV=1
11.07	2	671.87	1341.72	-2.64	57	ALGTNPTNAEVKK		P02600	Myosin light chain 1/3. skeletal muscle isoform OS=Rattus norvegicus GN=Myl1 PE=1 SV=2
15.58	2	672.34	1342.67	-2.01	60	QLEEEQQALQK		Q5FVG5	Similar to tropomyosin 1. embryonic fibroblast-rat. isoform CRA_c OS=Rattus norvegicus GN=Tpm2 PE=2 SV=1
19.36	2	672.36	1342.71	-4.50	48	ADIAESQVNKLR		P02564	Myosin-7 OS=Rattus norvegicus GN=Myh7 PE=2 SV=2
19.36	2	672.36	1342.71	-4.50	48	ADIAESQVNKLR		G3V885	Myosin-6 OS=Rattus norvegicus GN=Myh6 PE=4 SV=1
19.36	2	672.36	1342.71	-4.50	48	ADIAESQVNKLR		G3V6D8	Myosin-3 OS=Rattus norvegicus GN=Myh3 PE=4 SV=1
19.36	2	672.36	1342.71	-4.50	48	ADIAESQVNKLR		F1LMU0	Myosin-4 OS=Rattus norvegicus GN=Myh4 PE=2 SV=1
19.36	2	672.36	1342.71	-4.50	48	ADIAESQVNKLR		G3V8B0	Myosin-7 OS=Rattus norvegicus GN=Myh7 PE=4 SV=1
19.36	2	672.36	1342.71	-4.50	48	ADIAESQVNKLR		F1M8F6	Myosin-8 (Fragment) OS=Rattus norvegicus GN=Myh8 PE=4 SV=2
19.36	2	672.36	1342.71	-4.50	48	ADIAESQVNKLR		F1LRV9	Protein Myh1 OS=Rattus norvegicus GN=Myh1 PE=2 SV=2
19.36	2	672.36	1342.71	-4.50	48	ADIAESQVNKLR		F1M789	Protein Myh13 OS=Rattus norvegicus GN=Myh13 PE=4 SV=2
19.36	2	672.36	1342.71	-4.50	48	ADIAESQVNKLR		G3V6E1	Uncharacterized protein OS=Rattus norvegicus GN=Myh2 PE=4 SV=2
12.08	2	673.36	1344.70	-2.10	48	AKLQTENGELSR		P02564	Myosin-7 OS=Rattus norvegicus GN=Myh7 PE=2 SV=2
12.08	2	673.36	1344.70	-2.10	48	AKLQTENGELSR		G3V8B0	Myosin-7 OS=Rattus norvegicus GN=Myh7 PE=4 SV=1
30.11	2	673.37	1344.72	-2.38	69	TAVVVGTITDDVR		Q0QEW8	60S ribosomal protein L18 (Fragment) OS=Rattus norvegicus GN=Rpl18 PE=2 SV=1
12.94	2	674.37	1346.73	-3.39	57	KYELGRPAANTK		B2RYR8	40S ribosomal protein S8 OS=Rattus norvegicus GN=Rps8 PE=2 SV=1
31.65	2	675.34	1348.66	-0.70	82	TIEGIVMAADSAR	[7] Oxidation (M)	F1LWG8	5-hydroxytryptamine receptor 2B OS=Rattus norvegicus GN=Srl PE=2 SV=2

34.33	2	675.84	1349.67	-1.38	40	ELAEAEDVSIFK		P29419	ATP synthase subunit e. mitochondrial OS=Rattus norvegicus GN=Atp5i PE=1 SV=3
19.11	2	675.87	1349.73	-1.93	33	VHVQPAVDTSGIK		D3ZHA0	Protein Flnc OS=Rattus norvegicus GN=Flnc PE=4 SV=1
18.95	2	676.33	1350.64	-1.88	50	LTGAIMHYGNMK	[11] Oxidation (M)	G3V885	Myosin-6 OS=Rattus norvegicus GN=Myh6 PE=4 SV=1
18.95	2	676.33	1350.64	-1.88	51	LTGAIMHFGNMK	[6] Oxidation (M)|[11] Oxidation (M)	P02564	Myosin-7 OS=Rattus norvegicus GN=Myh7 PE=2 SV=2
18.95	2	676.33	1350.64	-1.88	51	LTGAIMHFGNMK	[6] Oxidation (M)|[11] Oxidation (M)	G3V8B0	Myosin-7 OS=Rattus norvegicus GN=Myh7 PE=4 SV=1
13.48	2	676.78	1351.55	-2.97	51	SSEEEEQVMER		D3ZWJ2	Nestin OS=Rattus norvegicus GN=Nes PE=3 SV=2
7.57	2	677.81	1353.61	-2.03	71	DSYVGDEAQSKR		P68035	Actin. alpha cardiac muscle 1 OS=Rattus norvegicus GN=Actc1 PE=2 SV=1
7.57	2	677.81	1353.61	-2.03	71	DSYVGDEAQSKR		P68136	Actin. alpha skeletal muscle OS=Rattus norvegicus GN=Acta1 PE=1 SV=1
7.57	2	677.81	1353.61	-2.03	71	DSYVGDEAQSKR		P60711	Actin. cytoplasmic 1 OS=Rattus norvegicus GN=Actb PE=1 SV=1
22.41	2	677.87	1353.72	-1.56	49	ALGQNPTNAEVLK		D3ZHA7	Protein Myl6b OS=Rattus norvegicus GN=Myl6b PE=4 SV=1
22.41	2	677.87	1353.72	-1.56	49	ALGQNPTNAEVLK		B2GV99	Myl6 protein OS=Rattus norvegicus GN=Myl6 PE=2 SV=1
26.95	2	679.33	1356.65	-1.36	45	MKFNPFVTSDR	[1] Oxidation (M)	G3V6I9	60S ribosomal protein L26 OS=Rattus norvegicus GN=Rpl26 PE=3 SV=1
18.79	2	679.86	1357.71	-1.94	50	LLATEQEDAAVAK		F1M853	Protein Rrbp1 OS=Rattus norvegicus GN=Rrbp1 PE=4 SV=2
13.34	2	680.35	1358.68	-2.41	47	SGAQASSTPLSPTR		G3V8L3	Lamin A. isoform CRA_b OS=Rattus norvegicus GN=Lmna PE=3 SV=1
39.53	2	680.85	1359.69	-1.17	58	EGDVLTLLESER		P62859	40S ribosomal protein S28 OS=Rattus norvegicus GN=Rps28 PE=1 SV=1
32.90	2	680.88	1359.75	-1.36	43	ALELFRNDIAAK		Q9QZ76	Myoglobin OS=Rattus norvegicus GN=Mb PE=1 SV=3

25.35	2	681.35	1360.69	-1.78	31	VDTWFNQPARK		P41123	60S ribosomal protein L13 OS=Rattus norvegicus GN=Rpl13 PE=1 SV=2
21.53	2	681.86	1361.70	-0.87	29	GASIVPTETSVSSK		M0R9L0	Protein Naca OS=Rattus norvegicus GN=Naca PE=4 SV=1
29.86	2	684.38	1366.75	-1.77	84	VISLSGEHSIIGR		Q6PEC5	Superoxide dismutase [Cu-Zn] (Fragment) OS=Rattus norvegicus PE=2 SV=1
24.35	2	684.86	1367.71	-2.06	33	TPVATVPAMPQEK		F1M853	Protein Rrbp1 OS=Rattus norvegicus GN=Rrbp1 PE=4 SV=2
21.53	2	685.37	1368.73	-1.13	75	GAAQNIIPASTGAAK		M0R590	Protein LOC685186 OS=Rattus norvegicus GN=LOC685186 PE=3 SV=1
21.53	2	685.37	1368.73	-1.13	75	GAAQNIIPASTGAAK		D3ZME3	Glyceraldehyde-3-phosphate dehydrogenase OS=Rattus norvegicus PE=3 SV=1
25.84	2	686.35	1370.69	-1.35	31	STLPVDEGSPLEK		Q5M7W5	Microtubule-associated protein 4 OS=Rattus norvegicus GN=Map4 PE=1 SV=1
33.64	2	686.87	1371.73	-1.16	47	ELGTIQQVISER		D4A111	Protein Col6a3 OS=Rattus norvegicus GN=Col6a3 PE=4 SV=2
37.55	2	687.36	1372.70	-1.06	30	MSPVPDLVPGSFK		A1L114	Fga protein OS=Rattus norvegicus GN=Fga PE=2 SV=1
29.70	2	687.86	1373.70	-1.79	38	RPFFPFHSPSR		P23928	Alpha-crystallin B chain OS=Rattus norvegicus GN=Cryab PE=1 SV=1
17.47	2	687.87	1373.73	-2.40	40	ISVREPMQTGIK	[7] Oxidation (M)	M0R4D7	Uncharacterized protein OS=Rattus norvegicus PE=3 SV=1
6.23	2	689.29	1376.56	-0.91	56	SSSSSSSSSSSSASR		B5DFG4	Heat shock 27kD protein family. member 7 (Cardiovascular) OS=Rattus norvegicus GN=Hspb7 PE=2 SV=1
21.04	2	689.32	1376.62	-1.46	52	GGGGNFGPGPGSNFR		M0R6J9	Heterogeneous nuclear ribonucleoproteins A2/B1 OS=Rattus norvegicus GN=Hnrnpa2b1 PE=4 SV=1
23.28	2	689.82	1377.62	-1.34	29	DGVLDSVTDQDSK		B2RZB2	Uncharacterized protein OS=Rattus norvegicus PE=2 SV=1
18.57	2	690.34	1378.66	-2.16	38	GLTSSEPVSVMEK	[11] Oxidation (M)	D3Z9F8	Collagen alpha-1(XII) chain OS=Rattus norvegicus GN=Col12a1 PE=4 SV=2
21.68	2	690.86	1379.70	-1.56	64	GNPTVEVDLHTAK		P15429	Beta-enolase OS=Rattus norvegicus GN=Eno3 PE=1 SV=3

21.68	2	690.86	1379.70	-1.56	64	GNPTVEVDLHTAK		P07323	Gamma-enolase OS=Rattus norvegicus GN=Eno2 PE=1 SV=2
34.76	2	691.43	1380.84	-1.65	53	KGLTPSQIGVILR		M0RBC7	Protein Bco2 (Fragment) OS=Rattus norvegicus GN=Bco2 PE=3 SV=1
34.76	2	691.43	1380.84	-1.65	53	KGLTPSQIGVILR		M0RCY2	Protein LOC683961 OS=Rattus norvegicus GN=LOC683961 PE=3 SV=1
18.41	2	692.39	1382.76	-1.90	41	QLQLAQEAAQKR		P30427	Plectin OS=Rattus norvegicus GN=Plec PE=1 SV=2
22.09	2	692.84	1383.67	-6.08	32	YLAEFATGNDRK		P62260	14-3-3 protein epsilon OS=Rattus norvegicus GN=Ywhae PE=1 SV=1
15.42	2	692.86	1383.71	-0.73	41	TPVATVPAMPQEK	[9] Oxidation (M)	F1M853	Protein Rrbp1 OS=Rattus norvegicus GN=Rrbp1 PE=4 SV=2
45.19	2	692.90	1383.79	-1.83	60	IGDLQSEIVGLLK		D4A111	Protein Col6a3 OS=Rattus norvegicus GN=Col6a3 PE=4 SV=2
8.19	2	693.35	1384.69	-4.67	40	GHTPTHPGALNQR		F1LTJ5	Protein Hspg2 OS=Rattus norvegicus GN=Hspg2 PE=4 SV=2
23.68	2	694.82	1387.64	-0.64	83	VVDLMAYMASKE	[5] Oxidation (M)|[8] Oxidation (M)	M0R590	Protein LOC685186 OS=Rattus norvegicus GN=LOC685186 PE=3 SV=1
23.68	2	694.82	1387.64	-0.64	83	VVDLMAYMASKE	[5] Oxidation (M)|[8] Oxidation (M)	M0R451	Uncharacterized protein OS=Rattus norvegicus PE=3 SV=1
23.68	2	694.82	1387.64	-0.64	83	VVDLMAYMASKE	[5] Oxidation (M)|[8] Oxidation (M)	D3ZIY0	Uncharacterized protein OS=Rattus norvegicus PE=3 SV=1
26.87	2	695.87	1389.72	-2.29	40	SMLRGGPLSGPYR		P14141	Carbonic anhydrase 3 OS=Rattus norvegicus GN=Ca3 PE=1 SV=3
27.69	2	696.36	1390.71	-1.46	37	SEIGIAMGSGTAVAK		E9PSX6	Sarcoplasmic/endoplasmic reticulum calcium ATPase 2 OS=Rattus norvegicus GN=Atp2a2 PE=3 SV=2
36.58	2	697.36	1392.70	-1.38	54	FVEGLPINDFSR		O88989	Malate dehydrogenase. cytoplasmic OS=Rattus norvegicus GN=Mdh1 PE=1 SV=3
16.30	2	697.87	1393.72	-3.12	59	VVANSKESYELR		Q6PDV8	RCG31311 OS=Rattus norvegicus GN=LOC100363800 PE=2 SV=1

31.80	2	697.91	1393.80	-1.77	26	NKNGVAPIIDVVR		D3ZF07	Protein RGD1562402 OS=Rattus norvegicus GN=RGD1562402 PE=3 SV=1
23.68	2	698.88	1395.75	-0.59	73	ALGQNPTQAEVLR		P16409	Myosin light chain 3 OS=Rattus norvegicus GN=Myl3 PE=2 SV=2
39.71	2	699.36	1396.70	-1.68	46	ELNDFISYLQR		P11598	Protein disulfide-isomerase A3 OS=Rattus norvegicus GN=Pdia3 PE=1 SV=2
29.62	2	700.38	1398.74	-1.59	37	RIQLVEEELDR		P04692	Tropomyosin alpha-1 chain OS=Rattus norvegicus GN=Tpm1 PE=1 SV=3
29.62	2	700.38	1398.74	-1.59	37	RIQLVEEELDR		Q5FVG5	Similar to tropomyosin 1. embryonic fibroblast-rat. isoform CRA_c OS=Rattus norvegicus GN=Tpm2 PE=2 SV=1
29.62	2	700.38	1398.74	-1.59	37	RIQLVEEELDR		Q6AZ25	Tropomyosin 1. alpha OS=Rattus norvegicus GN=Tpm1 PE=2 SV=1
29.62	2	700.38	1398.74	-1.59	37	RIQLVEEELDR		Q63610	Tropomyosin alpha-3 chain OS=Rattus norvegicus GN=Tpm3 PE=1 SV=2
29.62	2	700.38	1398.74	-1.59	37	RIQLVEEELDR		P09495	Tropomyosin alpha-4 chain OS=Rattus norvegicus GN=Tpm4 PE=1 SV=3
15.42	2	701.83	1401.65	-1.85	46	VLGNPSNEEMNAK		P02600	Myosin light chain 1/3. skeletal muscle isoform OS=Rattus norvegicus GN=Myl1 PE=1 SV=2
23.52	2	701.87	1401.73	-1.38	30	EASVLSPVPTSSTK		M0R9L0	Protein Naca OS=Rattus norvegicus GN=Naca PE=4 SV=1
29.78	2	701.90	1401.79	-2.30	43	EITALAPSTMKIK		P60711	Actin. cytoplasmic 1 OS=Rattus norvegicus GN=Actb PE=1 SV=1
29.78	2	701.90	1401.79	-2.30	43	EITALAPSTMKIK		P68035	Actin. alpha cardiac muscle 1 OS=Rattus norvegicus GN=Actc1 PE=2 SV=1
29.78	2	701.90	1401.79	-2.30	43	EITALAPSTMKIK		P68136	Actin. alpha skeletal muscle OS=Rattus norvegicus GN=Acta1 PE=1 SV=1
41.25	2	702.85	1403.69	-0.89	70	GADPEETILNAFK		P08733	Myosin regulatory light chain 2. ventricular/cardiac muscle isoform OS=Rattus norvegicus GN=Myl2 PE=1 SV=2
21.12	2	703.86	1405.71	-1.48	48	SMLRGGPLSGPYR	[2] Oxidation (M)	P14141	Carbonic anhydrase 3 OS=Rattus norvegicus GN=Ca3 PE=1 SV=3

19.91	2	704.36	1406.70	-2.27	106	SEIGIAMGSGTAVA K	[7] Oxidation (M)	E9PSX6	Sarcoplasmic/endoplasmic reticulum calcium ATPase 2 OS=Rattus norvegicus GN=Atp2a2 PE=3 SV=2
31.46	2	704.85	1407.69	-1.46	88	IGIFGQDEDVTSK		E9PSX6	Sarcoplasmic/endoplasmic reticulum calcium ATPase 2 OS=Rattus norvegicus GN=Atp2a2 PE=3 SV=2
10.17	2	705.85	1409.68	-2.14	46	YHTINGHNAEVR		M0R6J9	Heterogeneous nuclear ribonucleoproteins A2/B1 OS=Rattus norvegicus GN=Hnrnpa2b1 PE=4 SV=1
18.10	2	705.86	1409.70	-1.93	86	DAPTTLAESPPSPK		M0R9L0	Protein Naca OS=Rattus norvegicus GN=Naca PE=4 SV=1
33.15	2	705.89	1409.76	-1.91	30	QLFHPEQLITGK		Q6P9V9	Tubulin alpha-1B chain OS=Rattus norvegicus GN=Tuba1b PE=1 SV=1
33.15	2	705.89	1409.76	-1.91	30	QLFHPEQLITGK		Q5XIF6	Tubulin alpha-4A chain OS=Rattus norvegicus GN=Tuba4a PE=2 SV=1
33.15	2	705.89	1409.76	-1.91	30	QLFHPEQLITGK		M0R5B4	Uncharacterized protein OS=Rattus norvegicus PE=4 SV=1
26.62	2	707.38	1412.75	-1.21	63	RASAPLPGFSTPGR		P97541	Heat shock protein beta-6 OS=Rattus norvegicus GN=Hspb6 PE=1 SV=1
40.65	2	708.89	1415.76	-0.97	91	AAAITSDLLESLGR		D3ZAF5	Periostin. osteoblast specific factor (Predicted). isoform CRA_a OS=Rattus norvegicus GN=Postn PE=4 SV=1
34.25	2	709.39	1416.77	-1.67	49	AITGASLADIMAKR		P83732	60S ribosomal protein L24 OS=Rattus norvegicus GN=Rpl24 PE=2 SV=1
9.47	2	709.83	1417.65	-1.87	54	VLGNPSNEEMNAK	[10] Oxidation (M)	P02600	Myosin light chain 1/3. skeletal muscle isoform OS=Rattus norvegicus GN=Myl1 PE=1 SV=2
21.60	2	709.87	1417.72	-1.86	28	VRYSLDPENPTK		P24049	60S ribosomal protein L17 OS=Rattus norvegicus GN=Rpl17 PE=2 SV=3
23.28	2	709.90	1417.78	-1.25	49	EITALAPSTMKIK	[10] Oxidation (M)	P60711	Actin. cytoplasmic 1 OS=Rattus norvegicus GN=Actb PE=1 SV=1
23.28	2	709.90	1417.78	-1.25	49	EITALAPSTMKIK	[10] Oxidation (M)	P68035	Actin. alpha cardiac muscle 1 OS=Rattus norvegicus GN=Actc1 PE=2 SV=1
23.28	2	709.90	1417.78	-1.25	49	EITALAPSTMKIK	[10] Oxidation (M)	P68136	Actin. alpha skeletal muscle OS=Rattus norvegicus GN=Acta1 PE=1 SV=1
33.56	2	711.85	1421.69	-1.03	55	GVFPENFTERVQ		F1LMX1	Myc box-dependent-interacting protein 1 OS=Rattus norvegicus GN=Bin1 PE=4 SV=2

23.20	2	712.34	1422.66	-0.89	67	ELAEDGYSGVEVR		P62909	40S ribosomal protein S3 OS=Rattus norvegicus GN=Rps3 PE=1 SV=1
27.76	2	712.90	1423.78	-1.05	32	TPGNRIVYLYTK		F1LQ14	60S ribosomal protein L34 OS=Rattus norvegicus GN=Rpl34 PE=2 SV=1
10.63	2	714.86	1427.70	-2.07	45	AGFGTKGSSSSVTSR		Q6P725	Desmin OS=Rattus norvegicus GN=Des PE=2 SV=1
18.25	2	715.31	1428.61	-1.40	52	MGLAMGGAGGAS FDR	[1] Oxidation (M)|[5] Oxidation (M)	F1LV13	Heterogeneous nuclear ribonucleoprotein M OS=Rattus norvegicus GN=Hnrnpm PE=2 SV=1
22.09	2	715.36	1428.71	-0.96	52	IEDVTPIPSDSTR		Q6PDV6	40S ribosomal protein S14 OS=Rattus norvegicus GN=Rps14 PE=2 SV=1
21.45	2	716.36	1430.70	-1.50	90	EATEAATVAAAAEAR		Q3MIE4	Synaptic vesicle membrane protein VAT-1 homolog OS=Rattus norvegicus GN=Vat1 PE=1 SV=1
23.12	2	716.38	1430.74	-1.70	36	WSRPQAPITGYR		F1LST1	Fibronectin OS=Rattus norvegicus GN=Fn1 PE=4 SV=2
22.82	2	718.86	1435.71	-1.44	45	YADQEVPRSPFK		D3ZHA0	Protein Flnc OS=Rattus norvegicus GN=Flnc PE=4 SV=1
35.68	2	718.88	1435.75	-1.04	44	TNGKEPELLEPIP		D3ZRA9	Uncharacterized protein OS=Rattus norvegicus GN=RGD1560414 PE=4 SV=1
17.47	2	719.36	1436.71	-1.95	86	ATSPSTLVSTGSSSR		Q5M7W5	Microtubule-associated protein 4 OS=Rattus norvegicus GN=Map4 PE=1 SV=1
20.07	2	719.87	1437.73	-0.96	38	LTSDDVKEQIYK		M0RCY2	Protein LOC683961 OS=Rattus norvegicus GN=LOC683961 PE=3 SV=1
27.29	2	720.40	1438.78	-0.74	52	APQVSTPTLVEAAR		P02770	Serum albumin OS=Rattus norvegicus GN=Alb PE=1 SV=2
41.25	2	721.38	1440.76	-1.25	44	NNLLQAELEELR		P02564	Myosin-7 OS=Rattus norvegicus GN=Myh7 PE=2 SV=2
41.25	2	721.38	1440.76	-1.25	44	NNLLQAELEELR		G3V8B0	Myosin-7 OS=Rattus norvegicus GN=Myh7 PE=4 SV=1
27.76	2	721.88	1441.74	-0.96	51	EAGAGGLSIAVEGPSK		D3ZHA0	Protein Flnc OS=Rattus norvegicus GN=Flnc PE=4 SV=1
27.76	2	721.88	1441.74	-0.96	51	EAGAGGLSIAVEGPSK		D3ZD13	Uncharacterized protein (Fragment) OS=Rattus norvegicus PE=4 SV=2
22.24	2	722.86	1443.70	-0.83	47	SLYSSSPGGAYVTR		P31000	Vimentin OS=Rattus norvegicus GN=Vim PE=1 SV=2
9.81	2	722.86	1443.70	-2.48	44	ARNDESQLSPATR		P30427	Plectin OS=Rattus norvegicus GN=Plec PE=1 SV=2
49.34	2	722.89	1443.76	-1.31	64	EGIPALDNFLDKL		P05197	Elongation factor 2 OS=Rattus norvegicus GN=Eef2 PE=1 SV=4

14.18	2	725.36	1448.70	-0.30	95	ISTGGGETEETLQK		D3ZAF5	Periostin. osteoblast specific factor (Predicted). isoform CRA_a OS=Rattus norvegicus GN=Postn PE=4 SV=1
35.75	2	726.38	1450.75	-1.64	45	AVDIPHMDIEALK		Q4KM60	Ribosomal protein (Fragment) OS=Rattus norvegicus GN=Rpl10a PE=2 SV=1
31.65	2	727.92	1453.82	-1.61	44	LKVLDGIPPPYDK		Q5RK10	60S ribosomal protein L13a OS=Rattus norvegicus GN=Rpl13a PE=2 SV=1
31.65	2	727.92	1453.82	8.91	34	VEGEIKVPEVDIK		Q38PG0	AHNAK 1 (Fragment) OS=Rattus norvegicus PE=2 SV=1
22.74	2	730.35	1458.69	-1.36	81	MGPAMGPALGAGIER	[1] Oxidation (M)|[5] Oxidation (M)	F1LV13	Heterogeneous nuclear ribonucleoprotein M OS=Rattus norvegicus GN=Hnrnpm PE=2 SV=1
18.71	2	730.87	1459.72	-1.56	102	KATDAEADVASLNR		Q5FVG5	Similar to tropomyosin 1. embryonic fibroblast-rat. isoform CRA_c OS=Rattus norvegicus GN=Tpm2 PE=2 SV=1
18.71	2	730.87	1459.72	-1.56	102	KATDAEADVASLNR		P04692	Tropomyosin alpha-1 chain OS=Rattus norvegicus GN=Tpm1 PE=1 SV=3
28.41	2	732.38	1462.74	-3.79	52	TMLESAGGLIQTAR	[2] Oxidation (M)	G3V852	Protein Tln1 OS=Rattus norvegicus GN=Tln1 PE=4 SV=1
7.57	2	732.90	1463.79	-2.69	54	VTTAPPEKPATPQK		M0R9L0	Protein Naca OS=Rattus norvegicus GN=Naca PE=4 SV=1
13.62	2	733.83	1465.64	-1.52	48	HIMGQNVADYMR	[3] Oxidation (M)|[11] Oxidation (M)	P09895	60S ribosomal protein L5 OS=Rattus norvegicus GN=Rpl5 PE=1 SV=3
13.62	2	733.83	1465.64	-1.52	48	HIMGQNVADYMR	[3] Oxidation (M)|[11] Oxidation (M)	D3ZHP8	Protein Rpl5l1 OS=Rattus norvegicus GN=Rpl5l1 PE=3 SV=1
34.33	2	734.37	1466.73	-1.04	55	DEEVHTGLGELLR		Q5RJR9	Serine (Or cysteine) proteinase inhibitor. clade H. member 1. isoform CRA_b OS=Rattus norvegicus GN=Serpinh1 PE=2 SV=1
29.62	2	735.89	1469.76	-1.44	41	DEILPTTPISEQK		P62909	40S ribosomal protein S3 OS=Rattus norvegicus GN=Rps3 PE=1 SV=1
29.62	2	735.89	1469.76	-1.44	41	DEILPTTPISEQK		D3ZVH2	Protein RGD1560831 OS=Rattus norvegicus GN=RGD1560831 PE=3 SV=1

14.34	2	736.39	1470.77	-1.45	61	QLEEEQQALQKK		Q5FVG5	Similar to tropomyosin 1. embryonic fibroblast-rat. isoform CRA_c OS=Rattus norvegicus GN=Tpm2 PE=2 SV=1
27.38	2	736.89	1471.77	-1.09	30	SQHPYVLTADTLK		D4A111	Protein Col6a3 OS=Rattus norvegicus GN=Col6a3 PE=4 SV=2
12.16	2	737.88	1473.75	-2.22	56	NKSTESLQANVQR		P41123	60S ribosomal protein L13 OS=Rattus norvegicus GN=Rpl13 PE=1 SV=2
12.16	2	737.88	1473.75	-2.22	56	NKSTESLQANVQR		D3ZD02	60S ribosomal protein L13 OS=Rattus norvegicus GN=RGD1563145 PE=3 SV=1
26.79	2	738.40	1474.78	-0.01	62	IEEALGDKAVFAGR		P15429	Beta-enolase OS=Rattus norvegicus GN=Eno3 PE=1 SV=3
7.41	2	738.84	1475.67	-2.62	56	LEEAEKAADESER		P04692	Tropomyosin alpha-1 chain OS=Rattus norvegicus GN=Tpm1 PE=1 SV=3
7.41	2	738.84	1475.67	-2.62	56	LEEAEKAADESER		Q5FVG5	Similar to tropomyosin 1. embryonic fibroblast-rat. isoform CRA_c OS=Rattus norvegicus GN=Tpm2 PE=2 SV=1
7.41	2	738.84	1475.67	-2.62	56	LEEAEKAADESER		Q6AZ25	Tropomyosin 1. alpha OS=Rattus norvegicus GN=Tpm1 PE=2 SV=1
7.41	2	738.84	1475.67	-2.62	56	LEEAEKAADESER		Q63610	Tropomyosin alpha-3 chain OS=Rattus norvegicus GN=Tpm3 PE=1 SV=2
7.41	2	738.84	1475.67	-2.62	56	LEEAEKAADESER		P09495	Tropomyosin alpha-4 chain OS=Rattus norvegicus GN=Tpm4 PE=1 SV=3
10.25	2	738.90	1475.78	-1.84	70	VTAETENGKTVTVK		G3V885	Myosin-6 OS=Rattus norvegicus GN=Myh6 PE=4 SV=1
10.25	2	738.90	1475.78	-1.84	70	VTAETENGKTVTVK		P02564	Myosin-7 OS=Rattus norvegicus GN=Myh7 PE=2 SV=2
10.25	2	738.90	1475.78	-1.84	70	VTAETENGKTVTVK		G3V8B0	Myosin-7 OS=Rattus norvegicus GN=Myh7 PE=4 SV=1
29.05	2	738.91	1475.81	-1.14	61	YLSERIPTLQTR		F1LMC6	Troponin I. slow skeletal muscle (Fragment) OS=Rattus norvegicus GN=Tnni1 PE=4 SV=1
25.19	2	739.37	1476.72	-0.80	65	DIELVMSQANVSR	[6] Oxidation (M)	M0R9L0	Protein Naca OS=Rattus norvegicus GN=Naca PE=4 SV=1
32.31	2	739.85	1477.69	-0.45	32	APSWIDTGLSEMR	[12] Oxidation (M)	P23928	Alpha-crystallin B chain OS=Rattus norvegicus GN=Cryab PE=1 SV=1

31.46	2	739.86	1477.70	-0.82	74	IGIFSENEEVADR		B4F7E5	ATPase. Ca++ transporting. cardiac muscle. fast twitch 1 OS=Rattus norvegicus GN=Atp2a1 PE=2 SV=1
31.46	2	739.86	1477.70	-0.82	74	IGIFSENEEVADR		M0RCD2	Sarcoplasmic/endoplasmic reticulum calcium ATPase 1 OS=Rattus norvegicus GN=Atp2a1 PE=3 SV=1
35.27	2	742.90	1483.79	-1.45	43	KYDAFLASESLIK		Q4KM60	Ribosomal protein (Fragment) OS=Rattus norvegicus GN=Rpl10a PE=2 SV=1
27.84	2	744.35	1486.69	-0.62	63	TTPSYVAFTDTER		P63018	Heat shock cognate 71 kDa protein OS=Rattus norvegicus GN=Hspa8 PE=1 SV=1
27.84	2	744.35	1486.69	-0.62	63	TTPSYVAFTDTER		Q07439	Heat shock 70 kDa protein 1A/1B OS=Rattus norvegicus GN=Hspa1a PE=2 SV=2
19.11	2	744.87	1487.73	-1.79	38	ATDAEADVASLNRR		Q5FVG5	Similar to tropomyosin 1. embryonic fibroblast-rat. isoform CRA_c OS=Rattus norvegicus GN=Tpm2 PE=2 SV=1
19.11	2	744.87	1487.73	-1.79	38	ATDAEADVASLNRR		P04692	Tropomyosin alpha-1 chain OS=Rattus norvegicus GN=Tpm1 PE=1 SV=3
30.45	2	744.88	1487.75	-0.94	46	IFVGGLSPDTPEEK		G3V6A4	Heterogeneous nuclear ribonucleoprotein D. isoform CRA_b OS=Rattus norvegicus GN=Hnrpd PE=4 SV=1
22.24	2	744.90	1487.79	-1.23	77	GILAADESTGSIAKR		P05065	Fructose-bisphosphate aldolase A OS=Rattus norvegicus GN=Aldoa PE=1 SV=2
22.24	2	744.90	1487.79	-1.23	77	GILAADESTGSIAKR		Q6AY07	Fructose-bisphosphate aldolase OS=Rattus norvegicus GN=Aldoart2 PE=2 SV=1
40.23	2	745.39	1488.77	-0.13	31	GVAGVPAEFSIWTR		D3ZHA0	Protein Flnc OS=Rattus norvegicus GN=Flnc PE=4 SV=1
13.86	2	745.86	1489.70	-2.59	47	LQSIGTENTEENR		P05065	Fructose-bisphosphate aldolase A OS=Rattus norvegicus GN=Aldoa PE=1 SV=2
13.86	2	745.86	1489.70	-2.59	47	LQSIGTENTEENR		Q6AY07	Fructose-bisphosphate aldolase OS=Rattus norvegicus GN=Aldoart2 PE=2 SV=1
24.35	2	746.38	1490.74	-1.15	71	TALINATGEEVAMR	[13] Oxidation (M)	G3V8L3	Lamin A. isoform CRA_b OS=Rattus norvegicus GN=Lmna PE=3 SV=1
48.58	2	746.40	1490.79	-0.92	43	SFPDFPIPGVLFR		P36972	Adenine phosphoribosyltransferase OS=Rattus norvegicus GN=Aprt PE=1 SV=1
8.27	2	746.86	1491.70	-1.84	45	IRDEMVATEQER	[5] Oxidation (M)	E9PSX6	Sarcoplasmic/endoplasmic reticulum calcium ATPase 2 OS=Rattus norvegicus GN=Atp2a2 PE=3 SV=2

26.21	2	748.40	1494.78	-1.20	26	TYSLGSALRPSTSR	P31000	Vimentin OS=Rattus norvegicus GN=Vim PE=1 SV=2
17.78	2	750.86	1499.70	-1.56	64	QEYDEAGPSIVHR	P68035	Actin. alpha cardiac muscle 1 OS=Rattus norvegicus GN=Actc1 PE=2 SV=1
17.78	2	750.86	1499.70	-1.56	64	QEYDEAGPSIVHR	P68136	Actin. alpha skeletal muscle OS=Rattus norvegicus GN=Acta1 PE=1 SV=1
36.09	2	751.34	1500.67	-0.67	68	DTGTYEDFVEGLR	P16409	Myosin light chain 3 OS=Rattus norvegicus GN=Myl3 PE=2 SV=2
11.15	2	751.86	1501.71	-1.83	35	SGQSAAGASPAGGIDTR	C0JPT7	Filamin alpha OS=Rattus norvegicus GN=Flna PE=2 SV=1
23.93	2	752.39	1502.76	-0.40	81	ENQSILITGESGAGK	G3V885	Myosin-6 OS=Rattus norvegicus GN=Myh6 PE=4 SV=1
23.93	2	752.39	1502.76	-0.40	81	ENQSILITGESGAGK	G3V6D8	Myosin-3 OS=Rattus norvegicus GN=Myh3 PE=4 SV=1
23.93	2	752.39	1502.76	-0.40	81	ENQSILITGESGAGK	F1LMU0	Myosin-4 OS=Rattus norvegicus GN=Myh4 PE=2 SV=1
23.93	2	752.39	1502.76	-0.40	81	ENQSILITGESGAGK	P02564	Myosin-7 OS=Rattus norvegicus GN=Myh7 PE=2 SV=2
23.93	2	752.39	1502.76	-0.40	81	ENQSILITGESGAGK	G3V8B0	Myosin-7 OS=Rattus norvegicus GN=Myh7 PE=4 SV=1
23.93	2	752.39	1502.76	-0.40	81	ENQSILITGESGAGK	F1M8F6	Myosin-8 (Fragment) OS=Rattus norvegicus GN=Myh8 PE=4 SV=2
23.93	2	752.39	1502.76	-0.40	81	ENQSILITGESGAGK	F1LRV9	Protein Myh1 OS=Rattus norvegicus GN=Myh1 PE=2 SV=2
23.93	2	752.39	1502.76	-0.40	81	ENQSILITGESGAGK	G3V6E1	Uncharacterized protein OS=Rattus norvegicus GN=Myh2 PE=4 SV=2
36.58	2	752.43	1502.86	-1.23	51	NLLSVAYKNVVGAR	P61983	14-3-3 protein gamma OS=Rattus norvegicus GN=Ywhag PE=1 SV=2
36.58	2	752.43	1502.86	-1.23	51	NLLSVAYKNVVGAR	P35213	14-3-3 protein beta/alpha OS=Rattus norvegicus GN=Ywhab PE=1 SV=3
36.58	2	752.43	1502.86	-1.23	51	NLLSVAYKNVVGAR	P63102	14-3-3 protein zeta/delta OS=Rattus norvegicus GN=Ywhaz PE=1 SV=1
21.20	2	752.82	1503.63	-1.46	107	YSGDFGADAQGAMSK	Q9QZ76	Myoglobin OS=Rattus norvegicus GN=Mb PE=1 SV=3
24.78	3	502.26	1503.76	-2.82	39	NLVHIITHGEEKD	P08733	Myosin regulatory light chain 2. ventricular/cardiac muscle isoform OS=Rattus norvegicus GN=Myl2 PE=1 SV=2

24.78	2	752.89	1503.77	-0.95	47	NLVHIITHGEEKD		P08733	Myosin regulatory light chain 2. ventricular/cardiac muscle isoform OS=Rattus norvegicus GN=Myl2 PE=1 SV=2
30.28	2	754.35	1506.69	-1.01	93	GGDDLDPNYVLSSR		P00564	Creatine kinase M-type OS=Rattus norvegicus GN=Ckm PE=1 SV=2
14.78	2	754.92	1507.83	-1.80	62	VLKQVHPDTGISSK		G3V8B3	Histone H2B OS=Rattus norvegicus GN=LOC684797 PE=3 SV=1
14.34	2	755.86	1509.71	-1.14	45	VKEGMSIVEAMER	[5] Oxidation (M)|[11] Oxidation (M)	P10111	Peptidyl-prolyl cis-trans isomerase A OS=Rattus norvegicus GN=Ppia PE=1 SV=2
37.03	2	756.39	1510.77	-0.59	68	SAYLMGLNSADLLK	[5] Oxidation (M)	G3V885	Myosin-6 OS=Rattus norvegicus GN=Myh6 PE=4 SV=1
37.03	2	756.39	1510.77	-0.59	68	SAYLMGLNSADLLK	[5] Oxidation (M)	P02564	Myosin-7 OS=Rattus norvegicus GN=Myh7 PE=2 SV=2
37.03	2	756.39	1510.77	-0.59	68	SAYLMGLNSADLLK	[5] Oxidation (M)	G3V8B0	Myosin-7 OS=Rattus norvegicus GN=Myh7 PE=4 SV=1
33.89	2	757.40	1512.78	-1.74	52	GVVDSEDLPLNISR		P34058	Heat shock protein HSP 90-beta OS=Rattus norvegicus GN=Hsp90ab1 PE=1 SV=4
33.89	2	757.40	1512.78	-1.74	52	GVVDSEDIPLNLSR		Q5XHZ0	Heat shock protein 75 kDa. mitochondrial OS=Rattus norvegicus GN=Trap1 PE=1 SV=1
33.89	2	757.40	1512.78	-1.74	52	GVVDSEDLPLNISR		P82995	Heat shock protein HSP 90-alpha OS=Rattus norvegicus GN=Hsp90aa1 PE=1 SV=3
16.30	2	757.89	1513.77	-1.73	27	ASTSQVPSQGTPNLK		M0R9L0	Protein Naca OS=Rattus norvegicus GN=Naca PE=4 SV=1
27.55	3	505.92	1514.74	-2.90	54	IWHHTFYNELR		P60711	Actin. cytoplasmic 1 OS=Rattus norvegicus GN=Actb PE=1 SV=1
27.55	3	505.92	1514.74	-2.90	54	IWHHTFYNELR		P68035	Actin. alpha cardiac muscle 1 OS=Rattus norvegicus GN=Actc1 PE=2 SV=1
27.55	3	505.92	1514.74	-2.90	54	IWHHTFYNELR		P68136	Actin. alpha skeletal muscle OS=Rattus norvegicus GN=Acta1 PE=1 SV=1
27.55	3	505.92	1514.74	-2.90	54	IWHHTFYNELR		M0R8W7	Uncharacterized protein OS=Rattus norvegicus PE=3 SV=1

27.55	2	758.38	1514.74	-1.05	37	IWHHTFYNELR		P60711	Actin. cytoplasmic 1 OS=Rattus norvegicus GN=Actb PE=1 SV=1
27.55	2	758.38	1514.74	-1.05	37	IWHHTFYNELR		P68035	Actin. alpha cardiac muscle 1 OS=Rattus norvegicus GN=Actc1 PE=2 SV=1
27.55	2	758.38	1514.74	-1.05	37	IWHHTFYNELR		P68136	Actin. alpha skeletal muscle OS=Rattus norvegicus GN=Acta1 PE=1 SV=1
27.55	2	758.38	1514.74	-1.05	37	IWHHTFYNELR		M0R8W7	Uncharacterized protein OS=Rattus norvegicus PE=3 SV=1
30.11	2	758.39	1514.77	-0.83	79	LLGSLDIDHNQYK		G3V885	Myosin-6 OS=Rattus norvegicus GN=Myh6 PE=4 SV=1
30.11	2	758.39	1514.77	-0.83	79	LLGSLDIDHNQYK		P02564	Myosin-7 OS=Rattus norvegicus GN=Myh7 PE=2 SV=2
30.11	2	758.39	1514.77	-0.83	79	LLGSLDIDHNQYK		G3V8B0	Myosin-7 OS=Rattus norvegicus GN=Myh7 PE=4 SV=1
20.64	2	758.40	1514.79	-1.84	60	EVQTNDLKEVVNK		P49242	40S ribosomal protein S3a OS=Rattus norvegicus GN=Rps3a PE=1 SV=2
20.64	2	758.40	1514.79	-1.84	60	EVQTNDLKEVVNK		Q6TXJ6	LRRGT00003 OS=Rattus norvegicus GN=LOC100362727 PE=2 SV=1
15.35	2	760.82	1519.62	-1.54	99	YSGDFGADAQGA MSK	[13] Oxidation (M)	Q9QZ76	Myoglobin OS=Rattus norvegicus GN=Mb PE=1 SV=3
9.31	2	762.39	1522.77	-2.22	62	IQDKEGIPPDQQR		F1LML2	Polyubiquitin-C OS=Rattus norvegicus GN=Ubc PE=2 SV=1
29.05	2	762.42	1522.82	-1.05	30	AATPYRVSIYGVAR		M0RA80	Protein Tnc OS=Rattus norvegicus GN=Tnc PE=4 SV=1
36.85	2	762.93	1523.85	-0.71	52	VLGGLPDVVTIQEGK		F1M7T8	Protein Myom1 OS=Rattus norvegicus GN=Myom1 PE=4 SV=2
10.88	2	764.35	1526.69	-2.15	26	MFGGSGTSSRPSSNR		P31000	Vimentin OS=Rattus norvegicus GN=Vim PE=1 SV=2
35.75	2	764.43	1526.84	0.10	34	LAAVDATVNQVLASR		Q63081	Protein disulfide-isomerase A6 OS=Rattus norvegicus GN=Pdia6 PE=1 SV=2
13.93	2	765.88	1529.75	-1.70	49	KVLGNPSNEEMNAK		P02600	Myosin light chain 1/3. skeletal muscle isoform OS=Rattus norvegicus GN=Myl1 PE=1 SV=2
34.84	2	766.45	1530.88	-0.60	47	VLALPEPSPAAPTLR		P30427	Plectin OS=Rattus norvegicus GN=Plec PE=1 SV=2
14.18	2	766.86	1531.71	0.55	66	AVTEQGAELSNEER		P68255	14-3-3 protein theta OS=Rattus norvegicus GN=Ywhaq PE=1 SV=1
31.55	2	766.91	1531.80	-1.17	74	VAVGEEQAFLVNTR		D3ZHA0	Protein Flnc OS=Rattus norvegicus GN=Flnc PE=4 SV=1

20.72	2	767.37	1532.72	-1.29	38	ATEEPSGTGSDELIK		G3V8L9	Polymerase I and transcript release factor OS=Rattus norvegicus GN=Ptrf PE=4 SV=1
10.05	2	767.37	1532.72	-1.85	103	VVANSPANADYQER		Q5XIG1	Ldb3 protein OS=Rattus norvegicus GN=Ldb3 PE=2 SV=1
27.96	2	767.39	1532.77	-0.96	80	VVDSLQTSLDAETR		G3V885	Myosin-6 OS=Rattus norvegicus GN=Myh6 PE=4 SV=1
27.96	2	767.39	1532.77	-0.96	80	VVDSLQTSLDAETR		P02564	Myosin-7 OS=Rattus norvegicus GN=Myh7 PE=2 SV=2
27.96	2	767.39	1532.77	-0.96	80	VVDSLQTSLDAETR		G3V8B0	Myosin-7 OS=Rattus norvegicus GN=Myh7 PE=4 SV=1
17.47	2	768.41	1534.80	-2.03	77	KSEIGIAMGSGTAV AK	[8] Oxidation (M)	E9PSX6	Sarcoplasmic/endoplasmic reticulum calcium ATPase 2 OS=Rattus norvegicus GN=Atp2a2 PE=3 SV=2
11.03	2	768.88	1535.76	-1.62	43	NKQTYSTEPNNLK		Q642E2	Protein LOC100362069 OS=Rattus norvegicus GN=Rpl28 PE=2 SV=1
7.87	2	769.86	1537.70	-2.04	33	AGPAPDHQAEASTVTS		Q5PQU1	Kininogen 1 OS=Rattus norvegicus GN=Kng1 PE=2 SV=1
33.48	2	770.37	1538.73	-1.15	56	VDSSNGFLIDGYPR		P39069	Adenylate kinase isoenzyme 1 OS=Rattus norvegicus GN=Ak1 PE=1 SV=3
39.02	3	514.95	1541.83	-3.66	52	LGFHLPLEVAYQR		P30427	Plectin OS=Rattus norvegicus GN=Plec PE=1 SV=2
39.02	2	771.92	1541.83	-0.84	38	LGFHLPLEVAYQR		P30427	Plectin OS=Rattus norvegicus GN=Plec PE=1 SV=2
42.90	2	771.93	1541.84	-1.79	39	GVDEVTIVNILTNR		Q07936	Annexin A2 OS=Rattus norvegicus GN=Anxa2 PE=1 SV=2
7.41	2	772.35	1542.68	-2.43	25	MFGGSGTSSRPSSN R	[1] Oxidation (M)	P31000	Vimentin OS=Rattus norvegicus GN=Vim PE=1 SV=2
42.12	2	772.44	1542.86	-0.73	41	GVDEATIIDILTKR		P07150	Annexin A1 OS=Rattus norvegicus GN=Anxa1 PE=1 SV=2
8.86	2	773.88	1545.74	-1.90	60	KVLGNPSNEEMNA K	[11] Oxidation (M)	P02600	Myosin light chain 1/3. skeletal muscle isoform OS=Rattus norvegicus GN=Myl1 PE=1 SV=2
22.74	2	773.93	1545.84	-2.38	41	QVASMTKPTTIIEK		P07483	Fatty acid-binding protein. heart OS=Rattus norvegicus GN=Fabp3 PE=1 SV=2
32.40	2	773.93	1545.84	0.16	55	VLEQLTGQTPVFSK		Q4V8I6	Ribosomal protein L11 OS=Rattus norvegicus GN=Rpl11 PE=2 SV=1
8.19	2	774.41	1546.80	-1.86	46	APGTPHSHTKPYVR		Q0QEW8	60S ribosomal protein L18 (Fragment) OS=Rattus norvegicus GN=Rpl18 PE=2 SV=1
15.26	2	774.86	1547.70	-1.76	91	SVTEQGAELSNEER		P63102	14-3-3 protein zeta/delta OS=Rattus norvegicus GN=Ywhaz PE=1 SV=1

26.05	3	516.94	1547.80	-2.31	28	MQKEITALAPSTMK		P60711	Actin. cytoplasmic 1 OS=Rattus norvegicus GN=Actb PE=1 SV=1
26.05	3	516.94	1547.80	-2.31	28	MQKEITALAPSTMK		P68035	Actin. alpha cardiac muscle 1 OS=Rattus norvegicus GN=Actc1 PE=2 SV=1
26.05	3	516.94	1547.80	-2.31	28	MQKEITALAPSTMK		P68136	Actin. alpha skeletal muscle OS=Rattus norvegicus GN=Acta1 PE=1 SV=1
26.05	2	774.91	1547.80	-1.19	77	MQKEITALAPSTMK		P60711	Actin. cytoplasmic 1 OS=Rattus norvegicus GN=Actb PE=1 SV=1
26.05	2	774.91	1547.80	-1.19	77	MQKEITALAPSTMK		P68035	Actin. alpha cardiac muscle 1 OS=Rattus norvegicus GN=Actc1 PE=2 SV=1
26.05	2	774.91	1547.80	-1.19	77	MQKEITALAPSTMK		P68136	Actin. alpha skeletal muscle OS=Rattus norvegicus GN=Acta1 PE=1 SV=1
13.93	2	775.34	1548.66	-1.11	42	YLMEEDEDAYKK	[3] Oxidation (M)	P09895	60S ribosomal protein L5 OS=Rattus norvegicus GN=Rpl5 PE=1 SV=3
13.93	2	775.34	1548.66	-1.11	42	YLMEEDEDAYKK	[3] Oxidation (M)	D3ZHP8	Protein Rpl5l1 OS=Rattus norvegicus GN=Rpl5l1 PE=3 SV=1
40.83	2	775.91	1549.81	-1.95	33	GTDVNVFNTILTTR		P07150	Annexin A1 OS=Rattus norvegicus GN=Anxa1 PE=1 SV=2
13.62	2	775.98	1549.94	-1.14	26	ALVKPQAVKPKMPK	[12] Oxidation (M)	M0R665	Protein LOC686807 OS=Rattus norvegicus GN=LOC686807 PE=4 SV=1
37.46	2	776.45	1550.89	-0.66	55	VLVPAETLEVAVSPK		M0R9L0	Protein Naca OS=Rattus norvegicus GN=Naca PE=4 SV=1
24.17	2	777.42	1552.82	-0.63	82	TLQEEHVTVTQLR		P30427	Plectin OS=Rattus norvegicus GN=Plec PE=1 SV=2
17.62	2	778.40	1554.78	-1.44	54	FVPQEMGPHTVAVK	[6] Oxidation (M)	D3ZHA0	Protein Flnc OS=Rattus norvegicus GN=Flnc PE=4 SV=1
38.15	2	779.42	1556.83	-0.28	47	AIGVSNFNPLQIER		P07943	Aldose reductase OS=Rattus norvegicus GN=Akr1b1 PE=1 SV=3
19.44	2	781.40	1560.78	-1.35	107	SQPVSAIATTAMGSPK	[12] Oxidation (M)	M0RA80	Protein Tnc OS=Rattus norvegicus GN=Tnc PE=4 SV=1
26.29	2	781.42	1560.82	-0.73	78	AMGVVVATGVNTEIGK	[2] Oxidation (M)	E9PSX6	Sarcoplasmic/endoplasmic reticulum calcium ATPase 2 OS=Rattus norvegicus GN=Atp2a2 PE=3 SV=2
34.76	2	781.42	1560.82	-1.31	84	VGEATETALTTLVEK		B4F7E5	ATPase. Ca++ transporting. cardiac muscle. fast twitch 1 OS=Rattus norvegicus GN=Atp2a1 PE=2 SV=1

34.76	2	781.42	1560.82	-1.31	84	VGEATETALTTLVEK		M0RCD2	Sarcoplasmic/endoplasmic reticulum calcium ATPase 1 OS=Rattus norvegicus GN=Atp2a1 PE=3 SV=1
47.41	2	781.91	1561.81	-0.52	64	TAGWNIPMGLLFSR		Q7TMC7	Ab2-417 OS=Rattus norvegicus GN=Tf PE=2 SV=1
23.36	2	782.88	1563.74	-1.11	36	SIEYSPQLEDANAK		F1LTJ5	Protein Hspg2 OS=Rattus norvegicus GN=Hspg2 PE=4 SV=2
15.58	2	782.89	1563.76	-1.49	99	ALAAGGYDVEKNNSR		D3ZBN0	Histone H1.5 OS=Rattus norvegicus GN=Hist1h1b PE=3 SV=1
28.81	2	782.90	1563.79	-1.67	39	RIGIFGQDEDVTSK		E9PSX6	Sarcoplasmic/endoplasmic reticulum calcium ATPase 2 OS=Rattus norvegicus GN=Atp2a2 PE=3 SV=2
21.20	2	782.91	1563.80	-1.15	90	MQKEITALAPSTMK	[13] Oxidation (M)	P60711	Actin. cytoplasmic 1 OS=Rattus norvegicus GN=Actb PE=1 SV=1
21.20	2	782.91	1563.80	-1.15	90	MQKEITALAPSTMK	[13] Oxidation (M)	P68035	Actin. alpha cardiac muscle 1 OS=Rattus norvegicus GN=Actc1 PE=2 SV=1
21.20	2	782.91	1563.80	-1.15	90	MQKEITALAPSTMK	[13] Oxidation (M)	P68136	Actin. alpha skeletal muscle OS=Rattus norvegicus GN=Acta1 PE=1 SV=1
23.93	2	782.91	1563.80	-0.32	90	MQKEITALAPSTMK	[1] Oxidation (M)	P60711	Actin. cytoplasmic 1 OS=Rattus norvegicus GN=Actb PE=1 SV=1
23.93	2	782.91	1563.80	-0.32	90	MQKEITALAPSTMK	[1] Oxidation (M)	P68035	Actin. alpha cardiac muscle 1 OS=Rattus norvegicus GN=Actc1 PE=2 SV=1
23.93	2	782.91	1563.80	-0.32	90	MQKEITALAPSTMK	[1] Oxidation (M)	P68136	Actin. alpha skeletal muscle OS=Rattus norvegicus GN=Acta1 PE=1 SV=1
37.73	2	782.96	1563.90	-0.67	36	SIVVSPILIPENQR		F1M7X3	Protein Cdh13 (Fragment) OS=Rattus norvegicus GN=Cdh13 PE=4 SV=2
10.88	2	783.86	1565.70	-0.55	32	AGEVINQPMMMAAR	[9] Oxidation (M)|[10] Oxidation (M)|[11] Oxidation (M)	R9PXU6	Vinculin OS=Rattus norvegicus GN=Vcl PE=4 SV=1
26.45	2	783.88	1565.74	-0.72	69	SVGGSGGGSFGDNLVTR		G3V8L3	Lamin A. isoform CRA_b OS=Rattus norvegicus GN=Lmna PE=3 SV=1
29.70	2	783.89	1565.76	-8.08	47	ITPSYVAFTPEGER		P06761	78 kDa glucose-regulated protein OS=Rattus norvegicus GN=Hspa5 PE=1 SV=1

29.70	2	783.89	1565.76	-0.93	76	DVFVPDDKEEFVK	P02564	Myosin-7 OS=Rattus norvegicus GN=Myh7 PE=2 SV=2
29.70	2	783.89	1565.76	-0.93	76	DVFVPDDKEEFVK	G3V8B0	Myosin-7 OS=Rattus norvegicus GN=Myh7 PE=4 SV=1
43.66	2	784.96	1567.90	-0.64	38	ALQLGTLFPPAEALK	Q68G41	Dodecenoyl-Coenzyme A delta isomerase (3.2 trans-enoyl-Coenzyme A isomerase) OS=Rattus norvegicus GN=Eci1 PE=2 SV=1
25.19	2	785.39	1568.76	-1.64	47	KAEAGAGSATEFQFR	F1LT36	Protein RGD1564698 OS=Rattus norvegicus GN=RGD1564698 PE=4 SV=2
28.16	2	785.91	1569.80	-1.44	79	GAGTGGLGLAVEGPSEAK	C0JPT7	Filamin alpha OS=Rattus norvegicus GN=Flna PE=2 SV=1
45.67	2	785.95	1569.89	-0.84	74	ISLPLPNFSSLNLR	P31000	Vimentin OS=Rattus norvegicus GN=Vim PE=1 SV=2
27.29	2	786.44	1570.87	-0.68	55	SLQDLQLANNKISK	P51886	Lumican OS=Rattus norvegicus GN=Lum PE=2 SV=1
17.32	3	524.92	1571.73	-3.22	57	IGGHGGEYGEEALQR	B1H216	Hemoglobin alpha. adult chain 2 OS=Rattus norvegicus GN=Hba1 PE=2 SV=1
17.32	2	786.87	1571.73	-1.35	73	IGGHGGEYGEEALQR	B1H216	Hemoglobin alpha. adult chain 2 OS=Rattus norvegicus GN=Hba1 PE=2 SV=1
25.59	2	787.40	1572.80	-1.18	41	GGKPEPPAMPQPVPTA	P62909	40S ribosomal protein S3 OS=Rattus norvegicus GN=Rps3 PE=1 SV=1
24.01	2	787.92	1573.82	-0.71	38	FLSAGREDPTQVVR	D3ZUL3	Protein Col6a1 OS=Rattus norvegicus GN=Col6a1 PE=4 SV=1
33.89	2	787.93	1573.85	-1.22	74	VDQSILTGESVSVIK	E9PSX6	Sarcoplasmic/endoplasmic reticulum calcium ATPase 2 OS=Rattus norvegicus GN=Atp2a2 PE=3 SV=2
33.89	2	787.93	1573.85	-1.22	74	VDQSILTGESVSVIK	B4F7E5	ATPase. Ca++ transporting. cardiac muscle. fast twitch 1 OS=Rattus norvegicus GN=Atp2a1 PE=2 SV=1
33.89	2	787.93	1573.85	-1.22	74	VDQSILTGESVSVIK	M0RCD2	Sarcoplasmic/endoplasmic reticulum calcium ATPase 1 OS=Rattus norvegicus GN=Atp2a1 PE=3 SV=1
29.46	2	788.40	1574.78	-1.66	57	ILGADTSVDLEETGR	M0R4D7	Uncharacterized protein OS=Rattus norvegicus PE=3 SV=1
16.54	3	526.93	1577.77	-3.32	27	ALAAAGYDVEKNNSR	P15865	Histone H1.4 OS=Rattus norvegicus GN=Hist1h1e PE=1 SV=3
16.54	3	526.93	1577.77	-3.32	27	ALAAAGYDVEKNNSR	P06349	Histone H1t OS=Rattus norvegicus GN=Hist1h1t PE=1 SV=2

16.54	3	526.93	1577.77	-3.32	27	ALAAAGYDVEKNNSR		M0R7B4	Protein LOC684828 OS=Rattus norvegicus GN=LOC684828 PE=3 SV=1
16.54	2	789.90	1577.78	-1.39	69	ALAAAGYDVEKNNSR		P15865	Histone H1.4 OS=Rattus norvegicus GN=Hist1h1e PE=1 SV=3
16.54	2	789.90	1577.78	-1.39	69	ALAAAGYDVEKNNSR		P06349	Histone H1t OS=Rattus norvegicus GN=Hist1h1t PE=1 SV=2
16.54	2	789.90	1577.78	-1.39	69	ALAAAGYDVEKNNSR		M0R7B4	Protein LOC684828 OS=Rattus norvegicus GN=LOC684828 PE=3 SV=1
18.71	3	527.60	1579.79	-2.46	37	MQKEITALAPSTMK	[1] Oxidation (M)|[13] Oxidation (M)	P60711	Actin. cytoplasmic 1 OS=Rattus norvegicus GN=Actb PE=1 SV=1
18.71	3	527.60	1579.79	-2.46	37	MQKEITALAPSTMK	[1] Oxidation (M)|[13] Oxidation (M)	P68035	Actin. alpha cardiac muscle 1 OS=Rattus norvegicus GN=Actc1 PE=2 SV=1
18.71	3	527.60	1579.79	-2.46	37	MQKEITALAPSTMK	[1] Oxidation (M)|[13] Oxidation (M)	P68136	Actin. alpha skeletal muscle OS=Rattus norvegicus GN=Acta1 PE=1 SV=1
18.71	2	790.90	1579.79	-1.18	81	MQKEITALAPSTMK	[1] Oxidation (M)|[13] Oxidation (M)	P60711	Actin. cytoplasmic 1 OS=Rattus norvegicus GN=Actb PE=1 SV=1
18.71	2	790.90	1579.79	-1.18	81	MQKEITALAPSTMK	[1] Oxidation (M)|[13] Oxidation (M)	P68035	Actin. alpha cardiac muscle 1 OS=Rattus norvegicus GN=Actc1 PE=2 SV=1
18.71	2	790.90	1579.79	-1.18	81	MQKEITALAPSTMK	[1] Oxidation (M)|[13] Oxidation (M)	P68136	Actin. alpha skeletal muscle OS=Rattus norvegicus GN=Acta1 PE=1 SV=1
40.06	2	791.95	1581.88	-0.50	61	SPFVVNVAPPLDLSK		D3ZHA0	Protein Flnc OS=Rattus norvegicus GN=Flnc PE=4 SV=1
25.02	2	792.87	1583.73	-1.45	47	DHASIQMNVAEVDR		P05765	40S ribosomal protein S21 OS=Rattus norvegicus GN=Rps21 PE=1 SV=1
31.37	2	792.91	1583.81	-0.94	102	GAGTGGLGLAIEGPSEAK		D3ZHA0	Protein Flnc OS=Rattus norvegicus GN=Flnc PE=4 SV=1
8.62	2	792.98	1583.94	-1.81	31	LSAKPAPPKPEPKPK		Q4KLJ0	High mobility group nucleosomal binding domain 2 OS=Rattus norvegicus GN=Hmgn2 PE=4 SV=1

28.25	2	793.39	1584.77	-1.11	53	INFDKYHPGYFGK		D3ZF07	Protein RGD1562402 OS=Rattus norvegicus GN=RGD1562402 PE=3 SV=1
28.25	2	793.39	1584.77	-1.11	53	INFDKYHPGYFGK		F1M5H4	Uncharacterized protein OS=Rattus norvegicus PE=3 SV=2
20.15	3	529.28	1584.81	-2.43	43	IEDVTPIPSDSTRR		Q6PDV6	40S ribosomal protein S14 OS=Rattus norvegicus GN=Rps14 PE=2 SV=1
20.15	2	793.41	1584.81	-1.18	34	IEDVTPIPSDSTRR		Q6PDV6	40S ribosomal protein S14 OS=Rattus norvegicus GN=Rps14 PE=2 SV=1
34.16	2	793.43	1584.85	3.07	66	GQTVEQVTNAVGALAK		G3V6E1	Uncharacterized protein OS=Rattus norvegicus GN=Myh2 PE=4 SV=2
16.93	2	795.40	1588.79	-1.94	35	GGKPEPPAMPQPVPTA	[9] Oxidation (M)	P62909	40S ribosomal protein S3 OS=Rattus norvegicus GN=Rps3 PE=1 SV=1
20.88	3	530.99	1589.93	-2.13	41	DGLIIRKPVTVHSR		P84100	60S ribosomal protein L19 OS=Rattus norvegicus GN=Rpl19 PE=1 SV=1
20.88	2	795.98	1589.94	-0.55	28	DGLIIRKPVTVHSR		P84100	60S ribosomal protein L19 OS=Rattus norvegicus GN=Rpl19 PE=1 SV=1
16.30	2	797.38	1592.75	-1.60	57	SEETLDEGPPKYTK		G3V6L9	Peptidyl-prolyl cis-trans isomerase OS=Rattus norvegicus GN=Fkbp3 PE=4 SV=1
47.35	2	797.41	1592.80	-0.71	64	DLFEDELVPLFEK		D3ZF34	Uncharacterized protein (Fragment) OS=Rattus norvegicus PE=4 SV=2
17.70	2	797.43	1592.84	-1.34	44	EATNPPIIQEEKPK		P11598	Protein disulfide-isomerase A3 OS=Rattus norvegicus GN=Pdia3 PE=1 SV=2
13.56	2	797.88	1593.74	-1.91	26	HSGPSSYKVGTMSEK		Q6P792	Four and a half LIM domains 1 OS=Rattus norvegicus GN=Fhl1 PE=2 SV=1
27.69	2	798.90	1595.78	-0.60	37	QSEPQEITLPSPDR		M0RA80	Protein Tnc OS=Rattus norvegicus GN=Tnc PE=4 SV=1
19.91	2	799.87	1597.73	-1.53	38	TSMDMLVLEDEKR	[3] Oxidation (M)|[5] Oxidation (M)	D3ZM27	Ankyrin repeat domain 2 (Stretch responsive muscle) (Predicted) OS=Rattus norvegicus GN=Ankrd2 PE=4 SV=1
9.31	2	799.87	1597.73	-1.45	73	AVTEQGHELSNEER		P35213	14-3-3 protein beta/alpha OS=Rattus norvegicus GN=Ywhab PE=1 SV=3
34.58	2	800.89	1599.77	-0.91	31	VVFDDTFDRSMLR		P14141	Carbonic anhydrase 3 OS=Rattus norvegicus GN=Ca3 PE=1 SV=3

48.36	2	800.93	1599.85	-0.48	36	GELVPLETVLDMLR	[12] Oxidation (M)	P39069	Adenylate kinase isoenzyme 1 OS=Rattus norvegicus GN=Ak1 PE=1 SV=3
24.51	2	803.41	1604.80	-0.01	65	VAVEEVDEEGKFVR		G3V8L3	Lamin A. isoform CRA_b OS=Rattus norvegicus GN=Lmna PE=3 SV=1
29.54	2	804.91	1607.81	-0.62	60	VLSNMGAHFGGYL VK	[5] Oxidation (M)	M0R799	Troponin T. slow skeletal muscle OS=Rattus norvegicus GN=Tnnt1 PE=4 SV=1
8.02	2	805.88	1609.74	-1.14	44	HSGPSSYKVGTMSE K	[12] Oxidation (M)	Q6P792	Four and a half LIM domains 1 OS=Rattus norvegicus GN=Fhl1 PE=2 SV=1
33.39	2	807.39	1612.77	-0.88	44	MGPLGLDHMASSIER		F1LV13	Heterogeneous nuclear ribonucleoprotein M OS=Rattus norvegicus GN=Hnrnpm PE=2 SV=1
37.64	2	808.42	1614.83	-0.73	34	AILVDLEPGTMDSVR		P85108	Tubulin beta-2A chain OS=Rattus norvegicus GN=Tubb2a PE=1 SV=1
37.64	2	808.42	1614.83	-0.73	34	AILVDLEPGTMDSVR		Q4QRB4	Tubulin beta-3 chain OS=Rattus norvegicus GN=Tubb3 PE=1 SV=1
37.64	2	808.42	1614.83	-0.73	34	AILVDLEPGTMDSVR		P69897	Tubulin beta-5 chain OS=Rattus norvegicus GN=Tubb5 PE=1 SV=1
30.61	3	539.59	1615.76	-2.59	26	VVFDDTFDRSMLR	[11] Oxidation (M)	P14141	Carbonic anhydrase 3 OS=Rattus norvegicus GN=Ca3 PE=1 SV=3
30.61	2	808.89	1615.77	-0.60	47	VVFDDTFDRSMLR	[11] Oxidation (M)	P14141	Carbonic anhydrase 3 OS=Rattus norvegicus GN=Ca3 PE=1 SV=3
32.73	2	809.41	1616.81	5.70	30	VALVYGQMNEPPG AR	[8] Oxidation (M)	G3V6D3	ATP synthase subunit beta OS=Rattus norvegicus GN=Atp5b PE=3 SV=1
32.73	2	809.41	1616.81	-0.42	61	AVLVDLEPGTMDS VR	[11] Oxidation (M)	B4F7C2	Protein Tubb4a OS=Rattus norvegicus GN=Tubb4a PE=2 SV=1
32.73	2	809.41	1616.81	-0.42	61	AVLVDLEPGTMDS VR	[11] Oxidation (M)	G3V7C6	RCG45400 OS=Rattus norvegicus GN=Tubb4b PE=3 SV=1
38.24	2	809.42	1616.82	-0.26	27	AGLLGLLEEMRDER	[10] Oxidation (M)	G3V885	Myosin-6 OS=Rattus norvegicus GN=Myh6 PE=4 SV=1
38.24	2	809.42	1616.82	-0.26	27	AGLLGLLEEMRDER	[10] Oxidation (M)	P02564	Myosin-7 OS=Rattus norvegicus GN=Myh7 PE=2 SV=2
38.24	2	809.42	1616.82	-0.26	27	AGLLGLLEEMRDER	[10] Oxidation (M)	G3V8B0	Myosin-7 OS=Rattus norvegicus GN=Myh7 PE=4 SV=1

42.34	3	540.95	1619.82	-2.75	34	LHFFMPGFAPLTSR		B4F7C2	Protein Tubb4a OS=Rattus norvegicus GN=Tubb4a PE=2 SV=1
42.34	3	540.95	1619.82	-2.75	34	LHFFMPGFAPLTSR		G3V7C6	RCG45400 OS=Rattus norvegicus GN=Tubb4b PE=3 SV=1
42.34	3	540.95	1619.82	-2.75	34	LHFFMPGFAPLTSR		P85108	Tubulin beta-2A chain OS=Rattus norvegicus GN=Tubb2a PE=1 SV=1
42.34	3	540.95	1619.82	-2.75	34	LHFFMPGFAPLTSR		P69897	Tubulin beta-5 chain OS=Rattus norvegicus GN=Tubb5 PE=1 SV=1
42.34	2	810.92	1619.83	-0.86	53	LHFFMPGFAPLTSR		B4F7C2	Protein Tubb4a OS=Rattus norvegicus GN=Tubb4a PE=2 SV=1
42.34	2	810.92	1619.83	-0.86	53	LHFFMPGFAPLTSR		G3V7C6	RCG45400 OS=Rattus norvegicus GN=Tubb4b PE=3 SV=1
42.34	2	810.92	1619.83	-0.86	53	LHFFMPGFAPLTSR		P85108	Tubulin beta-2A chain OS=Rattus norvegicus GN=Tubb2a PE=1 SV=1
42.34	2	810.92	1619.83	-0.86	53	LHFFMPGFAPLTSR		P69897	Tubulin beta-5 chain OS=Rattus norvegicus GN=Tubb5 PE=1 SV=1
25.92	2	810.94	1619.86	-0.97	60	VGSLDNVGHLPAGGTVK		Q5M7W5	Microtubule-associated protein 4 OS=Rattus norvegicus GN=Map4 PE=1 SV=1
38.41	2	812.42	1622.83	-0.42	30	LDLAGRDLTDYLMK		P60711	Actin. cytoplasmic 1 OS=Rattus norvegicus GN=Actb PE=1 SV=1
38.41	2	812.42	1622.83	-0.42	30	LDLAGRDLTDYLMK		P68035	Actin. alpha cardiac muscle 1 OS=Rattus norvegicus GN=Actc1 PE=2 SV=1
38.41	2	812.42	1622.83	-0.42	30	LDLAGRDLTDYLMK		P68136	Actin. alpha skeletal muscle OS=Rattus norvegicus GN=Acta1 PE=1 SV=1
38.41	2	812.42	1622.83	-0.42	30	LDLAGRDLTDYLMK		D3ZRN3	Protein Actbl2 OS=Rattus norvegicus GN=Actbl2 PE=3 SV=1
18.41	2	812.43	1622.85	-1.11	49	EQIVPKPEEEVAQK		P24049	60S ribosomal protein L17 OS=Rattus norvegicus GN=Rpl17 PE=2 SV=3
23.36	2	813.91	1625.80	-1.00	36	LTGAIMHYGNMKFK	[6] Oxidation (M)	G3V885	Myosin-6 OS=Rattus norvegicus GN=Myh6 PE=4 SV=1
23.36	2	813.91	1625.80	-1.00	34	LTGAIMHFGNMKFK	[6] Oxidation (M)|[11] Oxidation (M)	P02564	Myosin-7 OS=Rattus norvegicus GN=Myh7 PE=2 SV=2

23.36	2	813.91	1625.80	-1.00	34	LTGAIMHFGNMKFK	[6] Oxidation (M)|[11] Oxidation (M)	G3V8B0	Myosin-7 OS=Rattus norvegicus GN=Myh7 PE=4 SV=1
46.42	3	542.95	1625.83	-1.68	27	DLTDYLMKILTER	[7] Oxidation (M)	P60711	Actin. cytoplasmic 1 OS=Rattus norvegicus GN=Actb PE=1 SV=1
46.42	3	542.95	1625.83	-1.68	27	DLTDYLMKILTER	[7] Oxidation (M)	P68035	Actin. alpha cardiac muscle 1 OS=Rattus norvegicus GN=Actc1 PE=2 SV=1
46.42	3	542.95	1625.83	-1.68	27	DLTDYLMKILTER	[7] Oxidation (M)	P68136	Actin. alpha skeletal muscle OS=Rattus norvegicus GN=Acta1 PE=1 SV=1
46.42	3	542.95	1625.83	-1.68	27	DLTDYLMKILTER	[7] Oxidation (M)	D3ZRN3	Protein Actbl2 OS=Rattus norvegicus GN=Actbl2 PE=3 SV=1
46.42	2	813.92	1625.83	-0.36	47	DLTDYLMKILTER	[7] Oxidation (M)	P60711	Actin. cytoplasmic 1 OS=Rattus norvegicus GN=Actb PE=1 SV=1
46.42	2	813.92	1625.83	-0.36	47	DLTDYLMKILTER	[7] Oxidation (M)	P68035	Actin. alpha cardiac muscle 1 OS=Rattus norvegicus GN=Actc1 PE=2 SV=1
46.42	2	813.92	1625.83	-0.36	47	DLTDYLMKILTER	[7] Oxidation (M)	P68136	Actin. alpha skeletal muscle OS=Rattus norvegicus GN=Acta1 PE=1 SV=1
46.42	2	813.92	1625.83	-0.36	47	DLTDYLMKILTER	[7] Oxidation (M)	D3ZRN3	Protein Actbl2 OS=Rattus norvegicus GN=Actbl2 PE=3 SV=1
15.75	3	543.60	1627.79	-3.46	27	QEYDEAGPSIVHRK		P68035	Actin. alpha cardiac muscle 1 OS=Rattus norvegicus GN=Actc1 PE=2 SV=1
15.75	3	543.60	1627.79	-3.46	27	QEYDEAGPSIVHRK		P68136	Actin. alpha skeletal muscle OS=Rattus norvegicus GN=Acta1 PE=1 SV=1
15.75	2	814.90	1627.79	-1.25	66	QEYDEAGPSIVHRK		P68035	Actin. alpha cardiac muscle 1 OS=Rattus norvegicus GN=Actc1 PE=2 SV=1
15.75	2	814.90	1627.79	-1.25	66	QEYDEAGPSIVHRK		P68136	Actin. alpha skeletal muscle OS=Rattus norvegicus GN=Acta1 PE=1 SV=1
15.02	2	815.90	1629.78	2.30	40	NTTPTGATPPAGMASTR		Q5M7W5	Microtubule-associated protein 4 OS=Rattus norvegicus GN=Map4 PE=1 SV=1
15.02	2	815.90	1629.78	-2.55	83	IQSQTVGQGSDAHFR		D4A4B0	Uncharacterized protein (Fragment) OS=Rattus norvegicus PE=4 SV=2
38.50	2	815.95	1629.88	-0.08	96	GQNVQQVAYAIGALAK		P02564	Myosin-7 OS=Rattus norvegicus GN=Myh7 PE=2 SV=2

38.50	2	815.95	1629.88	-0.08	96	GQNVQQVAYAIGALAK		G3V8B0	Myosin-7 OS=Rattus norvegicus GN=Myh7 PE=4 SV=1
34.67	2	816.42	1630.82	-0.89	73	AILVDLEPGTMDSVR	[11] Oxidation (M)	P85108	Tubulin beta-2A chain OS=Rattus norvegicus GN=Tubb2a PE=1 SV=1
34.67	2	816.42	1630.82	-0.89	73	AILVDLEPGTMDSVR	[11] Oxidation (M)	Q4QRB4	Tubulin beta-3 chain OS=Rattus norvegicus GN=Tubb3 PE=1 SV=1
34.67	2	816.42	1630.82	-0.89	73	AILVDLEPGTMDSVR	[11] Oxidation (M)	P69897	Tubulin beta-5 chain OS=Rattus norvegicus GN=Tubb5 PE=1 SV=1
33.15	2	816.89	1631.77	-0.60	79	SFYPEEVSSMVLTK	[10] Oxidation (M)	P63018	Heat shock cognate 71 kDa protein OS=Rattus norvegicus GN=Hspa8 PE=1 SV=1
26.13	2	816.91	1631.80	-0.61	43	IPEGEKVDFDDIQK		F1LPQ5	Troponin T. fast skeletal muscle (Fragment) OS=Rattus norvegicus GN=Tnnt3 PE=4 SV=2
44.59	2	817.46	1632.90	-1.05	101	INLPIQTFSALNFR		Q6P725	Desmin OS=Rattus norvegicus GN=Des PE=2 SV=1
24.78	2	818.88	1635.74	-0.59	40	MDATANDVPSPYEVK		P11598	Protein disulfide-isomerase A3 OS=Rattus norvegicus GN=Pdia3 PE=1 SV=2
38.24	2	818.92	1635.82	-0.12	37	LHFFMPGFAPLTSR	[5] Oxidation (M)	B4F7C2	Protein Tubb4a OS=Rattus norvegicus GN=Tubb4a PE=2 SV=1
38.24	2	818.92	1635.82	-0.12	37	LHFFMPGFAPLTSR	[5] Oxidation (M)	G3V7C6	RCG45400 OS=Rattus norvegicus GN=Tubb4b PE=3 SV=1
38.24	2	818.92	1635.82	-0.12	37	LHFFMPGFAPLTSR	[5] Oxidation (M)	P85108	Tubulin beta-2A chain OS=Rattus norvegicus GN=Tubb2a PE=1 SV=1
38.24	2	818.92	1635.82	-0.12	37	LHFFMPGFAPLTSR	[5] Oxidation (M)	P69897	Tubulin beta-5 chain OS=Rattus norvegicus GN=Tubb5 PE=1 SV=1
16.30	2	819.36	1636.71	-1.40	94	LEDEEEMNAELTAK	[7] Oxidation (M)	G3V885	Myosin-6 OS=Rattus norvegicus GN=Myh6 PE=4 SV=1
16.30	2	819.36	1636.71	-1.40	94	LEDEEEMNAELTAK	[7] Oxidation (M)	P02564	Myosin-7 OS=Rattus norvegicus GN=Myh7 PE=2 SV=2
16.30	2	819.36	1636.71	-1.40	94	LEDEEEMNAELTAK	[7] Oxidation (M)	G3V8B0	Myosin-7 OS=Rattus norvegicus GN=Myh7 PE=4 SV=1
35.51	2	820.42	1638.83	-0.79	42	LDLAGRDLTDYLMK	[13] Oxidation (M)	P60711	Actin. cytoplasmic 1 OS=Rattus norvegicus GN=Actb PE=1 SV=1
35.51	2	820.42	1638.83	-0.79	42	LDLAGRDLTDYLMK	[13] Oxidation (M)	P68035	Actin. alpha cardiac muscle 1 OS=Rattus norvegicus GN=Actc1 PE=2 SV=1

35.51	2	820.42	1638.83	-0.79	42	LDLAGRDLTDYLMK	[13] Oxidation (M)	P68136	Actin. alpha skeletal muscle OS=Rattus norvegicus GN=Acta1 PE=1 SV=1
35.51	2	820.42	1638.83	-0.79	42	LDLAGRDLTDYLMK	[13] Oxidation (M)	D3ZRN3	Protein Actbl2 OS=Rattus norvegicus GN=Actbl2 PE=3 SV=1
24.01	2	822.40	1642.78	-1.06	73	NVTELNEPLSNEER		P61983	14-3-3 protein gamma OS=Rattus norvegicus GN=Ywhag PE=1 SV=2
25.92	2	822.46	1642.91	-0.94	43	VVTRGPGLSQAFVGQK		D3ZHA0	Protein Flnc OS=Rattus norvegicus GN=Flnc PE=4 SV=1
14.86	2	822.90	1643.79	-1.76	38	QEYDESGPSIVHRK		P60711	Actin. cytoplasmic 1 OS=Rattus norvegicus GN=Actb PE=1 SV=1
22.41	2	823.40	1644.79	-0.49	60	KYEMFAQTLQQSR	[4] Oxidation (M)	P46462	Transitional endoplasmic reticulum ATPase OS=Rattus norvegicus GN=Vcp PE=1 SV=3
39.36	2	823.44	1644.87	-0.58	47	LKGADPEETILNAFK		P08733	Myosin regulatory light chain 2. ventricular/cardiac muscle isoform OS=Rattus norvegicus GN=Myl2 PE=1 SV=2
7.23	2	823.89	1645.77	-2.51	58	NTTPTGATPPAGMASTR	[13] Oxidation (M)	Q5M7W5	Microtubule-associated protein 4 OS=Rattus norvegicus GN=Map4 PE=1 SV=1
35.51	2	823.90	1645.79	-0.27	68	SFYPEEISSMVLTK	[10] Oxidation (M)	Q07439	Heat shock 70 kDa protein 1A/1B OS=Rattus norvegicus GN=Hspa1a PE=2 SV=2
12.32	3	549.61	1645.79	-4.19	28	LQSIGTENTEENRR		P05065	Fructose-bisphosphate aldolase A OS=Rattus norvegicus GN=Aldoa PE=1 SV=2
12.32	3	549.61	1645.79	-4.19	28	LQSIGTENTEENRR		Q6AY07	Fructose-bisphosphate aldolase OS=Rattus norvegicus GN=Aldoart2 PE=2 SV=1
28.89	2	825.40	1648.79	-1.05	31	NQVAMNPTNTVFDAK		P63018	Heat shock cognate 71 kDa protein OS=Rattus norvegicus GN=Hspa8 PE=1 SV=1
28.89	2	825.40	1648.79	-1.05	31	NQVAMNPTNTVFDAK		D3ZH98	Uncharacterized protein OS=Rattus norvegicus PE=3 SV=1
22.24	2	826.88	1651.74	-0.21	80	MDATANDVPSPYEVK	[1] Oxidation (M)	P11598	Protein disulfide-isomerase A3 OS=Rattus norvegicus GN=Pdia3 PE=1 SV=2
18.49	2	826.89	1651.76	-1.08	59	IFRDGEEAGAYDGPR		P11598	Protein disulfide-isomerase A3 OS=Rattus norvegicus GN=Pdia3 PE=1 SV=2
30.45	3	552.31	1653.92	-2.33	42	VYNVTQHAVGIIVNK		D3ZPN7	Protein LOC100360604 OS=Rattus norvegicus GN=LOC100364176 PE=4 SV=1

30.45	3	552.31	1653.92	-2.33	42	VYNVTQHAVGIIVNK		M0RCS9	Uncharacterized protein (Fragment) OS=Rattus norvegicus PE=4 SV=1
30.45	3	552.31	1653.92	-2.33	42	VYNVTQHAVGIIVNK		D3ZRA9	Uncharacterized protein OS=Rattus norvegicus GN=RGD1560414 PE=4 SV=1
30.45	2	827.97	1653.92	-0.87	70	VYNVTQHAVGIIVNK		D3ZPN7	Protein LOC100360604 OS=Rattus norvegicus GN=LOC100364176 PE=4 SV=1
30.45	2	827.97	1653.92	-0.87	70	VYNVTQHAVGIIVNK		M0RCS9	Uncharacterized protein (Fragment) OS=Rattus norvegicus PE=4 SV=1
30.45	2	827.97	1653.92	-0.87	70	VYNVTQHAVGIIVNK		D3ZRA9	Uncharacterized protein OS=Rattus norvegicus GN=RGD1560414 PE=4 SV=1
36.32	2	828.38	1654.75	-0.11	66	DSLLQDGEFTMDLR	[11] Oxidation (M)	P62963	Profilin-1 OS=Rattus norvegicus GN=Pfn1 PE=1 SV=2
38.24	2	830.40	1658.79	-0.51	64	LYGPSSVSFADDFVR		Q5RJR9	Serine (Or cysteine) proteinase inhibitor. clade H. member 1. isoform CRA_b OS=Rattus norvegicus GN=Serpinh1 PE=2 SV=1
40.92	2	830.45	1658.89	-0.52	53	ALTVPELTQQVFDAK		P69897	Tubulin beta-5 chain OS=Rattus norvegicus GN=Tubb5 PE=1 SV=1
51.72	2	830.47	1658.93	-0.81	38	IEQLSPFPFDLLLK		Q5XI78	2-oxoglutarate dehydrogenase. mitochondrial OS=Rattus norvegicus GN=Ogdh PE=1 SV=1
23.68	2	833.40	1664.78	-1.53	99	NQVAMNPTNTVFDAK	[5] Oxidation (M)	P63018	Heat shock cognate 71 kDa protein OS=Rattus norvegicus GN=Hspa8 PE=1 SV=1
23.68	2	833.40	1664.78	-1.53	99	NQVAMNPTNTVFDAK	[5] Oxidation (M)	D3ZH98	Uncharacterized protein OS=Rattus norvegicus PE=3 SV=1
38.67	2	833.99	1665.97	-0.68	36	QAQIEVVPSASALIIK		B2RYU2	RCG45615. isoform CRA_a OS=Rattus norvegicus GN=Rpl12 PE=2 SV=1
25.51	2	834.91	1667.81	-1.89	32	YAPISGGDHAEIDVPK		M0RA80	Protein Tnc OS=Rattus norvegicus GN=Tnc PE=4 SV=1
35.59	2	836.91	1671.80	0.06	51	EATEYEIELYGISR		M0RA80	Protein Tnc OS=Rattus norvegicus GN=Tnc PE=4 SV=1
26.62	2	836.93	1671.85	-0.18	39	LEKGQSIDDMIPAQK		P00564	Creatine kinase M-type OS=Rattus norvegicus GN=Ckm PE=1 SV=2
23.20	2	837.90	1673.79	-0.81	55	VERADGYEPPVQESV		P49242	40S ribosomal protein S3a OS=Rattus norvegicus GN=Rps3a PE=1 SV=2

28.16	2	841.39	1680.76	-0.28	33	TSFDEMLPGTHFQR	[6] Oxidation (M)	Q5XI78	2-oxoglutarate dehydrogenase. mitochondrial OS=Rattus norvegicus GN=Ogdh PE=1 SV=1
31.80	2	841.93	1681.85	-1.46	32	ETNLESLPLVDTHSK		P31000	Vimentin OS=Rattus norvegicus GN=Vim PE=1 SV=2
25.35	3	562.64	1684.91	-2.56	48	VTTTRLDAPSQIEVR		M0RA80	Protein Tnc OS=Rattus norvegicus GN=Tnc PE=4 SV=1
25.43	2	843.46	1684.91	-1.17	50	VTTTRLDAPSQIEVR		M0RA80	Protein Tnc OS=Rattus norvegicus GN=Tnc PE=4 SV=1
46.36	2	844.45	1686.88	-0.65	106	GLGTDEDSILNLLTAR		Q66HH8	Annexin OS=Rattus norvegicus GN=Anxa5 PE=2 SV=1
18.71	3	563.61	1687.81	-3.10	30	ASGNYATVISHNPETK		P62919	60S ribosomal protein L8 OS=Rattus norvegicus GN=Rpl8 PE=2 SV=2
18.71	2	844.91	1687.81	-1.52	61	ASGNYATVISHNPETK		P62919	60S ribosomal protein L8 OS=Rattus norvegicus GN=Rpl8 PE=2 SV=2
18.02	2	844.93	1687.84	-2.10	78	LEKGQSIDDMIPAQK	[10] Oxidation (M)	P00564	Creatine kinase M-type OS=Rattus norvegicus GN=Ckm PE=1 SV=2
36.76	2	845.93	1689.85	-0.29	101	NLTEEMAGLDEIIVK	[6] Oxidation (M)	P02564	Myosin-7 OS=Rattus norvegicus GN=Myh7 PE=2 SV=2
36.76	2	845.93	1689.85	-0.29	101	NLTEEMAGLDEIIVK	[6] Oxidation (M)	G3V8B0	Myosin-7 OS=Rattus norvegicus GN=Myh7 PE=4 SV=1
17.78	2	846.37	1690.72	-1.41	61	STAGDTHLGGEDFDNR		P63018	Heat shock cognate 71 kDa protein OS=Rattus norvegicus GN=Hspa8 PE=1 SV=1
41.34	2	846.44	1690.86	-1.00	75	ALTVPELTQQMFDAK		B4F7C2	Protein Tubb4a OS=Rattus norvegicus GN=Tubb4a PE=2 SV=1
41.34	2	846.44	1690.86	-1.00	75	ALTVPELTQQMFDAK		Q4QQV0	Protein Tubb6 OS=Rattus norvegicus GN=Tubb6 PE=2 SV=1
41.34	2	846.44	1690.86	-1.00	75	ALTVPELTQQMFDAK		G3V7C6	RCG45400 OS=Rattus norvegicus GN=Tubb4b PE=3 SV=1
41.34	2	846.44	1690.86	-1.00	75	ALTVPELTQQMFDAK		Q4QRB4	Tubulin beta-3 chain OS=Rattus norvegicus GN=Tubb3 PE=1 SV=1
39.08	2	847.00	1691.98	-0.55	46	GLLSAVALAPQTVPVEK		M0R9L0	Protein Naca OS=Rattus norvegicus GN=Naca PE=4 SV=1
34.50	2	848.42	1694.83	-1.14	29	VDIDVPDVNIEGPDAK		Q38PG0	AHNAK 1 (Fragment) OS=Rattus norvegicus PE=2 SV=1
16.93	2	848.44	1694.86	-1.33	89	GAEAANVTGPGGVPVQGSK		F1LPL7	Protein LOC100912427 OS=Rattus norvegicus GN=LOC100912427 PE=4 SV=1

20.39	2	849.45	1696.88	-1.24	72	DGRGAAQNIIPASTGAAK		M0R590	Protein LOC685186 OS=Rattus norvegicus GN=LOC685186 PE=3 SV=1
34.25	2	850.88	1699.75	-1.21	52	FNADEFEDMVAEKR		Q6PDV7	60S ribosomal protein L10 OS=Rattus norvegicus GN=Rpl10 PE=1 SV=3
16.22	2	851.44	1700.86	-1.44	93	VAVVQYSGQGQQQPGR		D3ZUL3	Protein Col6a1 OS=Rattus norvegicus GN=Col6a1 PE=4 SV=1
41.25	3	567.97	1700.89	-2.10	30	AVFVDLEPTVIDEVR		Q6P9V9	Tubulin alpha-1B chain OS=Rattus norvegicus GN=Tuba1b PE=1 SV=1
41.25	3	567.97	1700.89	-2.10	30	AVFVDLEPTVIDEVR		M0R5B4	Uncharacterized protein OS=Rattus norvegicus PE=4 SV=1
41.17	2	851.46	1700.90	-0.46	81	AVFVDLEPTVIDEVR		Q6P9V9	Tubulin alpha-1B chain OS=Rattus norvegicus GN=Tuba1b PE=1 SV=1
41.17	2	851.46	1700.90	-0.46	81	AVFVDLEPTVIDEVR		M0R5B4	Uncharacterized protein OS=Rattus norvegicus PE=4 SV=1
45.00	2	851.95	1701.89	-1.06	29	AAGVNVEPFWPGLFAK		P19944	60S acidic ribosomal protein P1 OS=Rattus norvegicus GN=Rplp1 PE=3 SV=1
34.58	2	854.43	1706.85	-2.08	32	ALTVPELTQQMFDAK	[11] Oxidation (M)	B4F7C2	Protein Tubb4a OS=Rattus norvegicus GN=Tubb4a PE=2 SV=1
34.58	2	854.43	1706.85	-2.08	32	ALTVPELTQQMFDAK	[11] Oxidation (M)	Q4QQV0	Protein Tubb6 OS=Rattus norvegicus GN=Tubb6 PE=2 SV=1
34.58	2	854.43	1706.85	-2.08	32	ALTVPELTQQMFDAK	[11] Oxidation (M)	G3V7C6	RCG45400 OS=Rattus norvegicus GN=Tubb4b PE=3 SV=1
34.58	2	854.43	1706.85	-2.08	32	ALTVPELTQQMFDAK	[11] Oxidation (M)	Q4QRB4	Tubulin beta-3 chain OS=Rattus norvegicus GN=Tubb3 PE=1 SV=1
30.11	2	854.92	1707.82	-1.33	48	LLDPEDVDVPQPDEK		P30427	Plectin OS=Rattus norvegicus GN=Plec PE=1 SV=2
23.85	2	854.92	1707.82	-2.21	46	AFDELQKQMEGLGAR	[9] Oxidation (M)	D3ZH41	Cytoskeleton-associated protein 4 (Predicted) OS=Rattus norvegicus GN=Ckap4 PE=4 SV=2
46.31	2	855.89	1709.76	-0.02	35	NNSNDIVNAIMELTM	[11] Oxidation (M)|[15] Oxidation (M)	M0R9L0	Protein Naca OS=Rattus norvegicus GN=Naca PE=4 SV=1
37.89	2	857.96	1713.90	-1.86	35	SSGPTSLFAVTVAPPGAR		Q6IMY8	Heterogeneous nuclear ribonucleoprotein U OS=Rattus norvegicus GN=Hnrnpu PE=2 SV=1

28.97	2	858.44	1714.86	-0.17	43	NVQVYNPTPNSLDVR		F1LQC3	Collagen alpha-1(XII) chain (Fragment) OS=Rattus norvegicus GN=Col12a1 PE=4 SV=2
45.60	3	572.64	1714.90	-3.05	52	GIGTDEATIIDIITQR		Q6IMZ3	Annexin OS=Rattus norvegicus GN=Anxa6 PE=2 SV=1
45.60	2	858.46	1714.91	-0.69	71	GIGTDEATIIDIITQR		Q6IMZ3	Annexin OS=Rattus norvegicus GN=Anxa6 PE=2 SV=1
21.12	3	572.97	1715.89	-2.44	32	VLGDVIEVHGKHEER		P23928	Alpha-crystallin B chain OS=Rattus norvegicus GN=Cryab PE=1 SV=1
21.12	2	858.95	1715.89	-1.29	66	VLGDVIEVHGKHEER		P23928	Alpha-crystallin B chain OS=Rattus norvegicus GN=Cryab PE=1 SV=1
17.47	2	859.45	1716.88	1.21	47	RSQPVSAIATTAMGSPK	[13] Oxidation (M)	M0RA80	Protein Tnc OS=Rattus norvegicus GN=Tnc PE=4 SV=1
33.73	2	859.96	1717.90	-0.62	65	IIDVVYNASNNELVR		B2RYR8	40S ribosomal protein S8 OS=Rattus norvegicus GN=Rps8 PE=2 SV=1
26.45	3	574.95	1721.84	-2.83	40	VFDKEGNGTVMGAEIR		B2GV99	Myl6 protein OS=Rattus norvegicus GN=Myl6 PE=2 SV=1
26.45	3	574.95	1721.84	-2.83	40	VFDKEGNGTVMGAELR		P02600	Myosin light chain 1/3. skeletal muscle isoform OS=Rattus norvegicus GN=Myl1 PE=1 SV=2
26.45	3	574.95	1721.84	-2.83	40	VFDKEGNGTVMGAELR		P16409	Myosin light chain 3 OS=Rattus norvegicus GN=Myl3 PE=2 SV=2
26.45	2	861.93	1721.84	-0.39	92	VFDKEGNGTVMGAEIR		B2GV99	Myl6 protein OS=Rattus norvegicus GN=Myl6 PE=2 SV=1
26.45	2	861.93	1721.84	-0.39	92	VFDKEGNGTVMGAELR		P02600	Myosin light chain 1/3. skeletal muscle isoform OS=Rattus norvegicus GN=Myl1 PE=1 SV=2
26.45	2	861.93	1721.84	-0.39	92	VFDKEGNGTVMGAELR		P16409	Myosin light chain 3 OS=Rattus norvegicus GN=Myl3 PE=2 SV=2
21.84	2	861.94	1721.87	-0.73	71	GDNQSPIELHTKDIR		P14141	Carbonic anhydrase 3 OS=Rattus norvegicus GN=Ca3 PE=1 SV=3
25.10	3	575.95	1724.83	-2.38	67	SPGVMDNPLVMHQLR	[5] Oxidation (M)|[11] Oxidation (M)	P02564	Myosin-7 OS=Rattus norvegicus GN=Myh7 PE=2 SV=2
25.10	3	575.95	1724.83	-2.38	67	SPGVMDNPLVMHQLR	[5] Oxidation (M)|[11] Oxidation (M)	G3V8B0	Myosin-7 OS=Rattus norvegicus GN=Myh7 PE=4 SV=1

25.10	2	863.42	1724.83	-0.34	70	SPGVMDNPLVMH QLR	[5] Oxidation (M)|[11] Oxidation (M)	P02564	Myosin-7 OS=Rattus norvegicus GN=Myh7 PE=2 SV=2
25.10	2	863.42	1724.83	-0.34	70	SPGVMDNPLVMH QLR	[5] Oxidation (M)|[11] Oxidation (M)	G3V8B0	Myosin-7 OS=Rattus norvegicus GN=Myh7 PE=4 SV=1
43.66	2	863.98	1725.94	-0.84	71	QLLQANPILEAFGNAK		E9PTU4	Myosin-11 OS=Rattus norvegicus GN=Myh11 PE=4 SV=2
30.45	3	576.63	1726.88	-2.03	25	IQLVEEELDRAQER		P04692	Tropomyosin alpha-1 chain OS=Rattus norvegicus GN=Tpm1 PE=1 SV=3
30.45	3	576.63	1726.88	-2.03	25	IQLVEEELDRAQER		Q5FVG5	Similar to tropomyosin 1. embryonic fibroblast-rat. isoform CRA_c OS=Rattus norvegicus GN=Tpm2 PE=2 SV=1
30.45	3	576.63	1726.88	-2.03	25	IQLVEEELDRAQER		Q6AZ25	Tropomyosin 1. alpha OS=Rattus norvegicus GN=Tpm1 PE=2 SV=1
30.45	3	576.63	1726.88	-2.03	25	IQLVEEELDRAQER		Q63610	Tropomyosin alpha-3 chain OS=Rattus norvegicus GN=Tpm3 PE=1 SV=2
30.45	3	576.63	1726.88	-2.03	25	IQLVEEELDRAQER		P09495	Tropomyosin alpha-4 chain OS=Rattus norvegicus GN=Tpm4 PE=1 SV=3
30.45	2	864.45	1726.88	-0.98	33	IQLVEEELDRAQER		P04692	Tropomyosin alpha-1 chain OS=Rattus norvegicus GN=Tpm1 PE=1 SV=3
30.45	2	864.45	1726.88	-0.98	33	IQLVEEELDRAQER		Q5FVG5	Similar to tropomyosin 1. embryonic fibroblast-rat. isoform CRA_c OS=Rattus norvegicus GN=Tpm2 PE=2 SV=1
30.45	2	864.45	1726.88	-0.98	33	IQLVEEELDRAQER		Q6AZ25	Tropomyosin 1. alpha OS=Rattus norvegicus GN=Tpm1 PE=2 SV=1
30.45	2	864.45	1726.88	-0.98	33	IQLVEEELDRAQER		Q63610	Tropomyosin alpha-3 chain OS=Rattus norvegicus GN=Tpm3 PE=1 SV=2
30.45	2	864.45	1726.88	-0.98	33	IQLVEEELDRAQER		P09495	Tropomyosin alpha-4 chain OS=Rattus norvegicus GN=Tpm4 PE=1 SV=3
17.55	2	865.44	1728.87	-1.95	62	VSDLTQAANKNNDALR		Q6P725	Desmin OS=Rattus norvegicus GN=Des PE=2 SV=1
28.41	2	868.44	1734.86	-0.57	53	TYFSHIDVSPGSAQVK		B1H216	Hemoglobin alpha. adult chain 2 OS=Rattus norvegicus GN=Hba1 PE=2 SV=1

19.52	3	580.28	1737.83	-1.73	56	VFDKEGNGTVMGA EIR	[11] Oxidation (M)	B2GV99	Myl6 protein OS=Rattus norvegicus GN=Myl6 PE=2 SV=1
19.52	3	580.28	1737.83	-1.73	56	VFDKEGNGTVMGA ELR	[11] Oxidation (M)	P02600	Myosin light chain 1/3. skeletal muscle isoform OS=Rattus norvegicus GN=Myl1 PE=1 SV=2
19.52	3	580.28	1737.83	-1.73	56	VFDKEGNGTVMGA ELR	[11] Oxidation (M)	P16409	Myosin light chain 3 OS=Rattus norvegicus GN=Myl3 PE=2 SV=2
19.60	2	869.92	1737.83	-1.21	98	VFDKEGNGTVMGA EIR	[11] Oxidation (M)	B2GV99	Myl6 protein OS=Rattus norvegicus GN=Myl6 PE=2 SV=1
19.60	2	869.92	1737.83	-1.21	98	VFDKEGNGTVMGA ELR	[11] Oxidation (M)	P02600	Myosin light chain 1/3. skeletal muscle isoform OS=Rattus norvegicus GN=Myl1 PE=1 SV=2
19.60	2	869.92	1737.83	-1.21	98	VFDKEGNGTVMGA ELR	[11] Oxidation (M)	P16409	Myosin light chain 3 OS=Rattus norvegicus GN=Myl3 PE=2 SV=2
26.71	2	870.92	1739.83	0.02	37	EGDPATINAATEIDAPR		M0RA80	Protein Tnc OS=Rattus norvegicus GN=Tnc PE=4 SV=1
36.32	3	580.98	1739.92	-1.78	45	ILNPAAIPEGQFIDSR		G3V885	Myosin-6 OS=Rattus norvegicus GN=Myh6 PE=4 SV=1
36.32	3	580.98	1739.92	-1.78	45	ILNPAAIPEGQFIDSR		P02564	Myosin-7 OS=Rattus norvegicus GN=Myh7 PE=2 SV=2
36.32	3	580.98	1739.92	-1.78	45	ILNPAAIPEGQFIDSR		G3V8B0	Myosin-7 OS=Rattus norvegicus GN=Myh7 PE=4 SV=1
36.32	2	870.97	1739.92	0.09	81	ILNPAAIPEGQFIDSR		G3V885	Myosin-6 OS=Rattus norvegicus GN=Myh6 PE=4 SV=1
36.32	2	870.97	1739.92	0.09	81	ILNPAAIPEGQFIDSR		P02564	Myosin-7 OS=Rattus norvegicus GN=Myh7 PE=2 SV=2
36.32	2	870.97	1739.92	0.09	81	ILNPAAIPEGQFIDSR		G3V8B0	Myosin-7 OS=Rattus norvegicus GN=Myh7 PE=4 SV=1
33.18	2	872.41	1742.81	-0.64	94	NKDTGTYEDFVEGLR		P16409	Myosin light chain 3 OS=Rattus norvegicus GN=Myl3 PE=2 SV=2
47.10	2	872.41	1742.81	-0.70	120	AMGIMNSFVNDIFER		G3V8B3	Histone H2B OS=Rattus norvegicus GN=LOC684797 PE=3 SV=1
28.56	3	583.70	1748.06	-2.12	36	AVVGVVAGGGRIDKPILK		P62919	60S ribosomal protein L8 OS=Rattus norvegicus GN=Rpl8 PE=2 SV=2
28.56	2	875.04	1748.07	-0.74	40	AVVGVVAGGGRIDKPILK		P62919	60S ribosomal protein L8 OS=Rattus norvegicus GN=Rpl8 PE=2 SV=2
32.90	2	875.49	1748.97	-0.93	31	TQVELLEPPTPELKR		A2RRU1	Glycogen [starch] synthase. muscle OS=Rattus norvegicus GN=Gys1 PE=2 SV=1
21.12	2	876.40	1750.78	-0.68	42	SLHQAIEGDTSGDF MK	[15] Oxidation (M)	Q6IMZ3	Annexin OS=Rattus norvegicus GN=Anxa6 PE=2 SV=1

24.01	2	876.93	1751.85	-0.47	83	NSNLVGAAHEELQQSR		G3V8L3	Lamin A. isoform CRA_b OS=Rattus norvegicus GN=Lmna PE=3 SV=1
29.94	2	878.49	1754.96	-1.38	73	TVGVEPAADGKGVVVVMK		Q642E2	Protein LOC100362069 OS=Rattus norvegicus GN=Rpl28 PE=2 SV=1
38.15	3	586.32	1755.95	-1.98	47	IHFPLATYAPVISAEK		Q6P9V9	Tubulin alpha-1B chain OS=Rattus norvegicus GN=Tuba1b PE=1 SV=1
38.15	3	586.32	1755.95	-1.98	47	IHFPLATYAPVISAEK		Q5XIF6	Tubulin alpha-4A chain OS=Rattus norvegicus GN=Tuba4a PE=2 SV=1
38.15	2	878.99	1755.96	-0.04	68	IHFPLATYAPVISAEK		Q6P9V9	Tubulin alpha-1B chain OS=Rattus norvegicus GN=Tuba1b PE=1 SV=1
38.15	2	878.99	1755.96	-0.04	68	IHFPLATYAPVISAEK		Q5XIF6	Tubulin alpha-4A chain OS=Rattus norvegicus GN=Tuba4a PE=2 SV=1
36.85	2	880.41	1758.80	0.39	32	SSASAPDVDDPEAFPALA		Q6AXS5	Plasminogen activator inhibitor 1 RNA-binding protein OS=Rattus norvegicus GN=Serbp1 PE=1 SV=2
45.00	2	880.41	1758.81	-0.90	111	AMGIMNSFVNDIF ER	[2] Oxidation (M)	G3V8B3	Histone H2B OS=Rattus norvegicus GN=LOC684797 PE=3 SV=1
42.16	2	880.41	1758.81	0.43	77	AMGIMNSFVNDIF ER	[5] Oxidation (M)	G3V8B3	Histone H2B OS=Rattus norvegicus GN=LOC684797 PE=3 SV=1
36.01	2	880.90	1759.79	-0.08	51	AISDELDHALNDMT SI	[13] Oxidation (M)	Q9QX08	Nonmuscle tropomyosin 5 (Fragment) OS=Rattus norvegicus GN=Tpm3 PE=2 SV=1
45.73	3	587.66	1759.97	-3.72	25	IRSFPDFPIPGVLFR		P36972	Adenine phosphoribosyltransferase OS=Rattus norvegicus GN=Aprt PE=1 SV=1
22.82	3	588.63	1762.87	-2.13	42	AAVEQLTEEQKNEFK		Q4PP99	Cardiac troponin C OS=Rattus norvegicus GN=Tnnc1 PE=2 SV=1
22.82	2	882.44	1762.87	-1.09	78	AAVEQLTEEQKNEFK		Q4PP99	Cardiac troponin C OS=Rattus norvegicus GN=Tnnc1 PE=2 SV=1
33.15	2	883.00	1763.98	-0.70	47	KGVNLPGAAVDLPAVSEK		P11980	Pyruvate kinase PKM OS=Rattus norvegicus GN=Pkm PE=1 SV=3
13.48	2	883.37	1764.73	-1.02	68	TAENATSGETLEENGAGD		Q6AYD3	Proliferation-associated 2G4 OS=Rattus norvegicus GN=Pa2g4 PE=2 SV=1
49.26	2	883.97	1765.93	-0.91	29	EKLGPVPFFSLLQYE		P36972	Adenine phosphoribosyltransferase OS=Rattus norvegicus GN=Aprt PE=1 SV=1
26.45	2	884.92	1767.83	-0.24	59	DGEVVSEATQQQHEVL		Q6P725	Desmin OS=Rattus norvegicus GN=Des PE=2 SV=1

31.88	3	590.29	1767.86	-3.24	63	MSQRFPNAEFAEITK		P02770	Serum albumin OS=Rattus norvegicus GN=Alb PE=1 SV=2
31.97	2	884.94	1767.86	-0.63	71	MSQRFPNAEFAEITK		P02770	Serum albumin OS=Rattus norvegicus GN=Alb PE=1 SV=2
17.17	2	885.45	1768.89	-1.51	47	AALAHSEEIATSQAAATK		P30427	Plectin OS=Rattus norvegicus GN=Plec PE=1 SV=2
39.53	2	885.46	1768.90	-0.44	87	ALEGSALYTGSALDFVR		D4A111	Protein Col6a3 OS=Rattus norvegicus GN=Col6a3 PE=4 SV=2
37.98	3	590.65	1768.93	-2.46	37	GPVREGDVLTLLESER		P62859	40S ribosomal protein S28 OS=Rattus norvegicus GN=Rps28 PE=1 SV=1
22.49	2	887.45	1772.89	0.39	64	SVSEAGVGESSAVTQPIR		D4A228	Protein Myom3 OS=Rattus norvegicus GN=Myom3 PE=4 SV=2
40.14	2	888.41	1774.80	-0.20	91	AMGIMNSFVNDIF ER	[2] Oxidation (M)|[5] Oxidation (M)	G3V8B3	Histone H2B OS=Rattus norvegicus GN=LOC684797 PE=3 SV=1
33.18	2	890.40	1778.79	-0.23	27	LISWYDNEYGYSNR		M0R590	Protein LOC685186 OS=Rattus norvegicus GN=LOC685186 PE=3 SV=1
33.18	2	890.40	1778.79	-0.23	27	LISWYDNEYGYSNR		M0R451	Uncharacterized protein OS=Rattus norvegicus PE=3 SV=1
33.18	2	890.40	1778.79	-0.23	27	LISWYDNEYGYSNR		D3ZIY0	Uncharacterized protein OS=Rattus norvegicus PE=3 SV=1
33.18	2	890.40	1778.79	-0.23	27	LISWYDNEYGYSNR		D3Z8U8	Uncharacterized protein OS=Rattus norvegicus PE=4 SV=1
19.91	2	890.90	1779.79	-1.88	63	TDYNASVSVPDSSGPER		M0R9K1	Protein Gm7964 OS=Rattus norvegicus GN=Gm7964 PE=4 SV=1
27.12	2	890.92	1779.83	-0.53	53	VDATEESDLAQQYGVR		P04785	Protein disulfide-isomerase OS=Rattus norvegicus GN=P4hb PE=1 SV=2
19.67	2	891.93	1781.85	-1.22	117	GAEAANVTGPDGVPVEGSR		Q62764	Y-box-binding protein 3 OS=Rattus norvegicus GN=Ybx3 PE=2 SV=1
19.67	2	891.93	1781.85	-1.22	117	GAEAANVTGPDGVPVEGSR		D4A0L4	Y-box-binding protein 3 OS=Rattus norvegicus GN=Ybx3 PE=4 SV=2
13.78	3	594.99	1781.94	-2.13	79	AAPAPAAAPAAAPEPERPK		P16409	Myosin light chain 3 OS=Rattus norvegicus GN=Myl3 PE=2 SV=2

13.78	2	891.98	1781.94	-1.12	101	AAPAPAAAPAAAPEPERPK		P16409	Myosin light chain 3 OS=Rattus norvegicus GN=Myl3 PE=2 SV=2
44.91	2	892.45	1782.88	-0.33	39	LTGKDVNFEFPEFQL		B5DEL9	RCG62292. isoform CRA_a OS=Rattus norvegicus GN=Rps7 PE=2 SV=1
17.94	3	595.62	1783.83	-3.25	54	IKDPDAAKPEDWDER		P18418	Calreticulin OS=Rattus norvegicus GN=Calr PE=1 SV=1
17.94	2	892.92	1783.83	-1.67	53	IKDPDAAKPEDWDER		P18418	Calreticulin OS=Rattus norvegicus GN=Calr PE=1 SV=1
31.12	3	595.62	1783.85	-1.87	35	MSQRFPNAEFAEIT K	[1] Oxidation (M)	P02770	Serum albumin OS=Rattus norvegicus GN=Alb PE=1 SV=2
31.03	2	892.94	1783.86	-0.26	57	MSQRFPNAEFAEIT K	[1] Oxidation (M)	P02770	Serum albumin OS=Rattus norvegicus GN=Alb PE=1 SV=2
37.12	3	595.99	1784.95	-2.12	34	LGSSEVEQVQLVVDGVK		P00564	Creatine kinase M-type OS=Rattus norvegicus GN=Ckm PE=1 SV=2
37.12	2	893.48	1784.95	-0.31	96	LGSSEVEQVQLVVDGVK		P00564	Creatine kinase M-type OS=Rattus norvegicus GN=Ckm PE=1 SV=2
35.27	2	895.95	1789.89	0.81	120	SYELPDGQVITIGNER		P60711	Actin. cytoplasmic 1 OS=Rattus norvegicus GN=Actb PE=1 SV=1
35.27	2	895.95	1789.89	0.81	120	SYELPDGQVITIGNER		P68136	Actin. alpha skeletal muscle OS=Rattus norvegicus GN=Acta1 PE=1 SV=1
35.27	2	895.95	1789.89	0.81	120	SYELPDGQVITIGNER		P68035	Actin. alpha cardiac muscle 1 OS=Rattus norvegicus GN=Actc1 PE=2 SV=1
35.27	2	895.95	1789.89	0.81	120	SYELPDGQVITIGNER		D3ZRN3	Protein Actbl2 OS=Rattus norvegicus GN=Actbl2 PE=3 SV=1
31.72	2	898.41	1794.81	-0.72	30	SVGDGETVEFDVVEGEK		Q62764	Y-box-binding protein 3 OS=Rattus norvegicus GN=Ybx3 PE=2 SV=1
31.72	2	898.41	1794.81	-0.72	30	SVGDGETVEFDVVEGEK		F1LPL7	Protein LOC100912427 OS=Rattus norvegicus GN=LOC100912427 PE=4 SV=1
31.72	2	898.41	1794.81	-0.72	30	SVGDGETVEFDVVEGEK		D4A0L4	Y-box-binding protein 3 OS=Rattus norvegicus GN=Ybx3 PE=4 SV=2
33.81	2	898.44	1794.86	-2.41	28	GADYLVTEVENGGSLGSK		P11980	Pyruvate kinase PKM OS=Rattus norvegicus GN=Pkm PE=1 SV=3
35.09	3	599.98	1796.93	-1.90	38	VSLDVNHFAPEELTVK		G3V913	Heat shock 27kDa protein 1 OS=Rattus norvegicus GN=Hspb1 PE=3 SV=1

35.09	2	899.47	1796.93	-0.31	44	VSLDVNHFAPEELTVK		G3V913	Heat shock 27kDa protein 1 OS=Rattus norvegicus GN=Hspb1 PE=3 SV=1
20.48	2	900.98	1799.94	-1.04	70	VAQPSITDNKDGTVTVR		C0JPT7	Filamin alpha OS=Rattus norvegicus GN=Flna PE=2 SV=1
44.00	2	900.99	1799.97	-0.31	46	LAPDYDALDVANKIGII		F1LT35	Protein RGD1564606 (Fragment) OS=Rattus norvegicus GN=RGD1564606 PE=3 SV=1
38.76	2	902.98	1803.94	0.11	97	AAVPSGASTGIYEALELR		P15429	Beta-enolase OS=Rattus norvegicus GN=Eno3 PE=1 SV=3
38.76	2	902.98	1803.94	0.11	97	AAVPSGASTGIYEALELR		P07323	Gamma-enolase OS=Rattus norvegicus GN=Eno2 PE=1 SV=2
38.76	2	902.98	1803.94	0.11	97	AAVPSGASTGIYEALELR		M0RAU4	Uncharacterized protein (Fragment) OS=Rattus norvegicus PE=3 SV=1
38.76	2	902.98	1803.94	0.11	97	AAVPSGASTGIYEALELR		M0R5J4	Uncharacterized protein OS=Rattus norvegicus PE=3 SV=1
28.56	2	905.94	1809.86	-0.76	28	AFTGREFDELSPSAQR		E9PSX6	Sarcoplasmic/endoplasmic reticulum calcium ATPase 2 OS=Rattus norvegicus GN=Atp2a2 PE=3 SV=2
27.12	2	906.46	1810.91	-1.49	31	TLQEQLENGPNTQLAR		F1M853	Protein Rrbp1 OS=Rattus norvegicus GN=Rrbp1 PE=4 SV=2
28.16	2	908.42	1814.83	-0.61	75	ISSSNPYSTVTMDEL R	[12] Oxidation (M)	D3ZCV0	Protein Actn2 OS=Rattus norvegicus GN=Actn2 PE=4 SV=1
40.83	2	908.48	1814.94	-0.56	27	LAQDPFPLYPGEVLEK		Q62667	Major vault protein OS=Rattus norvegicus GN=Mvp PE=1 SV=4
13.93	2	909.97	1817.92	-2.50	41	VATVPQHATSGPGPADVSK		C0JPT7	Filamin alpha OS=Rattus norvegicus GN=Flna PE=2 SV=1
19.67	2	911.43	1820.85	-1.52	29	AATPPNPSSTADSAGNPVAP		Q5U2U8	Bcl2-associated athanogene 3 OS=Rattus norvegicus GN=Bag3 PE=2 SV=1
21.12	2	911.45	1820.88	-1.21	69	NQVAMNPTNTVF DAKR	[5] Oxidation (M)	P63018	Heat shock cognate 71 kDa protein OS=Rattus norvegicus GN=Hspa8 PE=1 SV=1
21.12	2	911.45	1820.88	-1.21	69	NQVAMNPTNTVF DAKR	[5] Oxidation (M)	D3ZH98	Uncharacterized protein OS=Rattus norvegicus PE=3 SV=1
33.73	3	608.98	1823.92	-2.25	30	FSNEEIAMATVTAL RR	[8] Oxidation (M)	P05065	Fructose-bisphosphate aldolase A OS=Rattus norvegicus GN=Aldoa PE=1 SV=2
28.11	2	913.43	1824.84	-0.40	107	TEAAEIVEGEDSAYSVR		D3ZHA0	Protein Flnc OS=Rattus norvegicus GN=Flnc PE=4 SV=1

35.27	2	914.48	1826.94	4.97	36	HAQVISSSGIMSLGIGDR		D4A111	Protein Col6a3 OS=Rattus norvegicus GN=Col6a3 PE=4 SV=2
40.14	2	915.94	1829.86	-0.52	60	ELTEEKETAFEFLSSA		O88989	Malate dehydrogenase. cytoplasmic OS=Rattus norvegicus GN=Mdh1 PE=1 SV=3
37.46	2	916.99	1831.97	-0.47	71	AVTQSAEITIPVTFEAR		G3V913	Heat shock 27kDa protein 1 OS=Rattus norvegicus GN=Hspb1 PE=3 SV=1
44.35	2	918.03	1834.05	-2.11	57	KEGGLGPLNIPLLADVTK		P35704	Peroxiredoxin-2 OS=Rattus norvegicus GN=Prdx2 PE=1 SV=3
42.34	2	918.34	1834.67	-0.50	36	EESEESDEDMGFGLFD		D4A4D5	Protein LOC100362751 OS=Rattus norvegicus GN=LOC498555 PE=3 SV=1
42.34	2	918.34	1834.67	-0.50	36	EESEESDEDMGFGLFD		P19945	60S acidic ribosomal protein P0 OS=Rattus norvegicus GN=Rplp0 PE=1 SV=2
42.34	2	918.34	1834.67	-0.50	29	EESEESEDDMGFGLFD		P19944	60S acidic ribosomal protein P1 OS=Rattus norvegicus GN=Rplp1 PE=3 SV=1
24.01	2	918.47	1834.92	-0.14	56	KKPLNIDYMGEDQLR	[9] Oxidation (M)	M0R799	Troponin T. slow skeletal muscle OS=Rattus norvegicus GN=Tnnt1 PE=4 SV=1
16.93	2	918.90	1835.79	-1.28	69	DGQVINETSQHHDDLE		P31000	Vimentin OS=Rattus norvegicus GN=Vim PE=1 SV=2
31.12	2	919.97	1837.92	-0.59	108	DLEEATLQHEATAAALR		G3V885	Myosin-6 OS=Rattus norvegicus GN=Myh6 PE=4 SV=1
31.12	2	919.97	1837.92	-0.59	108	DLEEATLQHEATAAALR		F1LMU0	Myosin-4 OS=Rattus norvegicus GN=Myh4 PE=2 SV=1
31.12	2	919.97	1837.92	-0.59	108	DLEEATLQHEATAAALR		P02564	Myosin-7 OS=Rattus norvegicus GN=Myh7 PE=2 SV=2
31.12	2	919.97	1837.92	-0.59	108	DLEEATLQHEATAAALR		G3V8B0	Myosin-7 OS=Rattus norvegicus GN=Myh7 PE=4 SV=1
29.78	2	919.98	1837.95	-0.99	52	ETNLESLPLVDTHSKR		P31000	Vimentin OS=Rattus norvegicus GN=Vim PE=1 SV=2
46.59	3	614.35	1840.03	-2.04	41	VEPAVSSIVNSIQVLASK		F1M853	Protein Rrbp1 OS=Rattus norvegicus GN=Rrbp1 PE=4 SV=2
46.59	2	921.02	1840.03	-0.03	94	VEPAVSSIVNSIQVLASK		F1M853	Protein Rrbp1 OS=Rattus norvegicus GN=Rrbp1 PE=4 SV=2
24.51	2	924.50	1846.98	-1.00	52	AHLGTALKANPFGGASHAK		P62268	40S ribosomal protein S23 OS=Rattus norvegicus GN=Rps23 PE=1 SV=3
45.29	2	924.95	1847.89	-0.51	74	GLSDGEWQMVLNIWGK	[9] Oxidation (M)	Q9QZ76	Myoglobin OS=Rattus norvegicus GN=Mb PE=1 SV=3
44.84	2	925.58	1849.14	-0.65	32	NIPGITLLNVSKLNILK		Q6P3V9	60S ribosomal protein L4 OS=Rattus norvegicus GN=Rpl4 PE=2 SV=1

33.18	2	927.45	1852.88	-0.38	75	YLEENQETFVPLESR		D3ZWJ2	Nestin OS=Rattus norvegicus GN=Nes PE=3 SV=2
8.65	2	928.91	1855.81	-0.67	94	AGEAPTENPAPATEQSSAE		Q62764	Y-box-binding protein 3 OS=Rattus norvegicus GN=Ybx3 PE=2 SV=1
8.65	2	928.91	1855.81	-0.67	94	AGEAPTENPAPATEQSSAE		D4A0L4	Y-box-binding protein 3 OS=Rattus norvegicus GN=Ybx3 PE=4 SV=2
26.54	3	620.30	1857.87	-2.01	35	MLDAEDIVNTPKPDER	[1] Oxidation (M)	D3ZCV0	Protein Actn2 OS=Rattus norvegicus GN=Actn2 PE=4 SV=1
35.34	3	621.35	1861.03	-2.36	32	APIRPDIVNFVHTNLR		Q6P3V9	60S ribosomal protein L4 OS=Rattus norvegicus GN=Rpl4 PE=2 SV=1
30.36	2	934.97	1867.93	-0.62	94	DLEEATLQHEATAATLR		F1LRV9	Protein Myh1 OS=Rattus norvegicus GN=Myh1 PE=2 SV=2
30.36	2	934.97	1867.93	-0.62	94	DLEEATLQHEATAATLR		F1M789	Protein Myh13 OS=Rattus norvegicus GN=Myh13 PE=4 SV=2
30.36	2	934.97	1867.93	-0.62	94	DLEEATLQHEATAATLR		G3V6E1	Uncharacterized protein OS=Rattus norvegicus GN=Myh2 PE=4 SV=2
34.16	2	935.02	1868.02	3.08	45	ILNPAAIPEGQFIDSRK		G3V885	Myosin-6 OS=Rattus norvegicus GN=Myh6 PE=4 SV=1
34.16	2	935.02	1868.02	3.08	45	ILNPAAIPEGQFIDSRK		P02564	Myosin-7 OS=Rattus norvegicus GN=Myh7 PE=2 SV=2
34.16	2	935.02	1868.02	3.08	45	ILNPAAIPEGQFIDSRK		G3V8B0	Myosin-7 OS=Rattus norvegicus GN=Myh7 PE=4 SV=1
18.95	3	624.67	1870.98	-2.33	45	TARPNITDNKDGTITVR		D3ZHA0	Protein Flnc OS=Rattus norvegicus GN=Flnc PE=4 SV=1
18.95	2	936.50	1870.98	-0.73	56	TARPNITDNKDGTITVR		D3ZHA0	Protein Flnc OS=Rattus norvegicus GN=Flnc PE=4 SV=1
50.05	2	938.45	1874.89	2.48	38	FGVEQDVDMVFASFIR	[9] Oxidation (M)	P11980	Pyruvate kinase PKM OS=Rattus norvegicus GN=Pkm PE=1 SV=3
42.25	3	627.67	1879.98	-1.43	37	RVDFETFLPMLQAVAK	[10] Oxidation (M)	D3ZHA7	Protein Myl6b OS=Rattus norvegicus GN=Myl6b PE=4 SV=1
42.25	2	941.00	1879.99	-0.08	59	RVDFETFLPMLQAVAK	[10] Oxidation (M)	D3ZHA7	Protein Myl6b OS=Rattus norvegicus GN=Myl6b PE=4 SV=1
47.23	2	944.50	1886.99	-3.08	50	VLDFEHFLPMLQTVAK		B2GV99	Myl6 protein OS=Rattus norvegicus GN=Myl6 PE=2 SV=1
11.62	3	630.70	1889.08	-3.19	32	KPAAAAPAPAPAPAPAPAKPK		P02600	Myosin light chain 1/3. skeletal muscle isoform OS=Rattus norvegicus GN=Myl1 PE=1 SV=2
29.30	2	949.53	1897.05	-0.73	49	IVAPGKGILAADESTGSIAK		P05065	Fructose-bisphosphate aldolase A OS=Rattus norvegicus GN=Aldoa PE=1 SV=2

29.30	2	949.53	1897.05	-0.73	49	IVAPGKGILAADESTGSIAK		Q6AY07	Fructose-bisphosphate aldolase OS=Rattus norvegicus GN=Aldoart2 PE=2 SV=1
40.06	2	950.98	1899.95	-0.02	94	LAMQEFMILPVGA SSFK	[3] Oxidation (M)|[7] Oxidation (M)	P15429	Beta-enolase OS=Rattus norvegicus GN=Eno3 PE=1 SV=3
43.66	3	635.34	1902.99	-2.45	40	VLDFEHFLPMLQTV AK	[10] Oxidation (M)	B2GV99	Myl6 protein OS=Rattus norvegicus GN=Myl6 PE=2 SV=1
43.66	2	952.50	1902.99	-1.60	40	VLDFEHFLPMLQTV AK	[10] Oxidation (M)	B2GV99	Myl6 protein OS=Rattus norvegicus GN=Myl6 PE=2 SV=1
47.03	2	953.54	1905.07	-0.30	59	VTWAPPPSIELTNLLVR		F1LST1	Fibronectin OS=Rattus norvegicus GN=Fn1 PE=4 SV=2
14.94	2	955.90	1909.78	-1.14	130	SSGSPYGGGYGSGGGSGGYGSR		Q6URK4	Heterogeneous nuclear ribonucleoprotein A3 OS=Rattus norvegicus GN=Hnrnpa3 PE=1 SV=1
34.16	2	957.00	1911.99	-1.12	59	SSPVVIDASTAIDAPSNLR		F1LST1	Fibronectin OS=Rattus norvegicus GN=Fn1 PE=4 SV=2
35.84	3	640.37	1918.09	-1.76	43	VLDSGAPIKIPVGPETLGR		G3V6D3	ATP synthase subunit beta OS=Rattus norvegicus GN=Atp5b PE=3 SV=1
35.84	2	960.05	1918.09	-0.34	38	VLDSGAPIKIPVGPETLGR		G3V6D3	ATP synthase subunit beta OS=Rattus norvegicus GN=Atp5b PE=3 SV=1
40.23	2	964.98	1927.95	-0.35	96	LAMQEFMILPVGA SSFR	[3] Oxidation (M)|[7] Oxidation (M)	M0R964	Enolase (Fragment) OS=Rattus norvegicus PE=3 SV=1
40.23	2	964.98	1927.95	-0.35	96	LAMQEFMILPVGA SSFR	[3] Oxidation (M)|[7] Oxidation (M)	F1LTP6	Enolase OS=Rattus norvegicus PE=3 SV=2
40.23	2	964.98	1927.95	-0.35	96	LAMQEFMILPVGA SSFR	[3] Oxidation (M)|[7] Oxidation (M)	M0RAU4	Uncharacterized protein (Fragment) OS=Rattus norvegicus PE=3 SV=1
40.23	2	964.98	1927.95	-0.35	96	LAMQEFMILPVGA SSFR	[3] Oxidation (M)|[7] Oxidation (M)	M0R5J4	Uncharacterized protein OS=Rattus norvegicus PE=3 SV=1
45.51	3	644.39	1930.16	-2.81	39	VTIAQGGVLPNIQAVLLPK		M0RDM4	Histone H2A OS=Rattus norvegicus GN=LOC680322 PE=3 SV=1
45.51	2	966.09	1930.16	0.30	78	VTIAQGGVLPNIQAVLLPK		M0RDM4	Histone H2A OS=Rattus norvegicus GN=LOC680322 PE=3 SV=1

31.03	2	971.97	1941.92	-0.54	76	VWLDPNETNEIANANSR	P84100	60S ribosomal protein L19 OS=Rattus norvegicus GN=Rpl19 PE=1 SV=1
38.67	2	972.49	1942.96	-0.17	60	NEEDATELVTLAQAVNAR	Q5U300	Ubiquitin-like modifier-activating enzyme 1 OS=Rattus norvegicus GN=Uba1 PE=1 SV=1
39.80	2	975.38	1948.75	-0.40	45	KEESEESDDDMGFGLFD	P02401	60S acidic ribosomal protein P2 OS=Rattus norvegicus GN=Rplp2 PE=1 SV=2
32.31	3	652.03	1953.06	-0.98	42	VAPEEHPVLLTEAPLNPK	P60711	Actin. cytoplasmic 1 OS=Rattus norvegicus GN=Actb PE=1 SV=1
32.31	3	652.03	1953.06	-0.98	36	VAPDEHPILLTEAPLNPK	D3ZRN3	Protein Actbl2 OS=Rattus norvegicus GN=Actbl2 PE=3 SV=1
32.31	2	977.54	1953.06	0.67	71	VAPEEHPVLLTEAPLNPK	P60711	Actin. cytoplasmic 1 OS=Rattus norvegicus GN=Actb PE=1 SV=1
32.31	2	977.54	1953.06	0.67	26	VAPDEHPILLTEAPLNPK	D3ZRN3	Protein Actbl2 OS=Rattus norvegicus GN=Actbl2 PE=3 SV=1
31.37	2	978.53	1955.04	0.25	84	VAPEEHPTLLTEAPLNPK	P68035	Actin. alpha cardiac muscle 1 OS=Rattus norvegicus GN=Actc1 PE=2 SV=1
31.37	2	978.53	1955.04	0.25	84	VAPEEHPTLLTEAPLNPK	P68136	Actin. alpha skeletal muscle OS=Rattus norvegicus GN=Acta1 PE=1 SV=1
31.37	3	652.69	1955.04	0.37	48	VAPEEHPTLLTEAPLNPK	P68035	Actin. alpha cardiac muscle 1 OS=Rattus norvegicus GN=Actc1 PE=2 SV=1
31.37	3	652.69	1955.04	0.37	48	VAPEEHPTLLTEAPLNPK	P68136	Actin. alpha skeletal muscle OS=Rattus norvegicus GN=Acta1 PE=1 SV=1
32.57	2	978.53	1955.05	5.71	26	VAPEEHPTLLTEAPLNPK	P68035	Actin. alpha cardiac muscle 1 OS=Rattus norvegicus GN=Actc1 PE=2 SV=1
32.57	2	978.53	1955.05	5.71	26	VAPEEHPTLLTEAPLNPK	P68136	Actin. alpha skeletal muscle OS=Rattus norvegicus GN=Acta1 PE=1 SV=1
44.00	3	653.66	1957.97	-1.97	26	GHYTEGAELVDSVLDVVR	Q4QQV0	Protein Tubb6 OS=Rattus norvegicus GN=Tubb6 PE=2 SV=1
44.00	3	653.66	1957.97	-1.97	26	GHYTEGAELVDSVLDVVR	G3V7C6	RCG45400 OS=Rattus norvegicus GN=Tubb4b PE=3 SV=1
44.00	3	653.66	1957.97	-1.97	26	GHYTEGAELVDSVLDVVR	P85108	Tubulin beta-2A chain OS=Rattus norvegicus GN=Tubb2a PE=1 SV=1

44.00	3	653.66	1957.97	-1.97	26	GHYTEGAELVDSVLDVVR	Q4QRB4	Tubulin beta-3 chain OS=Rattus norvegicus GN=Tubb3 PE=1 SV=1
44.00	3	653.66	1957.97	-1.97	26	GHYTEGAELVDSVLDVVR	P69897	Tubulin beta-5 chain OS=Rattus norvegicus GN=Tubb5 PE=1 SV=1
44.00	2	979.99	1957.97	-0.83	66	GHYTEGAELVDSVLDVVR	Q4QQV0	Protein Tubb6 OS=Rattus norvegicus GN=Tubb6 PE=2 SV=1
44.00	2	979.99	1957.97	-0.83	66	GHYTEGAELVDSVLDVVR	G3V7C6	RCG45400 OS=Rattus norvegicus GN=Tubb4b PE=3 SV=1
44.00	2	979.99	1957.97	-0.83	66	GHYTEGAELVDSVLDVVR	P85108	Tubulin beta-2A chain OS=Rattus norvegicus GN=Tubb2a PE=1 SV=1
44.00	2	979.99	1957.97	-0.83	66	GHYTEGAELVDSVLDVVR	Q4QRB4	Tubulin beta-3 chain OS=Rattus norvegicus GN=Tubb3 PE=1 SV=1
44.00	2	979.99	1957.97	-0.83	66	GHYTEGAELVDSVLDVVR	P69897	Tubulin beta-5 chain OS=Rattus norvegicus GN=Tubb5 PE=1 SV=1
26.87	2	980.53	1959.04	-2.52	58	YTQKAPQVSTPTLVEAAR	P02770	Serum albumin OS=Rattus norvegicus GN=Alb PE=1 SV=2
26.87	3	654.02	1959.04	-1.22	51	YTQKAPQVSTPTLVEAAR	P02770	Serum albumin OS=Rattus norvegicus GN=Alb PE=1 SV=2
39.88	2	982.39	1962.76	-1.43	80	KEESEESEDDMGFGLFD	P19944	60S acidic ribosomal protein P1 OS=Rattus norvegicus GN=Rplp1 PE=3 SV=1
39.88	2	982.39	1962.76	-1.43	69	KEESEESDEDMGFGLFD	D4A4D5	Protein LOC100362751 OS=Rattus norvegicus GN=LOC498555 PE=3 SV=1
28.64	3	656.34	1966.01	-1.37	55	DLEEATLQHEATAAALRK	G3V885	Myosin-6 OS=Rattus norvegicus GN=Myh6 PE=4 SV=1
28.64	3	656.34	1966.01	-1.37	55	DLEEATLQHEATAAALRK	F1LMU0	Myosin-4 OS=Rattus norvegicus GN=Myh4 PE=2 SV=1
28.64	3	656.34	1966.01	-1.37	55	DLEEATLQHEATAAALRK	P02564	Myosin-7 OS=Rattus norvegicus GN=Myh7 PE=2 SV=2
28.64	3	656.34	1966.01	-1.37	55	DLEEATLQHEATAAALRK	G3V8B0	Myosin-7 OS=Rattus norvegicus GN=Myh7 PE=4 SV=1
28.64	2	984.01	1966.01	-0.32	34	DLEEATLQHEATAAALRK	G3V885	Myosin-6 OS=Rattus norvegicus GN=Myh6 PE=4 SV=1
28.64	2	984.01	1966.01	-0.32	34	DLEEATLQHEATAAALRK	F1LMU0	Myosin-4 OS=Rattus norvegicus GN=Myh4 PE=2 SV=1
28.64	2	984.01	1966.01	-0.32	34	DLEEATLQHEATAAALRK	P02564	Myosin-7 OS=Rattus norvegicus GN=Myh7 PE=2 SV=2
28.64	2	984.01	1966.01	-0.32	34	DLEEATLQHEATAAALRK	G3V8B0	Myosin-7 OS=Rattus norvegicus GN=Myh7 PE=4 SV=1
47.59	3	656.65	1966.93	-2.04	55	MMDFETFLPMLQHISK	P16409	Myosin light chain 3 OS=Rattus norvegicus GN=Myl3 PE=2 SV=2

47.59	2	984.47	1966.93	-1.08	75	MMDFETFLPMLQHISK		P16409	Myosin light chain 3 OS=Rattus norvegicus GN=Myl3 PE=2 SV=2
32.98	3	656.98	1967.91	-1.49	53	HKELAPYDENWFYTR		D4A6G6	Protein LOC100362339 OS=Rattus norvegicus GN=LOC100362339 PE=4 SV=1
31.37	2	985.97	1969.92	-0.76	81	HDPSLQPWSVSYDPGSAK		P14141	Carbonic anhydrase 3 OS=Rattus norvegicus GN=Ca3 PE=1 SV=3
14.70	2	986.44	1970.87	-1.44	47	MSMKEVDEQMLNVQNK	[1] Oxidation (M)|[3] Oxidation (M)|[10] Oxidation (M)	G3V7C6	RCG45400 OS=Rattus norvegicus GN=Tubb4b PE=3 SV=1
14.70	2	986.44	1970.87	-1.44	47	MSMKEVDEQMLNVQNK	[1] Oxidation (M)|[3] Oxidation (M)|[10] Oxidation (M)	P85108	Tubulin beta-2A chain OS=Rattus norvegicus GN=Tubb2a PE=1 SV=1
14.70	2	986.44	1970.87	-1.44	47	MSMKEVDEQMLNVQNK	[1] Oxidation (M)|[3] Oxidation (M)|[10] Oxidation (M)	P69897	Tubulin beta-5 chain OS=Rattus norvegicus GN=Tubb5 PE=1 SV=1
35.92	2	986.53	1971.06	-0.64	57	ETPPEGVTAVPLEIPPTPK		M0R9L0	Protein Naca OS=Rattus norvegicus GN=Naca PE=4 SV=1
40.58	3	658.70	1973.07	-1.06	27	ETPPEGVTAVPLEILPSPK		M0R9L0	Protein Naca OS=Rattus norvegicus GN=Naca PE=4 SV=1
40.58	2	987.54	1973.07	-0.05	52	ETPPEGVTAVPLEILPSPK		M0R9L0	Protein Naca OS=Rattus norvegicus GN=Naca PE=4 SV=1
48.83	3	659.35	1975.02	-1.75	51	TLDFEMFLPILQHISR	[6] Oxidation (M)	M0R4E1	Myosin light chain 4 OS=Rattus norvegicus GN=Myl4 PE=4 SV=1
36.09	2	990.39	1978.76	-0.11	85	KEESEESEDDMGFGLFD	[11] Oxidation (M)	P19944	60S acidic ribosomal protein P1 OS=Rattus norvegicus GN=Rplp1 PE=3 SV=1
36.09	2	990.39	1978.76	-0.11	72	KEESEESDEDMGFGLFD	[11] Oxidation (M)	D4A4D5	Protein LOC100362751 OS=Rattus norvegicus GN=LOC498555 PE=3 SV=1

43.95	3	661.98	1982.92	-2.75	36	MMDFETFLPMLQH ISK	[2] Oxidation (M)	P16409	Myosin light chain 3 OS=Rattus norvegicus GN=Myl3 PE=2 SV=2
45.85	2	992.47	1982.93	-1.45	47	MMDFETFLPMLQH ISK	[1] Oxidation (M)	P16409	Myosin light chain 3 OS=Rattus norvegicus GN=Myl3 PE=2 SV=2
40.23	2	994.52	1987.03	-0.03	68	AIAELGIYPAVDPLDSTSR		G3V6D3	ATP synthase subunit beta OS=Rattus norvegicus GN=Atp5b PE=3 SV=1
38.33	3	664.99	1991.95	-1.27	65	GTGGVDTAAVGAVFDISNADR		P00564	Creatine kinase M-type OS=Rattus norvegicus GN=Ckm PE=1 SV=2
38.41	2	996.98	1991.95	-0.04	128	GTGGVDTAAVGAVFDISNADR		P00564	Creatine kinase M-type OS=Rattus norvegicus GN=Ckm PE=1 SV=2
46.23	2	1001.52	2001.03	0.15	32	IGVVQFSNDVFPEFYLK		D4A111	Protein Col6a3 OS=Rattus norvegicus GN=Col6a3 PE=4 SV=2
46.88	3	668.67	2002.98	-1.11	43	FGVEQDVDMVFAS FIRK	[9] Oxidation (M)	P11980	Pyruvate kinase PKM OS=Rattus norvegicus GN=Pkm PE=1 SV=3
47.98	2	1004.00	2005.98	-0.66	49	ALKNNSNDIVNAIM ELTM	[18] Oxidation (M)	M0R9L0	Protein Naca OS=Rattus norvegicus GN=Naca PE=4 SV=1
37.64	2	1004.45	2006.89	-0.06	97	TIGGGDDSFNTFFSETGAGK		Q6P9V9	Tubulin alpha-1B chain OS=Rattus norvegicus GN=Tuba1b PE=1 SV=1
37.64	2	1004.45	2006.89	-0.06	97	TIGGGDDSFNTFFSETGAGK		M0R5B4	Uncharacterized protein OS=Rattus norvegicus PE=4 SV=1
29.86	3	670.72	2009.14	-2.35	44	VYNVTQHAVGIIVNKQVK		D3ZPN7	Protein LOC100360604 OS=Rattus norvegicus GN=LOC100364176 PE=4 SV=1
29.86	3	670.72	2009.14	-2.35	44	VYNVTQHAVGIIVNKQVK		M0RCS9	Uncharacterized protein (Fragment) OS=Rattus norvegicus PE=4 SV=1
29.86	3	670.72	2009.14	-2.35	44	VYNVTQHAVGIIVNKQVK		D3ZRA9	Uncharacterized protein OS=Rattus norvegicus GN=RGD1560414 PE=4 SV=1
49.34	2	1007.50	2012.99	-0.65	123	EAPGPINFTVFLTM FGEK	[14] Oxidation (M)	P08733	Myosin regulatory light chain 2. ventricular/cardiac muscle isoform OS=Rattus norvegicus GN=Myl2 PE=1 SV=2
38.76	3	672.65	2014.92	-1.61	54	MMDFETFLPMLQH ISK	[1] Oxidation (M)|[2] Oxidation	P16409	Myosin light chain 3 OS=Rattus norvegicus GN=Myl3 PE=2 SV=2

							(M)||[10] Oxidation (M)		
38.76	2	1008.47	2014.92	0.41	72	MMDFETFLPMLQHISK	[1] Oxidation (M)||[2] Oxidation (M)||[10] Oxidation (M)	P16409	Myosin light chain 3 OS=Rattus norvegicus GN=Myl3 PE=2 SV=2
44.09	2	1012.00	2021.98	-0.27	80	ALKNNSNDIVNAIMELTM	[14] Oxidation (M)||[18] Oxidation (M)	M0R9L0	Protein Naca OS=Rattus norvegicus GN=Naca PE=4 SV=1
39.02	2	1012.02	2022.03	-0.11	33	ITLPVDFVTADKFDENAK		M0R6Y8	Phosphoglycerate kinase OS=Rattus norvegicus GN=RGD1560402 PE=3 SV=1
40.31	2	1014.07	2026.12	-1.65	40	NHLVEIPPNLPSSLVELR		P47853	Biglycan OS=Rattus norvegicus GN=Bgn PE=2 SV=1
19.19	2	1014.95	2027.89	-1.32	71	ASEEEEIGGPEDSEAVQPR		G3V7Z3	Nucleolar protein 3 OS=Rattus norvegicus GN=Nol3 PE=4 SV=1
23.85	3	679.02	2034.03	-2.04	45	NGMILKPHFHKDWQQR		D3ZD02	60S ribosomal protein L13 OS=Rattus norvegicus GN=RGD1563145 PE=3 SV=1
23.85	3	679.02	2034.03	-2.04	45	NGMILKPHFHKDWQQR		P41123	60S ribosomal protein L13 OS=Rattus norvegicus GN=Rpl13 PE=1 SV=2
34.92	2	1018.55	2035.08	-0.86	62	EVPAAPSVGITAVSEISPSPK		M0R9L0	Protein Naca OS=Rattus norvegicus GN=Naca PE=4 SV=1
45.19	2	1020.57	2039.12	-1.29	61	APPSFFAQVPQAPPVLVFK		P13221	Aspartate aminotransferase. cytoplasmic OS=Rattus norvegicus GN=Got1 PE=1 SV=3
20.23	3	684.35	2050.03	-1.56	26	NGMILKPHFHKDWQQR	[3] Oxidation (M)	D3ZD02	60S ribosomal protein L13 OS=Rattus norvegicus GN=RGD1563145 PE=3 SV=1
20.23	3	684.35	2050.03	-1.56	26	NGMILKPHFHKDWQQR	[3] Oxidation (M)	P41123	60S ribosomal protein L13 OS=Rattus norvegicus GN=Rpl13 PE=1 SV=2
32.82	2	1028.53	2055.05	-1.24	56	LTAQPAPSPEDLALSMGTIK	[16] Oxidation (M)	D4AA52	Alpha-1-inhibitor 3 OS=Rattus norvegicus GN=A1i3 PE=4 SV=2
36.41	3	687.37	2059.08	-1.25	39	YRILNPAAIPEGQFIDSR		G3V885	Myosin-6 OS=Rattus norvegicus GN=Myh6 PE=4 SV=1
36.41	3	687.37	2059.08	-1.25	39	YRILNPAAIPEGQFIDSR		P02564	Myosin-7 OS=Rattus norvegicus GN=Myh7 PE=2 SV=2
36.41	3	687.37	2059.08	-1.25	39	YRILNPAAIPEGQFIDSR		G3V8B0	Myosin-7 OS=Rattus norvegicus GN=Myh7 PE=4 SV=1

36.41	2	1030.55	2059.08	-0.44	58	YRILNPAAIPEGQFIDSR		G3V885	Myosin-6 OS=Rattus norvegicus GN=Myh6 PE=4 SV=1
36.41	2	1030.55	2059.08	-0.44	58	YRILNPAAIPEGQFIDSR		P02564	Myosin-7 OS=Rattus norvegicus GN=Myh7 PE=2 SV=2
36.41	2	1030.55	2059.08	-0.44	58	YRILNPAAIPEGQFIDSR		G3V8B0	Myosin-7 OS=Rattus norvegicus GN=Myh7 PE=4 SV=1
37.20	2	1032.54	2063.07	2.57	26	LLSNLFANYAGADAPVDKGK		P02564	Myosin-7 OS=Rattus norvegicus GN=Myh7 PE=2 SV=2
37.20	2	1032.54	2063.07	2.57	26	LLSNLFANYAGADAPVDKGK		G3V8B0	Myosin-7 OS=Rattus norvegicus GN=Myh7 PE=4 SV=1
26.29	2	1037.00	2071.98	-0.93	76	LSGISMDVAVPENT GQDPAR	[6] Oxidation (M)	F1LTJ5	Protein Hspg2 OS=Rattus norvegicus GN=Hspg2 PE=4 SV=2
54.60	3	692.73	2075.17	-0.54	25	AIPDLTAPVAAVQAAVSNLVR		R9PXU6	Vinculin OS=Rattus norvegicus GN=Vcl PE=4 SV=1
54.52	2	1038.59	2075.17	-0.16	77	AIPDLTAPVAAVQAAVSNLVR		R9PXU6	Vinculin OS=Rattus norvegicus GN=Vcl PE=4 SV=1
44.18	2	1039.52	2077.02	4.78	53	TNGKEPELLEPIPYEFMA		D3ZPN7	Protein LOC100360604 OS=Rattus norvegicus GN=LOC100364176 PE=4 SV=1
43.48	3	693.36	2077.06	-1.51	41	TFGGAPGFSLGSPLSSPVFPR		Q6P725	Desmin OS=Rattus norvegicus GN=Des PE=2 SV=1
43.48	2	1039.54	2077.06	-0.60	98	TFGGAPGFSLGSPLSSPVFPR		Q6P725	Desmin OS=Rattus norvegicus GN=Des PE=2 SV=1
39.53	3	694.03	2079.06	-0.96	38	VFEVSLADLQNDEVAFRK		P49242	40S ribosomal protein S3a OS=Rattus norvegicus GN=Rps3a PE=1 SV=2
41.85	3	696.36	2086.07	-1.39	36	GHYTEGAELVDSVLDVVRK		Q4QQV0	Protein Tubb6 OS=Rattus norvegicus GN=Tubb6 PE=2 SV=1
41.85	3	696.36	2086.07	-1.39	36	GHYTEGAELVDSVLDVVRK		G3V7C6	RCG45400 OS=Rattus norvegicus GN=Tubb4b PE=3 SV=1
41.85	3	696.36	2086.07	-1.39	36	GHYTEGAELVDSVLDVVRK		P85108	Tubulin beta-2A chain OS=Rattus norvegicus GN=Tubb2a PE=1 SV=1
41.85	3	696.36	2086.07	-1.39	36	GHYTEGAELVDSVLDVVRK		Q4QRB4	Tubulin beta-3 chain OS=Rattus norvegicus GN=Tubb3 PE=1 SV=1
41.85	3	696.36	2086.07	-1.39	36	GHYTEGAELVDSVLDVVRK		P69897	Tubulin beta-5 chain OS=Rattus norvegicus GN=Tubb5 PE=1 SV=1
20.07	3	696.67	2086.99	-2.74	31	GYLNKDTHDQLSEPSEVR		P30427	Plectin OS=Rattus norvegicus GN=Plec PE=1 SV=2
20.07	2	1044.50	2086.99	-1.06	79	GYLNKDTHDQLSEPSEVR		P30427	Plectin OS=Rattus norvegicus GN=Plec PE=1 SV=2
28.11	2	1045.49	2088.97	-1.43	30	ESTGAQVQVAGDMLPNSTER		Q4V8F6	Pcbp2 protein OS=Rattus norvegicus GN=Pcbp2 PE=2 SV=1
34.41	2	1047.10	2092.19	-0.11	38	APPIEPAPTPIAAPVTAPVVGK		Q7TN00	Cardiac titin N2BA isoform (Fragment) OS=Rattus norvegicus PE=2 SV=2

34.41	2	1047.10	2092.19	-0.11	38	APPIEPAPTPIAAPVTAPVVGK		Q7TMZ9	Cardiac titin N2B isoform (Fragment) OS=Rattus norvegicus PE=2 SV=1
40.99	2	1047.51	2093.00	-0.23	53	TNGKEPELLEPIPYE FMA	[17] Oxidation (M)	D3ZPN7	Protein LOC100360604 OS=Rattus norvegicus GN=LOC100364176 PE=4 SV=1
21.04	2	1048.00	2093.99	-0.24	66	TVPHAHPATAEYEFANPSR		O35878	Heat shock protein beta-2 OS=Rattus norvegicus GN=Hspb2 PE=3 SV=1
30.28	3	699.04	2094.09	-2.00	47	LSGGHSLHETSTVLVETVTK		D3ZHA0	Protein Flnc OS=Rattus norvegicus GN=Flnc PE=4 SV=1
16.85	2	1048.50	2094.98	-1.04	56	ITYQPSTGEGNEQTTTVGGR		F1LQC3	Collagen alpha-1(XII) chain (Fragment) OS=Rattus norvegicus GN=Col12a1 PE=4 SV=2
16.85	2	1048.50	2094.98	-1.04	56	ITYQPSTGEGNEQTTTVGGR		D3Z9F8	Collagen alpha-1(XII) chain OS=Rattus norvegicus GN=Col12a1 PE=4 SV=2
34.84	2	1052.49	2102.96	-0.42	66	TDKSSASAPDVDDPEAFPALA		Q6AXS5	Plasminogen activator inhibitor 1 RNA-binding protein OS=Rattus norvegicus GN=Serbp1 PE=1 SV=2
21.53	2	1053.49	2104.97	-1.38	107	ESTGAQVQVAGD MLPNSTER	[13] Oxidation (M)	Q4V8F6	Pcbp2 protein OS=Rattus norvegicus GN=Pcbp2 PE=2 SV=1
41.94	2	1057.03	2112.05	-0.90	106	ILLQGTPVAQMAEDAVDGER		M0RBF1	Complement C3 OS=Rattus norvegicus GN=C3 PE=4 SV=1
36.01	2	1057.54	2113.07	-1.68	44	LNKDDPIGNINLAM EIAEK	[14] Oxidation (M)	D3ZCV0	Protein Actn2 OS=Rattus norvegicus GN=Actn2 PE=4 SV=1
48.35	2	1058.01	2114.01	0.70	51	AGTLSITEFADMLSGNAGGFR		P30427	Plectin OS=Rattus norvegicus GN=Plec PE=1 SV=2
15.50	3	706.00	2114.97	-2.00	33	TTHFVEGGDAGNREDQINR		B0K031	60S ribosomal protein L7 OS=Rattus norvegicus GN=Rpl7 PE=2 SV=1
15.50	2	1058.49	2114.97	-1.10	49	TTHFVEGGDAGNREDQINR		B0K031	60S ribosomal protein L7 OS=Rattus norvegicus GN=Rpl7 PE=2 SV=1
34.92	2	1065.03	2128.05	-0.21	77	ILLQGTPVAQMAE DAVDGER	[11] Oxidation (M)	M0RBF1	Complement C3 OS=Rattus norvegicus GN=C3 PE=4 SV=1
34.25	3	710.72	2129.15	-0.79	26	TLSDYNIQKESTLHLVLR		F1LML2	Polyubiquitin-C OS=Rattus norvegicus GN=Ubc PE=2 SV=1
28.56	2	1067.52	2133.03	0.20	112	AGSGIAESQSSGLGGQLSEVSGR		D3ZZ68	Protein Synpo2l OS=Rattus norvegicus GN=Synpo2l PE=4 SV=1
33.73	3	712.69	2135.06	-1.50	70	HAFSPVASVESASGEVLHSPK		F1LM19	Alpha-2-HS-glycoprotein OS=Rattus norvegicus GN=Ahsg PE=4 SV=2

44.68	3	713.75	2138.23	-1.50	27	VAKVEPAVSSIVNSIQVLASK		F1M853	Protein Rrbp1 OS=Rattus norvegicus GN=Rrbp1 PE=4 SV=2
45.56	2	1071.05	2140.10	-1.97	28	SPYLYPLYGLGELPQGFAR		P50399	Rab GDP dissociation inhibitor beta OS=Rattus norvegicus GN=Gdi2 PE=1 SV=2
47.94	3	715.71	2144.12	-1.09	34	ALMLQGVDLLADA VAVTMGPK	[3] Oxidation (M)|[18] Oxidation (M)	P63039	60 kDa heat shock protein. mitochondrial OS=Rattus norvegicus GN=Hspd1 PE=1 SV=1
35.68	2	1073.51	2145.00	-0.72	41	EEELQQTLQQEQNMLER		P30427	Plectin OS=Rattus norvegicus GN=Plec PE=1 SV=2
36.26	2	1075.03	2148.05	-0.48	44	RGTGGVDTAAVGAVFDISNADR		P00564	Creatine kinase M-type OS=Rattus norvegicus GN=Ckm PE=1 SV=2
33.81	3	719.38	2155.13	-1.47	59	AGFAGDDAPRAVFPSIVGRPR		P68035	Actin. alpha cardiac muscle 1 OS=Rattus norvegicus GN=Actc1 PE=2 SV=1
33.81	3	719.38	2155.13	-1.47	59	AGFAGDDAPRAVFPSIVGRPR		P68136	Actin. alpha skeletal muscle OS=Rattus norvegicus GN=Acta1 PE=1 SV=1
33.81	3	719.38	2155.13	-1.47	59	AGFAGDDAPRAVFPSIVGRPR		P60711	Actin. cytoplasmic 1 OS=Rattus norvegicus GN=Actb PE=1 SV=1
33.81	2	1078.57	2155.13	-0.75	28	AGFAGDDAPRAVFPSIVGRPR		P68035	Actin. alpha cardiac muscle 1 OS=Rattus norvegicus GN=Actc1 PE=2 SV=1
33.81	2	1078.57	2155.13	-0.75	28	AGFAGDDAPRAVFPSIVGRPR		P68136	Actin. alpha skeletal muscle OS=Rattus norvegicus GN=Acta1 PE=1 SV=1
33.81	2	1078.57	2155.13	-0.75	28	AGFAGDDAPRAVFPSIVGRPR		P60711	Actin. cytoplasmic 1 OS=Rattus norvegicus GN=Actb PE=1 SV=1
25.35	2	1080.48	2158.95	-0.84	82	EDVPAADLSDQVPDTDSETR		M0RBF1	Complement C3 OS=Rattus norvegicus GN=C3 PE=4 SV=1
16.06	2	1083.48	2164.95	-0.87	126	QQEGEASSQDMTAQVTSPSGK		D3ZHA0	Protein Flnc OS=Rattus norvegicus GN=Flnc PE=4 SV=1
47.71	2	1084.09	2166.16	0.36	40	APPPIAYNPLLSPFFPQAAR		D3ZZ68	Protein Synpo2l OS=Rattus norvegicus GN=Synpo2l PE=4 SV=1
42.03	2	1084.98	2167.95	-0.11	59	GLFDEEMNEILTDPSDDTK		P30427	Plectin OS=Rattus norvegicus GN=Plec PE=1 SV=2
35.92	2	1087.59	2173.16	0.00	30	APAAPSPNEALVAPTEIPTSLK		M0R9L0	Protein Naca OS=Rattus norvegicus GN=Naca PE=4 SV=1

48.78	3	726.36	2176.07	-1.34	52	GADPEETILNAFKVFDPEGK		P08733	Myosin regulatory light chain 2. ventricular/cardiac muscle isoform OS=Rattus norvegicus GN=Myl2 PE=1 SV=2
24.78	2	1089.49	2176.96	-0.05	111	LYGSGGPPPTGEEDTSEKDEL		P06761	78 kDa glucose-regulated protein OS=Rattus norvegicus GN=Hspa5 PE=1 SV=1
7.23	2	1091.48	2180.95	-1.34	114	QQEGEASSQDMTAQVTSPSGK	[11] Oxidation (M)	D3ZHA0	Protein Flnc OS=Rattus norvegicus GN=Flnc PE=4 SV=1
37.73	3	729.36	2185.06	-0.36	43	DFSPEDIIVTTSNNHIEVR		B5DFG4	Heat shock 27kD protein family. member 7 (Cardiovascular) OS=Rattus norvegicus GN=Hspb7 PE=2 SV=1
18.95	2	1095.46	2188.90	-0.79	135	NMGGPYGGGNYGPGGSGGSGGYGGR		M0R6J9	Heterogeneous nuclear ribonucleoproteins A2/B1 OS=Rattus norvegicus GN=Hnrnpa2b1 PE=4 SV=1
42.90	3	731.07	2190.19	-0.94	26	DIVPGDIVEIAVGDKVPADIR		E9PSX6	Sarcoplasmic/endoplasmic reticulum calcium ATPase 2 OS=Rattus norvegicus GN=Atp2a2 PE=3 SV=2
42.90	2	1096.10	2190.19	-0.09	76	DIVPGDIVEIAVGDKVPADIR		E9PSX6	Sarcoplasmic/endoplasmic reticulum calcium ATPase 2 OS=Rattus norvegicus GN=Atp2a2 PE=3 SV=2
41.25	3	734.05	2199.12	-0.60	66	GTLEDQIIQANPALEAFGNAK		G3V885	Myosin-6 OS=Rattus norvegicus GN=Myh6 PE=4 SV=1
41.25	3	734.05	2199.12	-0.60	66	GTLEDQIIQANPALEAFGNAK		P02564	Myosin-7 OS=Rattus norvegicus GN=Myh7 PE=2 SV=2
41.25	3	734.05	2199.12	-0.60	66	GTLEDQIIQANPALEAFGNAK		G3V8B0	Myosin-7 OS=Rattus norvegicus GN=Myh7 PE=4 SV=1
41.25	2	1100.57	2199.12	-0.29	101	GTLEDQIIQANPALEAFGNAK		G3V885	Myosin-6 OS=Rattus norvegicus GN=Myh6 PE=4 SV=1
41.25	2	1100.57	2199.12	-0.29	101	GTLEDQIIQANPALEAFGNAK		P02564	Myosin-7 OS=Rattus norvegicus GN=Myh7 PE=2 SV=2
41.25	2	1100.57	2199.12	-0.29	101	GTLEDQIIQANPALEAFGNAK		G3V8B0	Myosin-7 OS=Rattus norvegicus GN=Myh7 PE=4 SV=1
47.89	2	1101.08	2200.14	-0.61	42	GTLEDQIISANPLLEAFGNAK		G3V6D8	Myosin-3 OS=Rattus norvegicus GN=Myh3 PE=4 SV=1
22.16	2	1102.00	2201.98	-0.16	51	ESEGSPDTDAAPGPDTDVTLTK		Q5M7W5	Microtubule-associated protein 4 OS=Rattus norvegicus GN=Map4 PE=1 SV=1
15.99	2	1103.45	2204.89	0.01	130	NMGGPYGGGNYGPGGSGGSGGYGGR	[2] Oxidation (M)	M0R6J9	Heterogeneous nuclear ribonucleoproteins A2/B1 OS=Rattus norvegicus GN=Hnrnpa2b1 PE=4 SV=1
34.07	3	737.71	2210.09	-0.81	45	LGEHNINVLEGDEQFINAAK		P00762	Anionic trypsin-1 OS=Rattus norvegicus GN=Prss1 PE=1 SV=1
34.07	2	1106.06	2210.10	0.38	78	LGEHNINVLEGDEQFINAAK		P00762	Anionic trypsin-1 OS=Rattus norvegicus GN=Prss1 PE=1 SV=1

37.37	2	1108.04	2214.06	-0.04	86	DLYANTVLSGGTTMYPGIADR		P60711	Actin. cytoplasmic 1 OS=Rattus norvegicus GN=Actb PE=1 SV=1
18.10	3	741.35	2221.02	14.50	36	DMDPEEKEIVVWV CQEEK	[2] Oxidation (M)	F1LNM7	Ras association domain-containing protein 9 (Fragment) OS=Rattus norvegicus GN=Rassf9 PE=4 SV=1
36.68	2	1114.54	2227.06	-0.86	97	DLYANNVLSGGTTMYPGIADR		P68035	Actin. alpha cardiac muscle 1 OS=Rattus norvegicus GN=Actc1 PE=2 SV=1
34.67	2	1116.04	2230.06	-0.41	107	DLYANTVLSGGTT MYPGIADR	[14] Oxidation (M)	P60711	Actin. cytoplasmic 1 OS=Rattus norvegicus GN=Actb PE=1 SV=1
34.67	3	744.36	2230.06	-0.29	57	DLYANTVLSGGTT MYPGIADR	[14] Oxidation (M)	P60711	Actin. cytoplasmic 1 OS=Rattus norvegicus GN=Actb PE=1 SV=1
14.78	2	1121.51	2241.01	-1.10	58	QQPVSESPPTDEAAGSGGSEVGR		Q4G061	Eukaryotic translation initiation factor 3 subunit B OS=Rattus norvegicus GN=Eif3b PE=1 SV=1
34.16	2	1122.53	2243.05	-1.00	82	DLYANNVLSGGTT MYPGIADR	[14] Oxidation (M)	P68035	Actin. alpha cardiac muscle 1 OS=Rattus norvegicus GN=Actc1 PE=2 SV=1
34.58	2	1123.51	2245.01	-1.48	104	DLYANNVMSGGTTMYPGIADR		P68136	Actin. alpha skeletal muscle OS=Rattus norvegicus GN=Acta1 PE=1 SV=1
34.07	2	1129.52	2257.03	-0.33	100	YTPSGQSGAAASESLFISNHAY		P05065	Fructose-bisphosphate aldolase A OS=Rattus norvegicus GN=Aldoa PE=1 SV=2
31.72	2	1131.51	2261.01	-0.31	123	DLYANNVMSGGTT MYPGIADR	[14] Oxidation (M)	P68136	Actin. alpha skeletal muscle OS=Rattus norvegicus GN=Acta1 PE=1 SV=1
30.45	2	1131.51	2261.01	0.31	110	DLYANNVMSGGTT MYPGIADR	[8] Oxidation (M)	P68136	Actin. alpha skeletal muscle OS=Rattus norvegicus GN=Acta1 PE=1 SV=1
46.04	2	1136.57	2271.13	0.25	55	AVANETGAFFFLIN GPEIMSK	[19] Oxidation (M)	P46462	Transitional endoplasmic reticulum ATPase OS=Rattus norvegicus GN=Vcp PE=1 SV=3
41.08	2	1139.02	2276.03	-0.44	72	WGDAGAEYVVESTGVFTTMEK		M0R590	Protein LOC685186 OS=Rattus norvegicus GN=LOC685186 PE=3 SV=1
27.04	3	760.01	2277.00	-0.21	66	DLYANNVMSGGTT MYPGIADR	[8] Oxidation (M)|[14] Oxidation (M)	P68136	Actin. alpha skeletal muscle OS=Rattus norvegicus GN=Acta1 PE=1 SV=1
27.04	2	1139.51	2277.01	0.49	90	DLYANNVMSGGTT MYPGIADR	[8] Oxidation (M)|[14] Oxidation (M)	P68136	Actin. alpha skeletal muscle OS=Rattus norvegicus GN=Acta1 PE=1 SV=1

40.49	2	1143.57	2285.13	-4.30	58	EISPDTTLLDLQNNDISELR		P47853	Biglycan OS=Rattus norvegicus GN=Bgn PE=2 SV=1
29.30	3	766.41	2296.22	-0.82	62	VAPEEHPTLLTEAPLNPKANR		P68035	Actin. alpha cardiac muscle 1 OS=Rattus norvegicus GN=Actc1 PE=2 SV=1
29.30	3	766.41	2296.22	-0.82	62	VAPEEHPTLLTEAPLNPKANR		P68136	Actin. alpha skeletal muscle OS=Rattus norvegicus GN=Acta1 PE=1 SV=1
29.30	2	1149.12	2296.22	-0.67	79	VAPEEHPTLLTEAPLNPKANR		P68035	Actin. alpha cardiac muscle 1 OS=Rattus norvegicus GN=Actc1 PE=2 SV=1
29.30	2	1149.12	2296.22	-0.67	79	VAPEEHPTLLTEAPLNPKANR		P68136	Actin. alpha skeletal muscle OS=Rattus norvegicus GN=Acta1 PE=1 SV=1
28.41	3	769.42	2305.22	-1.97	51	TTLTGLRPGTEYGVGVSAVKGDK		M0RA80	Protein Tnc OS=Rattus norvegicus GN=Tnc PE=4 SV=1
28.97	3	771.36	2311.07	-1.06	54	QTNPSAMEVEEDDPVPEIRR		P46462	Transitional endoplasmic reticulum ATPase OS=Rattus norvegicus GN=Vcp PE=1 SV=3
47.88	2	1162.56	2323.11	-0.93	95	VEESTQVGGDPFPAVFGDFLGR		D3ZHA0	Protein Flnc OS=Rattus norvegicus GN=Flnc PE=4 SV=1
26.29	3	776.70	2327.07	-1.25	39	QTNPSAMEVEEDD PVPEIRR	[7] Oxidation (M)	P46462	Transitional endoplasmic reticulum ATPase OS=Rattus norvegicus GN=Vcp PE=1 SV=3
29.94	2	1174.96	2347.90	-0.01	43	DYEEVGVDSVEGEGEEEGEEY		Q6P9V9	Tubulin alpha-1B chain OS=Rattus norvegicus GN=Tuba1b PE=1 SV=1
42.76	2	1175.10	2348.18	-0.59	58	EAETALPIEMDLAPPEDVALPK		Q5M7W5	Microtubule-associated protein 4 OS=Rattus norvegicus GN=Map4 PE=1 SV=1
37.03	2	1175.11	2348.21	0.85	59	ETPTNPSPEGVTAAPLEIPLTSK		M0R9L0	Protein Naca OS=Rattus norvegicus GN=Naca PE=4 SV=1
20.23	3	784.36	2350.07	-0.97	69	HQGVMVGMGQKDSYVGDEAQSK		P68035	Actin. alpha cardiac muscle 1 OS=Rattus norvegicus GN=Actc1 PE=2 SV=1
20.23	3	784.36	2350.07	-0.97	69	HQGVMVGMGQKDSYVGDEAQSK		P68136	Actin. alpha skeletal muscle OS=Rattus norvegicus GN=Acta1 PE=1 SV=1
20.23	3	784.36	2350.07	-0.97	69	HQGVMVGMGQKDSYVGDEAQSK		P60711	Actin. cytoplasmic 1 OS=Rattus norvegicus GN=Actb PE=1 SV=1
31.72	3	785.72	2354.13	-0.60	41	DIRHDPSLQPWSVSYDPGSAK		P14141	Carbonic anhydrase 3 OS=Rattus norvegicus GN=Ca3 PE=1 SV=3
31.72	2	1178.07	2354.13	-0.03	53	DIRHDPSLQPWSVSYDPGSAK		P14141	Carbonic anhydrase 3 OS=Rattus norvegicus GN=Ca3 PE=1 SV=3

51.85	2	1183.13	2364.24	0.34	36	GAVPYVQAFDSLLANPVAEYLK		Q08163	Adenylyl cyclase-associated protein 1 OS=Rattus norvegicus GN=Cap1 PE=1 SV=3
15.99	3	789.69	2366.06	-1.37	41	HQGVMVGMGQK DSYVGDEAQSK	[5] Oxidation (M)	P68035	Actin. alpha cardiac muscle 1 OS=Rattus norvegicus GN=Actc1 PE=2 SV=1
15.99	3	789.69	2366.06	-1.37	41	HQGVMVGMGQK DSYVGDEAQSK	[5] Oxidation (M)	P68136	Actin. alpha skeletal muscle OS=Rattus norvegicus GN=Acta1 PE=1 SV=1
15.99	3	789.69	2366.06	-1.37	41	HQGVMVGMGQK DSYVGDEAQSK	[5] Oxidation (M)	P60711	Actin. cytoplasmic 1 OS=Rattus norvegicus GN=Actb PE=1 SV=1
27.63	3	789.72	2366.14	-1.24	28	LKPDSPYTITVSSQYPDGEGGR		F1LQC3	Collagen alpha-1(XII) chain (Fragment) OS=Rattus norvegicus GN=Col12a1 PE=4 SV=2
27.63	3	789.72	2366.14	-1.24	28	LKPDSPYTITVSSQYPDGEGGR		D3Z9F8	Collagen alpha-1(XII) chain OS=Rattus norvegicus GN=Col12a1 PE=4 SV=2
38.93	3	794.75	2381.22	-0.59	57	LAMLQEHVESLGPI SDLASTVR	[3] Oxidation (M)	D3ZH41	Cytoskeleton-associated protein 4 (Predicted) OS=Rattus norvegicus GN=Ckap4 PE=4 SV=2
13.86	3	795.03	2382.06	-0.97	50	HQGVMVGMGQK DSYVGDEAQSK	[5] Oxidation (M)|[8] Oxidation (M)	P68035	Actin. alpha cardiac muscle 1 OS=Rattus norvegicus GN=Actc1 PE=2 SV=1
13.86	3	795.03	2382.06	-0.97	50	HQGVMVGMGQK DSYVGDEAQSK	[5] Oxidation (M)|[8] Oxidation (M)	P68136	Actin. alpha skeletal muscle OS=Rattus norvegicus GN=Acta1 PE=1 SV=1
13.86	3	795.03	2382.06	-0.97	50	HQGVMVGMGQK DSYVGDEAQSK	[5] Oxidation (M)|[8] Oxidation (M)	P60711	Actin. cytoplasmic 1 OS=Rattus norvegicus GN=Actb PE=1 SV=1
13.86	3	795.03	2382.06	6.46	28	HQGVMVGMGQK DCYVGDEAQSK	[5] Oxidation (M)	D3ZRN3	Protein Actbl2 OS=Rattus norvegicus GN=Actbl2 PE=3 SV=1
13.86	2	1192.04	2382.06	-0.33	52	HQGVMVGMGQK DSYVGDEAQSK	[5] Oxidation (M)|[8] Oxidation (M)	P68035	Actin. alpha cardiac muscle 1 OS=Rattus norvegicus GN=Actc1 PE=2 SV=1
13.86	2	1192.04	2382.06	-0.33	52	HQGVMVGMGQK DSYVGDEAQSK	[5] Oxidation (M)|[8] Oxidation (M)	P68136	Actin. alpha skeletal muscle OS=Rattus norvegicus GN=Acta1 PE=1 SV=1

13.86	2	1192.04	2382.06	-0.33	52	HQGVMVGMGQK DSYVGDEAQSK	[5] Oxidation (M)|[8] Oxidation (M)	P60711	Actin. cytoplasmic 1 OS=Rattus norvegicus GN=Actb PE=1 SV=1
39.18	2	1195.11	2388.21	-0.37	42	VAGPQPAQTGVAQASLGEYLFER		Q7TP54	Ab2-162 OS=Rattus norvegicus GN=Fam65b PE=2 SV=1
23.93	3	802.71	2405.10	-0.51	92	KDLYANNVMSGGT TMYPGIADR	[9] Oxidation (M)|[15] Oxidation (M)	P68136	Actin. alpha skeletal muscle OS=Rattus norvegicus GN=Acta1 PE=1 SV=1
23.93	2	1203.56	2405.10	-0.31	103	KDLYANNVMSGGT TMYPGIADR	[9] Oxidation (M)|[15] Oxidation (M)	P68136	Actin. alpha skeletal muscle OS=Rattus norvegicus GN=Acta1 PE=1 SV=1
31.72	3	805.74	2414.20	-0.88	59	QLFHPEQLITGKEDAANNYAR		Q6P9V9	Tubulin alpha-1B chain OS=Rattus norvegicus GN=Tuba1b PE=1 SV=1
31.72	3	805.74	2414.20	-0.88	59	QLFHPEQLITGKEDAANNYAR		Q5XIF6	Tubulin alpha-4A chain OS=Rattus norvegicus GN=Tuba4a PE=2 SV=1
31.72	3	805.74	2414.20	-0.88	59	QLFHPEQLITGKEDAANNYAR		M0R5B4	Uncharacterized protein OS=Rattus norvegicus PE=4 SV=1
42.90	3	808.09	2421.24	0.16	56	IANDHSLNHEYLPILGLAEFR		P13221	Aspartate aminotransferase. cytoplasmic OS=Rattus norvegicus GN=Got1 PE=1 SV=3
14.63	2	1216.55	2431.08	-0.58	92	EAEAAEPEQPEQPEQPAAEEPR		F1LMW7	Myristoylated alanine-rich C-kinase substrate OS=Rattus norvegicus GN=Marcks PE=4 SV=2
45.09	2	1221.08	2440.14	-0.67	139	TASEMVLADDNFST IVAAVEEGR	[5] Oxidation (M)	E9PSX6	Sarcoplasmic/endoplasmic reticulum calcium ATPase 2 OS=Rattus norvegicus GN=Atp2a2 PE=3 SV=2
45.09	2	1221.08	2440.14	-0.67	139	TASEMVLADDNFST IVAAVEEGR	[5] Oxidation (M)	B4F7E5	ATPase. Ca++ transporting. cardiac muscle. fast twitch 1 OS=Rattus norvegicus GN=Atp2a1 PE=2 SV=1
45.09	2	1221.08	2440.14	-0.67	139	TASEMVLADDNFST IVAAVEEGR	[5] Oxidation (M)	M0RCD2	Sarcoplasmic/endoplasmic reticulum calcium ATPase 1 OS=Rattus norvegicus GN=Atp2a1 PE=3 SV=1
45.09	3	814.39	2440.14	-0.05	47	TASEMVLADDNFST IVAAVEEGR	[5] Oxidation (M)	E9PSX6	Sarcoplasmic/endoplasmic reticulum calcium ATPase 2 OS=Rattus norvegicus GN=Atp2a2 PE=3 SV=2
45.09	3	814.39	2440.14	-0.05	47	TASEMVLADDNFST IVAAVEEGR	[5] Oxidation (M)	B4F7E5	ATPase. Ca++ transporting. cardiac muscle. fast twitch 1 OS=Rattus norvegicus GN=Atp2a1 PE=2 SV=1
45.09	3	814.39	2440.14	-0.05	47	TASEMVLADDNFST IVAAVEEGR	[5] Oxidation (M)	M0RCD2	Sarcoplasmic/endoplasmic reticulum calcium ATPase 1 OS=Rattus norvegicus GN=Atp2a1 PE=3 SV=1

9.62	3	815.72	2444.14	-1.43	25	AANAASGAGGSSAAAGSRPGDGGSL GSGAR		Q5XIM5	Protein CDV3 homolog OS=Rattus norvegicus GN=Cdv3 PE=2 SV=1
35.75	2	1224.60	2447.19	-1.29	38	NLQPDTPYTVTVVPVYAEGDGGR		F1LQC3	Collagen alpha-1(XII) chain (Fragment) OS=Rattus norvegicus GN=Col12a1 PE=4 SV=2
35.75	2	1224.60	2447.19	-1.29	38	NLQPDTPYTVTVVPVYAEGDGGR		D3Z9F8	Collagen alpha-1(XII) chain OS=Rattus norvegicus GN=Col12a1 PE=4 SV=2
32.65	3	823.80	2468.37	-1.13	40	TMKAPPIEPAPTPIA APVTAPVVGK	[2] Oxidation (M)	Q7TN00	Cardiac titin N2BA isoform (Fragment) OS=Rattus norvegicus PE=2 SV=2
32.65	3	823.80	2468.37	-1.13	40	TMKAPPIEPAPTPIA APVTAPVVGK	[2] Oxidation (M)	Q7TMZ9	Cardiac titin N2B isoform (Fragment) OS=Rattus norvegicus PE=2 SV=1
26.29	3	825.71	2474.10	-0.92	49	QFHLHWGSSDDHGSEHTVDGVK		P14141	Carbonic anhydrase 3 OS=Rattus norvegicus GN=Ca3 PE=1 SV=3
47.71	2	1238.62	2475.23	-0.59	60	MQGTLEDQIISANP LLEAFGNAK	[1] Oxidation (M)	F1LMU0	Myosin-4 OS=Rattus norvegicus GN=Myh4 PE=2 SV=1
47.71	2	1238.62	2475.23	-0.59	60	MQGTLEDQIISANP LLEAFGNAK	[1] Oxidation (M)	F1M8F6	Myosin-8 (Fragment) OS=Rattus norvegicus GN=Myh8 PE=4 SV=2
47.71	2	1238.62	2475.23	-0.59	60	MQGTLEDQIISANP LLEAFGNAK	[1] Oxidation (M)	F1LRV9	Protein Myh1 OS=Rattus norvegicus GN=Myh1 PE=2 SV=2
47.71	2	1238.62	2475.23	-0.59	60	MQGTLEDQIISANP LLEAFGNAK	[1] Oxidation (M)	G3V6E1	Uncharacterized protein OS=Rattus norvegicus GN=Myh2 PE=4 SV=2
45.29	3	826.10	2475.27	-0.97	34	MKGTLEDQIISANP LLEAFGNAK	[1] Oxidation (M)	G3V6D8	Myosin-3 OS=Rattus norvegicus GN=Myh3 PE=4 SV=1
45.29	3	826.10	2475.27	13.74	34	MQGTLEDQIISANP LLEAFGNAK	[1] Oxidation (M)	F1LMU0	Myosin-4 OS=Rattus norvegicus GN=Myh4 PE=2 SV=1
45.29	3	826.10	2475.27	13.74	34	MQGTLEDQIISANP LLEAFGNAK	[1] Oxidation (M)	F1M8F6	Myosin-8 (Fragment) OS=Rattus norvegicus GN=Myh8 PE=4 SV=2
45.29	3	826.10	2475.27	13.74	34	MQGTLEDQIISANP LLEAFGNAK	[1] Oxidation (M)	F1LRV9	Protein Myh1 OS=Rattus norvegicus GN=Myh1 PE=2 SV=2
45.29	3	826.10	2475.27	13.74	34	MQGTLEDQIISANP LLEAFGNAK	[1] Oxidation (M)	G3V6E1	Uncharacterized protein OS=Rattus norvegicus GN=Myh2 PE=4 SV=2
33.89	2	1247.11	2492.21	-0.76	114	ITYVPVTGGPPSMVTVDGTDTETR		M0RA80	Protein Tnc OS=Rattus norvegicus GN=Tnc PE=4 SV=1
40.58	3	832.13	2493.36	-0.39	26	EIPSNEVTAVPLEISLPLKETSK		M0R9L0	Protein Naca OS=Rattus norvegicus GN=Naca PE=4 SV=1

40.58	2	1247.69	2493.36	0.15	29	EIPSNEVTAVPLEISLPLKETSK		M0R9L0	Protein Naca OS=Rattus norvegicus GN=Naca PE=4 SV=1
22.33	3	833.72	2498.14	-0.96	58	ILAQMTGTEYMQD PDEEALRR	[5] Oxidation (M)|[11] Oxidation (M)	Q5XIG1	Ldb3 protein OS=Rattus norvegicus GN=Ldb3 PE=2 SV=1
41.85	3	833.77	2498.30	-0.66	73	TTYLNSFSHVGTGIVHAINNVVR		F1LNH3	Procollagen. type VI. alpha 2. isoform CRA_a OS=Rattus norvegicus GN=Col6a2 PE=4 SV=2
36.76	3	834.44	2500.29	-0.81	41	GLENNVNVELLNALHSHMVNKR		D3ZAF5	Periostin. osteoblast specific factor (Predicted). isoform CRA_a OS=Rattus norvegicus GN=Postn PE=4 SV=1
30.28	2	1255.11	2508.20	-0.80	122	ITYVPVTGGPPSMV TVDGTDTETR	[13] Oxidation (M)	M0RA80	Protein Tnc OS=Rattus norvegicus GN=Tnc PE=4 SV=1
24.43	3	839.39	2515.16	-0.08	78	DNGDGTHTVHYTPATDGPYTVAVK		D3ZHA0	Protein Flnc OS=Rattus norvegicus GN=Flnc PE=4 SV=1
34.50	3	839.77	2516.29	-0.48	48	GLENNVNVELLNAL HSHMVNKR	[18] Oxidation (M)	D3ZAF5	Periostin. osteoblast specific factor (Predicted). isoform CRA_a OS=Rattus norvegicus GN=Postn PE=4 SV=1
31.72	3	841.06	2520.16	-0.81	33	EWGYASHNGPEHWHELYPIAK		P14141	Carbonic anhydrase 3 OS=Rattus norvegicus GN=Ca3 PE=1 SV=3
31.72	2	1261.09	2520.16	0.12	53	EWGYASHNGPEHWHELYPIAK		P14141	Carbonic anhydrase 3 OS=Rattus norvegicus GN=Ca3 PE=1 SV=3
38.24	3	841.12	2520.33	-0.36	65	VGNEYVTKGQNVQQVAYAIGALAK		P02564	Myosin-7 OS=Rattus norvegicus GN=Myh7 PE=2 SV=2
38.24	3	841.12	2520.33	-0.36	65	VGNEYVTKGQNVQQVAYAIGALAK		G3V8B0	Myosin-7 OS=Rattus norvegicus GN=Myh7 PE=4 SV=1
44.76	2	1262.19	2522.37	-0.73	42	TLTAVHDAILEDLVFPSEIVGKR		B5DEL9	RCG62292. isoform CRA_a OS=Rattus norvegicus GN=Rps7 PE=2 SV=1
44.76	3	841.80	2522.37	-0.64	61	TLTAVHDAILEDLVFPSEIVGKR		B5DEL9	RCG62292. isoform CRA_a OS=Rattus norvegicus GN=Rps7 PE=2 SV=1
37.20	2	1266.19	2530.37	0.09	34	VETGVLKPGMVVT FAPVNVTTEVK	[10] Oxidation (M)	F1M6C2	Elongation factor 1-alpha (Fragment) OS=Rattus norvegicus GN=LOC100360150 PE=3 SV=1
37.20	3	844.47	2530.38	4.58	58	VETGVLKPGMVVT FAPVNVTTEVK	[10] Oxidation (M)	F1M6C2	Elongation factor 1-alpha (Fragment) OS=Rattus norvegicus GN=LOC100360150 PE=3 SV=1
25.10	3	848.10	2541.27	-1.07	27	SHVEDGDVAGSPAVPPAEQDPVKLK		F1M853	Protein Rrbp1 OS=Rattus norvegicus GN=Rrbp1 PE=4 SV=2

30.02	3	858.08	2571.22	-0.74	53	TDLNHENLKGGDDLDPNYVLSSR		P00564	Creatine kinase M-type OS=Rattus norvegicus GN=Ckm PE=1 SV=2
31.88	3	858.12	2571.33	-0.03	30	TKTETITGFQVDAIPANGQTPVQR		F1LST1	Fibronectin OS=Rattus norvegicus GN=Fn1 PE=4 SV=2
40.06	2	1294.21	2586.41	0.97	30	VETGILRPGMVVTFAPVNITTEVK	[10] Oxidation (M)	P62632	Elongation factor 1-alpha 2 OS=Rattus norvegicus GN=Eef1a2 PE=2 SV=1
17.70	3	865.73	2594.16	-2.10	50	GNPVIAHQEEAEDVSEEAPMRDR	[20] Oxidation (M)	F1LWG8	5-hydroxytryptamine receptor 2B OS=Rattus norvegicus GN=Srl PE=2 SV=2
46.08	3	865.73	2594.16	-0.83	87	AYEDAAEEFHPYIPFFATFDSK		P19633	Calsequestrin-1 OS=Rattus norvegicus GN=Casq1 PE=1 SV=2
46.08	2	1298.09	2594.16	0.11	39	AYEDAAEEFHPYIPFFATFDSK		P19633	Calsequestrin-1 OS=Rattus norvegicus GN=Casq1 PE=1 SV=2
47.93	3	865.79	2594.35	-0.75	60	VIHDNFGIVEGLMTTVHAITATQK		M0R590	Protein LOC685186 OS=Rattus norvegicus GN=LOC685186 PE=3 SV=1
47.93	3	865.79	2594.35	-0.75	60	VIHDNFGIVEGLMTTVHAITATQK		M0R451	Uncharacterized protein OS=Rattus norvegicus PE=3 SV=1
37.98	3	870.09	2607.26	0.02	42	GIWHNDNKSFLVWVNEEDHLR		P00564	Creatine kinase M-type OS=Rattus norvegicus GN=Ckm PE=1 SV=2
42.76	4	653.59	2610.34	-1.77	67	VIHDNFGIVEGLMTTVHAITATQK	[13] Oxidation (M)	M0R590	Protein LOC685186 OS=Rattus norvegicus GN=LOC685186 PE=3 SV=1
42.76	4	653.59	2610.34	-1.77	67	VIHDNFGIVEGLMTTVHAITATQK	[13] Oxidation (M)	M0R451	Uncharacterized protein OS=Rattus norvegicus PE=3 SV=1
42.76	3	871.12	2610.35	-0.52	103	VIHDNFGIVEGLMTTVHAITATQK	[13] Oxidation (M)	M0R590	Protein LOC685186 OS=Rattus norvegicus GN=LOC685186 PE=3 SV=1
42.76	3	871.12	2610.35	-0.52	103	VIHDNFGIVEGLMTTVHAITATQK	[13] Oxidation (M)	M0R451	Uncharacterized protein OS=Rattus norvegicus PE=3 SV=1
42.76	3	871.12	2610.35	-0.52	52	VIHDNYGIVEGLMTTVHAITATQK		D3Z8U8	Uncharacterized protein OS=Rattus norvegicus PE=4 SV=1
42.76	2	1306.18	2610.35	0.33	104	VIHDNFGIVEGLMTTVHAITATQK	[13] Oxidation (M)	M0R590	Protein LOC685186 OS=Rattus norvegicus GN=LOC685186 PE=3 SV=1
42.76	2	1306.18	2610.35	0.33	104	VIHDNFGIVEGLMTTVHAITATQK	[13] Oxidation (M)	M0R451	Uncharacterized protein OS=Rattus norvegicus PE=3 SV=1
42.76	2	1306.18	2610.35	0.33	28	VIHDNYGIVEGLMTTVHAITATQK		D3Z8U8	Uncharacterized protein OS=Rattus norvegicus PE=4 SV=1

48.40	3	872.83	2615.48	-0.34	43	AVPPEEIPPAVAPSIPLLLPLPEEK		Q7TN00	Cardiac titin N2BA isoform (Fragment) OS=Rattus norvegicus PE=2 SV=2
48.58	2	1308.75	2615.48	0.03	78	AVPPEEIPPAVAPSIPLLLPLPEEK		Q7TN00	Cardiac titin N2BA isoform (Fragment) OS=Rattus norvegicus PE=2 SV=2
44.00	2	1312.69	2623.37	-0.93	70	IPADVDPLTITSSLSSDGVLTVNGPR		P23928	Alpha-crystallin B chain OS=Rattus norvegicus GN=Cryab PE=1 SV=1
44.00	3	875.46	2623.37	-0.39	58	IPADVDPLTITSSLSSDGVLTVNGPR		P23928	Alpha-crystallin B chain OS=Rattus norvegicus GN=Cryab PE=1 SV=1
48.90	3	879.14	2634.39	-0.41	31	NANPSELEQIVPSPAFILAAESLPK		D4A111	Protein Col6a3 OS=Rattus norvegicus GN=Col6a3 PE=4 SV=2
44.50	3	879.47	2635.40	-0.52	33	ALVPAAELLDSGVISHEVYQQLQR		P30427	Plectin OS=Rattus norvegicus GN=Plec PE=1 SV=2
18.95	3	887.45	2659.32	-1.38	59	AAPAPAAAPAAAPEPERPKEAEFDASK		P16409	Myosin light chain 3 OS=Rattus norvegicus GN=Myl3 PE=2 SV=2
47.29	3	891.17	2670.49	-2.49	33	NGVIHLIDEVLIPDSAKQVIELAGK		D3ZAF5	Periostin. osteoblast specific factor (Predicted). isoform CRA_a OS=Rattus norvegicus GN=Postn PE=4 SV=1
41.85	3	893.46	2677.35	-0.40	103	LGSSEVEQVQLVVDGVKLMVEMEK	[19] Oxidation (M)|[22] Oxidation (M)	P00564	Creatine kinase M-type OS=Rattus norvegicus GN=Ckm PE=1 SV=2
24.25	3	894.40	2680.19	-0.82	70	DLYANNVMSGGTTMYPGIADRMQK	[8] Oxidation (M)|[14] Oxidation (M)|[22] Oxidation (M)	P68136	Actin. alpha skeletal muscle OS=Rattus norvegicus GN=Acta1 PE=1 SV=1
42.25	2	1343.69	2685.37	-0.56	26	LEQQVPVNQVFGQDEMIDVIGVTK		P21531	60S ribosomal protein L3 OS=Rattus norvegicus GN=Rpl3 PE=1 SV=3
36.85	3	905.07	2712.20	-0.34	62	TSDSDIFTDIENPSSHVPEFSSSSK		A1L114	Fga protein OS=Rattus norvegicus GN=Fga PE=2 SV=1
27.38	3	906.10	2715.27	-0.62	68	NNRQPYAVSELAGHQTSAESWGTGR		Q6P3V9	60S ribosomal protein L4 OS=Rattus norvegicus GN=Rpl4 PE=2 SV=1
39.27	3	911.81	2732.40	0.08	34	KTFSHELSDFGLESTTGEIPVVAIR		P11598	Protein disulfide-isomerase A3 OS=Rattus norvegicus GN=Pdia3 PE=1 SV=2
41.51	2	1372.23	2742.45	-0.45	43	SLFASAENEPPVPLVGNWRPPQPIK		P14141	Carbonic anhydrase 3 OS=Rattus norvegicus GN=Ca3 PE=1 SV=3

41.51	3	915.16	2742.45	0.08	83	SLFASAENEPPVPLVGNWRPPQPIK		P14141	Carbonic anhydrase 3 OS=Rattus norvegicus GN=Ca3 PE=1 SV=3
27.04	3	915.44	2743.29	0.03	37	LRQFHLHWGSSDDHGSEHTVDGVK		P14141	Carbonic anhydrase 3 OS=Rattus norvegicus GN=Ca3 PE=1 SV=3
24.86	3	917.72	2750.13	-0.80	27	SLTMVEDNDDEEE DGDELLHHHR	[4] Oxidation (M)	G3V8L3	Lamin A. isoform CRA_b OS=Rattus norvegicus GN=Lmna PE=3 SV=1
26.21	3	925.48	2773.42	0.04	38	LASVPAGGAVAVSAAPGSAAPAAGSA PAAAEEK		D4A4D5	Protein LOC100362751 OS=Rattus norvegicus GN=LOC498555 PE=3 SV=1
26.21	3	925.48	2773.42	0.04	38	LASVPAGGAVAVSAAPGSAAPAAGSA PAAAEEK		P02401	60S acidic ribosomal protein P2 OS=Rattus norvegicus GN=Rplp2 PE=1 SV=2
26.21	2	1387.72	2773.43	0.76	35	LASVPAGGAVAVSAAPGSAAPAAGSA PAAAEEK		D4A4D5	Protein LOC100362751 OS=Rattus norvegicus GN=LOC498555 PE=3 SV=1
26.21	2	1387.72	2773.43	0.76	35	LASVPAGGAVAVSAAPGSAAPAAGSA PAAAEEK		P02401	60S acidic ribosomal protein P2 OS=Rattus norvegicus GN=Rplp2 PE=1 SV=2
38.15	3	926.77	2777.29	-0.36	43	FSKEEIDQMFAAFP PDVTGNLDYK	[9] Oxidation (M)	P08733	Myosin regulatory light chain 2. ventricular/cardiac muscle isoform OS=Rattus norvegicus GN=Myl2 PE=1 SV=2
42.90	4	696.62	2782.46	-1.02	29	TTYLNSFSHVGTGIVHAINNVVRGAR		F1LNH3	Procollagen. type VI. alpha 2. isoform CRA_a OS=Rattus norvegicus GN=Col6a2 PE=4 SV=2
43.00	3	928.49	2782.46	-0.90	35	TTYLNSFSHVGTGIVHAINNVVRGAR		F1LNH3	Procollagen. type VI. alpha 2. isoform CRA_a OS=Rattus norvegicus GN=Col6a2 PE=4 SV=2
42.16	3	933.45	2797.34	0.27	85	SGPFGQIFRPDNFVFGQSGAGNNWA K		B4F7C2	Protein Tubb4a OS=Rattus norvegicus GN=Tubb4a PE=2 SV=1
42.16	3	933.45	2797.34	0.27	85	SGPFGQIFRPDNFVFGQSGAGNNWA K		G3V7C6	RCG45400 OS=Rattus norvegicus GN=Tubb4b PE=3 SV=1
42.16	3	933.45	2797.34	0.27	85	SGPFGQIFRPDNFVFGQSGAGNNWA K		P85108	Tubulin beta-2A chain OS=Rattus norvegicus GN=Tubb2a PE=1 SV=1
42.16	3	933.45	2797.34	0.27	85	SGPFGQIFRPDNFVFGQSGAGNNWA K		P69897	Tubulin beta-5 chain OS=Rattus norvegicus GN=Tubb5 PE=1 SV=1
35.84	3	952.15	2853.43	-0.72	56	SYTITGLQPGTDYKIHLYTLNDNAR		F1LST1	Fibronectin OS=Rattus norvegicus GN=Fn1 PE=4 SV=2
39.53	3	952.81	2855.40	-0.19	31	ALMPELEELTFDPSS AHPSLVVSASGR	[3] Oxidation (M)	A0JPQ4	Tripartite motif-containing protein 72 OS=Rattus norvegicus GN=Trim72 PE=2 SV=1

57.00	3	961.87	2882.57	0.77	28	SGAVSTGNILLSPLSVATALSALSLGAEQR		Q80ZA3	Alpha-2 antiplasmin OS=Rattus norvegicus GN=Serpinf1 PE=2 SV=1
45.67	3	965.88	2894.63	1.65	27	EILIGDKNALQNIILYHLTPGVYIGK		D3ZAF5	Periostin. osteoblast specific factor (Predicted). isoform CRA_a OS=Rattus norvegicus GN=Postn PE=4 SV=1
24.78	3	968.18	2901.52	-0.08	68	LASVPAGGAVAVSAAPGSAAPAAGSAPAAAEEKK		D4A4D5	Protein LOC100362751 OS=Rattus norvegicus GN=LOC498555 PE=3 SV=1
24.78	3	968.18	2901.52	-0.08	68	LASVPAGGAVAVSAAPGSAAPAAGSAPAAAEEKK		P02401	60S acidic ribosomal protein P2 OS=Rattus norvegicus GN=Rplp2 PE=1 SV=2
22.24	3	970.49	2908.46	0.03	32	GVAMNPVEHPFGGGNHQHIGKPSTIRR	[4] Oxidation (M)	P62919	60S ribosomal protein L8 OS=Rattus norvegicus GN=Rpl8 PE=2 SV=2
50.28	3	972.82	2915.42	-1.00	41	TVGMVAGDEESYEVFADLFDPVIKLR	[4] Oxidation (M)	B0BNC0	Ckmt2 protein OS=Rattus norvegicus GN=Ckmt2 PE=2 SV=1
42.67	3	975.82	2924.45	-0.04	86	IVEFLQSFDEITAMTGDGVNDAPALKK	[14] Oxidation (M)	E9PSX6	Sarcoplasmic/endoplasmic reticulum calcium ATPase 2 OS=Rattus norvegicus GN=Atp2a2 PE=3 SV=2
43.08	3	981.85	2942.53	-0.34	75	YRIPADVDPLTITSSLSSDGVLTVNGPR		P23928	Alpha-crystallin B chain OS=Rattus norvegicus GN=Cryab PE=1 SV=1
47.51	3	986.20	2955.56	-0.81	32	DLLQPEVAVALLEAQAGTGHIIDPATSAR		P30427	Plectin OS=Rattus norvegicus GN=Plec PE=1 SV=2
39.27	4	739.90	2955.57	-0.45	64	SLFASAENEPPVPLVGNWRPPQPIKGR		P14141	Carbonic anhydrase 3 OS=Rattus norvegicus GN=Ca3 PE=1 SV=3
39.27	3	986.20	2955.57	0.35	72	SLFASAENEPPVPLVGNWRPPQPIKGR		P14141	Carbonic anhydrase 3 OS=Rattus norvegicus GN=Ca3 PE=1 SV=3
39.27	2	1478.79	2955.57	0.49	31	SLFASAENEPPVPLVGNWRPPQPIKGR		P14141	Carbonic anhydrase 3 OS=Rattus norvegicus GN=Ca3 PE=1 SV=3
40.65	3	991.18	2970.52	1.73	44	ERLEQQVPVNQVFGQDEMIDVIGVTK		P21531	60S ribosomal protein L3 OS=Rattus norvegicus GN=Rpl3 PE=1 SV=3
32.14	3	995.50	2983.48	-1.67	46	AMHPALDNPSTMSVAPATHVSPPASSGLIK		M0R9L0	Protein Naca OS=Rattus norvegicus GN=Naca PE=4 SV=1
27.69	3	999.48	2995.41	0.36	42	LQAALDNEAGGRPAMEPGNGSLDLGGDAAGR	[15] Oxidation (M)	Q6IMY8	Heterogeneous nuclear ribonucleoprotein U OS=Rattus norvegicus GN=Hnrnpu PE=2 SV=1

42.16	3	1003.20	3006.57	0.34	88	HIADLAGNPDLVLPVPAFNVINGGSHAGNK		P15429	Beta-enolase OS=Rattus norvegicus GN=Eno3 PE=1 SV=3
42.16	4	752.65	3006.57	1.13	26	HIADLAGNPDLVLPVPAFNVINGGSHAGNK		P15429	Beta-enolase OS=Rattus norvegicus GN=Eno3 PE=1 SV=3
40.74	3	1004.88	3011.61	-7.44	47	LRSLFASAENEPPVPLVGNWRPPQPIK		P14141	Carbonic anhydrase 3 OS=Rattus norvegicus GN=Ca3 PE=1 SV=3
28.64	3	1006.17	3015.47	-1.70	61	AMHPALDNPSTMSVAPATHVSPPASSGLIK	[2] Oxidation (M)|[12] Oxidation (M)	M0R9L0	Protein Naca OS=Rattus norvegicus GN=Naca PE=4 SV=1
41.85	3	1007.87	3020.58	0.06	72	HIADLAGNPEVILPVPAFNVINGGSHAGNK		F1LTP6	Enolase OS=Rattus norvegicus PE=3 SV=2
41.85	3	1007.87	3020.58	0.06	72	HIADLAGNPEVILPVPAFNVINGGSHAGNK		M0R5J4	Uncharacterized protein OS=Rattus norvegicus PE=3 SV=1
33.89	3	1009.19	3024.55	-0.89	31	DEILPTTPISEQKGGKPEPPAMPQPVPTA		P62909	40S ribosomal protein S3 OS=Rattus norvegicus GN=Rps3 PE=1 SV=1
35.34	3	1014.14	3039.38	-12.66	28	EEQAEPDGTEVADKTAYLMGLNSSDLLK	[19] Oxidation (M)	G3V6D8	Myosin-3 OS=Rattus norvegicus GN=Myh3 PE=4 SV=1
35.34	3	1014.14	3039.38	-0.69	59	EEQAEPDGTEEADKSAYLMGLNSADLLK	[19] Oxidation (M)	P02564	Myosin-7 OS=Rattus norvegicus GN=Myh7 PE=2 SV=2
35.34	3	1014.14	3039.38	-0.69	59	EEQAEPDGTEEADKSAYLMGLNSADLLK	[19] Oxidation (M)	G3V8B0	Myosin-7 OS=Rattus norvegicus GN=Myh7 PE=4 SV=1
33.89	3	1027.50	3079.47	0.56	42	NADLHSGTELTLDNSMTPPSDPALPLETK	[16] Oxidation (M)	Q5M7W5	Microtubule-associated protein 4 OS=Rattus norvegicus GN=Map4 PE=1 SV=1
49.82	3	1039.86	3116.57	-0.53	38	TIESILEPVAQQISHLVIMHEEGEVDGK	[19] Oxidation (M)	R9PXU6	Vinculin OS=Rattus norvegicus GN=Vcl PE=4 SV=1
45.73	3	1042.84	3125.51	-0.26	36	TVSGNTVEFELHDLEPATEYTLSVFAEK		M0RA80	Protein Tnc OS=Rattus norvegicus GN=Tnc PE=4 SV=1
49.58	3	1049.86	3146.55	-0.14	52	ALIDMYAEGVADLDEIVLHYPYIPPGEK	[5] Oxidation (M)	M0RDI1	Glutathione S-transferase OS=Rattus norvegicus PE=3 SV=1
38.50	3	1051.23	3150.67	-0.07	47	AFLADPSAFAAAAPVAAATTAAPAAAAAPAKVEAK		P19945	60S acidic ribosomal protein P0 OS=Rattus norvegicus GN=Rplp0 PE=1 SV=2
31.80	3	1052.17	3153.49	1.39	29	ETVEPPEDEPPGSLGSVDENRETLTSLEK		D3ZWJ2	Nestin OS=Rattus norvegicus GN=Nes PE=3 SV=2

44.35	3	1053.22	3156.63	-0.56	62	ELASQPDVDGFLVGGASLKPEFVDIINAKQ		Q6SA19	Triosephosphate isomerase OS=Rattus norvegicus GN=LOC500959 PE=2 SV=1
39.12	3	1061.88	3182.60	-1.11	59	TTGIVMDSGDGVTHTVPIYEGYALPHAILR		P60711	Actin. cytoplasmic 1 OS=Rattus norvegicus GN=Actb PE=1 SV=1
40.99	3	1065.24	3192.71	-1.56	44	KYTLPPGVDPTLVSSSLSPEGTLTVEAPLPK		G3V913	Heat shock 27kDa protein 1 OS=Rattus norvegicus GN=Hspb1 PE=3 SV=1
38.50	4	799.91	3195.60	-1.02	38	TTGIVLDSGDGVTHNVPIYEGYALPHAIMR		P68035	Actin. alpha cardiac muscle 1 OS=Rattus norvegicus GN=Actc1 PE=2 SV=1
38.50	4	799.91	3195.60	-1.02	38	TTGIVLDSGDGVTHNVPIYEGYALPHAIMR		P68136	Actin. alpha skeletal muscle OS=Rattus norvegicus GN=Acta1 PE=1 SV=1
38.50	3	1066.21	3195.60	0.42	106	TTGIVLDSGDGVTHNVPIYEGYALPHAIMR		P68035	Actin. alpha cardiac muscle 1 OS=Rattus norvegicus GN=Actc1 PE=2 SV=1
38.50	3	1066.21	3195.60	0.42	106	TTGIVLDSGDGVTHNVPIYEGYALPHAIMR		P68136	Actin. alpha skeletal muscle OS=Rattus norvegicus GN=Acta1 PE=1 SV=1
37.98	3	1067.21	3198.60	-0.27	64	TTGIVMDSGDGVTHTVPIYEGYALPHAILR	[6] Oxidation (M)	P60711	Actin. cytoplasmic 1 OS=Rattus norvegicus GN=Actb PE=1 SV=1
36.76	3	1067.56	3199.67	-0.32	40	IEAPKETLATSPEGVTAMPLEIPPSPAAPASK		M0R9L0	Protein Naca OS=Rattus norvegicus GN=Naca PE=4 SV=1
36.58	4	803.91	3211.59	-0.93	35	TTGIVLDSGDGVTHNVPIYEGYALPHAIMR	[29] Oxidation (M)	P68035	Actin. alpha cardiac muscle 1 OS=Rattus norvegicus GN=Actc1 PE=2 SV=1
36.58	4	803.91	3211.59	-0.93	35	TTGIVLDSGDGVTHNVPIYEGYALPHAIMR	[29] Oxidation (M)	P68136	Actin. alpha skeletal muscle OS=Rattus norvegicus GN=Acta1 PE=1 SV=1
36.58	3	1071.54	3211.60	-0.20	105	TTGIVLDSGDGVTHNVPIYEGYALPHAIMR	[29] Oxidation (M)	P68035	Actin. alpha cardiac muscle 1 OS=Rattus norvegicus GN=Actc1 PE=2 SV=1
36.58	3	1071.54	3211.60	-0.20	105	TTGIVLDSGDGVTHNVPIYEGYALPHAIMR	[29] Oxidation (M)	P68136	Actin. alpha skeletal muscle OS=Rattus norvegicus GN=Acta1 PE=1 SV=1

16.61	4	810.63	3238.51	-2.25	43	RPQYSNPPVQGEVMEGADNQGAGEQGRPVR	[14] Oxidation (M)	F1LPL7	Protein LOC100912427 OS=Rattus norvegicus GN=LOC100912427 PE=4 SV=1
16.54	3	1080.51	3238.51	-2.03	78	RPQYSNPPVQGEVMEGADNQGAGEQGRPVR	[14] Oxidation (M)	F1LPL7	Protein LOC100912427 OS=Rattus norvegicus GN=LOC100912427 PE=4 SV=1
48.12	3	1089.18	3264.53	-1.01	83	MYPTDDSAPLPAGTLDQPLFEVIQEAMQR	[1] Oxidation (M)|[27] Oxidation (M)	D4AEH9	Amylo-1. 6-glucosidase. 4-alpha-glucanotransferase (Glycogen debranching enzyme. glycogen storage disease type III) (Predicted). isoform CRA_a OS=Rattus norvegicus GN=Agl PE=4 SV=1
45.00	3	1093.54	3277.61	-0.33	74	LLVVYPWTQRYFDSFGDLSSASAIMGNPK	[25] Oxidation (M)	P02091	Hemoglobin subunit beta-1 OS=Rattus norvegicus GN=Hbb PE=1 SV=3
47.59	3	1095.22	3282.65	-1.12	36	LGTTRAPSYGAGELLDFSLADAVNQEFLATR		Q6P725	Desmin OS=Rattus norvegicus GN=Des PE=2 SV=1
39.12	3	1112.22	3333.64	-1.88	44	EQRVPAEALDNLEGGALEVPVAQSMPEVTER		D3ZWJ2	Nestin OS=Rattus norvegicus GN=Nes PE=3 SV=2
36.41	3	1116.17	3345.49	-0.54	44	LYQSAGGMPGGMPGGFPGGGAPPSGGASSGPTIEEVD		P63018	Heat shock cognate 71 kDa protein OS=Rattus norvegicus GN=Hspa8 PE=1 SV=1
44.68	3	1116.23	3345.67	0.69	32	IVKPNGEKPDEFESGISQALLELEMNSDLK	[25] Oxidation (M)	B5DEL9	RCG62292. isoform CRA_a OS=Rattus norvegicus GN=Rps7 PE=2 SV=1
37.03	3	1120.49	3358.45	0.55	58	APKPEDIDEEDDDVPDLVENFDEASKNEAN		D4A3I4	Protein Btf3l4 OS=Rattus norvegicus GN=Btf3l4 PE=4 SV=1
48.26	3	1120.58	3358.72	0.85	52	EGISQEALHTQMLTAVQEISHLIEPLASAAR	[12] Oxidation (M)	G3V852	Protein Tln1 OS=Rattus norvegicus GN=Tln1 PE=4 SV=1
16.69	3	1189.22	3564.65	-0.39	44	AEPEKSEGAAEEQPEPAPAPEQEAAAPGPAAGGEAPK		Q05175	Brain acid soluble protein 1 OS=Rattus norvegicus GN=Basp1 PE=1 SV=2
17.70	3	1192.55	3574.64	-6.65	42	SSGPPPPSGSSGSEAAAGAAAPASQHPATGTGAVQTEAMK		Q5M9G3	Caprin-1 OS=Rattus norvegicus GN=Caprin1 PE=1 SV=2
42.34	3	1192.92	3575.75	1.05	50	TVSGNTVEFELHDLEPATEYTLSVFAEKGHQK		M0RA80	Protein Tnc OS=Rattus norvegicus GN=Tnc PE=4 SV=1

40.68	3	1199.91	3596.71	0.20	70	ALSMFSEDFGSFMLPHSEPLTFPARPGGQGNIK	[4] Oxidation (M)|[13] Oxidation (M)	B5DFG4	Heat shock 27kD protein family. member 7 (Cardiovascular) OS=Rattus norvegicus GN=Hspb7 PE=2 SV=1
44.09	3	1215.61	3643.80	-0.02	52	SMTEQEQQQLIDDHFLFDKPVSPLLLASGMAR		P00564	Creatine kinase M-type OS=Rattus norvegicus GN=Ckm PE=1 SV=2
29.70	3	1216.57	3646.70	-0.73	61	TLDPEHLGQGGVQREDVPAADLSDQVPDTDSETR		M0RBF1	Complement C3 OS=Rattus norvegicus GN=C3 PE=4 SV=1
41.60	4	919.96	3675.79	0.04	42	SMTEQEQQQLIDDHFLFDKPVSPLLLASGMAR	[2] Oxidation (M)|[30] Oxidation (M)	P00564	Creatine kinase M-type OS=Rattus norvegicus GN=Ckm PE=1 SV=2
41.60	3	1226.27	3675.79	0.81	76	SMTEQEQQQLIDDHFLFDKPVSPLLLASGMAR	[2] Oxidation (M)|[30] Oxidation (M)	P00564	Creatine kinase M-type OS=Rattus norvegicus GN=Ckm PE=1 SV=2
48.07	3	1249.63	3745.88	-0.44	71	ALIDMYAEGVADLDEIVLHYPYIPPGEKEASLAK	[5] Oxidation (M)	M0RDI1	Glutathione S-transferase OS=Rattus norvegicus PE=3 SV=1
47.59	3	1253.97	3758.89	-0.58	29	GTGGVDTAAVGAVFDISNADRLGSSEVEQVQLVVDGVK		P00564	Creatine kinase M-type OS=Rattus norvegicus GN=Ckm PE=1 SV=2
45.00	3	1254.96	3761.85	0.65	30	EVTVPLEAAGPLVSDMTAILETEMTLGGGTATPTETK	[16] Oxidation (M)|[24] Oxidation (M)	Q5M7W5	Microtubule-associated protein 4 OS=Rattus norvegicus GN=Map4 PE=1 SV=1
25.02	3	1289.25	3864.74	-0.45	71	ALEMMAGQTTDAVPSKESEGSPDTDAAPGPDTDVTLTK	[4] Oxidation (M)|[5] Oxidation (M)	Q5M7W5	Microtubule-associated protein 4 OS=Rattus norvegicus GN=Map4 PE=1 SV=1
44.76	3	1291.27	3870.79	2.24	110	GGGGGGGGGLGGGLGNVLGGLISGAAGGGGGGGGGGMGLGGGGGGGGTAMR	[39] Oxidation (M)|[52] Oxidation (M)	Q64537	Calpain small subunit 1 OS=Rattus norvegicus GN=Capns1 PE=1 SV=3
36.17	3	1300.22	3897.64	0.40	92	GFGSFRFPSGNQGGAGPSQGSGGGTGGNVYTEDNDDDLYG		P46462	Transitional endoplasmic reticulum ATPase OS=Rattus norvegicus GN=Vcp PE=1 SV=3
48.36	3	1306.38	3916.13	-0.18	66	EGNASGVSLLEALDTILPPTRPTDKPLRLPLQDVYK		P62632	Elongation factor 1-alpha 2 OS=Rattus norvegicus GN=Eef1a2 PE=2 SV=1

30.95	3	1307.65	3919.92	0.60	73	SPAASGAPQAPAPAALLAGSPGGDAA PGPAPASSAPAGSEDAEKK		Q62764	Y-box-binding protein 3 OS=Rattus norvegicus GN=Ybx3 PE=2 SV=1
44.50	3	1323.34	3967.01	0.00	55	AQSAPPEAAVLPLSPLSAPAASPSPFQ DGGAPSPAPSIFNR		D3ZZ68	Protein Synpo2l OS=Rattus norvegicus GN=Synpo2l PE=4 SV=1
29.62	3	1330.27	3987.78	1.31	76	QQEGEASSQDMTA QVTSPSGKTEAAEI VEGEDSAYSVR	[11] Oxidation (M)	D3ZHA0	Protein Flnc OS=Rattus norvegicus GN=Flnc PE=4 SV=1
50.03	4	1019.54	4074.13	-0.41	43	VGPTVNDVQLTGLLPNTEYEVTVQAV LHDLTSEPAKAR		F1LQC3	Collagen alpha-1(XII) chain (Fragment) OS=Rattus norvegicus GN=Col12a1 PE=4 SV=2
50.03	4	1019.54	4074.13	-0.41	43	VGPTVNDVQLTGLLPNTEYEVTVQAV LHDLTSEPAKAR		D3Z9F8	Collagen alpha-1(XII) chain OS=Rattus norvegicus GN=Col12a1 PE=4 SV=2
42.51	3	1361.73	4082.17	0.32	118	YRLPPGVDPAAVTSALSPEGVLSIQAT PASAQASLPSPPAAK		P97541	Heat shock protein beta-6 OS=Rattus norvegicus GN=Hspb6 PE=1 SV=1
45.98	3	1427.29	4278.85	0.54	64	MLGQNPTPEELQE MIDEVDEDGSGTV DFDEFLVMMVR	[1] Oxidation (M)|[14] Oxidation (M)|[34] Oxidation (M)|[35] Oxidation (M)	Q4PP99	Cardiac troponin C OS=Rattus norvegicus GN=Tnnc1 PE=2 SV=1
49.72	4	1112.57	4446.25	-0.06	27	AADEDELKEIAFDSS LVFIPAEFRPAPLQS MLPSLMAPLR	[31] Oxidation (M)|[36] Oxidation (M)	D4A111	Protein Col6a3 OS=Rattus norvegicus GN=Col6a3 PE=4 SV=2
47.71	4	1140.29	4557.13	-0.88	82	EAMQQADDWLGV PQVIAPEEIVDPNV DEHSVMTYLSQFPK	[3] Oxidation (M)|[32] Oxidation (M)	D3ZHA0	Protein Flnc OS=Rattus norvegicus GN=Flnc PE=4 SV=1
45.85	4	1233.64	4930.53	1.41	45	HIADLAGNPEVILPV PAFNVINGGSHAG NKLAMQEFMILPV GASSFR	[33] Oxidation (M)|[37] Oxidation (M)	F1LTP6	Enolase OS=Rattus norvegicus PE=3 SV=2
45.85	4	1233.64	4930.53	1.41	45	HIADLAGNPEVILPV PAFNVINGGSHAG	[33] Oxidation (M)|[37] Oxidation (M)	M0R5J4	Uncharacterized protein OS=Rattus norvegicus PE=3 SV=1

						NKLAMQEFMILPV GASSFR			
50.71	4	1262.38	5045.50	0.67	29	AIAYGPGIEPQGNTVLQPAHFTVQTV DAGVGEVLVYIEDPEGHTEEAK		D3ZHA0	Protein Flnc OS=Rattus norvegicus GN=Flnc PE=4 SV=1
48.87	4	1294.40	5173.59	-0.44	49	KAIAYGPGIEPQGNTVLQPAHFTVQTV DAGVGEVLVYIEDPEGHTEEAK		D3ZHA0	Protein Flnc OS=Rattus norvegicus GN=Flnc PE=4 SV=1
49.75	4	1298.65	5190.55	0.46	32	NLPGIAPLPMPSDF LPSFPLVPEGSSAAS AGEPLSSLPAMGPP SDPVMTTAK	[10] Oxidation (M)|[40] Oxidation (M)|[48] Oxidation (M)	F6T071	Golgi reassembly-stacking protein 2 (Fragment) OS=Rattus norvegicus GN=Gorasp2 PE=4 SV=1
43.00	5	1307.90	6534.44	-0.51	34	SEDPASPVSSLSVPA AHKPFPAPSMSTTL GIPVSPLPATEGLQ NLPISVNAGAPVSP AQAGLPIRK	[25] Oxidation (M)	M0R9L0	Protein Naca OS=Rattus norvegicus GN=Naca PE=4 SV=1

Supplementary table 5. Comparison of ROC analysis with 6000 randomized selected spectra and all spectra.

Centroid [m/z]	± [Da]	6000 selected spectra		All Spectra	
		TAM-MSC-TX vs TAM control [AUC]	TM-MSC-TX vs TM control [AUC]	TAM-MSC-TX vs TAM control [AUC]	TM-MSC-TX vs TM control [AUC]
937.556	0.156	0.716742	0.596285	0.712015	0.595317
979.579	0.156	0.368217	0.167758	0.36131	0.172053
1.172.574	0.156	0.101009	0.256582	0.0969135	0.250509
1.323.707	0.156	0.181074	0.25901	0.177682	0.251923
1.328.641	0.156	0.127956	0.240739	0.12524	0.231868
1.390.746	0.156	0.117929	0.314035	0.110563	0.309775
1.396.764	0.156	0.711124	0.339262	0.707524	0.33479
1.489.896	0.156	0.731956	0.624215	0.72771	0.620058
1.668.807	0.156	0.978855	0.697584	0.977008	0.695899
1.701.976	0.156	0.19095	0.221345	0.191666	0.216121
1.740.945	0.156	0.854172	0.744556	0.851918	0.737561
1.782.988	0.156	0.689368	0.546025	0.687611	0.537935
